# Time-resolved pathogenic gene expression analysis of the plant pathogen *Xanthomonas oryzae* pv. *oryzae*

**DOI:** 10.1186/s12864-016-2657-7

**Published:** 2016-05-10

**Authors:** Seunghwan Kim, Yong-Joon Cho, Eun-Sung Song, Sang Hee Lee, Jeong-Gu Kim, Lin-Woo Kang

**Affiliations:** Genomics Division, National Academy of Agricultural Science (NAAS), Rural Development Administration (RDA), Jeonju, 54874 Korea; Chunlab, Inc., Seoul National University, Seoul, 08826 Korea; Department of Biological Sciences, National Leading Research Laboratory of Drug Resistance Proteomics, Myongji University, 116 Myongjiro, Yongin, Gyeonggido 17058 Korea; Department of Biological Sciences, Konkuk University, 1 Hwayang dong, Gwangjin-gu, Seoul 05029 Korea

**Keywords:** Pathogenicity, Plant–pathogen interactions, RNA-Seq, Time-resolved genome-wide gene expression, *Xanthomonas oryzae* pv. *oryzae*

## Abstract

**Background:**

Plant-pathogen interactions at early stages of infection are important to the fate of interaction. *Xanthomonas oryzae* pv. *oryzae* (*Xoo*) causes bacterial blight, which is a devastating disease in rice. Although in vivo and in vitro systems have been developed to study rice-*Xoo* interactions, both systems have limitations. The resistance mechanisms in rice can be better studied by the in vivo approach, whereas the in vitro systems are suitable for pathogenicity studies on *Xoo*. The current in vitro system uses minimal medium to activate the pathogenic signal (expression of pathogenicity-related genes) of *Xoo*, but lacks rice-derived factors needed for *Xoo* activation. This fact emphasizes the need of developing a new in vitro system that allow for an easy control of both pathogenic activation and for the experiment itself.

**Results:**

We employed an in vitro system that can activate pathogenicity-related genes in *Xoo* using rice leaf extract (RLX) and combined the in vitro assay with RNA-Seq to analyze the time-resolved genome-wide gene expression of *Xoo*. RNA-Seq was performed with samples from seven different time points within 1 h post-RLX treatment and the expression of up- or downregulated genes in RNA-Seq was validated by qRT-PCR. Global analysis of gene expression and regulation revealed the most dramatic changes in functional categories of genes related to inorganic ion transport and metabolism, and cell motility. Expression of many pathogenicity-related genes was induced within 15 min upon contact with RLX. *hrpG* and *hrpX* expression reached the maximum level within 10 and 15 min, respectively. Chemotaxis and flagella biosynthesis-related genes and cyclic-di-GMP controlling genes were downregulated for 10 min and were then upregulated. Genes related to inorganic ion uptake were upregulated within 5 min. We introduced a non-linear regression fit to generate continuous time-resolved gene expression levels and tested the essentiality of the transcriptionally upregulated genes by a pathogenicity assay of lesion length using single-gene knock-out *Xoo* strains.

**Conclusions:**

The in vitro system combined with RNA-Seq generated a genome-wide time-resolved pathogenic gene expression profile within 1 h of initial rice-*Xoo* interactions, demonstrating the expression order and interaction dependency of pathogenic genes. This combined system can be used as a novel tool to study the initial interactions between rice and *Xoo* during bacterial blight progression.

**Electronic supplementary material:**

The online version of this article (doi:10.1186/s12864-016-2657-7) contains supplementary material, which is available to authorized users.

## Background

Plants and pathogens have competed with each other throughout evolutionary history. Initial plant-pathogen interactions are crucial in determining the fate of infection to cause disease or to elicit hypersensitive reactions in resistant plants. Plants resist infection by a two-tiered innate immune system of pathogen-associated molecular pattern-triggered immunity and effector-triggered immunity [1]. Pathogen-derived pathogen-associated molecular patterns and plant-derived damage-associated molecular patterns are recognized by plants and activate plant resistance. On the contrary, the pathogen tries to avoid plant resistance by activating its pathogenic system, represented by effector secretions.

Rice (*Oryza sativa* L.) has contributed significantly to global food security in the Green Revolution of the 1960s and still remains the most widely consumed staple food globally. As the world population rapidly grows, rice production needs to increase by at least 25 % by 2030 under more severe environmental stresses like climate change and disease pressures [2]. Among rice diseases, bacterial blight is a destructive disease that results in severe losses, ranging from 10 to 20 % and up to 50 to 70 % in Asian countries [3, 4]. Bacterial blight is a vascular disease that causes tannish gray to white lesions along the leaf veins resulting in rapid drying of severely infected leaves [3]. The gram-negative plant pathogen *Xanthomonas oryzae* pv. *oryzae* (*Xoo*) is the causative agent of bacterial blight on rice [5]. Recently, qualitative and pathogen race-specific resistance to *Xoo* has been conferred in plants by introduction of major disease resistance genes [6]. In addition to *Xoo* on rice, pathogens of the genus *Xanthomonas* infect nearly 400 different plant hosts, including rice, cotton, soybean, oil-rape, citrus and banana, which are economically important crops [7].

In *Xoo*, many virulence genes have been identified, including those related to hypersensitive responses and pathogenicity (*hrp*), bacterial toxins and effectors of avirulence (*avr*), plant cell wall degradation, extracellular polysaccharide synthesis and secretion, and bacterial motility and chemotaxis [8, 9]. For instance, the type 3 secretion system (T3SS) encoded by *hrp* genes enables *Xoo* to inject T3 effectors into the host rice cells [10]. The OmpR-type response regulator HrpG is known to control the expression of genome-wide pathogenicity-related regulons, including *hrp* genes, T3 effectors and virulence genes, through another regulator known as HrpX [11]. Both *tonB* and *bfr* genes are essential for competing for iron uptake with the host [12, 13]. Chemotaxis and motility-related genes are also known to be controlled by *hrp* genes such as *hrpG* and *hrpX* [14]. A second messenger such as cyclic-di-GMP affects a wide array of pathogenic cellular functions, including type III secretion and virulence [15–20]. The two-component regulatory systems of PhoB-PhoR and PhoP-PhoQ are also closely involved in pathogenicity signaling [21].

It is known that *hrp* genes are only induced when phytobacteria are grown in the plant leaf apoplast or in close contact with plant cells [22, 23]. In order to activate the pathogenicity of the phytobacteria *Xanthomonas* represented by *hrp* gene expression, synthetic minimal medium such as XOM2 and XVM2 was used, which is known to mimic the plant apoplast environment to activate the pathogenic signals [24–27]. *Xoo* can be cultured in artificial media but the minimal medium condition is required to activate *hrp*-related gene expression. It is difficult to activate pathogenic signals in *Xoo* at the desired time point using the synthetic minimal medium. Mutant *Xoo* strains are usually used to study the pathogenic role of target genes by the comparison with wild-type *Xoo* strains. In this study, we used an in vitro assay system that activates *Xoo* pathogenicity by treating *Xoo* cells with rice leaf extracts (RLX) [28, 29]. The in vitro assay system using RLX could induce the expression and secretion of putative effectors of XoAvrBs2 and Xo2276 (AvrBs3-type TAL effector-like protein) and the T3SS-dependent secretions of XoAvrBs2 and Xo2276 were confirmed at 4 h after RLX treatment. The in vitro assay system with RLX enables activation of the pathogenic signal in *Xoo* at any specific time. We analyzed time-resolved genome-wide gene expression of *Xoo* by combining the in vitro assay system with RNA-Seq, allowing us to compare pathogenic gene expression patterns in the same genetic background without using single-gene knockout mutants. The time-resolved transcriptome data of *Xoo* were verified by qRT-PCR.

## Results

### In vitro assay system and RNA-Seq

**Fig. 1:**
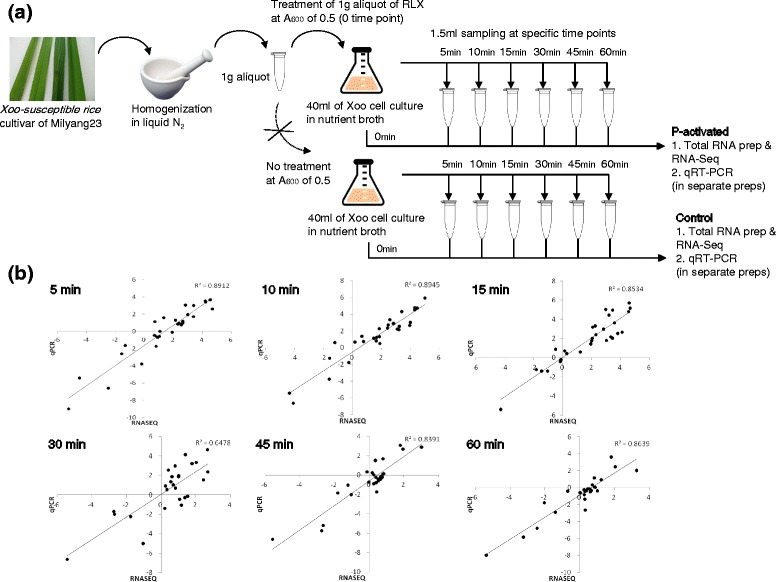
**a** Schematic representation of the in vitro assay system and sampling for RNA-Seq and qRT-PCR. **b** Comparison of transcription levels by RNA-Seq and qRT-PCR. The relative transcription levels for 28 randomly selected genes from RNA-Seq data were verified by qRT-PCR. The log_2_-scaled values from qRT-PCR were plotted against the log_2_-scaled values from RNA-Seq data. The correlation coefficient (R^2^) of the datasets was between 0.65 and 0.89

We combined an in vitro assay system with RNA-Seq to analyze genome-wide pathogenic gene expression of *Xoo* in a time-dependent manner (Fig. 1a). The in vitro assay system consists of fresh RLX preparation by grinding the leaves of a *Xoo*-susceptible rice cultivar (Milyang23) in liquid nitrogen and addition of RLX at the mid-exponential phase of *Xoo* cell culture in nutrient broth. Samples were harvested for RNA-Seq from the RLX-treated (Pathogenicity-activated; P-activated) and untreated (control) *Xoo* cells at specific time points. RNA-Seq data were verified by qRT-PCR in separate biological replicates.

The first hour after RLX treatment was examined by RNA-Seq using seven different time points of 0, 5, 10, 15, 30, 45 and 60 min (Additional file 1: Table S1). Most of the pathogenicity-related genes were upregulated within the first 30 min. To validate RNA-Seq data of the first hour, qRT-PCR was performed with 28 arbitrarily selected up- or downregulated *Xoo* genes from the RNA-Seq data (Additional file 2: Table S2). Significant correlation was observed between the log_2_-fold changes in the expression levels determined by RNA-Seq and qRT-PCR (Fig. 1b).

### Time-resolved gene expression patterns on Clusters of Orthologous Groups (COGs)

RNA-Seq experiments were performed with total RNA extracts from both P-activated and control *Xoo* cells. The RNA-Seq data covered more than 4125 ORFs of *Xoo*, which constitutes 89 % of all 4637 annotated protein-coding ORFs (Additional file 3: Table S3). For each gene, two different conditions (P-activated and control) and seven time-dependent expression levels of RNA were examined. For each gene in the whole genome, pathogenicity condition, gene expression level and time resulted in three-dimensional data. In control cells, the expression levels of most pathogenicity-related genes remained unchanged. In order to compare time-dependent transcriptional expression of all genes, we set the expression level at zero time point as the reference level of 1 for each gene. Accordingly, expression levels at all time-points were divided by that at the zero time point and uniformly started from 1 at the zero time point, which simplifies the comparison of time-dependent changes in gene expression levels.

RNA-Seq data showed that in P-activated *Xoo* cells, 305, 325, 300, 291, 204 and 224 genes were upregulated, whereas 110, 173, 148, 179, 210 and 205 genes were downregulated at each of the 6 time points, respectively, with an RPKM threshold of 2.0 and a difference filter of 2.0 (Additional file 4: Figure S1). The number of upregulated genes reached the maximum of 325 genes at 10 min point and decreased to 204 genes at 45 min point. The number of downregulated genes slowly increased until 1 h in a time-dependent manner.

**Fig. 2:**
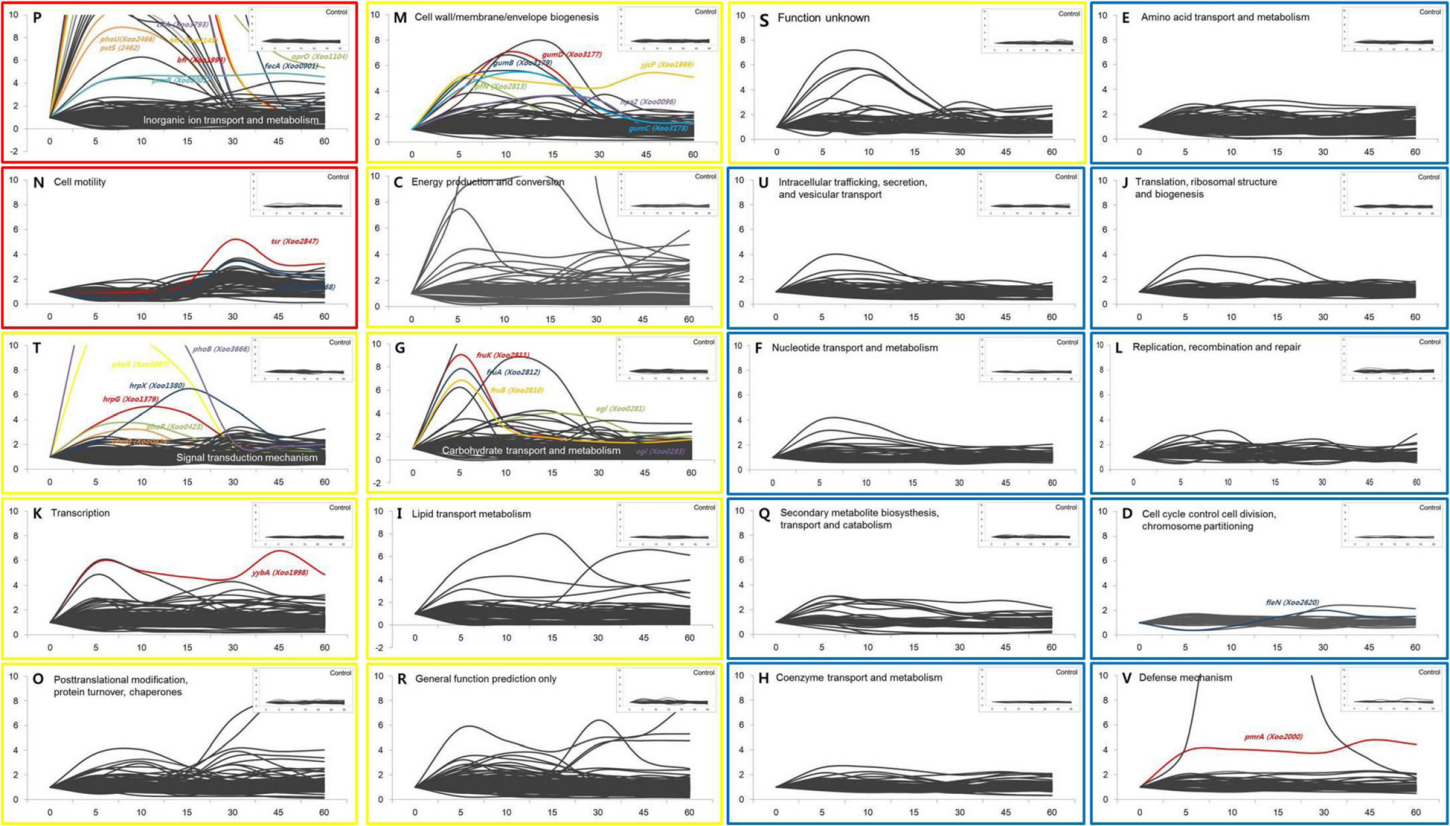
Gene expression patterns of COG categories. Two functional categories of genes showing the most changes in gene expression are represented as red boxes. Nine functional categories of genes that showed moderate changes in gene expression are represented as yellow boxes. The other nine functional categories of genes that showed little changes in gene expression are represented as blue boxes. Inset figure at the upper right of each functional category of genes indicates the control (untreated). Colored lines and text indicate the representative genes of each COG category. Y-axis represents fold-change and is fixed at the value of 10

In order to study the global gene expression pattern, we superimposed time-dependent gene expression patterns of genes according to functional categories in the clusters of orthologous groups (COG) database (Fig. 2 and Additional file 5: Table S4), with a further grouping into three classes (red, yellow and blue) depending on the observed pattern. The biggest changes in gene expression of *Xoo* cells upon RLX treatment were observed in the categories of inorganic ion transport and metabolism (P) and cell motility (N) further classified as red class (red boxes in Fig. 2). In category P, many genes were found to be upregulated from 5 min by more than 5-fold. In category N, many genes were downregulated until 15 min and were then upregulated from 30 min. Class yellow grouped genes that showed initial moderate upregulation followed by return to the original level of gene expression. This class had genes belonging to other 9 categories (yellow boxes in Fig. 2) namely of signal transduction mechanisms (T), transcription (K), posttranslational modification, protein turnover, chaperones (O), cell wall/membrane/envelope biogenesis (M), energy production and conversion (C), carbohydrate transport and metabolism (G), lipid transport metabolism (I), general function prediction only (R), and function unknown (S). In category T, key pathogenicity regulator genes such as *hrpG*, hrpX and two-component system (TCS) genes including *phoB-phoR* and *phoP-phoQ* were upregulated from 5 min and returned to the original expression level at 1 h time point. In category M, *gum* genes were upregulated from 5 to 10 min and in G, sugar-transporting genes like *fruK*, *fruA* and *fruB* were upregulated from 5 min. Whereas the last grouping class, blue, contains genes belonging to other nine categories which expression levels showed little changes or stayed at almost the same level as the zero time level, with the exception of only a few genes.

### *hrp* genes

**Fig. 3:**
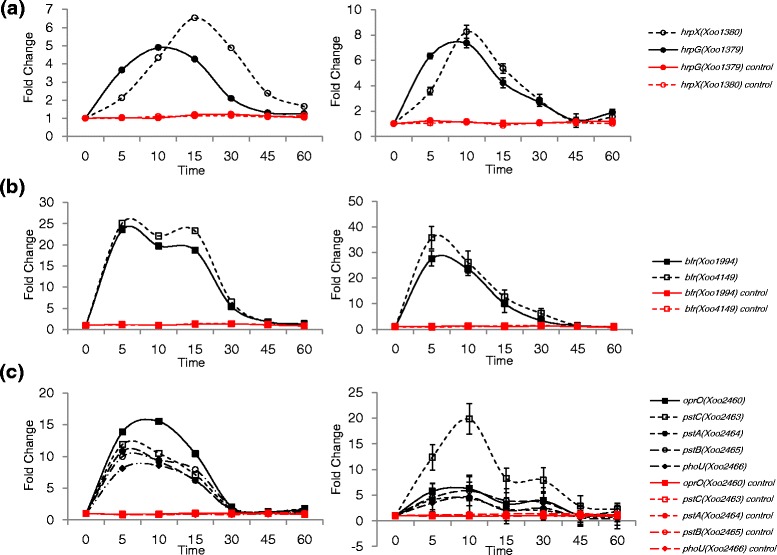
Time-resolved expression patterns of *hrpG* and *hrpX* genes, iron-uptake bacterioferritin genes and P*i*-uptake genes. **a** RNA-Seq expression data of *hrpG* and *hrpX* (*left*) and qRT-PCR expression data of *hrpG* and *hrpX* (*right*). **b** RNA-Seq expression data of two bacterioferritin genes, *Xoo1994* and *Xoo4149* (*left*) and qRT-PCR expression data of *Xoo1994* and *Xoo4149* (*right*). **c** RNA-Seq expression data of *oprO, pstC, pstA, pstB and phoU* (*left*) and qRT-PCR expression data of *oprO, pstC, pstA, pstB and phoU* (*right*)*.* The unit of time is min. Red lines indicate the expression levels of genes in the control (untreated) *Xoo* cells. Y-axis represents fold-change

The *hrp* genes are key global regulators of pathogenicity and play important roles in T3SS and virulence [10]. Our in vitro RNA-Seq data revealed that the expression of *hrpG* was induced within 5 min, and maximum expression was observed at 10 min. *hrpX* expression also started to increase within 5 min but at a slower rate and continued to increase until 15 min when maximum expression was achieved (Fig. 3a). The RNA-Seq result was confirmed by qRT-PCR. Most *hrp* genes were up-regulated within 1 h (Additional file 6: Figure S2). In the other in vitro system using the *hrp* genes-inducing minimum medium, the expression of *hrp* genes was also upregulated similarly but mostly at higher levels [30] (Additional file 7: Table S5).

### Inorganic ion transport and metabolism-related genes

In 20 categories of COG, P showed the largest number of upregulated genes. Iron-uptake related genes and inorganic phosphate-uptake related genes are the representative upregulated genes in P. In most living organisms, iron is an essential nutrient required for many important biological processes [12]. Pathogenic bacteria need to compete with their host to obtain iron; for instance, by producing and secreting bacterioferritins and siderophores in the host, which are high-affinity Fe (III) scavengers [13]. The bacterioferritin (*bfr*) genes are involved in storing ferric iron after uptake [31]. The expression of *bfr* genes *Xoo1994* and *Xoo4149* were upregulated at 5 min (Fig. 3b).

Iron-loaded siderophores are transported back into bacterial cells through an outer membrane receptor, which is regulated by other membrane-associated proteins such as TonB [32, 33]. Our results showed that *Xoo2114* (among the seven TonB proteins of *Xoo*) was upregulated at 5 min (Additional file 8: Figure S3A). Among 24 TonB-dependent receptors, *fecA* (*Xoo0901*) and *cirA* (*Xoo3793*) were upregulated from 5 min; notably, *fecA* (*Xoo0901)* expression increased by more than 80-fold at 10 min (Additional file 8: Figure S3B). On the contrary, *iroN* (*Xoo0394*), *iroN* (*Xoo1784*) and *cirA* (*Xoo4431*) were downregulated (Additional file 8: Figure S3C)*. fyuA* (*Xoo1099*) was upregulated at 5 min and downregulated afterwards. Among the *exb* genes, also involved in ferric iron uptake [34], only *tolR* (*Xoo1666*) showed upregulation from 5 to 15 min (Additional file 8: Figure S3D). The iron-uptake genes, highly expressed before the 5 min time point, can be good candidates as early initiators to activate the pathogenicity signal in *Xoo*.

Inorganic phosphate (P*i*) is another essential nutrient depleted by the host as a protective mechanism against bacterial infection. However, this depletion is sensed by some pathogens and prompts the expression of virulence genes. The P*i*-specific transport operon, *pstSCAB*-*phoU*, was upregulated from 5 min; *pstC* (*Xoo2463* encoding the phosphate ABC transporter permease), *pstA* (*Xoo2464* encoding the phosphate ABC transporter permease), *pstB* (*Xoo2465* encoding the phosphate transporter ATP-binding protein), and *phoU* (*Xoo2466* encoding the phosphate uptake regulator) were upregulated at 5 min by 12, 11, 10 and 8-fold, respectively (Fig. 3c). The *pstSCAB*-*phoU* genes are activated by the two-component system of PhoB-PhoR that detects and responds to changes in environmental P*i* concentrations [35]. Expression of *phoB* (*Xoo3666*) and *phoR* (*Xoo3667*) was also upregulated by 17- and 12-fold at 5 min, respectively (Additional file 9: Figure S4A). *oprO* (*Xoo2460* encoding the polyphosphate-selective porin O) is found in the same operon and its expression was upregulated until 15 min (Fig. 3c). Among *ppk* and *ppx* genes involved in polyphosphate metabolism, *ppk* (*Xoo3668*) was upregulated within 30 min by 2-fold (Additional file 9: Figure S4B).

Another two-component system of PhoP-PhoQ system regulates the expression of hundreds of genes encoding virulence proteins with various properties, including intracellular survival, invasion and lipid A structure via response to the magnesium ion [36]. *phoP* (*Xoo0423*) and *phoQ* (*Xoo0424*) were upregulated by 3-fold at 5 min (Additional file 9: Figure S4C).

### Chemotaxis and motility-related genes

**Fig. 4:**
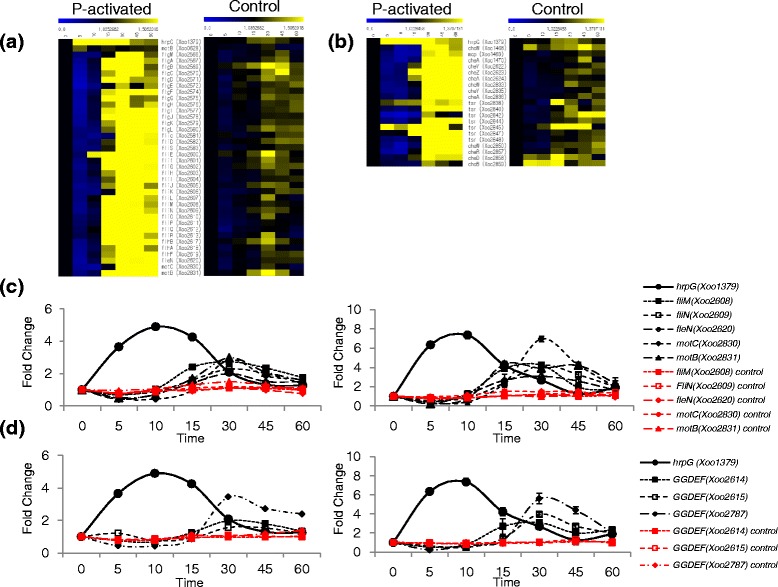
Time-resolved expression of chemotaxis and motility-related genes and the second messenger of cyclic-di-GMP related genes. **a** Time-resolved heat map of the flagella biosynthesis-related genes in the pathogenicity-activated (P-activated) *Xoo* and the control *Xoo*. The expression level of each gene is shown as color change according to the fold change. The separate upper bar indicates the relation between color and the fold change value of gene expression; blue color represents most downregulated genes and yellow color represents the most upregulated genes in the column of comparison. **b** Time-resolved heat map of chemotaxis-related genes. Representation is the same as that of (**a**). **c** Time-resolved expression of *hrpG* and flagella biosynthesis-related genes from RNA-Seq (*left*) and qRT-PCR (*right*). **d** Time-resolved expression of *hrpG* and genes of the GGDEF domain-containing proteins from RNA-Seq (*left*) and qRT-PCR (*right*). The unit of time is min. Red lines represent the expression levels of genes in the control (untreated) *Xoo* cells. Y-axis represents fold-change

Chemotaxis and motility-related genes comprise of those encoding chemoreceptors, chemotaxis proteins, twitching motility proteins, flagella motor proteins, pilus biogenesis proteins and pilus assembly proteins [14]. Motility-related genes like *fliEFGHIJKLMNOPQR* and *flgBCDEFGHIJKL* were downregulated up to 10 min, and were then upregulated between 10 and 60 min (Fig. 4a). At 5 min, the expressions of *fliM* (*Xoo2608* encoding the flagella motor switch protein), *fliN* (*Xoo2609* encoding the flagella motor switch protein), *fleN* (*Xoo2620* encoding the flagella biosynthesis switch protein) *motC* (*Xoo2830* flagella motor protein), and *motB* (*Xoo2831* encoding the flagella motor protein MotD) were downregulated by 0.7, 0.7, 0.4, 0.4 or 0.5-fold, respectively and they were upregulated at 30 min by 2.7, 2.3, 2.0, 2.9 or 3.0-fold, respectively (Fig. 4c). Their continued expression at substantially high levels was observed for up to 60 min. Chemotaxis related genes like *cheW*, *cheY* and *cheR* exhibited patterns similar to the flagella genes (Fig. 4b).

Expression of *hrpG* was previously reported to repress chemotaxis and flagella biosynthesis-related genes and consequently repress bacterial motility [37], which is consistent with our data. HrpG was expressed at 5 min and began to decrease immediately after 10 min. Prior to the 10 min time point, chemotaxis and motility related genes were downregulated, but their expression subsequently increased until 30 min followed by a slow decrease. When we compared the expression of chemotaxis and motility-related genes with that from other in vitro system using a minimum medium [30], the chemotaxis genes in the minimum medium system showed the downregulated expression pattern similar to that at 5 to 10 min of our in vitro system using RLX (Additional file 10: Table S6). The motility-related genes in the minimum medium system showed mostly upregulated expressions.

### Cyclic-di-GMP control-related genes

Cyclic-di-GMP, a universal bacterial second messenger, regulates cell cycle progression, development, cell motility, virulence, biofilm formation, RNA modulation, bacterial predation and responses to a variety of environmental stimuli including stress [15–20, 38]. The level of cyclic-di-GMP is modulated by cyclic-di-GMP synthetases and hydrolases containing GGDEF, EAL and HD-GYP domains [38]. Change in cyclic-di-GMP level affects the functional activity of the global regulator Clp [39]. Most Clp-dependent genes are associated with virulence functions and deletion of *clp* abolishes virulence of the phytopathogen [39].

In *Xoo*, 11 GGDEF domain-containing proteins exist, among which gene products of *Xoo2614*, *Xoo2615* and *Xoo2787* were downregulated before 10 min and upregulated from 15 to 60 min (Fig. 4d). *Xoo2561* and *Xoo2616* from amongst the five EAL-GGDEF domain-containing proteins and *Xoo4220* from amongst the three HD-GYP domain-containing proteins were similarly downregulated until 15 min and subsequently upregulated (Additional file 11: Figure S5). The absolute expression level of *Clp* was very high (RPKM: 1200–1400), but the fold changes in expression were very low (between 0.88 and 1.28). We speculate that Clp activity may be controlled after transcription, possibly posttranslationally via binding with its ligand, cyclic-di-GMP.

### Sugar transport-related genes

The fructose-specific phosphotransferase system (PTS) exists ubiquitously in gram-negative bacteria [40]. The fructose-specific PTS, including the carbohydrate selective porin encoded by *rpfN*, is important for the growth and pathogenicity of many phytobacteria [41]. The absence of the sugar porin reduces carbohydrate uptake and induces synthesis of cell wall-degrading enzymes to increase sugar supply [42, 43].

**Fig. 5:**
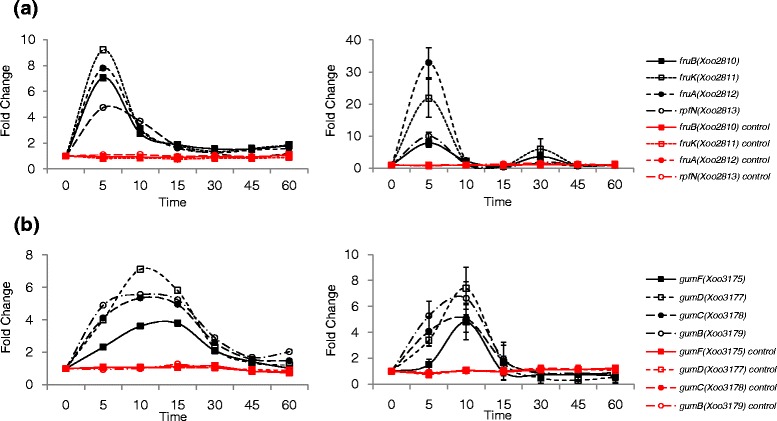
Time-resolved expression of sugar transport-related genes. **a** Time-resolved expression of fructose-specific phosphotransferase system genes and the carbohydrate selective porin gene, *rpfN,* from RNA-Seq (*left*) and qRT-PCR (*right*). **b** Time-resolved expression of *gum* operon genes from RNA-Seq (*left*) and qRT-PCR (*right*). The unit of time is min. Red lines indicate the expression levels of genes in the control (untreated) *Xoo* cells. Y-axis represents fold-change

*Xoo* has a fructose-specific PTS, consisting of *fruB* (*Xoo2810* encoding the multi-phosphoryl transfer protein), *fruK* (*Xoo2811* encoding the 1-phosphofructokinase), and *fruA* (*Xoo2812* encoding the PTS fructose-specific transporter subunit IIBC); all of which were upregulated at 5 min (Fig. 5a). In the same operon, *rpfN* (*Xoo2813*), a regulator of pathogenicity factors (carbohydrate selective porin), was also upregulated at 5 min. Carbohydrates obtained through RpfN and fructose-specific PTS could be used for extracellular polysaccharide (EPS) biosynthesis [41]. In our study, the genes *gumN*, *gumG*, *gumF*, *gumE*, *gumD*, *gumC* and *gumB* were upregulated from 5 min and the genes *gumM*, *gumL*, *gumK*, *gumJ*, *gumI* and *gumH* were upregulated from 10 to 15 min (Fig. 5b and Additional file 12: Figure S6). When we compared the expression of EPS biosynthetic *gum* genes with that from the minimum medium system [30], the similar upregulated expression pattern was observed in both systems (Additional file 13: Table S7).

### T2SS substrate genes

Substrates of the Type II secretion system (T2SS) such as proteases, lipases and cell wall-degrading enzymes, facilitate bacterial infection by degrading plant cell walls. Recent studies showed that the substrate genes of T2SS belong to the HrpG or HrpX regulon [44]. Our results showed that *htrA*, which encodes the protease DO, was upregulated from 5 to 10 min (Additional file 14: Figure S7A). *egl* (*Xoo0281* encoding the cellulase), *celS* (*Xoo1076* encoding the cellulase S), and *xynB* (*Xoo1371* encoding the xylanase) were also upregulated in a similar manner. In contrast, other genes like *egl* (*Xoo0282* encoding the cellulase), *Xoo1077* (encoding the cellulase S), *engXCA* (*Xoo4019* encoding the cellulase), and *Xoo4035* (encoding the 1, 4-beta-cellobiosidase) were downregulated in our system (Additional file 14: Figure S7B). In *Xoo*, there are three *egl* genes: *Xoo0281*, *Xoo0282* and *Xoo0283* that encode cellulases. Their expression patterns were all different: the expression of *Xoo0281* was upregulated from 5 to 60 min, the expression of *Xoo0283* was initially downregulated from 5 to 10 min and then upregulated from 30 to 60 min, and the expression of *Xoo0282* was downregulated from 15 min. The different expression patterns of each copy might arise from the neofunctionalization of functional divergence from gene duplication.

### Essentiality of transcriptionally upregulated pathogenicity-related genes

**Fig. 6:**
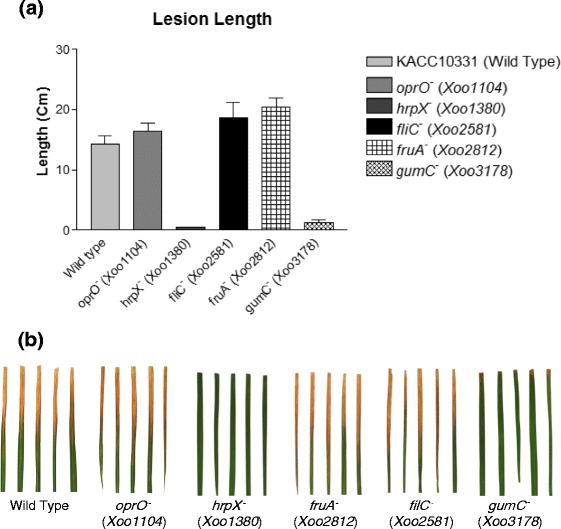
Pathogenicity test of transcriptionally upregulated genes using the lesion length test. **a** Lesion length in *Xoo*-susceptible rice leaves infected by single-gene knockout *Xoo* mutants. **b** Infected rice leaves in multiple experiments

We studied whether the transcriptionally upregulated genes are essential for causing bacterial blight (Additional file 15: Table S8). Pathogenicity tests were performed by measuring lesion length in the leaf of *Xoo*-susceptible rice Milyang23 [45] after infection with 6 *Xoo* strains including wild-type and single-gene knockout mutants of *oprO*^*−*^ (*Xoo1104*), *hrpX*^*−*^ (*Xoo1380*), *fruA*^*−*^ (*Xoo2812*), *fliC*^*−*^ (*Xoo2581*) and *gumC*^*−*^ (*Xoo3178*). *hrpX* (*Xoo1380*) was essential for *Xoo* pathogenicity (Fig. 6). In different *Xoo* strains of KACC10859, the mutants of *hrp* genes like *hrpB, hrpE, hrpD6, hrpD5,* as well as *hrpG* also showed no pathogenicity [46]. In the sugar transport signaling pathway, *gumC* (*Xoo3178*) which was upregulated at 5 min, was also strictly required for pathogenicity. In the KACC10859 strain, various *gum* genes were tested to assess their relation to pathogenicity with a different *Xoo*-susceptible rice cultivar, IR24 [47]. In addition to *gumC,* other genes like *gumB, gumD, gumE, gumH* and *gumK* were also essential for pathogenicity. The pathways involved in *hrp* and *gum* expression could be good target pathways to nullify the pathogenicity of *Xoo*. In our pathogenicity tests, not all the upregulated genes were essential to cause bacterial blight. For instance, upregulated genes such as *oprO* (*Xoo1104*), *fliC* (*Xoo2581*) and *fruA* (*Xoo2812*) were not essential for pathogenicity.

## Discussion

Although plant responses upon initial interaction with pathogens have been actively studied for the purpose of finding new disease-resistant genes or elucidating disease-development mechanisms, little is known about how the pathogens respond to initial interactions. In vivo and in vitro systems are available for studying the plant-pathogen interactions between rice and *Xoo*. The in vivo system involves local *Xoo* inoculation into rice leaf, which is useful to study the hypersensitive response of rice: for example, the resistance response of specific rice cultivars against specific *Xoo* strains. However, it is difficult to study the pathogenic responses of *Xoo* at the site of inoculation by using the in vivo system.

The in vitro system can allow control of the initial interactions between plant and pathogen in a time-dependent manner, even though interaction in the in vitro system could be different from the real in vivo interactions. The conventional in vitro system for the activation of pathogenicity of phytopathogen uses minimum media or nutrient-poor synthetic media such as XOM2, XOM3 and XVM2 [24–26]. XOM2 is a minimum media that uses xylose as the only sugar source and has no plant-derived factors, only constitutive activation of pathogenicity is obtained. The pathogenicity activation of *Xanthomonas* at a specific time requires the switching of culture media to minimum media, which is not technically possible without affecting many other environmental factors. A single-gene knockout strain of the pathogen has been compared with the wild-type strain to study the pathogenicity under the condition of consistent pathogenicity-activation. For example, microarray data from *Xanthomonas axonopodis* pv. *citri* were analyzed between a wild-type strain and mutant strains *of hrpG* or *hrpX* on XVM2 medium [26].

In the present study, we used a different in vitro system to activate the pathogenicity of *Xoo* via RLX treatment, which is closer to an in vivo system compared to the conventional in vitro system that uses minimum media. Our in vitro assay system mimics the initial interactions between rice and *Xoo* by treating the *Xoo* cell culture with RLX from a *Xoo*-susceptible rice cultivar (Milyang23) at its exponential growth stage. The RLX was freshly prepared to contain all the factors from rice leaves. Initial infections by *Xoo* usually occur through scars on rice leaves. The damaged regions of leaves allow *Xoo* cells to penetrate rice tissues easily and can provide signals for *Xoo* cells to activate their pathogenicity. The in vitro assay system using RLX treatment is designed to mimic these initial in vivo interactions. RLX is the total extract of rice leaves prepared by grinding fresh leaves in liquid nitrogen. Even though RLX treatment cannot exactly replicate the initial interaction signals, we speculate that RLX contains all the components that can be extracted from damaged leaves. Considering the pathogen, the state of *Xoo* cells at the time of RLX treatment may be different from the in vivo condition at the infection site in terms of nutrition, inorganic ions, pathogen population and environmental stress. However, in our in vitro assay system, all *Xoo* cells in culture receive the pathogenicity-activation signal from RLX simultaneously, which enables us to study the initial pathogenicity signals in a synchronized manner from a homogeneous population of *Xoo* cells providing an amplified signal-to-noise ratio. At in vivo infection site, the pathogenicity-related interactions could be heterogeneous from cell to cell and it is difficult to control and study these interactions at a single-cell level.

The in vitro assay system using RLX treatment was combined with RNA-Seq to study the time-dependent changes of genome-wide gene expression in pathogenicity-activated *Xoo* cells. The transcriptional expression response of *Xoo* at 5 min upon RLX treatment indicates how rapidly *Xoo* can respond to signals from the host rice. Our in vitro system showed the transient transcriptional up- or downregulation of pathogenicity-related genes upon the interaction with RLX. Thus far, the plant-pathogen interactions have been studied mostly in a time point and generally considered as persistently sustained throughout the interactions. In cases of animal pathogens like *Salmonella, Streptococcus pneumonaie, Streptomyces coelicolor* and *Escherichia coli*, the transient transcriptional upregulation (so-called transcription surge) which is similar to our result has been known [48–51]. The animal pathogens also have the conserved pathogenic systems of T3SS, effectors and TCS like plant pathogens. Currently, we do not understand if the virulence mechanism of *Xoo* or plant pathogen is conserved with that of the animal pathogens. However, we should be aware that the gene expression of pathogen upon plant-pathogen interactions can change dramatically depending on time, especially at the early stage.

The transient expression and different expression time of pathogenic genes also implies that there could be multiple stages in the *Xoo*-mediated infection and disease development. For instance, the initial stage may be the recognition of damage on rice leaf tissues by *Xoo* via direct contact, the middle stage could be the penetration of damaged rice leaf tissues and effector secretion by *Xoo*, and the later stages could involve the proliferation of *Xoo* cells from the infection site by evading the resistance response by rice.

Among 20 categories of COG, iron-uptake and inorganic phosphate-uptake genes in category P were the most noticeable early upregulated genes, which implies that one of the putative factors from rice to activate the pathogenicity of *Xoo* could be inorganic ions. The inorganic ions are actively depleted by rice and unavailable for *Xoo*, but the damaged rice tissues will release the essential inorganic nutrients for *Xoo*. We treated RLX on the exponentially growing state of Xoo, when most nutrients could start running out. In that case, when *Xoo* cells are in need of nutrients, *Xoo* cells seem to be supplied the necessary factors from the damaged rice tissues including inorganic ions. We should further investigate the needs of *Xoo* for nutrimental components and the environmental condition at the RLX treatment.

**Fig. 7:**
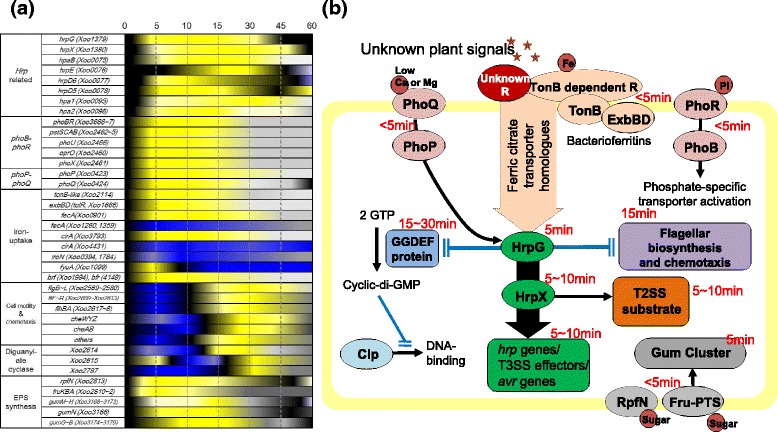
**a** Continuous time-resolved heat map of representative putative pathogenicity-related genes. Upregulated genes are shown in yellow and downregulated genes are shown in blue. Continuous gene expression levels are proposed by non-linear regression data fitting using RNA-Seq data from seven different time points. Details are shown in Additional file 16: Figure S8. **b** Schematic model of the pathogenicity signaling pathways in *Xoo.* Plant pathogenic signals activate unknown receptor proteins, which relay the pathogenic signal to the global regulators such as HrpG. Activated HrpG delivers the signal to HrpX, which activates a large set of virulence genes such as T3SS and T3SS effectors (*green*). HrpX activates T2SS substrate gene expression (*orange*). Activated HrpG suppresses flagella biosynthesis- and chemotaxis-related genes (*purple*) and GGDEF domain-containing proteins (*light blue*) by which the synthesized cyclic-di-GMP binds to Clp (*cyan*) and abolishes the DNA binding of Clp. Two-component systems (*salmon*) are upregulated by the pathogenicity signal. Sugar-uptake genes (*grey*) such as *rpfN* and fructose-specific phosphotransferase system genes are also upregulated. In the early pathogenicity signal, iron-uptake genes (*light orange*) such as the TonB-dependent receptor, TonB, ExbB, ExbD and ferric citrate transporter genes are upregulated. Black arrows indicate activation signals and blocked blue lines indicate repression signals. The time of gene expression at transcription level is labeled for each pathway in red letters

Many pathogenicity-related genes were expressed as early as 5 min in our system and it is difficult to further divide the 5 min time interval by every minute in order to study the gene expression pattern at finer time points. Herein, we introduced the non-linear regression method by using multiple time-point RNA-Seq data. An ordinary heat map of RNA-Seq represented the extent of up- or downregulated expression of a specific gene compared to a single reference point. Our time-dependent expression data of a gene are not a single point but rather multiple points that could be connected with the neighboring time points of every 5 min, which helped us to extend discontinued gene expression data to continuous data (Figs. 2, 3, 4, 5 and Additional file 6: Figure S2, Additional file 8: Figure S3, Additional file 9: Figure S4, Additional file 11: Figure S5, Additional file 12: Figure S6, Additional file 14: Figure S7, Additional file 16: Figure S8) and generate a time-resolved continuous gene expression heat map for the better interpretation of the initial rice-*Xoo* interaction (Fig. 7a). Although the representations using the non-linear regression method could be just a visual aid, it is better than the simple comparison of expression levels or slopes of increasing or decreasing gene expression levels between two consecutive time points. For example, the maximal expressions of HrpG and HrpX were observed at 10 and 15 min, respectively. In the time-resolved continuous presentation, the expected HrpG expression is higher than that of HrpX between 0 and 5 min (Fig. 3a). In the time-resolved continuous heat map, the hierarchy of transcriptional signals could be speculated better. The flagella-related genes were downregulated mostly at 5 min, and chemotaxis related genes were downregulated mostly at 10 min, which was not evident in the normal discontinuous heat map.

## Conclusions

In this study, we combined the in vitro assay system with RNA-Seq to study the time-resolved genome-wide gene expression of *Xoo* cells upon initial interactions with RLX. Pathogenicity of *Xoo* was activated immediately within minutes of the interaction with RLX (Fig. 7b), involving differentially expressed genes belonging either to the same or different pathways. The time information was previously unavailable with the conventional in vivo and in vitro assay system. Our in vitro system also enables the use of the wild-type strain of *Xoo* as the genetic background. We can initiate the pathogenicity of *Xoo* at any time with the present in vitro assay system. The timely order of pathogenic gene expression is useful to understand the plant-pathogen interactions at the infection site. Our gene expression data with the 5 min resolution provide an additional dimension of time to study the conventional RNA-Seq data of the expression of each gene in the genome. Our in vitro system combined with RNA-Seq will be useful to study the gene expression order or hierarchy between pathogenicity-related genes and the detail time-resolved gene expression such as transient up- or downregulation of pathogenic gene expression upon plant-pathogen interactions.

## Methods

### Bacterial strain and culture conditions

*Xanthomonas oryzae* pv. *oryzae* (*Xoo*) KACC10331 was obtained from the Korean Agricultural Collection Center (KACC), which has 4,941,439 nucleotides and 4065 protein genes and no autonomous plasmids were apparent [5]. *Xoo* KACC10859 is not yet completely sequenced. The bacteria were grown in nutrient broth (Difco, Detroit, MI, USA) or YGC (2.0 % d-(+)-glucose, 2.0 % CaCO_3_, 1.0 % yeast extract, 1.5 % agar) in a shaking incubator at 28 °C with an agitation speed of 200 rpm. The single-gene knockout mutants of *Xoo* were generated by the insertion of transposons containing a kanamycin resistant gene as the selection marker. To select the knockout mutants when necessary the culture medium was supplemented with 10 μL · mL^−1^ kanamycin.

### Treatment of rice leaf extract and total RNA extraction from *Xoo* cells

Rice was grown on a paddy field at Suwon in South Korea (37°15′43.8″N 126°59′15.8″E) before panicle initiation (approximately 8–9 weeks). The rice leaves (*Oryza sativa* L. *cv*. Milyang 23; a *Xoo*-susceptible rice cultivar) (40 clumps) were harvested and homogenized in liquid nitrogen with a mortar and pestle. The resulting homogenate (RLX) was aliquoted to 1 g in Eppendorf-tubes and stored at −80 °C. A 40 mL culture of *Xoo* was grown in nutrient broth to the mid-exponential phase (A_600_ of 0.5) in a shaking incubator at 28 °C with an agitation speed of 200 rpm and an RLX aliquot (1 g) was added to the culture medium. Samples (1.5 mL) of the culture were harvested from the shaking 40 mL culture at specified time intervals, centrifuged at 10,000 × *g* for 5 min at room temperature, and immediately mixed with RNA protect Bacterial Reagent (Qiagen, Valencia, CA, USA). Total RNA was extracted from each sample of the harvested *Xoo* cells separately using an RNeasy Plus Mini Kit (Qiagen) according to the manufacturer’s instructions. The concentration and quality of RNA in the samples was assessed using the NanoDrop^TM^ 1000 spectrophotometer (NanoDrop Technologies, Wilmington, DE, USA). The quantity of total RNA was determined by measuring the absorbance at 260 nm and 280 nm. In addition, the A_260_/A_280_ ratio was used to assess the level of protein contamination in RNA.

### RNA sequencing and data analysis

Total RNA from approximately 10 μg of each sample was used as the starting material for generating sequencing libraries. Ribosomal RNA was depleted by using MICROBExpress Bacterial mRNA Enrichment Kit (Ambion, Austin, TX, USA). Enriched mRNA was prepared with the Illumina TruSeq RNA Sample Preparation Kit (Illumina, San Diego, CA, USA) following the manufacturer’s instructions. As per the protocol, RNA was fragmented using the included buffer for 5 min and cleaned up. The resulting RNA was subsequently used for reverse transcription and second-strand cDNA synthesis with random hexamer primers. Purified cDNA fragments were end-repaired, A-tailed, ligated to TruSeq sequencing adapters, and amplified by 15 cycles of PCR. One lane per sample was used for sequencing with an Illumina Genome Analyzer IIx to generate non-directional, pair-ended 36-bp reads. Quality-filtered reads were mapped to the reference genome sequence (http://www.ncbi.nlm.nih.gov/nuccore/58579623?report=fasta) using CLC Genomics Workbench 4.0 (CLC bio, Aarhus, Denmark). Relative transcript abundance was calculated by counting the number of reads per kilobase per million mapped sequence reads (RPKM) [52].

### Quantitative real-time PCR (qRT-PCR)

RNA was reverse transcribed using a PrimeScript^TM^ 1st strand cDNA Synthesis Kit (Takara, Tokyo, Japan) with random hexamer primers. Gene-specific primers were designed to amplify sequences 100–150 bp in length from the *Xoo* genome using Beacon Designer^TM^ 8.0 (Premier Biosoft, Palo Alto, CA, USA). Real-time PCR was performed with a CFX96^TM^ Real-Time System (Bio-RAD, Hercules, CA) using the iQ^TM^ SYBR Green Supermix (Bio-RAD) following the manufacturer’s instructions. The 16S rRNA gene was used as an endogenous control. The expression level of each gene was calculated using three biological replicates. Fold changes of relative and normalized expression values were calculated with data from CFX Manager 3.0 (Bio-Rad).

### Time-resolved continuous heat map analysis

Log_2_-scaled RPKM values from RNA-Seq data at each time point within the first hour post-RLX treatment were fitted to a curve and non-linear regression was performed using GraphPad Prism (version 3.02 for Windows, GraphPad Software, San Diego California USA, www.graphpad.com) to obtain the resulting continuous RPKM values vs. time from the fitted curve. The maximum expressed level of each gene within 1 h was set as 1.0 and the continuous expression level vs. time was scaled based on the maximum reference level. When the expressed level was downregulated compared with that at 0 h, the lowest expressed level was set to −1.0 and the continuous expression level was scaled in the same manner. The increasing and decreasing expression level of each gene was compared with other genes in time-resolved continuous heat map. Heat maps were generated with MeV (Multi Experiment Viewer) [53] by adding the fold changes of RPKM values into a single-color array.

### Insertional transposon mutagenesis of *Xanthomonas oryzae* pv. *oryzae*

To perform transposon mutagenesis, 1 μL of Transposome^TM^ (20 ng · μL^−1^; Epicentre Technologies, Madison, WI, USA) was mixed with 50 μL of *Xoo* electrocompetent cells and placed in a 0.2 cm gap electroporation cuvette (Bio-Rad). A single high-voltage pulse (12.5 kV/cm for 5 ms, with a resistance value of 200 Ω and capacitance of 25 μF) was applied with the Gene Pulser II system (Bio-Rad). After pulse delivery, the cells were immediately removed from the electroporation cuvette and inoculated into 1 mL of SOC medium (2.0 % tryptone, 0.5 % yeast extract, 0.05 % NaCl, 20 mM glucose) without antibiotics. The cells were incubated for 18 h at 28 °C with constant shaking (120 rpm). After incubation, the putative transformants were plated onto nutrient agar plates containing 10 μL · mL^−1^ of kanamycin and incubated at 28 °C for 3 days. To select the transposon-inserted mutants from colonies, genomic DNA was isolated using the genomic DNA extraction kit (DNeasy Mini Kit, Qiagen). The genomic flanking sequences of the transposon were amplified by thermal asymmetric interlaced (TAIL)-PCR with KOD-Plus DNA polymerase (Toyobo, Japan) using the primer KAN2F (5′-CTCGATGAGTTTTTCTAATCAGAAT-3′). The first 30 cycles of the PCR were carried out at 55 °C, with a 30 s extension. The next 30 cycles were carried out at 30 °C, with a similar extension time, and the last 30 cycles were performed at 55 °C with a 2 min extension. One microliter of the TAIL-PCR product and primer KAN-2 FP-1 (5′-ACCTACAACAAAGCTCTCATCAACC-3′) were used in ‘2×’ Big Dye Terminator sequencing reactions, according to the manufacturer’s protocols (PE Applied Biosystems). To confirm whether the mutants obtained were derived from true transposition or not, southern hybridization was carried out with genomic DNA from the mutants, digested with *EcoRI* and separated by electrophoresis in 0.8 % agarose gel. The transposon was labeled as the probe with [α-32P] dCTP using the random priming method according to the manufacturer’s instructions (LaddermanTM Labeling kit, Takara, Japan).

### Pathogenicity assay

Leaves of 4-to-6-week-old *Xoo*-susceptible rice plant (*Oryza sativa* L. *cv.* Milyang 23)—five of the uppermost leaves of each plant—were used for pathogen inoculation by the leaf cutting method [45], which consists of clipping the leaf tip with sterile scissors dipped in a suspension of a mutated *Xoo* strain in sterile water at a concentration of A_600_ = 0.5. The plants were inoculated in a greenhouse maintained between 25 and 30 °C, and a relative humidity of 60 %. The control plants were inoculated similarly with sterile water under the same conditions. Before inoculation, the leaves were thoroughly washed under running tap water to remove dirt, surface disinfected in 70 % ethanol, and rinsed thrice with sterile distilled water. The susceptible rice was inoculated with the *Xoo* strain (five replicates each), and lesion lengths were measured after two weeks.

### Accession number

The RNA-Seq data were deposited in NCBI GEO (Gene Expression Omnibus) with the accession number GSE61607.

### Availability of data and materials

The RNA-Seq data were deposited in NCBI GEO (Gene Expression Omnibus) with the accession number GSE61607.

## Additional files

Additional file 1: Table S1. RPKM values of RNA-Seqs of the pathogenicity-activated and control *Xoo* cells. (XLSX 1168 kb) Additional file 2: Table S2. Randomly selected 28 genes for qRT-PCR and their p-values with RNA-Seq data. (DOCX 26 kb) Additional file 3: Table S3. Sequence reads for pathogenicity-activated and control *Xoo* cells. (DOCX 16 kb) Additional file 4: Figure S1. The number of upregulated (UR) and downregulated (DR) genes for each time (min) point 0, 5, 10, 15, 30, 45 and 60 min in P-activated *Xoo* cells (upper). The number of upregulated (UR) and downregulated (DR) genes for each time (min) point 0, 5, 10, 15, 30, 45 and 60 min in control *Xoo* cells (lower). (PPTX 51 kb) Additional file 5: Table S4. Differentially expressed genes in pathogenicity-activated *Xoo* cells (compared with the expression level at zero time with an RPKM threshold of 2.0 and a difference filter of 2.0). (DOCX 270 kb) Additional file 6: Figure S2. Time-resolved expression of *hrp* genes including *hpaB*, *hrpE*, *hrpD6*, *hrpD5*, *hpa1* and *hpa2*. The unit of time is min. Red lines indicate the expression levels of genes in control (untreated) *Xoo* cells. Y-axis represents fold-change. (PPTX 51 kb) Additional file 7: Table S5. Fold changes of transcriptional expression levels of *hrp* genes in *Xoo* and *Xcc* from two different in vitro assay systems. (DOCX 19 kb) Additional file 8: Figure S3. Time-resolved expression of genes related to iron-uptake. (A) Among *tonB* genes, *Xoo2114* was upregulated 6-fold. (B) Upregulated TonB-dependent receptor genes; *fecA* (*Xoo0901*) and *cirA* (*Xoo3793*) were upregulated 80- and 20-fold, respectively. (C) Downregulated TonB-dependent receptor genes; *iroN* (*Xoo0394*), *fyuA* (*Xoo1099*), *iroN* (*Xoo1784*) and *cirA* (*Xoo4431*) were downregulated 50-, 5-, 5- and 4-fold, respectively. (D) *tolR* (*Xoo1666, exbD*-like) was upregulated 2.5-fold. The unit of time is min. Red lines indicate the expression levels of genes in control (untreated) *Xoo* cells. Y-axis represents fold-change. (PPTX 84 kb) Additional file 9: Figure S4. Time-resolved expression of two component system (TCS) genes. (A) *phoB*-*phoR* genes were upregulated within 5 min. (B) *ppk* was upregulated within 5 min whereas *ppx* expression was not changed. (C) *phoP*-*phoQ* genes were also upregulated within 5 min. The unit of time is min. Red lines indicate the expression levels of genes in control (untreated) *Xoo* cells. Y-axis represents fold-change. (PPTX 71 kb) Additional file 10: Table S6. Fold changes of transcriptional expression levels of chemotaxis and motility-related genes in *Xoo* and *Xcc* from two different in vitro assay systems. (DOCX 25 kb) Additional file 11: Figure S5. Time-resolved expression of EAL-GGDEF and HD-GYP containing genes. The unit of time is min. Red lines indicate expression levels of genes in control (untreated) *Xoo* cells. Y-axis represents fold-change. (PPTX 70 kb) Additional file 12: Figure S6. Time-resolved expression of *gumH–gumM* genes. The unit of time is min. Red lines indicate expression levels of genes in control (untreated) *Xoo* cells. Y-axis represents fold-change. (PPTX 51 kb) Additional file 13: Table S7. Fold changes of transcriptional expression levels of *gum* genes in *Xoo* and *Xcc* from two different in vitro assay systems. (DOCX 17 kb) Additional file 14: Figure S7. Time-resolved expression of T2SS substrate genes. (A) Genes like *htrA*, *egl* (*Xoo0281*), *celS* and *xynB* were upregulated. (B) Genes like *egl* (*Xoo0282*), *Xoo1077*, *engXCA* and *Xoo4035* were downregulated. *egl* (*Xoo0283*) was downregulated within 10 min and upregulated subsequently. The unit of time is min. Red lines indicate the expression levels of genes in control (untreated) *Xoo* cells. Y-axis represents fold-change. (PPTX 59 kb) Additional file 15: Table S8. Virulence of wild-type and mutant *Xoo* strains on rice. (DOCX 16 kb) Additional file 16: Figure S8. Representation of continuous time-resolved gene expression of selected genes. (A) Scaled continuous time-resolved gene expression. The absolute expression levels of each gene were log_2_-scaled and the highest level was set to 1.0. The expression levels at each time point were fitted by non-linear regression method to derive time-resolved continuous gene expression levels. (B) Unscaled continuous time-resolved gene expression. The absolute expression levels of each gene were log_2_-scaled as in (A), but the highest level was not set to 1.0. The expression levels at each time point were also fitted by non-linear regression method to derive the time-resolved continuous gene expression levels. (PPTX 3239 kb)

## Abbreviations

P-activated: pathogenicity activated
RLX: rice leaf extract
RPKM: reads per kilobase per million mapped reads
