# Supplementary material for: Time-resolved pathogenic gene expression analysis of the plant pathogen Xanthomonas oryzae pv. oryzae
Source: BMC Genomics. 2016 May 10;17:345. doi: 10.1186/s12864-016-2657-7 (PMC4862043; doi:10.1186/s12864-016-2657-7)
Supplement: Additional file 2: Table S2. — Randomly selected 28 genes for qRT-PCR and their p-values with RNA-Seq data. (DOCX 26 kb) [file 12864_2016_2657_MOESM2_ESM.docx]

**Table S2.** Randomly selected 28 genes for qRT-PCR and their p-values with RNA-Seq data.

| Gene | RNA-Seq | qRT-PCR | | |
| --- | --- | --- | --- | --- |
|  | log_2_FC | | log_2_FC | p-value |
| **5 min** |  |  | |  |
| *Pcp (Xoo1511)* | 2.07 | 3.19 | | 2.95E-03 |
| *Brf (Xoo1994)* | 4.56 | 4.81 | | 2.00E-06 |
| *Xoo2375* | 0.28 | 0.43 | | 6.53E-03 |
| *oprO (Xoo2460)* | 3.79 | 2.53 | | 1.56E-04 |
| *pstC (Xoo2463)* | 3.57 | 3.63 | | 9.42E-04 |
| *pstA (Xoo2464)* | 3.44 | 2.04 | | 1.20E-05 |
| *pstB (Xoo2465)* | 3.32 | 2.18 | | 8.20E-05 |
| *phoU (Xoo2466)* | 3.02 | 1.80 | | 1.00E-06 |
| *acvB (Xoo2738)* | 1.93 | 1.44 | | 1.74E-04 |
| *fruB (Xoo2810)* | 2.82 | 2.98 | | 1.28E-03 |
| *fruK (Xoo2811)* | 3.20 | 4.44 | | 7.40E-05 |
| *fruA (Xoo2812)* | 2.96 | 5.04 | | 9.98E-04 |
| *rpfN (Xoo2813)* | 2.25 | 3.31 | | 1.32E-04 |
| *gumF (Xoo3175)* | 1.21 | 0.59 | | 1.19E-03 |
| *gumD (Xoo3177)* | 2.00 | 1.76 | | 8.80E-04 |
| *gumC (Xoo3178)* | 2.04 | 2.03 | | 3.33E-04 |
| *gumB (Xoo3179)* | 2.29 | 2.40 | | 4.91E-04 |
| *ahpC (Xoo3645)* | -0.13 | -0.22 | | 1.37E-03 |
| *ahpF (Xoo3646)* | 0.13 | 0.71 | | 3.47E-03 |
| *phoB (Xoo3666)* | 4.10 | 2.66 | | 8.50E-05 |
| *bfr (Xoo4149)* | 4.65 | 5.16 | | 1.80E-05 |
| *phoD (Xoo4555)* | 4.60 | 5.70 | | 7.60E-04 |
| *atsE (Xoo0041)* | -0.17 | -0.43 | | 4.03E-03 |
| *iron (Xoo0394)* | -1.47 | -1.40 | | 2.18E-04 |
| *fabA (Xoo0762)* | -1.83 | -1.26 | | 1.09E-03 |
| *fabD (Xoo0880)* | -1.05 | -1.40 | | 1.90E-03 |
| *Xoo4150* | -4.29 | -5.41 | | 2.00E-06 |
| *ucpA (Xoo4537)* | -0.50 | 0.87 | | 3.55E-03 |
| **10 min** |  |  | |  |
| *Pcp (Xoo1511)* | 2.58 | 3.35 | | 1.08E-03 |
| *Brf (Xoo1994)* | 4.30 | 4.50 | | 2.00E-06 |
| *Xoo2375* | 0.76 | 0.74 | | 4.34E-03 |
| *oprO (Xoo2460)* | 3.96 | 2.65 | | 5.62E-03 |
| *pstC (Xoo2463)* | 3.39 | 4.31 | | 4.67E-04 |
| *pstA (Xoo2464)* | 3.21 | 2.14 | | 9.40E-05 |
| *pstB (Xoo2465)* | 3.23 | 2.56 | | 4.75E-04 |
| *phoU (Xoo2466)* | 3.10 | 2.23 | | 7.90E-05 |
| *acvB (Xoo2738)* | 1.86 | 1.34 | | 1.81E-03 |
| *fruB (Xoo2810)* | 1.46 | 1.14 | | 6.43E-04 |
| *fruK (Xoo2811)* | 1.64 | 0.83 | | 1.34E-03 |
| *fruA (Xoo2812)* | 1.66 | 1.25 | | 3.29E-03 |
| *rpfN (Xoo2813)* | 1.89 | 0.50 | | 2.89E-03 |
| *gumF (Xoo3175)* | 1.85 | 2.27 | | 2.87E-04 |
| *gumD (Xoo3177)* | 2.83 | 2.89 | | 6.00E-06 |
| *gumC (Xoo3178)* | 2.42 | 2.34 | | 1.90E-05 |
| *gumB (Xoo3179)* | 2.47 | 2.73 | | 1.80E-05 |
| *ahpC (Xoo3645)* | 0.17 | 0.69 | | 1.27E-03 |
| *ahpF (Xoo3646)* | 0.65 | 1.38 | | 1.16E-04 |
| *phoB (Xoo3666)* | 4.01 | 3.03 | | 3.00E-06 |
| *bfr (Xoo4149)* | 4.46 | 4.73 | | 4.00E-06 |
| *phoD (Xoo4555)* | 5.00 | 5.94 | | 0.00E+00 |
| *atsE (Xoo0041)* | -0.26 | -1.77 | | 6.20E-05 |
| *iron (Xoo0394)* | -4.10 | -6.64 | | 3.00E-06 |
| *fabA (Xoo0762)* | -1.58 | -1.24 | | 1.20E-03 |
| *fabD (Xoo0880)* | -1.23 | 0.62 | | 7.58E-03 |
| *Xoo4150* | -4.36 | -5.41 | | 2.00E-06 |
| *ucpA (Xoo4537)* | -1.61 | -3.74 | | 1.30E-03 |
| **15 min** |  |  | |  |
| *Pcp (Xoo1511)* | 2.14 | 1.30 | | 3.28E-04 |
| *Brf (Xoo1994)* | 4.23 | 3.38 | | 2.20E-05 |
| *Xoo2375* | 1.08 | 0.02 | | 2.41E-03 |
| *oprO (Xoo2460)* | 3.38 | 1.72 | | 1.10E-04 |
| *pstC (Xoo2463)* | 2.83 | 3.04 | | 1.10E-05 |
| *pstA (Xoo2464)* | 2.64 | 1.19 | | 8.85E-03 |
| *pstB (Xoo2465)* | 2.99 | 1.96 | | 1.90E-05 |
| *phoU (Xoo2466)* | 2.64 | 0.98 | | 2.84E-03 |
| *acvB (Xoo2738)* | 1.03 | -0.58 | | 4.45E-03 |
| *fruB (Xoo2810)* | 0.91 | -0.71 | | 2.91E-03 |
| *fruK (Xoo2811)* | 0.88 | -0.64 | | 4.02E-03 |
| *fruA (Xoo2812)* | 0.69 | -0.45 | | 3.38E-03 |
| *rpfN (Xoo2813)* | 0.81 | -1.74 | | 8.30E-04 |
| *gumF (Xoo3175)* | 1.92 | -0.09 | | 7.27E-04 |
| *gumD (Xoo3177)* | 2.54 | 0.74 | | 1.91E-03 |
| *gumC (Xoo3178)* | 2.31 | 0.84 | | 8.90E-05 |
| *gumB (Xoo3179)* | 2.39 | 0.96 | | 6.10E-05 |
| *ahpC (Xoo3645)* | 0.72 | 1.14 | | 1.29E-03 |
| *ahpF (Xoo3646)* | 1.34 | 1.59 | | 7.11E-04 |
| *phoB (Xoo3666)* | 3.39 | 3.01 | | 4.43E-04 |
| *bfr (Xoo4149)* | 4.54 | 3.66 | | 1.70E-05 |
| *phoD (Xoo4555)* | 4.21 | 3.54 | | 1.11E-04 |
| *atsE (Xoo0041)* | -0.19 | -3.78 | | 1.70E-05 |
| *iron (Xoo0394)* | -5.25 | -8.97 | | 3.00E-06 |
| *fabA (Xoo0762)* | -1.58 | -2.59 | | 3.09E-03 |
| *fabD (Xoo0880)* | -1.32 | -1.63 | | 1.40E-03 |
| *Xoo4150* | -4.49 | -5.41 | | 2.00E-06 |
| *ucpA (Xoo4537)* | -2.50 | -6.59 | | 1.04E-03 |
| **30 min** |  |  | |  |
| *Pcp (Xoo1511)* | 0.82 | 0.69 | | 4.93E-04 |
| *Brf (Xoo1994)* | 2.44 | 1.53 | | 3.00E-06 |
| *Xoo2375* | 2.68 | 4.65 | | 3.68E-04 |
| *oprO (Xoo2460)* | 1.01 | 1.98 | | 4.00E-06 |
| *pstC (Xoo2463)* | 0.81 | 2.98 | | 1.16E-03 |
| *pstA (Xoo2464)* | 0.70 | 1.03 | | 1.28E-03 |
| *pstB (Xoo2465)* | 1.01 | 1.87 | | 1.33E-03 |
| *phoU (Xoo2466)* | 0.57 | 1.34 | | 7.65E-03 |
| *acvB (Xoo2738)* | 0.22 | -1.40 | | 2.36E-03 |
| *fruB (Xoo2810)* | 0.63 | 1.87 | | 3.44E-03 |
| *fruK (Xoo2811)* | 0.42 | 2.56 | | 5.00E-06 |
| *fruA (Xoo2812)* | 0.34 | 0.55 | | 3.24E-03 |
| *rpfN (Xoo2813)* | 0.26 | 0.91 | | 1.42E-03 |
| *gumF (Xoo3175)* | 1.07 | -0.45 | | 1.22E-03 |
| *gumD (Xoo3177)* | 1.18 | -1.06 | | 3.39E-03 |
| *gumC (Xoo3178)* | 1.38 | -0.28 | | 4.37E-03 |
| *gumB (Xoo3179)* | 1.53 | -0.15 | | 4.89E-04 |
| *ahpC (Xoo3645)* | 1.78 | 3.22 | | 0.00E+00 |
| *ahpF (Xoo3646)* | 2.02 | 3.34 | | 1.58E-04 |
| *phoB (Xoo3666)* | 1.26 | 2.63 | | 2.47E-04 |
| *bfr (Xoo4149)* | 2.70 | 2.36 | | 1.68E-03 |
| *phoD (Xoo4555)* | 1.41 | 4.14 | | 3.00E-06 |
| *atsE (Xoo0041)* | -1.03 | -5.00 | | 1.50E-05 |
| *iron (Xoo0394)* | -5.41 | -6.64 | | 3.00E-06 |
| *fabA (Xoo0762)* | -1.76 | -2.23 | | 3.83E-03 |
| *fabD (Xoo0880)* | -1.42 | 2.14 | | 1.95E-04 |
| *Xoo4150* | -2.73 | -1.71 | | 6.00E-06 |
| *ucpA (Xoo4537)* | -2.68 | -1.99 | | 2.52E-03 |
| **45 min** |  |  | |  |
| *Pcp (Xoo1511)* | 0.26 | -0.89 | | 2.00E-05 |
| *Brf (Xoo1994)* | 0.84 | 0.15 | | 2.11E-03 |
| *Xoo2375* | 3.02 | 2.88 | | 8.13E-04 |
| *oprO (Xoo2460)* | 0.26 | 0.03 | | 8.04E-03 |
| *pstC (Xoo2463)* | 0.35 | 1.51 | | 3.51E-03 |
| *pstA (Xoo2464)* | 0.38 | -0.25 | | 1.23E-03 |
| *pstB (Xoo2465)* | 0.17 | 0.24 | | 7.37E-03 |
| *phoU (Xoo2466)* | -0.02 | -0.71 | | 8.06E-04 |
| *acvB (Xoo2738)* | 0.40 | -0.79 | | 1.08E-03 |
| *fruB (Xoo2810)* | 0.61 | -0.34 | | 3.55E-04 |
| *fruK (Xoo2811)* | 0.67 | -0.18 | | 4.33E-03 |
| *fruA (Xoo2812)* | 0.54 | -0.62 | | 2.72E-03 |
| *rpfN (Xoo2813)* | -0.13 | 0.32 | | 3.22E-03 |
| *gumF (Xoo3175)* | 0.51 | -0.42 | | 1.27E-03 |
| *gumD (Xoo3177)* | 0.43 | -1.74 | | 2.21E-03 |
| *gumC (Xoo3178)* | 0.65 | -0.42 | | 6.32E-04 |
| *gumB (Xoo3179)* | 0.75 | -0.22 | | 8.48E-03 |
| *ahpC (Xoo3645)* | 1.79 | 3.08 | | 7.85E-04 |
| *ahpF (Xoo3646)* | 1.94 | 2.68 | | 3.69E-04 |
| *phoB (Xoo3666)* | 1.03 | 1.41 | | 5.10E-04 |
| *bfr (Xoo4149)* | 0.78 | 0.08 | | 3.64E-03 |
| *phoD (Xoo4555)* | 0.80 | 1.68 | | 3.22E-04 |
| *atsE (Xoo0041)* | -2.73 | -5.75 | | 1.40E-05 |
| *iron (Xoo0394)* | -5.56 | -6.64 | | 3.00E-06 |
| *fabA (Xoo0762)* | -1.82 | -1.84 | | 5.29E-03 |
| *fabD (Xoo0880)* | -1.04 | -2.02 | | 3.99E-03 |
| *Xoo4150* | -1.22 | -1.05 | | 1.97E-03 |
| *ucpA (Xoo4537)* | -2.65 | -5.19 | | 1.10E-03 |
| **60 min** |  |  | |  |
| *Pcp (Xoo1511)* | 0.33 | -2.65 | | 2.20E-05 |
| *Brf (Xoo1994)* | 0.37 | -0.41 | | 8.61E-03 |
| *Xoo2375* | 3.27 | 2.02 | | 9.22E-04 |
| *oprO (Xoo2460)* | 0.73 | 0.34 | | 1.18E-03 |
| *pstC (Xoo2463)* | 0.84 | 1.14 | | 4.33E-03 |
| *pstA (Xoo2464)* | 0.61 | -0.49 | | 1.09E-03 |
| *pstB (Xoo2465)* | 0.52 | 0.88 | | 3.40E-05 |
| *phoU (Xoo2466)* | 0.30 | -1.36 | | 3.40E-05 |
| *acvB (Xoo2738)* | 0.31 | -0.45 | | 3.23E-03 |
| *fruB (Xoo2810)* | 0.86 | 0.06 | | 7.19E-04 |
| *fruK (Xoo2811)* | 0.90 | 0.01 | | 9.63E-04 |
| *fruA (Xoo2812)* | 0.67 | -0.34 | | 4.26E-03 |
| *rpfN (Xoo2813)* | 0.26 | -0.25 | | 1.04E-03 |
| *gumF (Xoo3175)* | 0.03 | -0.60 | | 1.72E-03 |
| *gumD (Xoo3177)* | 0.27 | -0.85 | | 1.18E-03 |
| *gumC (Xoo3178)* | 0.56 | -0.14 | | 0.001451 |
| *gumB (Xoo3179)* | 1.02 | -0.38 | | 6.10E-04 |
| *ahpC (Xoo3645)* | 1.81 | 3.62 | | 6.40E-05 |
| *ahpF (Xoo3646)* | 2.04 | 2.45 | | 2.03E-04 |
| *phoB (Xoo3666)* | 1.06 | 1.03 | | 5.57E-04 |
| *bfr (Xoo4149)* | 0.47 | -0.16 | | 6.97E-04 |
| *phoD (Xoo4555)* | 1.24 | 0.91 | | 1.00E-04 |
| *atsE (Xoo0041)* | -3.23 | -5.84 | | 1.40E-05 |
| *iron (Xoo0394)* | -5.36 | -7.97 | | 3.00E-06 |
| *fabA (Xoo0762)* | -2.02 | -1.79 | | 5.59E-03 |
| *fabD (Xoo0880)* | -1.39 | -2.90 | | 6.50E-03 |
| *Xoo4150* | -0.68 | -0.43 | | 1.32E-03 |
| *ucpA (Xoo4537)* | -2.45 | -4.80 | | 1.14E-03 |
