# Supplementary material for: Time-resolved pathogenic gene expression analysis of the plant pathogen Xanthomonas oryzae pv. oryzae
Source: BMC Genomics. 2016 May 10;17:345. doi: 10.1186/s12864-016-2657-7 (PMC4862043; doi:10.1186/s12864-016-2657-7)
Supplement: Additional file 3: Table S3. — Sequence reads for pathogenicity-activated and control Xoo cells. (DOCX 16 kb) [file 12864_2016_2657_MOESM3_ESM.docx]

**Table S3.** Sequence reads for pathogenicity-activated and control *Xoo* cells

| Sample tag | Total read | rRNA read | mRNA read | Intergenic read | Unmapped read |
| --- | --- | --- | --- | --- | --- |
| M23 0 | 32,139,762 | 27,919 | 23,733,825 | 8,220,543 | 157,475 |
| M23 5 | 31,385,588 | 43,059 | 22,284,167 | 8,574,498 | 483,864 |
| M23 10 | 33,038,730 | 54,176 | 23,862,902 | 8,551,095 | 570,557 |
| M23 15 | 32,280,008 | 101,060 | 20,570,655 | 11,213,078 | 395,215 |
| M23 30 | 25,612,494 | 130,834 | 15,779,935 | 9,367,162 | 334,563 |
| M23 45 | 38,468,356 | 585,898 | 22,012,374 | 12,240,014 | 3,630,070 |
| M23 60 | 35,432,984 | 38,565 | 25,753,490 | 9,172,106 | 468,823 |
| w/o 0 | 31,167,172 | 74,207 | 23,263,970 | 7,668,438 | 160,557 |
| w/o 5 | 31,412,538 | 65,162 | 23,155,326 | 8,052,594 | 139,456 |
| w/o 10 | 33,387,468 | 26,321 | 24,950,069 | 8,221,782 | 189,296 |
| w/o 15 | 29,845,772 | 91,498 | 19,605,978 | 10,064,128 | 84,168 |
| w/o 30 | 32,153,462 | 117,870 | 21,357,917 | 10,577,366 | 100,309 |
| w/o 45 | 58,300,752 | 42,662 | 36,489,790 | 17,207,264 | 4,561,036 |
| w/o 60 | 36,968,108 | 106,358 | 27,028,052 | 9,649,156 | 184,542 |
