# Supplementary material for: Time-resolved pathogenic gene expression analysis of the plant pathogen Xanthomonas oryzae pv. oryzae
Source: BMC Genomics. 2016 May 10;17:345. doi: 10.1186/s12864-016-2657-7 (PMC4862043; doi:10.1186/s12864-016-2657-7)
Supplement: Additional file 4: Figure S1. — The number of upregulated (UR) and downregulated (DR) genes for each time (min) point 0, 5, 10, 15, 30, 45 and 60 min in P-activated Xoo cells (upper). The number of upregulated (UR) and downregulated (DR) genes for each time (min) point 0, 5, 10, 15, 30, 45 and 60 min in control Xoo cells (lower). (PPTX 51 kb) [file 12864_2016_2657_MOESM4_ESM.pptx]

## Slide 1
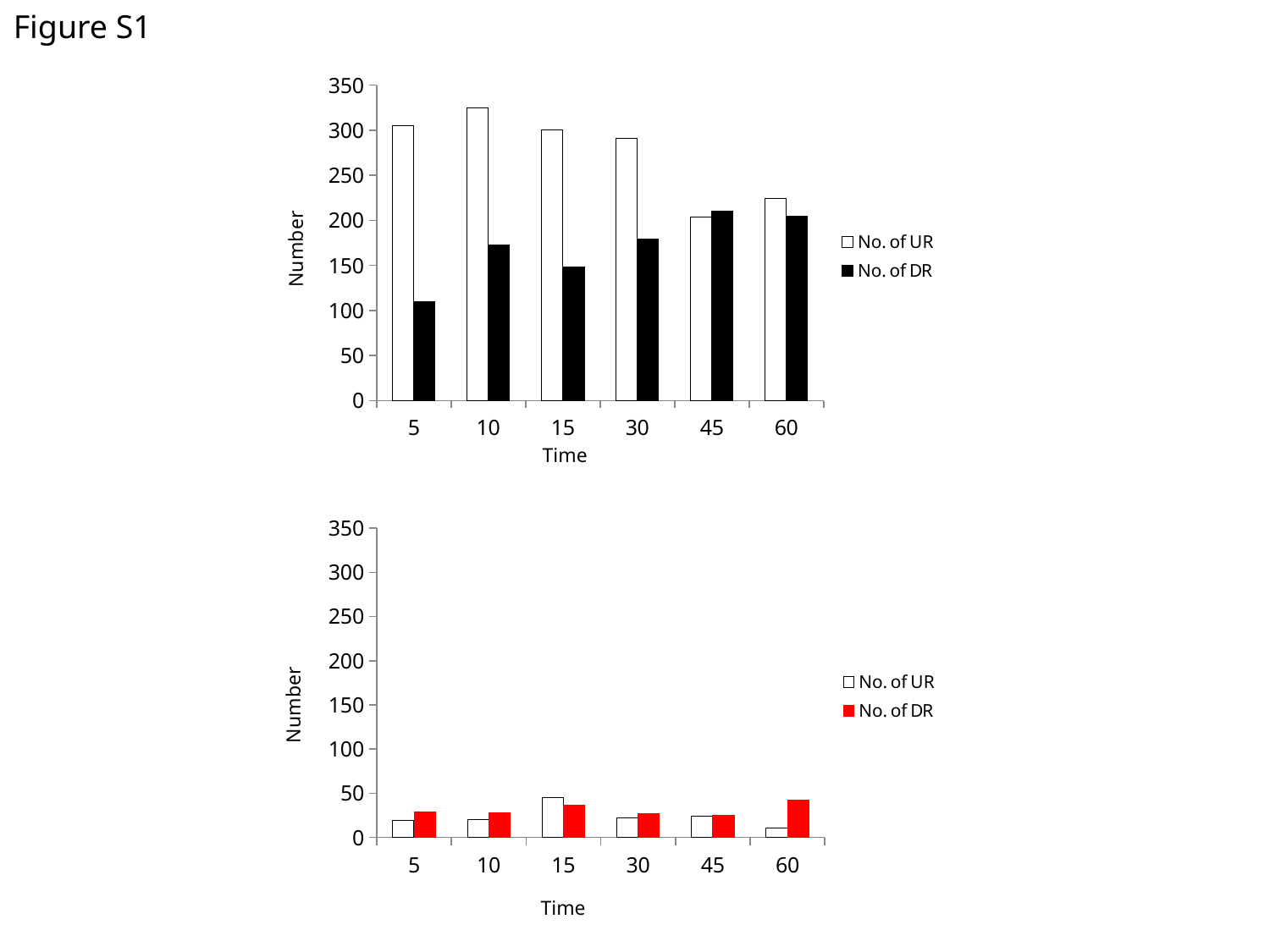

Figure S1
### Chart
| Category | No. of UR | No. of DR |
|---|---|---|
| 5 | 305.0 | 110.0 |
| 10 | 325.0 | 173.0 |
| 15 | 300.0 | 148.0 |
| 30 | 291.0 | 179.0 |
| 45 | 204.0 | 210.0 |
| 60 | 224.0 | 205.0 |Number
Time
### Chart
| Category | No. of UR | No. of DR |
|---|---|---|
| 5 | 19.0 | 29.0 |
| 10 | 20.0 | 28.0 |
| 15 | 45.0 | 37.0 |
| 30 | 22.0 | 27.0 |
| 45 | 24.0 | 25.0 |
| 60 | 11.0 | 42.0 |Number
Time
