# Supplementary material for: Time-resolved pathogenic gene expression analysis of the plant pathogen Xanthomonas oryzae pv. oryzae
Source: BMC Genomics. 2016 May 10;17:345. doi: 10.1186/s12864-016-2657-7 (PMC4862043; doi:10.1186/s12864-016-2657-7)
Supplement: Additional file 5: Table S4. — Differentially expressed genes in pathogenicity-activated Xoo cells (compared with the expression level at zero time with an RPKM threshold of 2.0 and a difference filter of 2.0). (DOCX 270 kb) [file 12864_2016_2657_MOESM5_ESM.docx]

**Table S4.** Differentially expressed genes in pathogenicity-activated *Xoo* cells (compared with the expression level at zero time with an RPKM threshold of 2.0 and a difference filter of 2.0)

| ***Gene name*** | ***FC** | **COG**  **category** | **Product** |
| --- | --- | --- | --- |
| **5 min upregulated** | | | |
| *Xoo0023* | 3.31 | S | hypothetical protein |
| *Xoo0038* | 2.05 | G | proline/betaine transporter |
| *Xoo0059* | 2.31 | O | protease Do |
| *Xoo0073* | 2.31 | L | ISXo8 transposase |
| *Xoo0078* | 2.56 | S | protein HrpD5 |
| *Xoo0096* | 2.48 | M | protein Hpa2 |
| *Xoo0097* | 2.11 | S | hypothetical protein |
| *Xoo0106* | 2.60 | L | transposase |
| *Xoo0134* | 2.01 | L | IS1479 transposase |
| *Xoo0163* | 2.54 | S | hypothetical protein |
| *Xoo0169* | 2.37 | K | xylose repressor-like protein |
| *Xoo0172* | 2.61 | S | hypothetical protein |
| *Xoo0177* | 2.02 | E | catalyzes the formation of glutamate from glutamine and alpha-ketoglutarate\|glutamate synthase subunit alpha |
| *Xoo0217* | 2.07 |  | hypothetical protein |
| *Xoo0218* | 2.37 | S | hypothetical protein |
| *Xoo0281* | 2.10 | G | cellulase |
| *Xoo0295* | 2.34 | L | IS1404 transposase |
| *Xoo0307* | 2.09 | Q | toluene tolerance protein |
| *Xoo0308* | 2.27 | Q | toluene tolerance protein |
| *Xoo0309* | 3.02 | Q | toluene tolerance protein |
| *Xoo0310* | 3.02 | Q | toluene tolerance protein |
| *Xoo0315* | 2.40 | L | hypothetical protein |
| *Xoo0316* | 2.60 | L | transposase |
| *Xoo0320* | 2.32 | L | ISxac1 transposase |
| *Xoo0329* | 2.50 | S | polyvinylalcohol dehydrogenase |
| *Xoo0337* | 4.74 | S | hypothetical protein |
| *Xoo0400* | 2.10 | S | hypothetical protein |
| *Xoo0405* | 2.87 | K | LysR family transcriptional regulator |
| *Xoo0406* | 2.24 | F | endonuclease |
| *Xoo0414* | 2.40 | L | transposase |
| *Xoo0421* | 2.76 | J | tRNA-dihydrouridine synthase A |
| *Xoo0422* | 5.05 |  | hypothetical protein |
| *Xoo0423* | 3.43 | T | two-component system regulatory protein |
| *Xoo0424* | 2.76 | T | two-component system sensor protein |
| *Xoo0431* | 2.03 | F | catalyzes the reversible phosphoryl transfer from adenosine triphosphate (ATP) to thymidine monophosphate (dTMP) to form thymidine diphosphate (dTDP)\|thymidylate kinase |
| *Xoo0488* | 2.04 | K | transcriptional regulator |
| *Xoo0500* | 2.07 | M | hypothetical protein |
| *Xoo0501* | 2.01 | M | dolichol-phosphate mannosyltransferase |
| *Xoo0516* | 2.34 | L | IS1404 transposase |
| *Xoo0546* | 2.40 | L | transposase |
| *Xoo0589* | 3.69 | M | hypothetical protein |
| *Xoo0609* | 2.42 | L | phage-related integrase |
| *Xoo0640* | 2.63 |  | hypothetical protein |
| *Xoo0656* | 2.05 | T | transcriptional regulator |
| *Xoo0669* | 2.09 | R | hypothetical protein |
| *Xoo0675* | 3.95 | P | Na+:H+ antiporter |
| *Xoo0716* | 2.52 |  | hypothetical protein |
| *Xoo0717* | 3.09 | M | hypothetical protein |
| *Xoo0719* | 2.27 |  | hypothetical protein |
| *Xoo0735* | 2.08 |  | hypothetical protein |
| *Xoo0768* | 5.88 |  | hypothetical protein |
| *Xoo0770* | 2.63 | L | ATP-dependent RNA helicase |
| *Xoo0786* | 2.74 | L | IS1478 transposase |
| *Xoo0823* | 3.55 | L | transposase |
| *Xoo0859* | 2.75 | K | transcriptional regulator |
| *Xoo0875* | 27.03 | L | transposase |
| *Xoo0898* | 2.11 | F | inosine-uridine preferring nucleoside hydrolase |
| *Xoo0900* | 3.19 | S | hypothetical protein |
| *Xoo0901* | 30.93 | P | TonB-dependent receptor |
| *Xoo0902* | 9.28 | C | glycerophosphodiester phosphodiesterase |
| *Xoo0903* | 4.41 | V | hypothetical protein |
| *Xoo0907* | 2.12 | P | bacterioferritin |
| *Xoo0929* | 2.22 | K | transcriptional regulator |
| *Xoo0930* | 2.17 | T | hypothetical protein |
| *Xoo0948* | 2.52 | K | hypothetical protein |
| *Xoo0953* | 2.74 | S | hypothetical protein |
| *Xoo0958* | 2.67 |  | hypothetical protein |
| *Xoo0959* | 5.83 | L | transposase |
| *Xoo0960* | 2.09 | L | transposase |
| *Xoo1008* | 2.66 | L | ISxac1 transposase |
| *Xoo1011* | 8.16 | L | transposase |
| *Xoo1014* | 4.63 | M | hypothetical protein |
| *Xoo1038* | 2.65 | M | murein hydrolase D |
| *Xoo1046* | 2.17 | L | XfasO2027\|site-specific DNA methylase |
| *Xoo1047* | 2.29 | S | Vgr-related protein |
| *Xoo1068* | 2.57 | M | outer membrane protein Slp |
| *Xoo1071* | 2.10 | R | methanol dehydrogenase regulator |
| *Xoo1075* | 3.70 | J | cleaves off formyl group from N-terminal methionine residues of newly synthesized proteins; binds iron(2+)\|peptide deformylase |
| *Xoo1076* | 2.15 |  | cellulase S |
| *Xoo1098* | 6.25 | G | amylosucrase or alpha amylase |
| *Xoo1099* | 2.52 | P | TonB-dependent receptor |
| *Xoo1100* | 2.48 | G | sugar transporter |
| *Xoo1102* | 5.26 | IQ | catalyzes the first of the two reduction steps in the elongation cycle of fatty acid synthesis\|3-ketoacyl-ACP reductase |
| *Xoo1103* | 8.69 | C | Mg++/citrate complex transporter |
| *Xoo1104* | 16.70 | P | porin |
| *Xoo1117* | 7.46 | C | involved in the transport of C4-dicarboxylates across the membrane\|C4-dicarboxylate transporter DctA |
| *Xoo1136* | 2.60 | L | transposase |
| *Xoo1184* | 2.51 | SI | long-chain acyl-CoA synthetase |
| *Xoo1186* | 3.72 | R | Avin0576\|short chain dehydrogenase |
| *Xoo1235* | 2.44 | L | Avin3395\|transposase |
| *Xoo1269* | 2.83 | R | proline imino-peptidase |
| *Xoo1272* | 2.31 | L | transposase |
| *Xoo1273* | 4.18 | C | quinol oxidase subunit I |
| *Xoo1274* | 2.74 | C | quinol oxidase subunit II |
| *Xoo1288* | 2.22 | R | hypothetical protein |
| *Xoo1292* | 2.12 | S | hypothetical protein |
| *Xoo1293* | 3.13 | S | hypothetical protein |
| *Xoo1302* | 2.06 | M | nucleotidyl transferase |
| *Xoo1303* | 2.44 | R | hypothetical protein |
| *Xoo1304* | 2.29 | S | hypothetical protein |
| *Xoo1317* | 2.32 | G | alpha-L-arabinofuranosidase |
| *Xoo1362* | 2.64 | P | cation:proton antiporter |
| *Xoo1376* | 4.10 | S | hypothetical protein |
| *Xoo1379* | 3.60 | T | HrpG protein |
| *Xoo1380* | 2.14 | T | HrpX protein |
| *Xoo1410* | 3.02 |  | hypothetical protein |
| *Xoo1429* | 5.43 | S | hypothetical protein |
| *Xoo1443* | 2.09 | L | ISXo8 transposase |
| *Xoo1478* | 2.15 | L | transposase |
| *Xoo1502* | 2.56 | R | nitrilase |
| *Xoo1509* | 2.13 | S | hypothetical protein |
| *Xoo1511* | 4.02 |  | peptidoglycan-associated outer membrane lipoprotein |
| *Xoo1512* | 2.80 |  | hypothetical protein |
| *Xoo1516* | 6.28 | S | hypothetical protein |
| *Xoo1546* | 2.69 | T | hypothetical protein |
| *Xoo1655* | 4.99 | S | hypothetical protein |
| *Xoo1664* | 2.81 | R | hypothetical protein |
| *Xoo1665* | 2.59 | U | hypothetical protein |
| *Xoo1666* | 2.18 | U | hypothetical protein |
| *Xoo1667* | 2.20 | S | hypothetical protein |
| *Xoo1722* | 3.38 |  | hypothetical protein |
| *Xoo1756* | 2.92 | R | hypothetical protein |
| *Xoo1803* | 5.77 | R | hypothetical protein |
| *Xoo1817* | 3.27 | E | catalyzes the formation of O-acetyl -L-homoserine from L-homoserine and acetyl-CoA\|homoserine O-acetyltransferase |
| *Xoo1820* | 2.37 | E | homoserine dehydrogenase |
| *Xoo1833* | 3.84 | L | hypothetical protein |
| *Xoo1867* | 2.27 | G | hydrolyzes the terminal non-reducing N-acetyl-D-hexosamine residues in N-acetyl-beta-D-hexosaminides\|beta-hexosaminidase |
| *Xoo1887* | 2.28 | T | hypothetical protein |
| *Xoo1890* | 2.66 | L | ISxac1 transposase |
| *Xoo1901* | 10.57 | G | MFS transporter |
| *Xoo1925* | 3.31 | S | hypothetical protein |
| *Xoo1952* | 3.68 | I | PHB depolymerase |
| *Xoo1994* | 23.01 | P | bacterioferritin |
| *Xoo1998* | 5.80 | K | MarR family transcriptional regulator |
| *Xoo1999* | 5.04 | M | outer membrane efflux protein |
| *Xoo2000* | 3.91 | V | multidrug resistance efflux pump |
| *Xoo2001* | 3.96 | P | multidrug resistance membrane translocase |
| *Xoo2005* | 2.86 |  | hypothetical protein |
| *Xoo2012* | 21.53 | L | Reut4275\|IS30 family transposase |
| *Xoo2056* | 2.13 | L | Reut4275\|IS30 family transposase |
| *Xoo2073* | 2.14 | E | 5-methyltetrahydrofolate--homocysteinemethyltransferase |
| *Xoo2074* | 2.21 | E | 5-methyltetrahydrofolate--homocysteinemethyltransferase |
| *Xoo2075* | 2.77 | KQ | transcriptional regulator |
| *Xoo2076* | 3.13 | I | acyl-CoA dehydrogenase |
| *Xoo2082* | 2.01 | L | hypothetical protein |
| *Xoo2105* | 4.59 | L | ISxac1 transposase |
| *Xoo2114* | 4.63 | S | TonB-like protein |
| *Xoo2132* | 3.62 | L | ISXo8 transposase |
| *Xoo2133* | 2.89 | R | hypothetical protein |
| *Xoo2145* | 2.99 | S | hypothetical protein |
| *Xoo2198* | 5.91 | K | LysR family transcriptional regulator |
| *Xoo2199* | 3.60 | S | AGl1036\|hypothetical protein |
| *Xoo2228* | 2.35 | T | two-component system sensor protein |
| *Xoo2231* | 2.24 | G | Prop transport protein |
| *Xoo2280* | 2.21 | L | Avin3395\|transposase |
| *Xoo2281* | 2.45 | L | IS163, transposase |
| *Xoo2303* | 2.03 | R | hypothetical protein |
| *Xoo2323* | 2.26 | T | two-component system sensor protein |
| *Xoo2330* | 2.22 | T | sensor histidine kinase |
| *Xoo2342* | 2.45 | O | extracellular protease |
| *Xoo2376* | 2.25 | P | cation efflux system protein |
| *Xoo2396* | 2.50 | Q | catalyzing the hydrolysis of 4-imidazolone-5-propionate to N-formimidoyl-L-glutamate, the third step in the histidine degradation pathway\|imidazolonepropionase |
| *Xoo2397* | 3.09 | F | catalyzes the deimination of N-formimino-L-glutamate to ammonia and N-formyl-L-glutamate\|N-formimino-L-glutamate deiminase |
| *Xoo2398* | 2.51 | E | catalyzes the degradation of histidine to urocanate and ammmonia\|histidine ammonia-lyase |
| *Xoo2411* | 2.29 | E | aminotransferase |
| *Xoo2460* | 12.77 | P | polyphosphate-selective porin O |
| *Xoo2461* | 4.61 | P | phosphate binding protein |
| *Xoo2462* | 9.31 | P | phosphate ABC transporter substrate-binding protein |
| *Xoo2463* | 11.67 | P | phosphate ABC transporter permease |
| *Xoo2464* | 10.97 | P | phosphate ABC transporter permease |
| *Xoo2465* | 10.13 | P | ATP-binding protein; PstABCS is an ATP dependent phosphate uptake system which is responsible for inorganic phosphate uptake during phosphate starvation\|phosphate transporter ATP-binding protein |
| *Xoo2466* | 7.98 | P | phosphate regulon transcriptional regulator |
| *Xoo2467* | 6.77 | S | hypothetical protein |
| *Xoo2470* | 2.09 | L | transposase |
| *Xoo2536* | 2.97 | K | TetR family transcriptional regulator |
| *Xoo2537* | 2.07 | I | 3-hydroxyacyl-CoA dehydrogenase |
| *Xoo2538* | 2.05 | I | Catalyzes the synthesis of acetoacetyl coenzyme A from two molecules of acetyl coenzyme A. It can also act as a thiolase, catalyzing the reverse reaction and generating two-carbon units from the four-carbon product of fatty acid oxidation\|acetyl-CoA acetyltransferase |
| *Xoo2539* | 2.22 | P | hypothetical protein |
| *Xoo2543* | 2.00 | S | hypothetical protein |
| *Xoo2627* | 4.72 | L | Reut4275\|IS30 family transposase |
| *Xoo2654* | 2.08 | V | transporter |
| *Xoo2677* | 2.05 |  | hypothetical protein |
| *Xoo2685* | 2.54 | E | ethanolamine ammonia-lyase light subunit |
| *Xoo2686* | 2.76 | L | transposase |
| *Xoo2713* | 4.02 | F | ribonuclease |
| *Xoo2714* | 3.02 | S | hypothetical protein |
| *Xoo2718* | 2.51 | S | hypothetical protein |
| *Xoo2738* | 3.86 | U | virulence protein |
| *Xoo2745* | 5.35 | L | RSp1628\|hypothetical protein |
| *Xoo2750* | 2.07 | L | responsible for recognizing base lesions in the genome and initiating base excision DNA repair\|3-methyladenine DNA glycosylase |
| *Xoo2778* | 2.10 | M | outer membrane protein OprN |
| *Xoo2779* | 2.16 | P | transporter |
| *Xoo2799* | 2.24 | P | drug resistance translocase |
| *Xoo2803* | 2.66 | K | TetR/AcrR family transcriptional regulator |
| *Xoo2804* | 2.03 | M | multidrug resistance protein |
| *Xoo2808* | 4.75 | L | transposase |
| *Xoo2809* | 4.85 | K | LacI family transcriptional regulator |
| *Xoo2810* | 6.84 | G | multiphosphoryl transfer protein |
| *Xoo2811* | 9.06 | G | 1-phosphofructokinase |
| *Xoo2812* | 7.85 | G | PTS system fructose-specific transporter subunit IIBC |
| *Xoo2813* | 4.84 | M | regulator of pathogenicity factors |
| *Xoo2861* | 2.30 | R | beta-ketoadipate enol-lactone hydrolase |
| *Xoo2893* | 2.60 | L | transposase |
| *Xoo2930* | 2.01 | R | hypothetical protein |
| *Xoo2943* | 3.41 | R | ubiquinone biosynthesis protein |
| *Xoo2944* | 2.62 | R | hypothetical protein |
| *Xoo2968* | 2.30 |  | hypothetical protein |
| *Xoo2986* | 2.06 | M | glycosyltransferase |
| *Xoo3071* | 2.99 | S | hypothetical protein |
| *Xoo3101* | 2.04 | T | histidine kinase/response regulator hybrid protein |
| *Xoo3102* | 2.58 | H | siroheme synthase |
| *Xoo3125* | 2.59 | O | peptidase |
| *Xoo3147* | 2.07 | T | sensor histidine kinase |
| *Xoo3157* | 2.27 | L | transposase |
| *Xoo3174* | 2.05 | G | protein GumG |
| *Xoo3175* | 2.29 | G | protein GumF |
| *Xoo3176* | 2.82 | S | protein GumE |
| *Xoo3177* | 3.95 | M | protein GumD |
| *Xoo3178* | 4.12 | DM | protein GumC |
| *Xoo3179* | 4.82 | M | protein GumB |
| *Xoo3243* | 2.60 | L | transposase |
| *Xoo3317* | 3.30 | S | hypothetical protein |
| *Xoo3351* | 3.39 | L | transposase |
| *Xoo3383* | 2.08 | L | transposase |
| *Xoo3389* | 4.82 |  | acetyltransferase |
| *Xoo3408* | 2.37 | H | siroheme synthase |
| *Xoo3445* | 3.28 | L | transposase |
| *Xoo3451* | 2.09 | T | serine/threonine kinase |
| *Xoo3490* | 2.25 | S | hypothetical protein |
| *Xoo3492* | 2.36 | S | hypothetical protein |
| *Xoo3501* | 2.06 | S | RSp0176\|hypothetical protein |
| *Xoo3544* | 2.11 | U | membrane fusion protein RaxA |
| *Xoo3552* | 2.30 | L | IS1404 transposase |
| *Xoo3554* | 2.60 | L | transposase |
| *Xoo3618* | 3.93 | G | xylanase |
| *Xoo3658* | 2.19 | S | hypothetical protein |
| *Xoo3659* | 2.58 | T | two-component system regulatory protein |
| *Xoo3663* | 2.09 | S | hypothetical protein |
| *Xoo3664* | 2.36 | O | glutaredoxin |
| *Xoo3665* | 3.74 | O | hypothetical protein |
| *Xoo3666* | 16.20 | T | two-component system regulatory protein |
| *Xoo3667* | 11.38 | T | two-component system sensor protein |
| *Xoo3668* | 2.15 | P | catalyzes the reversible transfer of the terminal phosphate of ATP to form a long chain polyphosphate\|polyphosphate kinase |
| *Xoo3698* | 3.31 | C | 2,4-dienoyl-CoA reductase |
| *Xoo3699* | 3.15 |  | hypothetical protein |
| *Xoo3708* | 2.27 | L | transposase |
| *Xoo3714* | 3.14 | L | IS30 family transposase |
| *Xoo3764* | 2.24 | E | acyl-CoA thioesterase |
| *Xoo3792* | 2.61 | R | phosphoanhydride phosphohydrolase |
| *Xoo3793* | 15.36 | P | TonB-dependent receptor |
| *Xoo3797* | 2.78 | E | hypothetical protein |
| *Xoo3809* | 2.08 | S | hypothetical protein |
| *Xoo3811* | 3.72 |  | hypothetical protein |
| *Xoo3836* | 2.01 | S | hypothetical protein |
| *Xoo3847* | 2.32 |  | hypothetical protein |
| *Xoo3874* | 2.21 | L | ISXo8 transposase |
| *Xoo3878* | 3.27 | S | nodulin 21-like protein |
| *Xoo3909* | 2.25 | P | TonB-dependent receptor |
| *Xoo3928* | 2.97 | L | transposase |
| *Xoo3940* | 2.78 |  | hypothetical protein |
| *Xoo3941* | 2.72 | S | hypothetical protein |
| *Xoo3977* | 2.22 | R | hypothetical protein |
| *Xoo4045* | 2.44 | S | hypothetical protein |
| *Xoo4063* | 2.22 | M | hypothetical protein |
| *Xoo4076* | 2.26 | R | nuclease |
| *Xoo4121* | 5.53 | L | transposase |
| *Xoo4122* | 2.38 | L | hypothetical protein |
| *Xoo4143* | 2.60 | L | transposase |
| *Xoo4147* | 2.40 | L | transposase |
| *Xoo4149* | 22.00 | P | bacterioferritin |
| *Xoo4151* | 2.48 | L | hydrolyzes diadenosine polyphosphate\|dinucleoside polyphosphate hydrolase |
| *Xoo4171* | 2.66 | L | ISxac1 transposase |
| *Xoo4199* | 2.23 | M | hypothetical protein |
| *Xoo4201* | 2.11 | T | two-component system sensor protein |
| *Xoo4208* | 2.56 | S | hypothetical protein |
| *Xoo4290* | 2.20 | L | ISxac1 transposase |
| *Xoo4294* | 2.77 | C | NADH dehydrogenase |
| *Xoo4349* | 3.48 | G | MFS transporter |
| *Xoo4358* | 2.31 | K | LysR family transcriptional regulator |
| *Xoo4368* | 2.09 | L | IS1404 transposase |
| *Xoo4385* | 2.80 | S | hypothetical protein |
| *Xoo4392* | 2.02 | S | hypothetical protein |
| *Xoo4409* | 2.78 | S | hypothetical protein |
| *Xoo4428* | 2.32 | G | secreted xylanase |
| *Xoo4463* | 2.27 | S | Psyr4406\|hypothetical protein |
| *Xoo4464* | 2.19 |  | hypothetical protein |
| *Xoo4475* | 2.64 | Q | hemagglutinin |
| *Xoo4554* | 10.69 | P | hypothetical protein |
| *Xoo4555* | 22.82 | P | alkaline phosphatase |
| *Xoo4560* | 2.11 | L | transposase |
| *Xoo4565* | 2.15 | H | hypothetical protein |
| *Xoo4583* | 3.22 | R | hypothetical protein |
| *Xoo4603* | 2.02 | S | hypothetical protein |
| *Xoo4709* | 2.33 |  | identified by sequence similarity; ORF located using Blastx/FrameD\|hypothetical protein |
| *Xoo4718* | 2.79 |  | hypothetical protein |
| *Xoo4721* | 3.99 |  | hypothetical protein |
| *Xoo4725* | 2.11 |  | hypothetical protein |
| *Xoo4744* | 3.82 | SI | identified by sequence similarity; ORF located using Blastx/FrameD\|carbamoyl-phosphate synthase large subunit |
| *Xoo4766* | 4.01 |  | hypothetical protein |
| *Xoo4794* | 2.33 |  | hypothetical protein |
| *Xoo4799* | 2.89 | L | identified by sequence similarity; ORF located using Blastx/FrameD\|IS1404 transposase |
| *Xoo4806* | 2.10 | S | hypothetical protein |
| *Xoo4823* | 2.04 |  | hypothetical protein |
| *Xoo4951* | 4.05 |  | hypothetical protein |
| *YXoop10* | 2.45 |  | hypothetical protein |
| **5 min downregulated** | | | |
| *Xoo0120* | 0.41 | L | ISxac1 transposase |
| *Xoo0128* | 0.41 | L | ISxac1 transposase |
| *Xoo0283* | 0.28 | G | cellulase |
| *Xoo0297* | 0.42 | L | transposase |
| *Xoo0394* | 0.36 | P | TonB-dependent receptor |
| *Xoo0556* | 0.44 | I | an AccC homodimer forms the biotin carboxylase subunit of the acetyl CoA carboxylase, an enzyme that catalyzes the formation of malonyl-CoA, which in turn controls the rate of fatty acid metabolism\|acetyl-CoA carboxylase biotin carboxylase subunit |
| *Xoo0557* | 0.41 | S | crystal structure of protein from Xanthomonas shows pentameric toroidal structure; physiological function is unknown\|hypothetical protein |
| *Xoo0558* | 0.43 | I | composes the biotin carboxyl carrier protein subunit of the acetyl-CoA carboxylase complex, the enzyme that catalyzes the carboxylation of acetyl-CoA to malonyl-CoA, which in turn controls the rate of fatty acid metabolism\|acetyl-CoA carboxylase biotin carboxyl carrier protein subunit |
| *Xoo0659* | 0.46 | E | amino acid transporter |
| *Xoo0701* | 0.47 |  | hypothetical protein |
| *Xoo0748* | 0.45 | S | hypothetical protein |
| *Xoo0749* | 0.44 | L | transposase |
| *Xoo0761* | 0.32 | I | beta-ketoacyl- synthase I |
| *Xoo0762* | 0.27 | I | catalyzes the dehydration of (3R)-3-hydroxydecanoyl-ACP to 2,3-decenoyl-ACP or 3,4-decenoyl-ACP\|3-hydroxydecanoyl-ACP dehydratase |
| *Xoo0814* | 0.41 | L | ISxac1 transposase |
| *Xoo0880* | 0.48 | I | ACP S-malonyltransferase |
| *Xoo0881* | 0.38 | IQ | Catalyzes the first of the two reduction steps in the elongation cycle of fatty acid synthesis\|3-ketoacyl-ACP reductase |
| *Xoo0892* | 0.18 | G | catalyzes the formation of pyruvate and succinate from 2-methylisocitrate\|2-methylisocitrate lyase |
| *Xoo0893* | 0.36 | C | catalyzes the synthesis of 2-methylcitrate from propionyl-CoA and oxaloacetate; also catalyzes the condensation of oxaloacetate with acetyl-CoA but with a lower specificity\|methylcitrate synthase |
| *Xoo1332* | 0.41 | L | ISxac1 transposase |
| *Xoo1357* | 0.46 | Q | hypothetical protein |
| *Xoo1358* | 0.22 | I | hypothetical protein |
| *Xoo1359* | 0.03 | P | citrate-dependent iron transporter |
| *Xoo1360* | 0.03 | G | 4-hydroxy-2-oxovalerate aldolase |
| *Xoo1454* | 0.35 | S | hypothetical protein |
| *Xoo1604* | 0.38 | P | ferric enterobactin receptor |
| *Xoo1608* | 0.49 | L | transposase |
| *Xoo1771* | 0.45 | S | hypothetical protein |
| *Xoo1823* | 0.34 | S | cold shock domain-containing protein |
| *Xoo1842* | 0.19 | C | methylmalonate-semialdehyde dehydrogenase |
| *Xoo1843* | 0.18 | I | acyl-CoA dehydrogenase |
| *Xoo1844* | 0.23 | S | Catalyzes the reversible hydration of unsaturated fatty acyl-CoA to beta-hydroxyacyl-CoA\|enoyl-CoA hydratase |
| *Xoo1845* | 0.32 | I | enoyl-CoA hydratase |
| *Xoo1846* | 0.27 | I | 3-hydroxyisobutyrate dehydrogenase |
| *Xoo1982* | 0.46 | S | protein U |
| *Xoo1992* | 0.07 | P | iron permease |
| *Xoo1993* | 0.24 | R | penicillin acylase II |
| *Xoo2054* | 0.47 | L | transposase |
| *Xoo2095* | 0.34 | L | transposase |
| *Xoo2152* | 0.47 |  | hypothetical protein |
| *Xoo2338* | 0.47 | C | ferredoxin-NADP reductase |
| *Xoo2351* | 0.39 | E | hypothetical protein |
| *Xoo2352* | 0.44 | G | beta-glucosidase |
| *Xoo2510* | 0.47 | O | molecular chaperone\|heat shock protein 90 |
| *Xoo2558* | 0.47 | S | chemotaxis protein |
| *Xoo2559* | 0.45 |  | hypothetical protein |
| *Xoo2567* | 0.48 | N | required for the assembly of the flagellar basal body P-ring\|flagellar basal body P-ring biosynthesis protein FlgA |
| *Xoo2568* | 0.48 | NT | chemotaxis protein |
| *Xoo2569* | 0.36 | N | with FlgF and C makes up the proximal portion of the flagellar basal body rod\|flagellar basal-body rod protein FlgB |
| *Xoo2570* | 0.32 | N | with FlgF and B makes up the proximal portion of the flagellar basal body rod\|flagellar basal body rod protein FlgC |
| *Xoo2571* | 0.30 | N | acts as a scaffold for the assembly of hook proteins onto the flagellar basal body rod\|flagellar basal body rod modification protein |
| *Xoo2572* | 0.25 | N | the hook connects flagellar basal body to the flagellar filament\|flagellar hook protein FlgE |
| *Xoo2574* | 0.40 | N | FlgF, with FlgB and C, makes up the proximal portion of the flagellar basal body rod\|flagellar basal body rod protein FlgF |
| *Xoo2575* | 0.38 | N | makes up the distal portion of the flagellar basal body rod\|flagellar basal body rod protein FlgG |
| *Xoo2576* | 0.37 | N | part of the flagellar basal body which consists of four rings L,P, S and M mounted on a central rod\|flagellar basal body L-ring protein |
| *Xoo2577* | 0.38 | N | part of the basal body which consists of four rings L, P, S, and M mounted on a central rod; Vibrio parahaemolyticus, Yersinia, Bradyrhizobium and other bacteria have two copies of this and other flagellar genes; the V. parahaemolyticus protein is associated with the polar flagella and the Bradyrhizobium protein is associated with the thick flagellum\|flagellar basal body P-ring biosynthesis protein FlgA |
| *Xoo2578* | 0.41 | NU | Flagellum-specific muramidase which hydrolyzes the peptidoglycan layer to assemble the rod structure in the periplasmic space\|flagellar rod assembly protein/muramidase FlgJ |
| *Xoo2579* | 0.44 | N | with FlgL acts as a hook filament junction protein to join the flagellar filament to the hook\|flagellar hook-associated protein FlgK |
| *Xoo2582* | 0.49 | N | flagellar protein |
| *Xoo2587* | 0.47 | K | RNA polymerase sigma-54 factor |
| *Xoo2588* | 0.44 | T | response regulator |
| *Xoo2591* | 0.43 | S | acyl carrier protein |
| *Xoo2592* | 0.46 | I | 3-oxoacyl-ACP synthase |
| *Xoo2602* | 0.47 | N | flagellar protein |
| *Xoo2605* | 0.45 | N | flagellar FliJ protein |
| *Xoo2606* | 0.49 | N | flagellar protein |
| *Xoo2608* | 0.47 | N | with FliG and FliN makes up the switch complex which is involved in switching the direction of the flagella rotation\|flagellar motor switch protein FliM |
| *Xoo2609* | 0.46 | N | flagellar protein |
| *Xoo2618* | 0.47 | N | membrane protein involved in the flagellar export apparatus\|flagellar biosynthesis protein FlhA |
| *Xoo2619* | 0.42 | N | Positive regulator of class III flagellar genes\|flagellar biosynthesis regulator FlhF |
| *Xoo2620* | 0.39 | D | flagellar biosynthesis switch protein |
| *Xoo2621* | 0.39 | K | RNA polymerase sigma factor FliA |
| *Xoo2622* | 0.46 | T | chemotaxis protein |
| *Xoo2787* | 0.42 | T | transcriptional regulator |
| *Xoo2814* | 0.34 | L | transposase |
| *Xoo2819* | 0.18 | L | ISxac3 transposase |
| *Xoo2823* | 0.41 | L | ISxac1 transposase |
| *Xoo2831* | 0.49 | N | Homologous to MotB. These organism have both MotB and MotD. With MotC (a MotA homolog) forms the ion channels that couple flagellar rotation to proton/sodium motive force across the membrane and forms the stator elements of the rotary flagellar machine. Either MotAB or MotCD is sufficient for swimming, but both are necessary for swarming motility\|flagellar motor protein MotD |
| *Xoo2832* | 0.41 | D | chromosome partioning protein |
| *Xoo2833* | 0.45 | S | chemotaxis protein |
| *Xoo2835* | 0.48 | T | chemotaxis response regulator |
| *Xoo2842* | 0.33 | NT | chemotaxis protein |
| *Xoo2849* | 0.39 | M | hypothetical protein |
| *Xoo2857* | 0.40 | NT | chemotaxis protein methyltransferase |
| *Xoo2897* | 0.19 |  | hypothetical protein |
| *Xoo2898* | 0.17 |  | hypothetical protein |
| *Xoo2899* | 0.18 | P | ferrous iron transport protein B |
| *Xoo2900* | 0.16 | P | ferrous iron transport protein |
| *Xoo3320* | 0.48 | P | Oar protein |
| *Xoo3376* | 0.49 |  | hypothetical protein |
| *Xoo3423* | 0.49 | P | catalase |
| *Xoo3780* | 0.05 |  | outer membrane hemin receptor |
| *Xoo3781* | 0.03 | S | hypothetical protein |
| *Xoo3894* | 0.34 | L | transposase |
| *Xoo3899* | 0.19 | L | ISXo8 transposase |
| *Xoo4113* | 0.28 | S | hypothetical protein |
| *Xoo4150* | 0.06 | P | bacterioferritin-associated ferredoxin |
| *Xoo4288* | 0.46 | O | 60 kDa chaperone family; promotes refolding of misfolded polypeptides especially under stressful conditions; forms two stacked rings of heptamers to form a barrel-shaped 14mer; ends can be capped by GroES; misfolded proteins enter the barrel where they are refolded when GroES binds; many bacteria have multiple copies of the groEL gene which are active under different environmental conditions; the B.japonicum protein in this cluster is expressed constitutively; in Rhodobacter, Corynebacterium and Rhizobium this protein is essential for growth\|molecular chaperone GroEL |
| *Xoo4377* | 0.10 | I | biotin carboxylase |
| *Xoo4378* | 0.07 | I | acyl-CoA carboxyltransferase subunit beta |
| *Xoo4379* | 0.06 | I | acyl-CoA dehydrogenase |
| *Xoo4380* | 0.31 | K | AcrR family transcriptional regulator |
| *Xoo4396* | 0.49 | P | TonB-dependent receptor |
| *Xoo4431* | 0.45 | P | TonB-dependent receptor |
| *Xoo4440* | 0.04 | L | transposase |
| *Xoo4446* | 0.14 | I | hypothetical protein |
| *Xoo4447* | 0.20 | C | oxidoreductase |
| *Xoo4545* | 0.25 | S | hypothetical protein |
| *Xoo4546* | 0.14 | S | hypothetical protein |
| *Xoo4759* | 0.42 | S | hypothetical protein |
| **10 min upregulated** | | | |
| *Xoo0038* | 3.30 | G | proline/betaine transporter |
| *Xoo0057* | 2.04 | T | two-component system sensor protein |
| *Xoo0058* | 2.08 | K | histone H1-like protein |
| *Xoo0059* | 3.10 | O | protease Do |
| *Xoo0062* | 2.36 |  | hypothetical protein |
| *Xoo0064* | 2.37 | L | transposase |
| *Xoo0075* | 2.26 | S | protein HpaB |
| *Xoo0078* | 2.44 | S | protein HrpD5 |
| *Xoo0081* | 2.04 | U | part of a set of proteins involved in the infection of eukaryotic cells; in plant pathogens involved in the hypersensitivity response\|type III secretion system protein |
| *Xoo0096* | 3.27 | M | protein Hpa2 |
| *Xoo0097* | 2.81 | S | hypothetical protein |
| *Xoo0099* | 3.13 | L | transposase |
| *Xoo0103* | 2.44 | S | hypothetical protein |
| *Xoo0134* | 2.02 | L | IS1479 transposase |
| *Xoo0135* | 2.51 | C | dihydrolipoamide acyltransferase |
| *Xoo0163* | 2.46 | S | hypothetical protein |
| *Xoo0169* | 2.31 | K | xylose repressor-like protein |
| *Xoo0172* | 7.12 | S | hypothetical protein |
| *Xoo0177* | 2.96 | E | catalyzes the formation of glutamate from glutamine and alpha-ketoglutarate\|glutamate synthase subunit alpha |
| *Xoo0178* | 2.41 | E | glutamate synthase is composed of subunits alpha and beta; beta subunit is a flavin adenine dinucleotide-NADPH dependent oxidoreductase; provides electrons to the alpha subunit, which binds L-glutamine and 2-oxoglutarate and forms L-glutamate\|glutamate synthase subunit beta |
| *Xoo0218* | 2.52 | S | hypothetical protein |
| *Xoo0222* | 3.87 | S | Vgr-related protein |
| *Xoo0223* | 2.23 | S | hypothetical protein |
| *Xoo0281* | 3.43 | G | cellulase |
| *Xoo0305* | 2.34 | M | lipoprotein |
| *Xoo0306* | 2.23 | R | hypothetical protein |
| *Xoo0307* | 2.77 | Q | toluene tolerance protein |
| *Xoo0308* | 2.47 | Q | toluene tolerance protein |
| *Xoo0309* | 2.74 | Q | toluene tolerance protein |
| *Xoo0310* | 2.59 | Q | toluene tolerance protein |
| *Xoo0315* | 2.66 | L | hypothetical protein |
| *Xoo0320* | 2.05 | L | ISxac1 transposase |
| *Xoo0326* | 2.14 | L | IS1404 transposase |
| *Xoo0329* | 3.13 | S | polyvinylalcohol dehydrogenase |
| *Xoo0337* | 3.99 | S | hypothetical protein |
| *Xoo0368* | 2.05 | L | ISXo8 transposase |
| *Xoo0374* | 2.23 | L | transposase |
| *Xoo0400* | 2.35 | S | hypothetical protein |
| *Xoo0405* | 2.34 | K | LysR family transcriptional regulator |
| *Xoo0406* | 2.57 | F | endonuclease |
| *Xoo0421* | 2.63 | J | tRNA-dihydrouridine synthase A |
| *Xoo0422* | 4.53 |  | hypothetical protein |
| *Xoo0423* | 3.71 | T | two-component system regulatory protein |
| *Xoo0424* | 3.20 | T | two-component system sensor protein |
| *Xoo0425* | 2.92 | S | hypothetical protein |
| *Xoo0426* | 2.72 | S | hypothetical protein |
| *Xoo0454* | 2.16 | R | competence protein F |
| *Xoo0543* | 2.13 | I | hypothetical protein |
| *Xoo0589* | 3.51 | M | hypothetical protein |
| *Xoo0600* | 2.03 | S | hypothetical protein |
| *Xoo0609* | 2.22 | L | phage-related integrase |
| *Xoo0640* | 3.23 |  | hypothetical protein |
| *Xoo0662* | 2.08 | O | metallopeptidase |
| *Xoo0667* | 2.01 | S | hypothetical protein |
| *Xoo0669* | 2.41 | R | hypothetical protein |
| *Xoo0670* | 2.06 | L | DNA polymerase related protein |
| *Xoo0675* | 4.44 | P | Na+:H+ antiporter |
| *Xoo0677* | 2.26 | V | ABC transporter |
| *Xoo0716* | 2.47 |  | hypothetical protein |
| *Xoo0717* | 7.07 | M | hypothetical protein |
| *Xoo0719* | 2.56 |  | hypothetical protein |
| *Xoo0740* | 2.34 | L | transposase |
| *Xoo0768* | 10.62 |  | hypothetical protein |
| *Xoo0770* | 4.63 | L | ATP-dependent RNA helicase |
| *Xoo0823* | 3.74 | L | transposase |
| *Xoo0859* | 2.67 | K | transcriptional regulator |
| *Xoo0875* | 21.55 | L | transposase |
| *Xoo0900* | 2.76 | S | hypothetical protein |
| *Xoo0901* | 81.68 | P | TonB-dependent receptor |
| *Xoo0902* | 41.75 | C | glycerophosphodiester phosphodiesterase |
| *Xoo0903* | 25.64 | V | hypothetical protein |
| *Xoo0904* | 2.76 | L | ISXo8 transposase |
| *Xoo0909* | 2.94 | O | low molecular weight heat shock protein |
| *Xoo0929* | 2.42 | K | transcriptional regulator |
| *Xoo0930* | 2.08 | T | hypothetical protein |
| *Xoo0948* | 2.65 | K | hypothetical protein |
| *Xoo0949* | 2.33 | K | transcriptional regulator |
| *Xoo0953* | 2.73 | S | hypothetical protein |
| *Xoo0958* | 3.27 |  | hypothetical protein |
| *Xoo0959* | 11.72 | L | transposase |
| *Xoo1004* | 2.57 | M | hypothetical protein |
| *Xoo1011* | 3.39 | L | transposase |
| *Xoo1014* | 6.83 | M | hypothetical protein |
| *Xoo1030* | 2.01 | L | Bcep0071\|DNA replication protein |
| *Xoo1038* | 2.46 | M | murein hydrolase D |
| *Xoo1047* | 3.74 | S | Vgr-related protein |
| *Xoo1068* | 3.13 | M | outer membrane protein Slp |
| *Xoo1075* | 3.85 | J | cleaves off formyl group from N-terminal methionine residues of newly synthesized proteins; binds iron(2+)\|peptide deformylase |
| *Xoo1076* | 2.87 |  | cellulase S |
| *Xoo1102* | 7.04 | IQ | catalyzes the first of the two reduction steps in the elongation cycle of fatty acid synthesis\|3-ketoacyl-ACP reductase |
| *Xoo1103* | 9.73 | C | Mg++/citrate complex transporter |
| *Xoo1104* | 16.96 | P | porin |
| *Xoo1117* | 3.29 | C | involved in the transport of C4-dicarboxylates across the membrane\|C4-dicarboxylate transporter DctA |
| *Xoo1178* | 2.13 | M | hypothetical protein |
| *Xoo1179* | 2.18 | S | hypothetical protein |
| *Xoo1184* | 2.22 | SI | long-chain acyl-CoA synthetase |
| *Xoo1235* | 2.55 | L | Avin3395\|transposase |
| *Xoo1241* | 3.72 | L | Tra8\|hypothetical protein |
| *Xoo1268* | 2.03 | K | AhyR/AsaR family transcriptional regulator |
| *Xoo1272* | 2.80 | L | transposase |
| *Xoo1273* | 4.15 | C | quinol oxidase subunit I |
| *Xoo1274* | 3.07 | C | quinol oxidase subunit II |
| *Xoo1288* | 2.23 | R | hypothetical protein |
| *Xoo1292* | 3.06 | S | hypothetical protein |
| *Xoo1293* | 3.30 | S | hypothetical protein |
| *Xoo1302* | 2.49 | M | nucleotidyl transferase |
| *Xoo1303* | 2.79 | R | hypothetical protein |
| *Xoo1304* | 2.81 | S | hypothetical protein |
| *Xoo1315* | 3.52 | L | transposase |
| *Xoo1317* | 2.95 | G | alpha-L-arabinofuranosidase |
| *Xoo1362* | 2.69 | P | cation:proton antiporter |
| *Xoo1376* | 6.03 | S | hypothetical protein |
| *Xoo1379* | 5.01 | T | HrpG protein |
| *Xoo1380* | 4.45 | T | HrpX protein |
| *Xoo1410* | 3.62 |  | hypothetical protein |
| *Xoo1429* | 6.33 | S | hypothetical protein |
| *Xoo1454* | 2.37 | S | hypothetical protein |
| *Xoo1477* | 2.06 | T | two-component system sensor protein |
| *Xoo1478* | 2.04 | L | transposase |
| *Xoo1502* | 2.30 | R | nitrilase |
| *Xoo1511* | 5.95 |  | peptidoglycan-associated outer membrane lipoprotein |
| *Xoo1512* | 2.47 |  | hypothetical protein |
| *Xoo1516* | 7.05 | S | hypothetical protein |
| *Xoo1518* | 4.91 |  | hypothetical protein |
| *Xoo1528* | 2.12 | R | short chain dehydrogenase |
| *Xoo1529* | 2.21 | L | hypothetical protein |
| *Xoo1532* | 2.68 | P | outer membrane hemin receptor |
| *Xoo1546* | 2.26 | T | hypothetical protein |
| *Xoo1655* | 5.63 | S | hypothetical protein |
| *Xoo1664* | 2.42 | R | hypothetical protein |
| *Xoo1665* | 2.62 | U | hypothetical protein |
| *Xoo1666* | 2.41 | U | hypothetical protein |
| *Xoo1667* | 2.39 | S | hypothetical protein |
| *Xoo1699* | 2.31 | R | I\|phage-related tail protein |
| *Xoo1712* | 2.35 | R | N\|phage-related major capsid protein |
| *Xoo1715* | 2.27 | R | Q\|phage-related capsid packaging protein |
| *Xoo1722* | 3.35 |  | hypothetical protein |
| *Xoo1734* | 2.07 | H | Required in the synthesis of PPQ, but its exact function is unknown\|pyrroloquinoline quinone biosynthesis protein PqqC |
| *Xoo1756* | 2.44 | R | hypothetical protein |
| *Xoo1803* | 4.74 | R | hypothetical protein |
| *Xoo1813* | 5.92 | M | hypothetical protein |
| *Xoo1818* | 2.22 | E | catalyzes the formation of cystathionine from L-cysteine and O-succinyl-L-homoserine\|cystathionine gamma-synthase |
| *Xoo1820* | 2.62 | E | homoserine dehydrogenase |
| *Xoo1833* | 2.04 | L | hypothetical protein |
| *Xoo1867* | 2.04 | G | hydrolyzes the terminal non-reducing N-acetyl-D-hexosamine residues in N-acetyl-beta-D-hexosaminides\|beta-hexosaminidase |
| *Xoo1887* | 2.50 | T | hypothetical protein |
| *Xoo1901* | 19.77 | G | MFS transporter |
| *Xoo1925* | 2.18 | S | hypothetical protein |
| *Xoo1951* | 2.17 | S | hypothetical protein |
| *Xoo1952* | 4.28 | I | PHB depolymerase |
| *Xoo1978* | 2.02 | NU | pili assembly chaperone |
| *Xoo1991* | 2.49 | S | hypothetical protein |
| *Xoo1994* | 19.16 | P | bacterioferritin |
| *Xoo1998* | 5.14 | K | MarR family transcriptional regulator |
| *Xoo1999* | 4.87 | M | outer membrane efflux protein |
| *Xoo2000* | 4.05 | V | multidrug resistance efflux pump |
| *Xoo2001* | 4.63 | P | multidrug resistance membrane translocase |
| *Xoo2005* | 2.47 |  | hypothetical protein |
| *Xoo2012* | 22.24 | L | Reut4275\|IS30 family transposase |
| *Xoo2035* | 2.27 | L | ISXo8 transposase |
| *Xoo2076* | 2.52 | I | acyl-CoA dehydrogenase |
| *Xoo2113* | 2.40 | L | IS1404 transposase |
| *Xoo2114* | 5.66 | S | TonB-like protein |
| *Xoo2132* | 2.90 | L | ISXo8 transposase |
| *Xoo2133* | 2.06 | R | hypothetical protein |
| *Xoo2145* | 2.62 | S | hypothetical protein |
| *Xoo2146* | 2.96 | L | IS1404 transposase |
| *Xoo2160* | 2.22 | S | hypothetical protein |
| *Xoo2198* | 4.98 | K | LysR family transcriptional regulator |
| *Xoo2199* | 3.08 | S | AGl1036\|hypothetical protein |
| *Xoo2228* | 2.28 | T | two-component system sensor protein |
| *Xoo2231* | 2.16 | G | Prop transport protein |
| *Xoo2280* | 2.10 | L | Avin3395\|transposase |
| *Xoo2281* | 3.11 | L | IS163, transposase |
| *Xoo2303* | 2.30 | R | hypothetical protein |
| *Xoo2323* | 2.39 | T | two-component system sensor protein |
| *Xoo2341* | 2.15 | E | peptidyl-dipeptidase |
| *Xoo2342* | 2.17 | O | extracellular protease |
| *Xoo2357* | 2.06 | R | alkaline phosphatase |
| *Xoo2376* | 2.42 | P | cation efflux system protein |
| *Xoo2395* | 2.10 | FK | histidine utilization repressor |
| *Xoo2396* | 2.05 | Q | catalyzing the hydrolysis of 4-imidazolone-5-propionate to N-formimidoyl-L-glutamate, the third step in the histidine degradation pathway\|imidazolonepropionase |
| *Xoo2397* | 2.80 | F | catalyzes the deimination of N-formimino-L-glutamate to ammonia and N-formyl-L-glutamate\|N-formimino-L-glutamate deiminase |
| *Xoo2398* | 2.42 | E | catalyzes the degradation of histidine to urocanate and ammmonia\|histidine ammonia-lyase |
| *Xoo2460* | 14.27 | P | polyphosphate-selective porin O |
| *Xoo2461* | 6.30 | P | phosphate binding protein |
| *Xoo2462* | 10.37 | P | phosphate ABC transporter substrate-binding protein |
| *Xoo2463* | 10.37 | P | phosphate ABC transporter permease |
| *Xoo2464* | 9.35 | P | phosphate ABC transporter permease |
| *Xoo2465* | 9.54 | P | ATP-binding protein; PstABCS is an ATP dependent phosphate uptake system which is responsible for inorganic phosphate uptake during phosphate starvation\|phosphate transporter ATP-binding protein |
| *Xoo2466* | 8.67 | P | phosphate regulon transcriptional regulator |
| *Xoo2467* | 7.11 | S | hypothetical protein |
| *Xoo2470* | 2.14 | L | transposase |
| *Xoo2489* | 2.29 | R | hypothetical protein |
| *Xoo2493* | 2.29 | L | ISXo8 transposase |
| *Xoo2522* | 4.54 | L | RSp1587\|hypothetical protein |
| *Xoo2539* | 2.05 | P | hypothetical protein |
| *Xoo2660* | 2.03 | C | cytochrome D ubiquinol oxidase subunit I |
| *Xoo2677* | 2.01 |  | hypothetical protein |
| *Xoo2713* | 3.77 | F | ribonuclease |
| *Xoo2714* | 2.66 | S | hypothetical protein |
| *Xoo2718* | 2.56 | S | hypothetical protein |
| *Xoo2726* | 2.00 | O | metalloprotease\|heat shock protein HtpX |
| *Xoo2738* | 3.58 | U | virulence protein |
| *Xoo2745* | 4.41 | L | RSp1628\|hypothetical protein |
| *Xoo2778* | 2.11 | M | outer membrane protein OprN |
| *Xoo2779* | 2.26 | P | transporter |
| *Xoo2780* | 2.11 | V | transporter |
| *Xoo2803* | 2.41 | K | TetR/AcrR family transcriptional regulator |
| *Xoo2804* | 2.58 | M | multidrug resistance protein |
| *Xoo2805* | 2.36 | P | multidrug efflux transporter |
| *Xoo2808* | 3.15 | L | transposase |
| *Xoo2809* | 2.63 | K | LacI family transcriptional regulator |
| *Xoo2810* | 2.73 | G | multiphosphoryl transfer protein |
| *Xoo2811* | 3.14 | G | 1-phosphofructokinase |
| *Xoo2812* | 3.17 | G | PTS system fructose-specific transporter subunit IIBC |
| *Xoo2813* | 3.84 | M | regulator of pathogenicity factors |
| *Xoo2861* | 3.56 | R | beta-ketoadipate enol-lactone hydrolase |
| *Xoo2865* | 2.13 | C | Catalyzes the conversion of citrate to isocitrate\|aconitate hydratase |
| *Xoo2930* | 2.77 | R | hypothetical protein |
| *Xoo2943* | 3.31 | R | ubiquinone biosynthesis protein |
| *Xoo2944* | 2.68 | R | hypothetical protein |
| *Xoo2968* | 2.32 |  | hypothetical protein |
| *Xoo3063* | 2.04 | L | IS1404 transposase |
| *Xoo3071* | 5.20 | S | hypothetical protein |
| *Xoo3102* | 2.64 | H | siroheme synthase |
| *Xoo3125* | 3.29 | O | peptidase |
| *Xoo3147* | 2.15 | T | sensor histidine kinase |
| *Xoo3150* | 3.46 | R | hypothetical protein |
| *Xoo3153* | 2.37 |  | hypothetical protein |
| *Xoo3164* | 2.07 | L | transposase |
| *Xoo3166* | 2.15 | S | GumN protein |
| *Xoo3172* | 2.44 | M | protein GumI |
| *Xoo3173* | 3.08 | M | protein GumH |
| *Xoo3174* | 3.33 | G | protein GumG |
| *Xoo3175* | 3.78 | G | protein GumF |
| *Xoo3176* | 4.84 | S | protein GumE |
| *Xoo3177* | 7.04 | M | protein GumD |
| *Xoo3178* | 5.42 | DM | protein GumC |
| *Xoo3179* | 5.60 | M | protein GumB |
| *Xoo3294* | 2.31 | R | hypothetical protein |
| *Xoo3317* | 4.35 | S | hypothetical protein |
| *Xoo3351* | 2.36 | L | transposase |
| *Xoo3389* | 4.05 |  | acetyltransferase |
| *Xoo3408* | 2.34 | H | siroheme synthase |
| *Xoo3438* | 2.26 |  | prolyl oligopeptidase |
| *Xoo3451* | 2.77 | T | serine/threonine kinase |
| *Xoo3490* | 2.13 | S | hypothetical protein |
| *Xoo3492* | 3.16 | S | hypothetical protein |
| *Xoo3504* | 2.14 | S | hypothetical protein |
| *Xoo3618* | 8.63 | G | xylanase |
| *Xoo3623* | 2.03 | EO | extracellular protease |
| *Xoo3658* | 2.06 | S | hypothetical protein |
| *Xoo3659* | 2.29 | T | two-component system regulatory protein |
| *Xoo3660* | 2.94 | L | ISXo8 transposase |
| *Xoo3662* | 3.01 | L | transposase |
| *Xoo3663* | 2.73 | S | hypothetical protein |
| *Xoo3664* | 2.66 | O | glutaredoxin |
| *Xoo3665* | 4.06 | O | hypothetical protein |
| *Xoo3666* | 15.80 | T | two-component system regulatory protein |
| *Xoo3667* | 10.48 | T | two-component system sensor protein |
| *Xoo3668* | 2.43 | P | catalyzes the reversible transfer of the terminal phosphate of ATP to form a long chain polyphosphate\|polyphosphate kinase |
| *Xoo3671* | 2.17 | R | hypothetical protein |
| *Xoo3698* | 2.75 | C | 2,4-dienoyl-CoA reductase |
| *Xoo3699* | 2.48 |  | hypothetical protein |
| *Xoo3714* | 2.50 | L | IS30 family transposase |
| *Xoo3764* | 2.52 | E | acyl-CoA thioesterase |
| *Xoo3792* | 3.76 | R | phosphoanhydride phosphohydrolase |
| *Xoo3793* | 20.62 | P | TonB-dependent receptor |
| *Xoo3796* | 2.30 | C | catalyzes the formation of oxaloacetate from phosphoenolpyruvate\|phosphoenolpyruvate carboxylase |
| *Xoo3797* | 2.46 | E | hypothetical protein |
| *Xoo3847* | 2.50 |  | hypothetical protein |
| *Xoo3872* | 2.03 |  | hypothetical protein |
| *Xoo3874* | 2.31 | L | ISXo8 transposase |
| *Xoo3878* | 2.58 | S | nodulin 21-like protein |
| *Xoo3909* | 2.02 | P | TonB-dependent receptor |
| *Xoo3928* | 2.17 | L | transposase |
| *Xoo3940* | 3.08 |  | hypothetical protein |
| *Xoo3941* | 3.25 | S | hypothetical protein |
| *Xoo3977* | 3.01 | R | hypothetical protein |
| *Xoo4006* | 2.43 | M | hypothetical protein |
| *Xoo4063* | 2.42 | M | hypothetical protein |
| *Xoo4076* | 2.04 | R | nuclease |
| *Xoo4087* | 2.25 | H | hypothetical protein |
| *Xoo4121* | 4.47 | L | transposase |
| *Xoo4149* | 19.16 | P | bacterioferritin |
| *Xoo4151* | 2.04 | L | hydrolyzes diadenosine polyphosphate\|dinucleoside polyphosphate hydrolase |
| *Xoo4166* | 2.02 | K | B-lactamase regulatory protein |
| *Xoo4199* | 3.04 | M | hypothetical protein |
| *Xoo4201* | 2.09 | T | two-component system sensor protein |
| *Xoo4208* | 2.75 | S | hypothetical protein |
| *Xoo4233* | 2.58 | L | IS1478 transposase |
| *Xoo4255* | 2.04 | S | avirulence/virulence protein |
| *Xoo4290* | 3.20 | L | ISxac1 transposase |
| *Xoo4293* | 2.19 |  | hypothetical protein |
| *Xoo4294* | 3.47 | C | NADH dehydrogenase |
| *Xoo4312* | 2.16 | I | hypothetical protein |
| *Xoo4349* | 2.81 | G | MFS transporter |
| *Xoo4358* | 2.28 | K | LysR family transcriptional regulator |
| *Xoo4409* | 3.18 | S | hypothetical protein |
| *Xoo4427* | 2.05 | G | catalyzes the interconversion of D-glucuronate to D-fructuronate or D-galacturonate to D-tagaturonate; functions in glucuronic and galacturonic metabolism\|glucuronate isomerase |
| *Xoo4428* | 3.73 | G | secreted xylanase |
| *Xoo4455* | 2.40 | K | MarR family transcriptional regulator |
| *Xoo4463* | 2.38 | S | Psyr4406\|hypothetical protein |
| *Xoo4464* | 2.53 |  | hypothetical protein |
| *Xoo4475* | 2.81 | Q | hemagglutinin |
| *Xoo4476* | 2.42 | S | microcystin dependent protein |
| *Xoo4486* | 2.05 | P | ammonium transporter |
| *Xoo4487* | 2.00 | E | nitrogen regulatory protein P-II |
| *Xoo4554* | 13.42 | P | hypothetical protein |
| *Xoo4555* | 30.76 | P | alkaline phosphatase |
| *Xoo4560* | 2.30 | L | transposase |
| *Xoo4709* | 3.03 |  | identified by sequence similarity; ORF located using Blastx/FrameD\|hypothetical protein |
| *Xoo4718* | 2.74 |  | hypothetical protein |
| *Xoo4720* | 2.95 | R | identified by sequence similarity; ORF located using Blastx/FrameD\|plasmid-related lipoprotein |
| *Xoo4725* | 2.80 |  | hypothetical protein |
| *Xoo4744* | 3.05 | SI | identified by sequence similarity; ORF located using Blastx/FrameD\|carbamoyl-phosphate synthase large subunit |
| *Xoo4766* | 2.98 |  | hypothetical protein |
| *Xoo4794* | 2.14 |  | hypothetical protein |
| *Xoo4799* | 2.70 | L | identified by sequence similarity; ORF located using Blastx/FrameD\|IS1404 transposase |
| *Xoo4823* | 2.48 |  | hypothetical protein |
| *Xoo4903* | 2.01 |  | hypothetical protein |
| *Xoo4922* | 2.23 | S | identified by sequence similarity; ORF located using Blastx/FrameD\|hypothetical protein |
| *Xoo4951* | 5.99 |  | hypothetical protein |
| *YXoop10* | 4.14 |  | hypothetical protein |
| **10 min downregulated** | | | |
| *Xoo0252* | 0.32 | K | LacI family transcriptional regulator |
| *Xoo0283* | 0.47 | G | cellulase |
| *Xoo0297* | 0.04 | L | transposase |
| *Xoo0301* | 0.45 | L | transposase |
| *Xoo0339* | 0.48 | G | fucose permease |
| *Xoo0358* | 0.40 | L | transposase |
| *Xoo0392* | 0.19 | S | Pass1-like protein |
| *Xoo0393* | 0.20 | S | protein SapC protein |
| *Xoo0394* | 0.06 | P | TonB-dependent receptor |
| *Xoo0433* | 0.43 | R | hypothetical protein |
| *Xoo0556* | 0.35 | I | an AccC homodimer forms the biotin carboxylase subunit of the acetyl CoA carboxylase, an enzyme that catalyzes the formation of malonyl-CoA, which in turn controls the rate of fatty acid metabolism\|acetyl-CoA carboxylase biotin carboxylase subunit |
| *Xoo0557* | 0.39 | S | crystal structure of protein from Xanthomonas shows pentameric toroidal structure; physiological function is unknown\|hypothetical protein |
| *Xoo0558* | 0.39 | I | composes the biotin carboxyl carrier protein subunit of the acetyl-CoA carboxylase complex, the enzyme that catalyzes the carboxylation of acetyl-CoA to malonyl-CoA, which in turn controls the rate of fatty acid metabolism\|acetyl-CoA carboxylase biotin carboxyl carrier protein subunit |
| *Xoo0657* | 0.32 |  | hypothetical protein |
| *Xoo0659* | 0.30 | E | amino acid transporter |
| *Xoo0748* | 0.41 | S | hypothetical protein |
| *Xoo0749* | 0.41 | L | transposase |
| *Xoo0761* | 0.33 | I | beta-ketoacyl- synthase I |
| *Xoo0762* | 0.35 | I | catalyzes the dehydration of (3R)-3-hydroxydecanoyl-ACP to 2,3-decenoyl-ACP or 3,4-decenoyl-ACP\|3-hydroxydecanoyl-ACP dehydratase |
| *Xoo0880* | 0.43 | I | ACP S-malonyltransferase |
| *Xoo0881* | 0.35 | IQ | Catalyzes the first of the two reduction steps in the elongation cycle of fatty acid synthesis\|3-ketoacyl-ACP reductase |
| *Xoo0892* | 0.17 | G | catalyzes the formation of pyruvate and succinate from 2-methylisocitrate\|2-methylisocitrate lyase |
| *Xoo0893* | 0.35 | C | catalyzes the synthesis of 2-methylcitrate from propionyl-CoA and oxaloacetate; also catalyzes the condensation of oxaloacetate with acetyl-CoA but with a lower specificity\|methylcitrate synthase |
| *Xoo0894* | 0.40 | C | catalyzes the conversion of citrate to isocitrate\|aconitate hydratase |
| *Xoo0899* | 0.50 |  | hypothetical protein |
| *Xoo0987* | 0.38 | L | IS1404 transposase |
| *Xoo1015* | 0.41 | C | glycerophosphodiester phosphodiesterase |
| *Xoo1100* | 0.46 | G | sugar transporter |
| *Xoo1116* | 0.44 | P | polyphosphate-selective porin O |
| *Xoo1188* | 0.46 | T | response regulator |
| *Xoo1189* | 0.47 | T | Avin0574\|Signal transduction histidine kinase |
| *Xoo1217* | 0.32 |  | hypothetical protein |
| *Xoo1261* | 0.37 | S | hypothetical protein |
| *Xoo1262* | 0.37 | K | hypothetical protein |
| *Xoo1263* | 0.31 | S | tryptophan halogenase |
| *Xoo1265* | 0.48 |  | hypothetical protein |
| *Xoo1266* | 0.46 | S | endoproteinase ArgC |
| *Xoo1336* | 0.32 | L | GIY-YIG nuclease superfamily protein |
| *Xoo1354* | 0.12 | E | diaminopimelate decarboxylase |
| *Xoo1355* | 0.08 | Q | iron transporter |
| *Xoo1356* | 0.07 | P | transporter |
| *Xoo1357* | 0.04 | Q | hypothetical protein |
| *Xoo1358* | 0.04 | I | hypothetical protein |
| *Xoo1359* | 0.02 | P | citrate-dependent iron transporter |
| *Xoo1360* | 0.02 | G | 4-hydroxy-2-oxovalerate aldolase |
| *Xoo1422* | 0.43 | S | hypothetical protein |
| *Xoo1467* | 0.37 | T | response regulator |
| *Xoo1468* | 0.39 | S | chemotaxis protein |
| *Xoo1469* | 0.38 | NT | chemotaxis protein |
| *Xoo1470* | 0.40 | T | chemotaxis histidine protein kinase |
| *Xoo1471* | 0.38 | S | hypothetical protein |
| *Xoo1530* | 0.21 | P | TonB-dependent receptor |
| *Xoo1604* | 0.50 | P | ferric enterobactin receptor |
| *Xoo1769* | 0.23 | G | beta-galactosidase |
| *Xoo1770* | 0.22 | G | hypothetical protein |
| *Xoo1771* | 0.16 | S | hypothetical protein |
| *Xoo1821* | 0.41 | E | L-serine dehydratase |
| *Xoo1842* | 0.17 | C | methylmalonate-semialdehyde dehydrogenase |
| *Xoo1843* | 0.19 | I | acyl-CoA dehydrogenase |
| *Xoo1844* | 0.15 | S | Catalyzes the reversible hydration of unsaturated fatty acyl-CoA to beta-hydroxyacyl-CoA\|enoyl-CoA hydratase |
| *Xoo1845* | 0.24 | I | enoyl-CoA hydratase |
| *Xoo1846* | 0.20 | I | 3-hydroxyisobutyrate dehydrogenase |
| *Xoo1902* | 0.42 | S | hypothetical protein |
| *Xoo1992* | 0.06 | P | iron permease |
| *Xoo1993* | 0.24 | R | penicillin acylase II |
| *Xoo2069* | 0.28 | K | transcriptional regulator |
| *Xoo2070* | 0.24 | G | glucose-galactose transporter |
| *Xoo2071* | 0.30 | G | fructokinase |
| *Xoo2072* | 0.35 | G | hypothetical protein |
| *Xoo2107* | 0.32 | L | transposase |
| *Xoo2152* | 0.42 |  | hypothetical protein |
| *Xoo2158* | 0.18 | L | transposase |
| *Xoo2167* | 0.40 | L | transposase |
| *Xoo2203* | 0.36 |  | hypothetical protein |
| *Xoo2204* | 0.27 | S | methyl-accepting chemotaxis protein |
| *Xoo2217* | 0.40 |  | transposase |
| *Xoo2338* | 0.45 | C | ferredoxin-NADP reductase |
| *Xoo2348* | 0.41 |  | hypothetical protein |
| *Xoo2351* | 0.11 | E | hypothetical protein |
| *Xoo2352* | 0.10 | G | beta-glucosidase |
| *Xoo2355* | 0.46 | R | sodium/glucose cotransport protein |
| *Xoo2356* | 0.49 | G | glucan 1,4-beta-glucosidase |
| *Xoo2385* | 0.49 | L | IS1478 transposase |
| *Xoo2523* | 0.41 | L | transposase |
| *Xoo2550* | 0.47 | M | hypothetical protein |
| *Xoo2558* | 0.36 | S | chemotaxis protein |
| *Xoo2559* | 0.34 |  | hypothetical protein |
| *Xoo2561* | 0.41 | T | hypothetical protein |
| *Xoo2572* | 0.49 | N | the hook connects flagellar basal body to the flagellar filament\|flagellar hook protein FlgE |
| *Xoo2581* | 0.46 | N | structural flagella protein; Vibrio contains multiple flagellin genes usually localized into two region on the chromosome, flaAC and flaCEDB in V. cholerae, flaFBA and flaCDE in V. parahemolyticus; FlaA is sigma 54 dependent and essential for motility in V.cholerae but not in V. parahaemolyticus\|flagellin |
| *Xoo2584* | 0.49 |  | hypothetical protein |
| *Xoo2591* | 0.34 | S | acyl carrier protein |
| *Xoo2592* | 0.38 | I | 3-oxoacyl-ACP synthase |
| *Xoo2593* | 0.44 | R | 3-oxoacyl-ACP reductase |
| *Xoo2594* | 0.46 | IQ | dehydrogenase |
| *Xoo2596* | 0.43 | P | ring hydroxylating dioxygenase subunit alpha |
| *Xoo2597* | 0.45 | S | hypothetical protein |
| *Xoo2598* | 0.34 | R | hypothetical protein |
| *Xoo2686* | 0.41 | L | transposase |
| *Xoo2760* | 0.44 | C | ubiquinol cytochrome C oxidoreductase, cytochrome C1 subunit |
| *Xoo2761* | 0.36 | C | ubiquinol cytochrome C oxidoreductase, cytochrome B subunit |
| *Xoo2762* | 0.47 | C | ubiquinol cytochrome C oxidoreductase, iron-sulfur subunit |
| *Xoo2787* | 0.38 | T | transcriptional regulator |
| *Xoo2826* | 0.36 | P | TonB-dependent receptor |
| *Xoo2835* | 0.46 | T | chemotaxis response regulator |
| *Xoo2836* | 0.49 | T | chemotaxis protein |
| *Xoo2842* | 0.23 | NT | chemotaxis protein |
| *Xoo2844* | 0.45 | NT | chemotaxis protein |
| *Xoo2849* | 0.30 | M | hypothetical protein |
| *Xoo2850* | 0.25 | NT | chemotaxis protein |
| *Xoo2857* | 0.37 | NT | chemotaxis protein methyltransferase |
| *Xoo2896* | 0.11 |  | hypothetical protein |
| *Xoo2897* | 0.22 |  | hypothetical protein |
| *Xoo2898* | 0.14 |  | hypothetical protein |
| *Xoo2899* | 0.16 | P | ferrous iron transport protein B |
| *Xoo2900* | 0.16 | P | ferrous iron transport protein |
| *Xoo2912* | 0.31 | S | hypothetical protein |
| *Xoo2913* | 0.27 | S | hypothetical protein |
| *Xoo2975* | 0.49 | S | hypothetical protein |
| *Xoo3128* | 0.27 | G | hypothetical protein |
| *Xoo3129* | 0.29 | G | hypothetical protein |
| *Xoo3137* | 0.49 | E | dihydrodipicolinate synthetase |
| *Xoo3199* | 0.43 | U | protein PilX |
| *Xoo3200* | 0.46 | U | hypothetical protein |
| *Xoo3201* | 0.45 | U | pre-pilin leader sequence |
| *Xoo3202* | 0.48 | U | pre-pilin like leader sequence |
| *Xoo3246* | 0.47 | T | phosphodiesterase |
| *Xoo3247* | 0.46 | S | hypothetical protein |
| *Xoo3272* | 0.50 | J | catalyzes the methylthiolation of an aspartic acid residue of the S12 protein of the 30S ribosomal subunit\|30S ribosomal protein S12 methylthiotransferase |
| *Xoo3347* | 0.37 | L | transposase |
| *Xoo3370* | 0.18 | IN | lipase/esterase |
| *Xoo3378* | 0.50 | L | IS1478 transposase |
| *Xoo3433* | 0.50 | E | alanyl dipeptidyl peptidase |
| *Xoo3435* | 0.39 | S | oligopeptide transporter |
| *Xoo3445* | 0.15 | L | transposase |
| *Xoo3661* | 0.31 | L | transposase |
| *Xoo3684* | 0.39 | C | converts threonine and NAD to 1,2-amino-3-oxobutanoate and NADH; functions in threonine catabolism\|L-threonine 3-dehydrogenase |
| *Xoo3780* | 0.03 |  | outer membrane hemin receptor |
| *Xoo3781* | 0.01 | S | hypothetical protein |
| *Xoo3794* | 0.42 | L | ISXo8 transposase |
| *Xoo3879* | 0.31 | L | transposase |
| *Xoo3892* | 0.46 | L | ISxac1 transposase |
| *Xoo4079* | 0.44 | L | transposase |
| *Xoo4090* | 0.31 | L | transposase |
| *Xoo4136* | 0.31 | S | hypothetical protein |
| *Xoo4137* | 0.31 | C | cytochrome C oxidase subunit III |
| *Xoo4138* | 0.31 | O | involved in the insertion of copper into subunit I of cytochrome C oxidase\|cytochrome C oxidase assembly protein |
| *Xoo4139* | 0.22 |  | hypothetical protein |
| *Xoo4140* | 0.34 | C | cytochrome C oxidase subunit I |
| *Xoo4141* | 0.45 | C | cytochrome C oxidase subunit II |
| *Xoo4150* | 0.05 | P | bacterioferritin-associated ferredoxin |
| *Xoo4219* | 0.45 | K | MarR family transcriptional regulator |
| *Xoo4377* | 0.08 | I | biotin carboxylase |
| *Xoo4378* | 0.05 | I | acyl-CoA carboxyltransferase subunit beta |
| *Xoo4379* | 0.04 | I | acyl-CoA dehydrogenase |
| *Xoo4380* | 0.29 | K | AcrR family transcriptional regulator |
| *Xoo4389* | 0.20 | S | hypothetical protein |
| *Xoo4396* | 0.30 | P | TonB-dependent receptor |
| *Xoo4425* | 0.44 | E | bleomycin resistance protein |
| *Xoo4431* | 0.28 | P | TonB-dependent receptor |
| *Xoo4446* | 0.18 | I | hypothetical protein |
| *Xoo4447* | 0.21 | C | oxidoreductase |
| *Xoo4503* | 0.38 |  | hypothetical protein |
| *Xoo4535* | 0.41 | M | RTS beta protein |
| *Xoo4536* | 0.32 | Q | 2-hydroxyhepta-2,4-diene-1, 7-dioate isomerase |
| *Xoo4537* | 0.32 | IQ | oxidoreductase |
| *Xoo4538* | 0.28 | R | hypothetical protein |
| *Xoo4539* | 0.26 | C | oxidoreductase |
| *Xoo4545* | 0.19 | S | hypothetical protein |
| *Xoo4546* | 0.19 | S | hypothetical protein |
| *Xoo4604* | 0.49 | G | dicarboxylate transport protein |
| *Xoo4617* | 0.47 | S | hypothetical protein |
| *Xoo4761* | 0.20 |  | hypothetical protein |
| **15 min upregulated** | | | |
| *Xoo0023* | 2.47 | S | hypothetical protein |
| *Xoo0026* | 2.93 | RK | transcriptional regulator |
| *Xoo0038* | 2.55 | G | proline/betaine transporter |
| *Xoo0064* | 2.37 | L | transposase |
| *Xoo0075* | 2.07 | S | protein HpaB |
| *Xoo0078* | 2.30 | S | protein HrpD5 |
| *Xoo0096* | 3.65 | M | protein Hpa2 |
| *Xoo0097* | 3.51 | S | hypothetical protein |
| *Xoo0099* | 2.42 | L | transposase |
| *Xoo0106* | 3.41 | L | transposase |
| *Xoo0121* | 2.15 | L | ISXo8 transposase |
| *Xoo0134* | 2.40 | L | IS1479 transposase |
| *Xoo0135* | 2.91 | C | dihydrolipoamide acyltransferase |
| *Xoo0137* | 2.68 | C | dihydrolipoamide acyltransferase |
| *Xoo0163* | 3.62 | S | hypothetical protein |
| *Xoo0169* | 2.20 | K | xylose repressor-like protein |
| *Xoo0172* | 7.19 | S | hypothetical protein |
| *Xoo0177* | 3.13 | E | catalyzes the formation of glutamate from glutamine and alpha-ketoglutarate\|glutamate synthase subunit alpha |
| *Xoo0178* | 2.73 | E | glutamate synthase is composed of subunits alpha and beta; beta subunit is a flavin adenine dinucleotide-NADPH dependent oxidoreductase; provides electrons to the alpha subunit, which binds L-glutamine and 2-oxoglutarate and forms L-glutamate\|glutamate synthase subunit beta |
| *Xoo0222* | 4.33 | S | Vgr-related protein |
| *Xoo0223* | 2.92 | S | hypothetical protein |
| *Xoo0281* | 4.02 | G | cellulase |
| *Xoo0298* | 2.84 | L | ISXo8 transposase |
| *Xoo0307* | 2.54 | Q | toluene tolerance protein |
| *Xoo0308* | 2.11 | Q | toluene tolerance protein |
| *Xoo0309* | 2.32 | Q | toluene tolerance protein |
| *Xoo0310* | 2.19 | Q | toluene tolerance protein |
| *Xoo0316* | 3.41 | L | transposase |
| *Xoo0329* | 2.30 | S | polyvinylalcohol dehydrogenase |
| *Xoo0337* | 3.89 | S | hypothetical protein |
| *Xoo0405* | 2.38 | K | LysR family transcriptional regulator |
| *Xoo0422* | 2.78 |  | hypothetical protein |
| *Xoo0423* | 2.68 | T | two-component system regulatory protein |
| *Xoo0425* | 2.21 | S | hypothetical protein |
| *Xoo0426* | 2.04 | S | hypothetical protein |
| *Xoo0433* | 2.30 | R | hypothetical protein |
| *Xoo0543* | 2.08 | I | hypothetical protein |
| *Xoo0589* | 2.27 | M | hypothetical protein |
| *Xoo0609* | 2.02 | L | phage-related integrase |
| *Xoo0637* | 2.01 | L | transposase |
| *Xoo0650* | 2.54 | T | hypothetical protein |
| *Xoo0662* | 2.31 | O | metallopeptidase |
| *Xoo0664* | 2.29 | C | oxidoreductase |
| *Xoo0669* | 2.06 | R | hypothetical protein |
| *Xoo0675* | 3.69 | P | Na+:H+ antiporter |
| *Xoo0716* | 2.18 |  | hypothetical protein |
| *Xoo0717* | 7.73 | M | hypothetical protein |
| *Xoo0719* | 2.00 |  | hypothetical protein |
| *Xoo0768* | 13.53 |  | hypothetical protein |
| *Xoo0770* | 4.50 | L | ATP-dependent RNA helicase |
| *Xoo0786* | 2.13 | L | IS1478 transposase |
| *Xoo0859* | 2.15 | K | transcriptional regulator |
| *Xoo0899* | 2.52 |  | hypothetical protein |
| *Xoo0900* | 2.16 | S | hypothetical protein |
| *Xoo0901* | 80.52 | P | TonB-dependent receptor |
| *Xoo0902* | 47.90 | C | glycerophosphodiester phosphodiesterase |
| *Xoo0903* | 20.87 | V | hypothetical protein |
| *Xoo0904* | 4.02 | L | ISXo8 transposase |
| *Xoo0907* | 2.11 | P | bacterioferritin |
| *Xoo0929* | 2.98 | K | transcriptional regulator |
| *Xoo0930* | 2.46 | T | hypothetical protein |
| *Xoo0931* | 3.18 | T | hypothetical protein |
| *Xoo0948* | 2.24 | K | hypothetical protein |
| *Xoo0949* | 3.13 | K | transcriptional regulator |
| *Xoo0958* | 2.08 |  | hypothetical protein |
| *Xoo1004* | 2.18 | M | hypothetical protein |
| *Xoo1006* | 2.46 | L | transposase |
| *Xoo1011* | 20.31 | L | transposase |
| *Xoo1014* | 5.16 | M | hypothetical protein |
| *Xoo1044* | 7.22 | L | transposase |
| *Xoo1047* | 5.07 | S | Vgr-related protein |
| *Xoo1048* | 2.70 | S | hypothetical protein |
| *Xoo1054* | 2.06 | L | integrase |
| *Xoo1068* | 2.59 | M | outer membrane protein Slp |
| *Xoo1075* | 3.60 | J | cleaves off formyl group from N-terminal methionine residues of newly synthesized proteins; binds iron(2+)\|peptide deformylase |
| *Xoo1102* | 7.91 | IQ | catalyzes the first of the two reduction steps in the elongation cycle of fatty acid synthesis\|3-ketoacyl-ACP reductase |
| *Xoo1103* | 9.85 | C | Mg++/citrate complex transporter |
| *Xoo1104* | 18.01 | P | porin |
| *Xoo1117* | 2.25 | C | involved in the transport of C4-dicarboxylates across the membrane\|C4-dicarboxylate transporter DctA |
| *Xoo1136* | 3.41 | L | transposase |
| *Xoo1155* | 2.03 | S | hypothetical protein |
| *Xoo1178* | 2.19 | M | hypothetical protein |
| *Xoo1180* | 2.36 | P | TonB-dependent receptor |
| *Xoo1184* | 2.91 | SI | long-chain acyl-CoA synthetase |
| *Xoo1186* | 2.26 | R | Avin0576\|short chain dehydrogenase |
| *Xoo1204* | 2.46 | L | transposase |
| *Xoo1259* | 2.06 | E | peptidase |
| *Xoo1269* | 2.37 | R | proline imino-peptidase |
| *Xoo1272* | 2.19 | L | transposase |
| *Xoo1273* | 3.77 | C | quinol oxidase subunit I |
| *Xoo1274* | 2.53 | C | quinol oxidase subunit II |
| *Xoo1292* | 2.16 | S | hypothetical protein |
| *Xoo1293* | 2.33 | S | hypothetical protein |
| *Xoo1303* | 2.18 | R | hypothetical protein |
| *Xoo1304* | 2.22 | S | hypothetical protein |
| *Xoo1309* | 2.77 | L | DNA transport competence protein |
| *Xoo1315* | 2.65 | L | transposase |
| *Xoo1317* | 2.30 | G | alpha-L-arabinofuranosidase |
| *Xoo1362* | 2.32 | P | cation:proton antiporter |
| *Xoo1376* | 5.39 | S | hypothetical protein |
| *Xoo1379* | 4.44 | T | HrpG protein |
| *Xoo1380* | 6.49 | T | HrpX protein |
| *Xoo1392* | 2.16 | L | IS1404 transposase |
| *Xoo1410* | 2.69 |  | hypothetical protein |
| *Xoo1412* | 2.12 |  | hypothetical protein |
| *Xoo1429* | 3.46 | S | hypothetical protein |
| *Xoo1449* | 2.37 | O | hypothetical protein |
| *Xoo1478* | 2.12 | L | transposase |
| *Xoo1502* | 3.13 | R | nitrilase |
| *Xoo1511* | 4.23 |  | peptidoglycan-associated outer membrane lipoprotein |
| *Xoo1516* | 4.34 | S | hypothetical protein |
| *Xoo1518* | 3.77 |  | hypothetical protein |
| *Xoo1528* | 2.03 | R | short chain dehydrogenase |
| *Xoo1655* | 3.36 | S | hypothetical protein |
| *Xoo1665* | 2.19 | U | hypothetical protein |
| *Xoo1677* | 2.46 | L | transposase |
| *Xoo1688* | 2.37 | K | Z2969\|hypothetical protein |
| *Xoo1703* | 2.11 | R | R\|phage-related tail protein |
| *Xoo1715* | 2.08 | R | Q\|phage-related capsid packaging protein |
| *Xoo1722* | 2.64 |  | hypothetical protein |
| *Xoo1796* | 2.46 | S | hypothetical protein |
| *Xoo1803* | 3.88 | R | hypothetical protein |
| *Xoo1813* | 3.21 | M | hypothetical protein |
| *Xoo1822* | 2.03 | O | hypothetical protein |
| *Xoo1827* | 2.07 | T | histidine kinase-response regulator hybrid protein |
| *Xoo1833* | 2.85 | L | hypothetical protein |
| *Xoo1834* | 2.87 | L | ISxac3 transposase |
| *Xoo1901* | 24.16 | G | MFS transporter |
| *Xoo1951* | 2.16 | S | hypothetical protein |
| *Xoo1952* | 3.82 | I | PHB depolymerase |
| *Xoo1991* | 2.26 | S | hypothetical protein |
| *Xoo1994* | 18.17 | P | bacterioferritin |
| *Xoo1998* | 4.64 | K | MarR family transcriptional regulator |
| *Xoo1999* | 4.53 | M | outer membrane efflux protein |
| *Xoo2000* | 3.90 | V | multidrug resistance efflux pump |
| *Xoo2001* | 4.61 | P | multidrug resistance membrane translocase |
| *Xoo2012* | 15.79 | L | Reut4275\|IS30 family transposase |
| *Xoo2076* | 2.48 | I | acyl-CoA dehydrogenase |
| *Xoo2082* | 2.18 | L | hypothetical protein |
| *Xoo2111* | 2.05 | L | ISXo8 transposase |
| *Xoo2114* | 3.49 | S | TonB-like protein |
| *Xoo2122* | 2.19 | L | transposase |
| *Xoo2132* | 4.17 | L | ISXo8 transposase |
| *Xoo2133* | 3.12 | R | hypothetical protein |
| *Xoo2145* | 2.15 | S | hypothetical protein |
| *Xoo2198* | 3.44 | K | LysR family transcriptional regulator |
| *Xoo2199* | 2.44 | S | AGl1036\|hypothetical protein |
| *Xoo2228* | 2.32 | T | two-component system sensor protein |
| *Xoo2231* | 2.46 | G | Prop transport protein |
| *Xoo2280* | 2.12 | L | Avin3395\|transposase |
| *Xoo2323* | 2.11 | T | two-component system sensor protein |
| *Xoo2341* | 2.02 | E | peptidyl-dipeptidase |
| *Xoo2375* | 2.15 | O | serine protease |
| *Xoo2376* | 2.34 | P | cation efflux system protein |
| *Xoo2396* | 2.10 | Q | catalyzing the hydrolysis of 4-imidazolone-5-propionate to N-formimidoyl-L-glutamate, the third step in the histidine degradation pathway\|imidazolonepropionase |
| *Xoo2397* | 2.20 | F | catalyzes the deimination of N-formimino-L-glutamate to ammonia and N-formyl-L-glutamate\|N-formimino-L-glutamate deiminase |
| *Xoo2398* | 2.19 | E | catalyzes the degradation of histidine to urocanate and ammmonia\|histidine ammonia-lyase |
| *Xoo2457* | 2.01 | P | carbonic anhydrase |
| *Xoo2460* | 10.18 | P | polyphosphate-selective porin O |
| *Xoo2461* | 4.62 | P | phosphate binding protein |
| *Xoo2462* | 7.55 | P | phosphate ABC transporter substrate-binding protein |
| *Xoo2463* | 7.10 | P | phosphate ABC transporter permease |
| *Xoo2464* | 6.16 | P | phosphate ABC transporter permease |
| *Xoo2465* | 8.30 | P | ATP-binding protein; PstABCS is an ATP dependent phosphate uptake system which is responsible for inorganic phosphate uptake during phosphate starvation\|phosphate transporter ATP-binding protein |
| *Xoo2466* | 6.26 | P | phosphate regulon transcriptional regulator |
| *Xoo2467* | 4.94 | S | hypothetical protein |
| *Xoo2470* | 2.14 | L | transposase |
| *Xoo2493* | 3.16 | L | ISXo8 transposase |
| *Xoo2495* | 2.14 | L | transposase |
| *Xoo2502* | 2.46 | L | transposase |
| *Xoo2520* | 2.06 | L | ISxac1 transposase |
| *Xoo2600* | 2.52 | N | flagellar protein |
| *Xoo2607* | 2.17 | N | flagellar protein |
| *Xoo2612* | 2.24 | N | flagellar biosynthesis |
| *Xoo2625* | 2.07 | L | IS1478 transposase |
| *Xoo2627* | 3.83 | L | Reut4275\|IS30 family transposase |
| *Xoo2647* | 2.02 |  | hypothetical protein |
| *Xoo2713* | 2.48 | F | ribonuclease |
| *Xoo2714* | 2.35 | S | hypothetical protein |
| *Xoo2738* | 2.11 | U | virulence protein |
| *Xoo2745* | 4.77 | L | RSp1628\|hypothetical protein |
| *Xoo2750* | 2.06 | L | responsible for recognizing base lesions in the genome and initiating base excision DNA repair\|3-methyladenine DNA glycosylase |
| *Xoo2778* | 2.20 | M | outer membrane protein OprN |
| *Xoo2779* | 2.18 | P | transporter |
| *Xoo2780* | 2.14 | V | transporter |
| *Xoo2799* | 2.12 | P | drug resistance translocase |
| *Xoo2803* | 2.75 | K | TetR/AcrR family transcriptional regulator |
| *Xoo2804* | 2.25 | M | multidrug resistance protein |
| *Xoo2805* | 2.29 | P | multidrug efflux transporter |
| *Xoo2808* | 2.15 | L | transposase |
| *Xoo2809* | 2.37 | K | LacI family transcriptional regulator |
| *Xoo2861* | 2.93 | R | beta-ketoadipate enol-lactone hydrolase |
| *Xoo2893* | 3.41 | L | transposase |
| *Xoo2933* | 2.36 | R | hypothetical protein |
| *Xoo2943* | 2.84 | R | ubiquinone biosynthesis protein |
| *Xoo2971* | 2.10 | L | transposase |
| *Xoo3046* | 2.53 | S | hypothetical protein |
| *Xoo3071* | 3.50 | S | hypothetical protein |
| *Xoo3101* | 2.31 | T | histidine kinase/response regulator hybrid protein |
| *Xoo3102* | 2.33 | H | siroheme synthase |
| *Xoo3125* | 2.69 | O | peptidase |
| *Xoo3147* | 2.01 | T | sensor histidine kinase |
| *Xoo3157* | 3.10 | L | transposase |
| *Xoo3158* | 2.31 | L | transposase |
| *Xoo3168* | 2.17 | M | protein GumM |
| *Xoo3169* | 2.21 | S | GumL protein |
| *Xoo3171* | 2.42 | M | protein GumJ |
| *Xoo3172* | 3.09 | M | protein GumI |
| *Xoo3173* | 3.58 | M | protein GumH |
| *Xoo3174* | 3.36 | G | protein GumG |
| *Xoo3175* | 3.56 | G | protein GumF |
| *Xoo3176* | 4.57 | S | protein GumE |
| *Xoo3177* | 5.67 | M | protein GumD |
| *Xoo3178* | 5.00 | DM | protein GumC |
| *Xoo3179* | 4.97 | M | protein GumB |
| *Xoo3204* | 2.06 | S | Oar protein |
| *Xoo3243* | 3.41 | L | transposase |
| *Xoo3317* | 2.68 | S | hypothetical protein |
| *Xoo3337* | 2.46 | L | transposase |
| *Xoo3351* | 2.80 | L | transposase |
| *Xoo3363* | 2.24 | R | virulence regulator |
| *Xoo3364* | 2.08 |  | hypothetical protein |
| *Xoo3389* | 4.66 |  | acetyltransferase |
| *Xoo3451* | 2.18 | T | serine/threonine kinase |
| *Xoo3554* | 3.41 | L | transposase |
| *Xoo3618* | 7.68 | G | xylanase |
| *Xoo3646* | 2.58 | O | alkyl hydroperoxide reductase |
| *Xoo3647* | 2.12 | K | oxidative stress transcriptional regulator |
| *Xoo3658* | 2.44 | S | hypothetical protein |
| *Xoo3659* | 2.12 | T | two-component system regulatory protein |
| *Xoo3662* | 2.30 | L | transposase |
| *Xoo3663* | 2.15 | S | hypothetical protein |
| *Xoo3664* | 2.23 | O | glutaredoxin |
| *Xoo3665* | 2.71 | O | hypothetical protein |
| *Xoo3666* | 9.66 | T | two-component system regulatory protein |
| *Xoo3667* | 7.29 | T | two-component system sensor protein |
| *Xoo3668* | 2.08 | P | catalyzes the reversible transfer of the terminal phosphate of ATP to form a long chain polyphosphate\|polyphosphate kinase |
| *Xoo3698* | 2.35 | C | 2,4-dienoyl-CoA reductase |
| *Xoo3699* | 2.49 |  | hypothetical protein |
| *Xoo3708* | 3.10 | L | transposase |
| *Xoo3716* | 2.46 | L | transposase |
| *Xoo3723* | 2.03 | G | gluconolactonase |
| *Xoo3764* | 2.10 | E | acyl-CoA thioesterase |
| *Xoo3792* | 3.52 | R | phosphoanhydride phosphohydrolase |
| *Xoo3793* | 17.08 | P | TonB-dependent receptor |
| *Xoo3796* | 2.31 | C | catalyzes the formation of oxaloacetate from phosphoenolpyruvate\|phosphoenolpyruvate carboxylase |
| *Xoo3797* | 2.29 | E | hypothetical protein |
| *Xoo3809* | 2.01 | S | hypothetical protein |
| *Xoo3811* | 2.56 |  | hypothetical protein |
| *Xoo3836* | 2.02 | S | hypothetical protein |
| *Xoo3909* | 2.40 | P | TonB-dependent receptor |
| *Xoo3928* | 2.63 | L | transposase |
| *Xoo3939* | 2.14 | G | similar to full-length Gnd, these proteins seems to have a truncated C-terminal 6PGD domainin; in Methylobacillus flagellatus this gene is essential for NAD+-dependent oxidation of 6-phosphogluconate\|6-phosphogluconate dehydrogenase |
| *Xoo3941* | 2.49 | S | hypothetical protein |
| *Xoo3977* | 2.34 | R | hypothetical protein |
| *Xoo4001* | 2.51 | F | hypothetical protein |
| *Xoo4006* | 2.19 | M | hypothetical protein |
| *Xoo4026* | 2.46 | L | transposase |
| *Xoo4045* | 3.55 | S | hypothetical protein |
| *Xoo4103* | 2.19 | L | transposase |
| *Xoo4104* | 2.01 | L | transposase |
| *Xoo4107* | 2.46 | L | transposase |
| *Xoo4119* | 2.46 | L | transposase |
| *Xoo4121* | 5.36 | L | transposase |
| *Xoo4129* | 2.22 | L | ISXo8 transposase |
| *Xoo4143* | 3.41 | L | transposase |
| *Xoo4149* | 19.97 | P | bacterioferritin |
| *Xoo4151* | 2.12 | L | hydrolyzes diadenosine polyphosphate\|dinucleoside polyphosphate hydrolase |
| *Xoo4166* | 2.51 | K | B-lactamase regulatory protein |
| *Xoo4199* | 3.59 | M | hypothetical protein |
| *Xoo4208* | 2.67 | S | hypothetical protein |
| *Xoo4254* | 3.16 | L | transposase |
| *Xoo4255* | 2.03 | S | avirulence/virulence protein |
| *Xoo4294* | 3.17 | C | NADH dehydrogenase |
| *Xoo4348* | 2.16 | K | MerR family transcriptional regulator |
| *Xoo4349* | 2.07 | G | MFS transporter |
| *Xoo4358* | 2.87 | K | LysR family transcriptional regulator |
| *Xoo4385* | 3.10 | S | hypothetical protein |
| *Xoo4402* | 2.81 | L | transposase |
| *Xoo4409* | 2.66 | S | hypothetical protein |
| *Xoo4428* | 4.17 | G | secreted xylanase |
| *Xoo4455* | 3.49 | K | MarR family transcriptional regulator |
| *Xoo4463* | 2.37 | S | Psyr4406\|hypothetical protein |
| *Xoo4464* | 2.44 |  | hypothetical protein |
| *Xoo4475* | 2.80 | Q | hemagglutinin |
| *Xoo4489* | 2.03 | V | BacA; phosphatase activity in Escherichia coli not kinase; involved in bacitracin resistance as bacitracin supposedly sequesters undecaprenyl disphosphate which reduces the pool of lipid carrier available to the cell\|undecaprenyl pyrophosphate phosphatase |
| *Xoo4554* | 8.23 | P | hypothetical protein |
| *Xoo4555* | 18.20 | P | alkaline phosphatase |
| *Xoo4579* | 2.46 | L | transposase |
| *Xoo4718* | 3.14 |  | hypothetical protein |
| *Xoo4720* | 3.20 | R | identified by sequence similarity; ORF located using Blastx/FrameD\|plasmid-related lipoprotein |
| *Xoo4721* | 2.15 |  | hypothetical protein |
| *Xoo4739* | 2.53 |  | hypothetical protein |
| *Xoo4744* | 2.15 | SI | identified by sequence similarity; ORF located using Blastx/FrameD\|carbamoyl-phosphate synthase large subunit |
| *Xoo4748* | 2.06 | J | hypothetical protein |
| *Xoo4766* | 5.38 |  | hypothetical protein |
| *Xoo4794* | 2.18 |  | hypothetical protein |
| *Xoo4799* | 3.29 | L | identified by sequence similarity; ORF located using Blastx/FrameD\|IS1404 transposase |
| *Xoo4823* | 2.21 |  | hypothetical protein |
| *Xoo4922* | 2.18 | S | identified by sequence similarity; ORF located using Blastx/FrameD\|hypothetical protein |
| **15 min downregulated** | | | |
| *Xoo0248* | 0.42 | S | tryptophan halogenase |
| *Xoo0249* | 0.35 | S | OmpA-like protein |
| *Xoo0252* | 0.21 | K | LacI family transcriptional regulator |
| *Xoo0301* | 0.49 | L | transposase |
| *Xoo0339* | 0.26 | G | fucose permease |
| *Xoo0340* | 0.27 | S | hypothetical protein |
| *Xoo0392* | 0.21 | S | Pass1-like protein |
| *Xoo0393* | 0.19 | S | protein SapC protein |
| *Xoo0394* | 0.03 | P | TonB-dependent receptor |
| *Xoo0556* | 0.36 | I | an AccC homodimer forms the biotin carboxylase subunit of the acetyl CoA carboxylase, an enzyme that catalyzes the formation of malonyl-CoA, which in turn controls the rate of fatty acid metabolism\|acetyl-CoA carboxylase biotin carboxylase subunit |
| *Xoo0557* | 0.37 | S | crystal structure of protein from Xanthomonas shows pentameric toroidal structure; physiological function is unknown\|hypothetical protein |
| *Xoo0558* | 0.32 | I | composes the biotin carboxyl carrier protein subunit of the acetyl-CoA carboxylase complex, the enzyme that catalyzes the carboxylation of acetyl-CoA to malonyl-CoA, which in turn controls the rate of fatty acid metabolism\|acetyl-CoA carboxylase biotin carboxyl carrier protein subunit |
| *Xoo0584* | 0.45 | S | hypothetical protein |
| *Xoo0659* | 0.38 | E | amino acid transporter |
| *Xoo0761* | 0.32 | I | beta-ketoacyl- synthase I |
| *Xoo0762* | 0.33 | I | catalyzes the dehydration of (3R)-3-hydroxydecanoyl-ACP to 2,3-decenoyl-ACP or 3,4-decenoyl-ACP\|3-hydroxydecanoyl-ACP dehydratase |
| *Xoo0880* | 0.38 | I | ACP S-malonyltransferase |
| *Xoo0881* | 0.33 | IQ | Catalyzes the first of the two reduction steps in the elongation cycle of fatty acid synthesis\|3-ketoacyl-ACP reductase |
| *Xoo0892* | 0.13 | G | catalyzes the formation of pyruvate and succinate from 2-methylisocitrate\|2-methylisocitrate lyase |
| *Xoo0893* | 0.36 | C | catalyzes the synthesis of 2-methylcitrate from propionyl-CoA and oxaloacetate; also catalyzes the condensation of oxaloacetate with acetyl-CoA but with a lower specificity\|methylcitrate synthase |
| *Xoo0894* | 0.40 | C | catalyzes the conversion of citrate to isocitrate\|aconitate hydratase |
| *Xoo1053* | 0.20 | L | transposase |
| *Xoo1099* | 0.37 | P | TonB-dependent receptor |
| *Xoo1100* | 0.25 | G | sugar transporter |
| *Xoo1116* | 0.42 | P | polyphosphate-selective porin O |
| *Xoo1158* | 0.48 | U | fimbrial assembly membrane protein |
| *Xoo1161* | 0.49 | U | fimbrial assembly protein |
| *Xoo1188* | 0.42 | T | response regulator |
| *Xoo1189* | 0.45 | T | Avin0574\|Signal transduction histidine kinase |
| *Xoo1261* | 0.34 | S | hypothetical protein |
| *Xoo1262* | 0.38 | K | hypothetical protein |
| *Xoo1263* | 0.28 | S | tryptophan halogenase |
| *Xoo1337* | 0.48 | O | ATP-dependent Clp protease subunit |
| *Xoo1354* | 0.08 | E | diaminopimelate decarboxylase |
| *Xoo1355* | 0.06 | Q | iron transporter |
| *Xoo1356* | 0.06 | P | transporter |
| *Xoo1357* | 0.04 | Q | hypothetical protein |
| *Xoo1358* | 0.03 | I | hypothetical protein |
| *Xoo1359* | 0.03 | P | citrate-dependent iron transporter |
| *Xoo1360* | 0.04 | G | 4-hydroxy-2-oxovalerate aldolase |
| *Xoo1467* | 0.44 | T | response regulator |
| *Xoo1469* | 0.38 | NT | chemotaxis protein |
| *Xoo1530* | 0.23 | P | TonB-dependent receptor |
| *Xoo1569* | 0.43 | L | transposase |
| *Xoo1604* | 0.45 | P | ferric enterobactin receptor |
| *Xoo1730* | 0.48 | G | glucose kinase |
| *Xoo1769* | 0.15 | G | beta-galactosidase |
| *Xoo1770* | 0.13 | G | hypothetical protein |
| *Xoo1771* | 0.13 | S | hypothetical protein |
| *Xoo1783* | 0.50 | G | alpha-L-fucosidase |
| *Xoo1784* | 0.45 | P | TonB-dependent receptor |
| *Xoo1821* | 0.43 | E | L-serine dehydratase |
| *Xoo1823* | 0.41 | S | cold shock domain-containing protein |
| *Xoo1842* | 0.20 | C | methylmalonate-semialdehyde dehydrogenase |
| *Xoo1843* | 0.18 | I | acyl-CoA dehydrogenase |
| *Xoo1844* | 0.21 | S | Catalyzes the reversible hydration of unsaturated fatty acyl-CoA to beta-hydroxyacyl-CoA\|enoyl-CoA hydratase |
| *Xoo1845* | 0.24 | I | enoyl-CoA hydratase |
| *Xoo1846* | 0.23 | I | 3-hydroxyisobutyrate dehydrogenase |
| *Xoo1992* | 0.05 | P | iron permease |
| *Xoo1993* | 0.23 | R | penicillin acylase II |
| *Xoo2030* | 0.46 | O | with DnaK and DnaJ acts in response to hyperosmotic and heat shock by preventing the aggregation of stress-denatured proteins; may act as a thermosensor\|heat shock protein GrpE |
| *Xoo2031* | 0.50 | O | heat shock protein 70; assists in folding of nascent polypeptide chains; refolding of misfolded proteins; utilizes ATPase activity to help fold; co-chaperones are DnaJ and GrpE; multiple copies in some bacteria\|molecular chaperone DnaK |
| *Xoo2069* | 0.22 | K | transcriptional regulator |
| *Xoo2070* | 0.15 | G | glucose-galactose transporter |
| *Xoo2071* | 0.16 | G | fructokinase |
| *Xoo2072* | 0.17 | G | hypothetical protein |
| *Xoo2097* | 0.18 | L | ISXo8 transposase |
| *Xoo2149* | 0.49 | O | C-type cytochrome biogenesis protein/thioredoxin |
| *Xoo2203* | 0.34 |  | hypothetical protein |
| *Xoo2204* | 0.28 | S | methyl-accepting chemotaxis protein |
| *Xoo2319* | 0.45 | G | KDPG and KHG aldolase |
| *Xoo2338* | 0.48 | C | ferredoxin-NADP reductase |
| *Xoo2351* | 0.10 | E | hypothetical protein |
| *Xoo2352* | 0.06 | G | beta-glucosidase |
| *Xoo2355* | 0.34 | R | sodium/glucose cotransport protein |
| *Xoo2356* | 0.35 | G | glucan 1,4-beta-glucosidase |
| *Xoo2507* | 0.50 |  | hypothetical protein |
| *Xoo2510* | 0.45 | O | molecular chaperone\|heat shock protein 90 |
| *Xoo2598* | 0.48 | R | hypothetical protein |
| *Xoo2599* | 0.48 | M | O-antigen biosynthesis protein |
| *Xoo2760* | 0.42 | C | ubiquinol cytochrome C oxidoreductase, cytochrome C1 subunit |
| *Xoo2761* | 0.35 | C | ubiquinol cytochrome C oxidoreductase, cytochrome B subunit |
| *Xoo2762* | 0.45 | C | ubiquinol cytochrome C oxidoreductase, iron-sulfur subunit |
| *Xoo2826* | 0.29 | P | TonB-dependent receptor |
| *Xoo2842* | 0.34 | NT | chemotaxis protein |
| *Xoo2897* | 0.16 |  | hypothetical protein |
| *Xoo2898* | 0.22 |  | hypothetical protein |
| *Xoo2899* | 0.17 | P | ferrous iron transport protein B |
| *Xoo2900* | 0.20 | P | ferrous iron transport protein |
| *Xoo2912* | 0.27 | S | hypothetical protein |
| *Xoo2913* | 0.23 | S | hypothetical protein |
| *Xoo2973* | 0.45 | S | hypothetical protein |
| *Xoo3118* | 0.44 | L | IS1113 transposase |
| *Xoo3129* | 0.15 | G | hypothetical protein |
| *Xoo3132* | 0.39 | E | amino acid permease |
| *Xoo3133* | 0.45 | S | hypothetical protein |
| *Xoo3199* | 0.38 | U | protein PilX |
| *Xoo3200* | 0.42 | U | hypothetical protein |
| *Xoo3201* | 0.46 | U | pre-pilin leader sequence |
| *Xoo3202* | 0.44 | U | pre-pilin like leader sequence |
| *Xoo3342* | 0.13 | R | hypothetical protein |
| *Xoo3347* | 0.17 | L | transposase |
| *Xoo3370* | 0.13 | IN | lipase/esterase |
| *Xoo3403* | 0.48 | P | TonB-dependent receptor |
| *Xoo3414* | 0.49 | R | indigoidine synthesis-like protein |
| *Xoo3433* | 0.44 | E | alanyl dipeptidyl peptidase |
| *Xoo3435* | 0.37 | S | oligopeptide transporter |
| *Xoo3617* | 0.25 | R | tryptophan repressor binding protein |
| *Xoo3661* | 0.48 | L | transposase |
| *Xoo3684* | 0.40 | C | converts threonine and NAD to 1,2-amino-3-oxobutanoate and NADH; functions in threonine catabolism\|L-threonine 3-dehydrogenase |
| *Xoo3760* | 0.27 | L | transposase |
| *Xoo3780* | 0.03 |  | outer membrane hemin receptor |
| *Xoo3781* | 0.03 | S | hypothetical protein |
| *Xoo3799* | 0.45 | R | methyltransferase |
| *Xoo3800* | 0.42 | S | sulfotransferase |
| *Xoo3879* | 0.48 | L | transposase |
| *Xoo4090* | 0.48 | L | transposase |
| *Xoo4113* | 0.40 | S | hypothetical protein |
| *Xoo4115* | 0.49 | G | dehydrogenase |
| *Xoo4136* | 0.21 | S | hypothetical protein |
| *Xoo4137* | 0.28 | C | cytochrome C oxidase subunit III |
| *Xoo4138* | 0.26 | O | involved in the insertion of copper into subunit I of cytochrome C oxidase\|cytochrome C oxidase assembly protein |
| *Xoo4140* | 0.29 | C | cytochrome C oxidase subunit I |
| *Xoo4145* | 0.49 | E | proline utilization protein A; multifunctional protein that functions in proline catabolism in the first two enzymatic steps resulting in the conversion of proline to glutamate; in Escherichia coli this protein self regulates transcription via a DNA-binding domain at the N-terminus but the proteins from this group do not and in addition appear to have a truncated C-terminal domain\|bifunctional proline dehydrogenase/pyrroline-5-carboxylate dehydrogenase |
| *Xoo4150* | 0.04 | P | bacterioferritin-associated ferredoxin |
| *Xoo4219* | 0.36 | K | MarR family transcriptional regulator |
| *Xoo4278* | 0.27 | S | hypothetical protein |
| *Xoo4377* | 0.06 | I | biotin carboxylase |
| *Xoo4378* | 0.06 | I | acyl-CoA carboxyltransferase subunit beta |
| *Xoo4379* | 0.05 | I | acyl-CoA dehydrogenase |
| *Xoo4380* | 0.28 | K | AcrR family transcriptional regulator |
| *Xoo4386* | 0.50 | S | hypothetical protein |
| *Xoo4396* | 0.27 | P | TonB-dependent receptor |
| *Xoo4431* | 0.31 | P | TonB-dependent receptor |
| *Xoo4440* | 0.22 | L | transposase |
| *Xoo4441* | 0.39 | L | transposase |
| *Xoo4446* | 0.25 | I | hypothetical protein |
| *Xoo4447* | 0.27 | C | oxidoreductase |
| *Xoo4535* | 0.20 | M | RTS beta protein |
| *Xoo4536* | 0.15 | Q | 2-hydroxyhepta-2,4-diene-1, 7-dioate isomerase |
| *Xoo4537* | 0.18 | IQ | oxidoreductase |
| *Xoo4538* | 0.16 | R | hypothetical protein |
| *Xoo4539* | 0.17 | C | oxidoreductase |
| *Xoo4545* | 0.17 | S | hypothetical protein |
| *Xoo4546* | 0.11 | S | hypothetical protein |
| *Xoo4727* | 0.46 | S | identified by sequence similarity; ORF located using Blastx/FrameD\|hypothetical protein |
| *Xoo4759* | 0.48 | S | hypothetical protein |
| *Xoo4903* | 0.36 |  | hypothetical protein |
| **30 min upregulated** | | | |
| *Xoo0038* | 2.19 | G | proline/betaine transporter |
| *Xoo0070* | 2.03 | L | ISXo8 transposase |
| *Xoo0075* | 2.03 | S | protein HpaB |
| *Xoo0095* | 2.84 | O | protein Hpa1 |
| *Xoo0096* | 3.27 | M | protein Hpa2 |
| *Xoo0097* | 2.84 | S | hypothetical protein |
| *Xoo0106* | 3.41 | L | transposase |
| *Xoo0131* | 2.01 | S | VirK protein |
| *Xoo0135* | 2.40 | C | dihydrolipoamide acyltransferase |
| *Xoo0137* | 2.09 | C | dihydrolipoamide acyltransferase |
| *Xoo0163* | 3.61 | S | hypothetical protein |
| *Xoo0172* | 2.26 | S | hypothetical protein |
| *Xoo0177* | 2.79 | E | catalyzes the formation of glutamate from glutamine and alpha-ketoglutarate\|glutamate synthase subunit alpha |
| *Xoo0178* | 2.49 | E | glutamate synthase is composed of subunits alpha and beta; beta subunit is a flavin adenine dinucleotide-NADPH dependent oxidoreductase; provides electrons to the alpha subunit, which binds L-glutamine and 2-oxoglutarate and forms L-glutamate\|glutamate synthase subunit beta |
| *Xoo0222* | 4.14 | S | Vgr-related protein |
| *Xoo0223* | 3.77 | S | hypothetical protein |
| *Xoo0279* | 2.64 | L | ISXo8 transposase |
| *Xoo0280* | 2.54 | C | alcohol dehydrogenase |
| *Xoo0281* | 3.52 | G | cellulase |
| *Xoo0283* | 2.37 | G | cellulase |
| *Xoo0297* | 4.83 | L | transposase |
| *Xoo0298* | 3.60 | L | ISXo8 transposase |
| *Xoo0316* | 3.41 | L | transposase |
| *Xoo0337* | 3.55 | S | hypothetical protein |
| *Xoo0367* | 2.09 | L | ISXo8 transposase |
| *Xoo0374* | 2.08 | L | transposase |
| *Xoo0405* | 2.60 | K | LysR family transcriptional regulator |
| *Xoo0417* | 2.33 | P | catalase |
| *Xoo0418* | 2.04 | R | ankyrin-like protein |
| *Xoo0419* | 2.54 |  | hypothetical protein |
| *Xoo0420* | 2.97 |  | hypothetical protein |
| *Xoo0432* | 2.39 | R | hypothetical protein |
| *Xoo0547* | 2.18 | L | ISXo8 transposase |
| *Xoo0637* | 2.33 | L | transposase |
| *Xoo0662* | 2.07 | O | metallopeptidase |
| *Xoo0664* | 4.13 | C | oxidoreductase |
| *Xoo0665* | 4.46 | R | tryptophan repressor binding protein |
| *Xoo0675* | 2.32 | P | Na+:H+ antiporter |
| *Xoo0687* | 2.11 | N | with MotA forms the ion channels that couple flagellar rotation to proton/sodium motive force across the membrane and forms the stator elements of the rotary flagellar machine\|flagellar motor protein MotB |
| *Xoo0688* | 2.79 | N | With MotB forms the ion channels that couple flagellar rotation to proton/sodium motive force across the membrane and forms the stator elements of the rotary flagellar machine\|flagellar motor protein MotA |
| *Xoo0717* | 3.34 | M | hypothetical protein |
| *Xoo0719* | 2.34 |  | hypothetical protein |
| *Xoo0735* | 2.22 |  | hypothetical protein |
| *Xoo0747* | 2.21 | S | hypothetical protein |
| *Xoo0748* | 4.78 | S | hypothetical protein |
| *Xoo0749* | 3.16 | L | transposase |
| *Xoo0768* | 11.26 |  | hypothetical protein |
| *Xoo0770* | 3.81 | L | ATP-dependent RNA helicase |
| *Xoo0786* | 2.41 | L | IS1478 transposase |
| *Xoo0901* | 16.19 | P | TonB-dependent receptor |
| *Xoo0902* | 9.37 | C | glycerophosphodiester phosphodiesterase |
| *Xoo0903* | 6.59 | V | hypothetical protein |
| *Xoo0904* | 2.77 | L | ISXo8 transposase |
| *Xoo0929* | 3.22 | K | transcriptional regulator |
| *Xoo0930* | 2.54 | T | hypothetical protein |
| *Xoo0949* | 2.14 | K | transcriptional regulator |
| *Xoo1011* | 14.79 | L | transposase |
| *Xoo1014* | 2.24 | M | hypothetical protein |
| *Xoo1044* | 8.46 | L | transposase |
| *Xoo1047* | 5.43 | S | Vgr-related protein |
| *Xoo1048* | 3.01 | S | hypothetical protein |
| *Xoo1075* | 2.02 | J | cleaves off formyl group from N-terminal methionine residues of newly synthesized proteins; binds iron(2+)\|peptide deformylase |
| *Xoo1102* | 4.44 | IQ | catalyzes the first of the two reduction steps in the elongation cycle of fatty acid synthesis\|3-ketoacyl-ACP reductase |
| *Xoo1103* | 5.80 | C | Mg++/citrate complex transporter |
| *Xoo1104* | 10.23 | P | porin |
| *Xoo1117* | 2.56 | C | involved in the transport of C4-dicarboxylates across the membrane\|C4-dicarboxylate transporter DctA |
| *Xoo1136* | 3.41 | L | transposase |
| *Xoo1184* | 3.08 | SI | long-chain acyl-CoA synthetase |
| *Xoo1235* | 2.28 | L | Avin3395\|transposase |
| *Xoo1259* | 2.25 | E | peptidase |
| *Xoo1269* | 2.07 | R | proline imino-peptidase |
| *Xoo1273* | 4.25 | C | quinol oxidase subunit I |
| *Xoo1274* | 2.61 | C | quinol oxidase subunit II |
| *Xoo1309* | 5.40 | L | DNA transport competence protein |
| *Xoo1315* | 2.52 | L | transposase |
| *Xoo1379* | 2.19 | T | HrpG protein |
| *Xoo1380* | 4.84 | T | HrpX protein |
| *Xoo1401* | 2.16 | K | acetyltransferase |
| *Xoo1412* | 4.89 |  | hypothetical protein |
| *Xoo1448* | 4.31 | R | pirin |
| *Xoo1449* | 4.94 | O | hypothetical protein |
| *Xoo1471* | 3.04 | S | hypothetical protein |
| *Xoo1480* | 2.83 | O | cysteine protease |
| *Xoo1487* | 3.91 | O | cysteine protease |
| *Xoo1497* | 2.44 | O | cysteine protease |
| *Xoo1502* | 2.92 | R | nitrilase |
| *Xoo1518* | 3.30 |  | hypothetical protein |
| *Xoo1551* | 2.61 | T | hypothetical protein |
| *Xoo1629* | 2.02 | C | cytochrome O ubiquinol oxidase subunit II |
| *Xoo1682* | 2.63 |  | hypothetical protein |
| *Xoo1684* | 2.43 |  | hypothetical protein |
| *Xoo1688* | 2.68 | K | Z2969\|hypothetical protein |
| *Xoo1698* | 2.09 | S | hypothetical protein |
| *Xoo1699* | 2.07 | R | I\|phage-related tail protein |
| *Xoo1712* | 2.15 | R | N\|phage-related major capsid protein |
| *Xoo1755* | 2.03 | L | tISRso5\|ISRSO5-transposase protein |
| *Xoo1756* | 2.22 | R | hypothetical protein |
| *Xoo1803* | 4.97 | R | hypothetical protein |
| *Xoo1824* | 2.73 | S | hypothetical protein |
| *Xoo1825* | 2.85 | C | cytochrome B561 |
| *Xoo1826* | 2.09 | S | hypothetical protein |
| *Xoo1827* | 2.03 | T | histidine kinase-response regulator hybrid protein |
| *Xoo1833* | 3.71 | L | hypothetical protein |
| *Xoo1901* | 28.94 | G | MFS transporter |
| *Xoo1952* | 3.37 | I | PHB depolymerase |
| *Xoo1978* | 2.90 | NU | pili assembly chaperone |
| *Xoo1994* | 5.28 | P | bacterioferritin |
| *Xoo1998* | 4.57 | K | MarR family transcriptional regulator |
| *Xoo1999* | 4.25 | M | outer membrane efflux protein |
| *Xoo2000* | 3.78 | V | multidrug resistance efflux pump |
| *Xoo2001* | 4.68 | P | multidrug resistance membrane translocase |
| *Xoo2012* | 14.46 | L | Reut4275\|IS30 family transposase |
| *Xoo2075* | 2.14 | KQ | transcriptional regulator |
| *Xoo2076* | 2.93 | I | acyl-CoA dehydrogenase |
| *Xoo2082* | 2.97 | L | hypothetical protein |
| *Xoo2089* | 2.40 | L | ISXo8 transposase |
| *Xoo2102* | 3.50 | L | ISXo8 transposase |
| *Xoo2110* | 2.05 | L | transposase |
| *Xoo2111* | 2.15 | L | ISXo8 transposase |
| *Xoo2122* | 6.17 | L | transposase |
| *Xoo2123* | 5.54 | I | ACP phosphodieterase |
| *Xoo2124* | 2.17 | K | transcriptional regulator |
| *Xoo2132* | 2.01 | L | ISXo8 transposase |
| *Xoo2133* | 3.92 | R | hypothetical protein |
| *Xoo2168* | 2.19 | S | hypothetical protein |
| *Xoo2228* | 2.23 | T | two-component system sensor protein |
| *Xoo2231* | 2.59 | G | Prop transport protein |
| *Xoo2271* | 2.29 | L | transposase |
| *Xoo2280* | 2.03 | L | Avin3395\|transposase |
| *Xoo2348* | 3.49 |  | hypothetical protein |
| *Xoo2354* | 3.14 | T | hypothetical protein |
| *Xoo2375* | 6.58 | O | serine protease |
| *Xoo2376* | 3.26 | P | cation efflux system protein |
| *Xoo2377* | 2.50 | P | cation efflux system protein |
| *Xoo2465* | 2.12 | P | ATP-binding protein; PstABCS is an ATP dependent phosphate uptake system which is responsible for inorganic phosphate uptake during phosphate starvation\|phosphate transporter ATP-binding protein |
| *Xoo2493* | 4.27 | L | ISXo8 transposase |
| *Xoo2498* | 2.70 |  | hypothetical protein |
| *Xoo2520* | 3.30 | L | ISxac1 transposase |
| *Xoo2523* | 2.22 | L | transposase |
| *Xoo2558* | 3.32 | S | chemotaxis protein |
| *Xoo2559* | 2.16 |  | hypothetical protein |
| *Xoo2561* | 2.81 | T | hypothetical protein |
| *Xoo2565* | 2.33 |  | hypothetical protein |
| *Xoo2568* | 3.44 | NT | chemotaxis protein |
| *Xoo2571* | 2.28 | N | acts as a scaffold for the assembly of hook proteins onto the flagellar basal body rod\|flagellar basal body rod modification protein |
| *Xoo2574* | 2.38 | N | FlgF, with FlgB and C, makes up the proximal portion of the flagellar basal body rod\|flagellar basal body rod protein FlgF |
| *Xoo2578* | 2.45 | NU | Flagellum-specific muramidase which hydrolyzes the peptidoglycan layer to assemble the rod structure in the periplasmic space\|flagellar rod assembly protein/muramidase FlgJ |
| *Xoo2579* | 2.21 | N | with FlgL acts as a hook filament junction protein to join the flagellar filament to the hook\|flagellar hook-associated protein FlgK |
| *Xoo2580* | 2.47 | N | with FlgK acts as a hook filament junction protein to join the flagellar filament to the hook; Yersinia, Vibrio parahaemolyticus, Bradyrhizobium and other organisms have 2 copies of this and other flagellar genes.\|flagellar hook-associated protein FlgL |
| *Xoo2581* | 2.37 | N | structural flagella protein; Vibrio contains multiple flagellin genes usually localized into two region on the chromosome, flaAC and flaCEDB in V. cholerae, flaFBA and flaCDE in V. parahemolyticus; FlaA is sigma 54 dependent and essential for motility in V.cholerae but not in V. parahaemolyticus\|flagellin |
| *Xoo2583* | 2.63 | N | flagellar protein |
| *Xoo2585* | 2.39 | S | hypothetical protein |
| *Xoo2587* | 2.77 | K | RNA polymerase sigma-54 factor |
| *Xoo2588* | 2.02 | T | response regulator |
| *Xoo2596* | 2.06 | P | ring hydroxylating dioxygenase subunit alpha |
| *Xoo2600* | 2.63 | N | flagellar protein |
| *Xoo2601* | 2.45 | N | the MS-ring anchors the flagellum to the cytoplasmic membrane; part of the flagellar basal body which consists of four rings L, P, S, and M mounted on a central rod\|flagellar MS-ring protein |
| *Xoo2602* | 2.11 | N | flagellar protein |
| *Xoo2605* | 2.09 | N | flagellar FliJ protein |
| *Xoo2607* | 2.47 | N | flagellar protein |
| *Xoo2608* | 2.24 | N | with FliG and FliN makes up the switch complex which is involved in switching the direction of the flagella rotation\|flagellar motor switch protein FliM |
| *Xoo2609* | 2.25 | N | flagellar protein |
| *Xoo2610* | 2.37 | N | flagellar protein |
| *Xoo2611* | 2.61 | N | FliP, with proteins FliQ and FliR, forms the core of the central channel in the flagella export apparatus\|flagellar biosynthesis protein FliP |
| *Xoo2612* | 2.50 | N | flagellar biosynthesis |
| *Xoo2613* | 2.08 | N | flagellar biosynthetic protein |
| *Xoo2616* | 2.13 | T | diguanylate cyclase |
| *Xoo2617* | 2.16 | N | membrane protein responsible for substrate specificity switching from rod/hook-type export to filament-type export\|flagellar biosynthesis protein FlhB |
| *Xoo2620* | 2.01 | D | flagellar biosynthesis switch protein |
| *Xoo2621* | 2.23 | K | RNA polymerase sigma factor FliA |
| *Xoo2622* | 2.28 | T | chemotaxis protein |
| *Xoo2623* | 2.70 | T | chemotaxis related protein |
| *Xoo2624* | 2.61 | T | chemotaxis related protein |
| *Xoo2627* | 3.97 | L | Reut4275\|IS30 family transposase |
| *Xoo2685* | 3.05 | E | ethanolamine ammonia-lyase light subunit |
| *Xoo2745* | 3.32 | L | RSp1628\|hypothetical protein |
| *Xoo2748* | 2.40 | L | ISXo8 transposase |
| *Xoo2778* | 2.16 | M | outer membrane protein OprN |
| *Xoo2779* | 2.32 | P | transporter |
| *Xoo2780* | 2.16 | V | transporter |
| *Xoo2787* | 3.35 | T | transcriptional regulator |
| *Xoo2799* | 3.02 | P | drug resistance translocase |
| *Xoo2803* | 2.81 | K | TetR/AcrR family transcriptional regulator |
| *Xoo2804* | 2.03 | M | multidrug resistance protein |
| *Xoo2805* | 2.12 | P | multidrug efflux transporter |
| *Xoo2830* | 3.04 | N | homologous to MotA; this protein with a related protein (a MotB homolog) forms the ion channels that couple flagellar rotation to proton/sodium motive force across the membrane and forms the stator elements of the rotary flagellar machine; either MotAB or MotCD is sufficient for swimming, but both are necessary for swarming motility; these organisms have both MotA and MotC\|flagellar motor protein |
| *Xoo2831* | 2.66 | N | Homologous to MotB. These organism have both MotB and MotD. With MotC (a MotA homolog) forms the ion channels that couple flagellar rotation to proton/sodium motive force across the membrane and forms the stator elements of the rotary flagellar machine. Either MotAB or MotCD is sufficient for swimming, but both are necessary for swarming motility\|flagellar motor protein MotD |
| *Xoo2832* | 2.32 | D | chromosome partioning protein |
| *Xoo2833* | 2.62 | S | chemotaxis protein |
| *Xoo2834* | 2.34 |  | hypothetical protein |
| *Xoo2835* | 2.17 | T | chemotaxis response regulator |
| *Xoo2836* | 2.53 | T | chemotaxis protein |
| *Xoo2844* | 2.50 | NT | chemotaxis protein |
| *Xoo2847* | 5.19 | NT | chemotaxis protein |
| *Xoo2848* | 3.44 | NT | chemotaxis protein |
| *Xoo2849* | 3.71 | M | hypothetical protein |
| *Xoo2850* | 3.66 | NT | chemotaxis protein |
| *Xoo2857* | 3.54 | NT | chemotaxis protein methyltransferase |
| *Xoo2866* | 2.46 | S | hypothetical protein |
| *Xoo2893* | 3.41 | L | transposase |
| *Xoo2930* | 2.07 | R | hypothetical protein |
| *Xoo2931* | 3.32 | SP | hypothetical protein |
| *Xoo2933* | 6.41 | R | hypothetical protein |
| *Xoo2934* | 2.86 | G | endolysin |
| *Xoo2998* | 2.42 | S | hypothetical protein |
| *Xoo3027* | 2.10 | L | IS1404 transposase |
| *Xoo3046* | 2.14 | S | hypothetical protein |
| *Xoo3101* | 2.38 | T | histidine kinase/response regulator hybrid protein |
| *Xoo3126* | 2.09 | N | hypothetical protein |
| *Xoo3156* | 2.14 | L | hypothetical protein |
| *Xoo3157* | 3.01 | L | transposase |
| *Xoo3173* | 2.05 | M | protein GumH |
| *Xoo3174* | 2.31 | G | protein GumG |
| *Xoo3176* | 2.08 | S | protein GumE |
| *Xoo3177* | 2.20 | M | protein GumD |
| *Xoo3178* | 2.62 | DM | protein GumC |
| *Xoo3179* | 2.74 | M | protein GumB |
| *Xoo3243* | 3.41 | L | transposase |
| *Xoo3294* | 3.35 | R | hypothetical protein |
| *Xoo3295* | 2.86 | C | glutamate symporter |
| *Xoo3347* | 2.70 | L | transposase |
| *Xoo3351* | 2.69 | L | transposase |
| *Xoo3363* | 2.03 | R | virulence regulator |
| *Xoo3364* | 6.17 |  | hypothetical protein |
| *Xoo3428* | 2.65 |  | hypothetical protein |
| *Xoo3430* | 2.07 | S | IS1595 transposase |
| *Xoo3445* | 3.91 | L | transposase |
| *Xoo3492* | 2.60 | S | hypothetical protein |
| *Xoo3493* | 2.19 | S | RSp74\|hypothetical protein |
| *Xoo3551* | 2.21 | S | PA0821\|hypothetical protein |
| *Xoo3552* | 2.07 | L | IS1404 transposase |
| *Xoo3554* | 3.41 | L | transposase |
| *Xoo3612* | 2.75 |  | hypothetical protein |
| *Xoo3618* | 2.12 | G | xylanase |
| *Xoo3645* | 3.51 | O | alkyl hydroperoxide reductase |
| *Xoo3646* | 4.15 | O | alkyl hydroperoxide reductase |
| *Xoo3647* | 3.62 | K | oxidative stress transcriptional regulator |
| *Xoo3658* | 2.26 | S | hypothetical protein |
| *Xoo3659* | 2.86 | T | two-component system regulatory protein |
| *Xoo3662* | 2.16 | L | transposase |
| *Xoo3666* | 2.22 | T | two-component system regulatory protein |
| *Xoo3698* | 2.14 | C | 2,4-dienoyl-CoA reductase |
| *Xoo3708* | 3.01 | L | transposase |
| *Xoo3714* | 2.24 | L | IS30 family transposase |
| *Xoo3793* | 2.89 | P | TonB-dependent receptor |
| *Xoo3809* | 2.54 | S | hypothetical protein |
| *Xoo3811* | 3.30 |  | hypothetical protein |
| *Xoo3836* | 2.63 | S | hypothetical protein |
| *Xoo3855* | 2.17 |  | hypothetical protein |
| *Xoo3874* | 2.23 | L | ISXo8 transposase |
| *Xoo3909* | 2.71 | P | TonB-dependent receptor |
| *Xoo3939* | 3.36 | G | similar to full-length Gnd, these proteins seems to have a truncated C-terminal 6PGD domainin; in Methylobacillus flagellatus this gene is essential for NAD+-dependent oxidation of 6-phosphogluconate\|6-phosphogluconate dehydrogenase |
| *Xoo3977* | 2.55 | R | hypothetical protein |
| *Xoo4001* | 3.64 | F | hypothetical protein |
| *Xoo4021* | 2.41 | T | histidine kinase-response regulator hybrid protein |
| *Xoo4103* | 2.92 | L | transposase |
| *Xoo4104* | 2.33 | L | transposase |
| *Xoo4143* | 3.41 | L | transposase |
| *Xoo4149* | 5.46 | P | bacterioferritin |
| *Xoo4186* | 2.03 | L | Bcep2833\|transposase |
| *Xoo4199* | 2.60 | M | hypothetical protein |
| *Xoo4208* | 2.01 | S | hypothetical protein |
| *Xoo4220* | 2.84 | T | hypothetical protein |
| *Xoo4234* | 2.20 | L | IS1478 transposase |
| *Xoo4254* | 2.15 | L | transposase |
| *Xoo4266* | 2.17 | S | hypothetical protein |
| *Xoo4267* | 3.72 | C | hypothetical protein |
| *Xoo4280* | 2.18 | S | hypothetical protein |
| *Xoo4290* | 2.29 | L | ISxac1 transposase |
| *Xoo4294* | 2.52 | C | NADH dehydrogenase |
| *Xoo4296* | 2.03 | K | binds with the catalytic core of RNA polymerase to produce the holoenzyme; this sigma factor is responsible for the expression of heat shock promoters\|RNA polymerase factor sigma-32 |
| *Xoo4345* | 2.14 | I | dehydrogenase |
| *Xoo4346* | 2.47 | C | FMN oxidoreductase |
| *Xoo4358* | 2.05 | K | LysR family transcriptional regulator |
| *Xoo4401* | 2.52 | S | hypothetical protein |
| *Xoo4402* | 2.71 | L | transposase |
| *Xoo4428* | 2.08 | G | secreted xylanase |
| *Xoo4455* | 4.31 | K | MarR family transcriptional regulator |
| *Xoo4456* | 2.98 | O | organic hydroperoxide resistance protein |
| *Xoo4463* | 2.13 | S | Psyr4406\|hypothetical protein |
| *Xoo4472* | 3.37 | S | hypothetical protein |
| *Xoo4475* | 2.58 | Q | hemagglutinin |
| *Xoo4493* | 2.33 | S | hypothetical protein |
| *Xoo4555* | 2.59 | P | alkaline phosphatase |
| *Xoo4565* | 2.05 | H | hypothetical protein |
| *Xoo4718* | 3.28 |  | hypothetical protein |
| *Xoo4720* | 4.05 | R | identified by sequence similarity; ORF located using Blastx/FrameD\|plasmid-related lipoprotein |
| *Xoo4744* | 2.79 | SI | identified by sequence similarity; ORF located using Blastx/FrameD\|carbamoyl-phosphate synthase large subunit |
| *Xoo4922* | 2.31 | S | identified by sequence similarity; ORF located using Blastx/FrameD\|hypothetical protein |
| *Xoo4951* | 2.76 |  | hypothetical protein |
| *YXoop10* | 2.19 |  | hypothetical protein |
| **30 min downregulated** | | | |
| *Xoo0040* | 0.48 | O | thioredoxin |
| *Xoo0041* | 0.45 | N | AtsE |
| *Xoo0148* | 0.50 | L | transposase |
| *Xoo0248* | 0.42 | S | tryptophan halogenase |
| *Xoo0249* | 0.28 | S | OmpA-like protein |
| *Xoo0252* | 0.17 | K | LacI family transcriptional regulator |
| *Xoo0301* | 0.48 | L | transposase |
| *Xoo0334* | 0.37 | G | NdvB protein |
| *Xoo0339* | 0.16 | G | fucose permease |
| *Xoo0340* | 0.18 | S | hypothetical protein |
| *Xoo0342* | 0.27 | L | transposase |
| *Xoo0392* | 0.18 | S | Pass1-like protein |
| *Xoo0393* | 0.15 | S | protein SapC protein |
| *Xoo0394* | 0.02 | P | TonB-dependent receptor |
| *Xoo0411* | 0.43 |  | hypothetical protein |
| *Xoo0484* | 0.47 | F | Catalyzes the cycloisomerization of cis,cis-muconate\|3-carboxy-cis,cis-muconate cycloisomerase |
| *Xoo0556* | 0.35 | I | an AccC homodimer forms the biotin carboxylase subunit of the acetyl CoA carboxylase, an enzyme that catalyzes the formation of malonyl-CoA, which in turn controls the rate of fatty acid metabolism\|acetyl-CoA carboxylase biotin carboxylase subunit |
| *Xoo0557* | 0.34 | S | crystal structure of protein from Xanthomonas shows pentameric toroidal structure; physiological function is unknown\|hypothetical protein |
| *Xoo0558* | 0.32 | I | composes the biotin carboxyl carrier protein subunit of the acetyl-CoA carboxylase complex, the enzyme that catalyzes the carboxylation of acetyl-CoA to malonyl-CoA, which in turn controls the rate of fatty acid metabolism\|acetyl-CoA carboxylase biotin carboxyl carrier protein subunit |
| *Xoo0584* | 0.47 | S | hypothetical protein |
| *Xoo0671* | 0.40 | M | hypothetical protein |
| *Xoo0701* | 0.47 |  | hypothetical protein |
| *Xoo0708* | 0.49 | R | hypothetical protein |
| *Xoo0740* | 0.20 | L | transposase |
| *Xoo0761* | 0.27 | I | beta-ketoacyl- synthase I |
| *Xoo0762* | 0.29 | I | catalyzes the dehydration of (3R)-3-hydroxydecanoyl-ACP to 2,3-decenoyl-ACP or 3,4-decenoyl-ACP\|3-hydroxydecanoyl-ACP dehydratase |
| *Xoo0880* | 0.36 | I | ACP S-malonyltransferase |
| *Xoo0881* | 0.32 | IQ | Catalyzes the first of the two reduction steps in the elongation cycle of fatty acid synthesis\|3-ketoacyl-ACP reductase |
| *Xoo0892* | 0.12 | G | catalyzes the formation of pyruvate and succinate from 2-methylisocitrate\|2-methylisocitrate lyase |
| *Xoo0893* | 0.35 | C | catalyzes the synthesis of 2-methylcitrate from propionyl-CoA and oxaloacetate; also catalyzes the condensation of oxaloacetate with acetyl-CoA but with a lower specificity\|methylcitrate synthase |
| *Xoo0894* | 0.36 | C | catalyzes the conversion of citrate to isocitrate\|aconitate hydratase |
| *Xoo0895* | 0.44 |  | hypothetical protein |
| *Xoo1030* | 0.23 | L | Bcep0071\|DNA replication protein |
| *Xoo1053* | 0.39 | L | transposase |
| *Xoo1098* | 0.43 | G | amylosucrase or alpha amylase |
| *Xoo1099* | 0.28 | P | TonB-dependent receptor |
| *Xoo1100* | 0.24 | G | sugar transporter |
| *Xoo1189* | 0.49 | T | Avin0574\|Signal transduction histidine kinase |
| *Xoo1261* | 0.27 | S | hypothetical protein |
| *Xoo1262* | 0.34 | K | hypothetical protein |
| *Xoo1263* | 0.24 | S | tryptophan halogenase |
| *Xoo1266* | 0.47 | S | endoproteinase ArgC |
| *Xoo1354* | 0.08 | E | diaminopimelate decarboxylase |
| *Xoo1355* | 0.06 | Q | iron transporter |
| *Xoo1356* | 0.06 | P | transporter |
| *Xoo1357* | 0.04 | Q | hypothetical protein |
| *Xoo1358* | 0.03 | I | hypothetical protein |
| *Xoo1359* | 0.08 | P | citrate-dependent iron transporter |
| *Xoo1360* | 0.10 | G | 4-hydroxy-2-oxovalerate aldolase |
| *Xoo1475* | 0.31 | L | transposase |
| *Xoo1530* | 0.29 | P | TonB-dependent receptor |
| *Xoo1569* | 0.18 | L | transposase |
| *Xoo1604* | 0.46 | P | ferric enterobactin receptor |
| *Xoo1658* | 0.47 | R | hypothetical protein |
| *Xoo1730* | 0.44 | G | glucose kinase |
| *Xoo1769* | 0.09 | G | beta-galactosidase |
| *Xoo1770* | 0.09 | G | hypothetical protein |
| *Xoo1771* | 0.07 | S | hypothetical protein |
| *Xoo1782* | 0.50 | G | hypothetical protein |
| *Xoo1783* | 0.42 | G | alpha-L-fucosidase |
| *Xoo1784* | 0.35 | P | TonB-dependent receptor |
| *Xoo1791* | 0.34 | GS | hypothetical protein |
| *Xoo1816* | 0.49 | M | hypothetical protein |
| *Xoo1821* | 0.45 | E | L-serine dehydratase |
| *Xoo1838* | 0.50 | G | beta-galactosidase |
| *Xoo1842* | 0.21 | C | methylmalonate-semialdehyde dehydrogenase |
| *Xoo1843* | 0.14 | I | acyl-CoA dehydrogenase |
| *Xoo1844* | 0.18 | S | Catalyzes the reversible hydration of unsaturated fatty acyl-CoA to beta-hydroxyacyl-CoA\|enoyl-CoA hydratase |
| *Xoo1845* | 0.19 | I | enoyl-CoA hydratase |
| *Xoo1846* | 0.22 | I | 3-hydroxyisobutyrate dehydrogenase |
| *Xoo1912* | 0.47 | S | hypothetical protein |
| *Xoo1992* | 0.09 | P | iron permease |
| *Xoo1993* | 0.48 | R | penicillin acylase II |
| *Xoo2031* | 0.38 | O | heat shock protein 70; assists in folding of nascent polypeptide chains; refolding of misfolded proteins; utilizes ATPase activity to help fold; co-chaperones are DnaJ and GrpE; multiple copies in some bacteria\|molecular chaperone DnaK |
| *Xoo2069* | 0.22 | K | transcriptional regulator |
| *Xoo2070* | 0.12 | G | glucose-galactose transporter |
| *Xoo2071* | 0.12 | G | fructokinase |
| *Xoo2072* | 0.13 | G | hypothetical protein |
| *Xoo2319* | 0.50 | G | KDPG and KHG aldolase |
| *Xoo2351* | 0.08 | E | hypothetical protein |
| *Xoo2352* | 0.05 | G | beta-glucosidase |
| *Xoo2355* | 0.31 | R | sodium/glucose cotransport protein |
| *Xoo2356* | 0.30 | G | glucan 1,4-beta-glucosidase |
| *Xoo2382* | 0.40 | L | transposase |
| *Xoo2383* | 0.38 | S | hypothetical protein |
| *Xoo2419* | 0.42 |  | hypothetical protein |
| *Xoo2507* | 0.49 |  | hypothetical protein |
| *Xoo2510* | 0.44 | O | molecular chaperone\|heat shock protein 90 |
| *Xoo2536* | 0.45 | K | TetR family transcriptional regulator |
| *Xoo2537* | 0.35 | I | 3-hydroxyacyl-CoA dehydrogenase |
| *Xoo2538* | 0.38 | I | Catalyzes the synthesis of acetoacetyl coenzyme A from two molecules of acetyl coenzyme A. It can also act as a thiolase, catalyzing the reverse reaction and generating two-carbon units from the four-carbon product of fatty acid oxidation\|acetyl-CoA acetyltransferase |
| *Xoo2645* | 0.44 | C | cytochrome C4 |
| *Xoo2729* | 0.41 | S | hypothetical protein |
| *Xoo2760* | 0.36 | C | ubiquinol cytochrome C oxidoreductase, cytochrome C1 subunit |
| *Xoo2761* | 0.26 | C | ubiquinol cytochrome C oxidoreductase, cytochrome B subunit |
| *Xoo2762* | 0.40 | C | ubiquinol cytochrome C oxidoreductase, iron-sulfur subunit |
| *Xoo2798* | 0.38 | T | two-component system regulatory protein |
| *Xoo2819* | 0.42 | L | ISxac3 transposase |
| *Xoo2826* | 0.28 | P | TonB-dependent receptor |
| *Xoo2897* | 0.26 |  | hypothetical protein |
| *Xoo2898* | 0.31 |  | hypothetical protein |
| *Xoo2899* | 0.43 | P | ferrous iron transport protein B |
| *Xoo2912* | 0.15 | S | hypothetical protein |
| *Xoo2913* | 0.20 | S | hypothetical protein |
| *Xoo2917* | 0.46 | G | catalyzes the formation of D-glyceraldehyde 3-phosphate and pyruvate from 2-dehydro-3-deoxy-D-galactonate 6-phosphate; functions in galactonate metabolism\|2-dehydro-3-deoxy-6-phosphogalactonate aldolase |
| *Xoo3089* | 0.46 | L | transposase |
| *Xoo3111* | 0.31 | L | transposase |
| *Xoo3129* | 0.26 | G | hypothetical protein |
| *Xoo3132* | 0.48 | E | amino acid permease |
| *Xoo3149* | 0.44 | L | IS1478 transposase |
| *Xoo3199* | 0.41 | U | protein PilX |
| *Xoo3200* | 0.43 | U | hypothetical protein |
| *Xoo3201* | 0.47 | U | pre-pilin leader sequence |
| *Xoo3202* | 0.46 | U | pre-pilin like leader sequence |
| *Xoo3292* | 0.49 | M | hypothetical protein |
| *Xoo3319* | 0.31 | L | transposase |
| *Xoo3342* | 0.17 | R | hypothetical protein |
| *Xoo3370* | 0.12 | IN | lipase/esterase |
| *Xoo3402* | 0.43 | S | hypothetical protein |
| *Xoo3403* | 0.45 | P | TonB-dependent receptor |
| *Xoo3414* | 0.48 | R | indigoidine synthesis-like protein |
| *Xoo3423* | 0.40 | P | catalase |
| *Xoo3424* | 0.49 | S | RSp1239\|hypothetical protein |
| *Xoo3433* | 0.43 | E | alanyl dipeptidyl peptidase |
| *Xoo3435* | 0.33 | S | oligopeptide transporter |
| *Xoo3446* | 0.31 | L | transposase |
| *Xoo3617* | 0.17 | R | tryptophan repressor binding protein |
| *Xoo3684* | 0.39 | C | converts threonine and NAD to 1,2-amino-3-oxobutanoate and NADH; functions in threonine catabolism\|L-threonine 3-dehydrogenase |
| *Xoo3717* | 0.31 | L | transposase |
| *Xoo3759* | 0.32 | E | hypothetical protein |
| *Xoo3775* | 0.44 |  | hypothetical protein |
| *Xoo3780* | 0.07 |  | outer membrane hemin receptor |
| *Xoo3781* | 0.05 | S | hypothetical protein |
| *Xoo3799* | 0.38 | R | methyltransferase |
| *Xoo3800* | 0.38 | S | sulfotransferase |
| *Xoo4055* | 0.31 | L | transposase |
| *Xoo4071* | 0.44 | E | 4-hydroxyphenylpyruvate dioxygenase |
| *Xoo4096* | 0.33 | S | hypothetical protein |
| *Xoo4113* | 0.22 | S | hypothetical protein |
| *Xoo4115* | 0.35 | G | dehydrogenase |
| *Xoo4136* | 0.20 | S | hypothetical protein |
| *Xoo4137* | 0.22 | C | cytochrome C oxidase subunit III |
| *Xoo4138* | 0.26 | O | involved in the insertion of copper into subunit I of cytochrome C oxidase\|cytochrome C oxidase assembly protein |
| *Xoo4140* | 0.21 | C | cytochrome C oxidase subunit I |
| *Xoo4141* | 0.39 | C | cytochrome C oxidase subunit II |
| *Xoo4145* | 0.42 | E | proline utilization protein A; multifunctional protein that functions in proline catabolism in the first two enzymatic steps resulting in the conversion of proline to glutamate; in Escherichia coli this protein self regulates transcription via a DNA-binding domain at the N-terminus but the proteins from this group do not and in addition appear to have a truncated C-terminal domain\|bifunctional proline dehydrogenase/pyrroline-5-carboxylate dehydrogenase |
| *Xoo4150* | 0.15 | P | bacterioferritin-associated ferredoxin |
| *Xoo4219* | 0.36 | K | MarR family transcriptional regulator |
| *Xoo4276* | 0.43 | G | gluconolactonase |
| *Xoo4288* | 0.46 | O | 60 kDa chaperone family; promotes refolding of misfolded polypeptides especially under stressful conditions; forms two stacked rings of heptamers to form a barrel-shaped 14mer; ends can be capped by GroES; misfolded proteins enter the barrel where they are refolded when GroES binds; many bacteria have multiple copies of the groEL gene which are active under different environmental conditions; the B.japonicum protein in this cluster is expressed constitutively; in Rhodobacter, Corynebacterium and Rhizobium this protein is essential for growth\|molecular chaperone GroEL |
| *Xoo4289* | 0.49 | O | 10 kDa chaperonin; Cpn10; GroES; forms homoheptameric ring; binds to one or both ends of the GroEL double barrel in the presence of adenine nucleotides capping it; folding of unfolded substrates initiates in a GroEL-substrate bound and capped by GroES; release of the folded substrate is dependent on ATP binding and hydrolysis in the trans ring\|co-chaperonin GroES |
| *Xoo4309* | 0.42 | L | transposase |
| *Xoo4376* | 0.46 | E | dipeptidyl anminopeptidase |
| *Xoo4377* | 0.05 | I | biotin carboxylase |
| *Xoo4378* | 0.05 | I | acyl-CoA carboxyltransferase subunit beta |
| *Xoo4379* | 0.06 | I | acyl-CoA dehydrogenase |
| *Xoo4380* | 0.16 | K | AcrR family transcriptional regulator |
| *Xoo4389* | 0.44 | S | hypothetical protein |
| *Xoo4396* | 0.26 | P | TonB-dependent receptor |
| *Xoo4431* | 0.37 | P | TonB-dependent receptor |
| *Xoo4432* | 0.49 | G | transporter |
| *Xoo4440* | 0.21 | L | transposase |
| *Xoo4446* | 0.31 | I | hypothetical protein |
| *Xoo4447* | 0.32 | C | oxidoreductase |
| *Xoo4503* | 0.35 |  | hypothetical protein |
| *Xoo4535* | 0.13 | M | RTS beta protein |
| *Xoo4536* | 0.12 | Q | 2-hydroxyhepta-2,4-diene-1, 7-dioate isomerase |
| *Xoo4537* | 0.16 | IQ | oxidoreductase |
| *Xoo4538* | 0.13 | R | hypothetical protein |
| *Xoo4539* | 0.14 | C | oxidoreductase |
| *Xoo4543* | 0.50 | T | two-component system regulatory protein |
| *Xoo4544* | 0.43 | I | Acs; catalyzes the conversion of acetate and CoA to acetyl-CoA\|acetyl-CoA synthetase |
| *Xoo4545* | 0.16 | S | hypothetical protein |
| *Xoo4546* | 0.08 | S | hypothetical protein |
| *Xoo4591* | 0.46 | L | ISXo8 transposase |
| *Xoo4637* | 0.50 | J | protein component of RNaseP which catalyzes the removal of the 5'-leader sequence from pre-tRNA to produce the mature 5'terminus; this enzyme also cleaves other RNA substrates\|ribonuclease P |
| *Xoo4727* | 0.32 | S | identified by sequence similarity; ORF located using Blastx/FrameD\|hypothetical protein |
| *Xoo4840* | 0.40 |  | hypothetical protein |
| *Xoo4955* | 0.42 | T | hypothetical protein |
| **45 min upregulated** | | | |
| *Xoo0095* | 2.17 | O | protein Hpa1 |
| *Xoo0135* | 2.32 | C | dihydrolipoamide acyltransferase |
| *Xoo0177* | 2.67 | E | catalyzes the formation of glutamate from glutamine and alpha-ketoglutarate\|glutamate synthase subunit alpha |
| *Xoo0178* | 2.50 | E | glutamate synthase is composed of subunits alpha and beta; beta subunit is a flavin adenine dinucleotide-NADPH dependent oxidoreductase; provides electrons to the alpha subunit, which binds L-glutamine and 2-oxoglutarate and forms L-glutamate\|glutamate synthase subunit beta |
| *Xoo0222* | 4.12 | S | Vgr-related protein |
| *Xoo0223* | 2.91 | S | hypothetical protein |
| *Xoo0279* | 2.30 | L | ISXo8 transposase |
| *Xoo0280* | 2.60 | C | alcohol dehydrogenase |
| *Xoo0281* | 2.00 | G | cellulase |
| *Xoo0297* | 4.91 | L | transposase |
| *Xoo0337* | 3.70 | S | hypothetical protein |
| *Xoo0405* | 2.32 | K | LysR family transcriptional regulator |
| *Xoo0417* | 2.42 | P | catalase |
| *Xoo0418* | 2.05 | R | ankyrin-like protein |
| *Xoo0419* | 2.54 |  | hypothetical protein |
| *Xoo0420* | 2.55 |  | hypothetical protein |
| *Xoo0432* | 2.22 | R | hypothetical protein |
| *Xoo0477* | 2.01 | O | thioredoxin |
| *Xoo0594* | 2.08 | H | catalyzes the formation of formate and 2,5-diamino-6-hydroxy-4-(5-phosphoribosylamino)pyrimidine from GTP\|GTP cyclohydrolase |
| *Xoo0664* | 3.57 | C | oxidoreductase |
| *Xoo0665* | 4.75 | R | tryptophan repressor binding protein |
| *Xoo0667* | 2.70 | S | hypothetical protein |
| *Xoo0675* | 2.29 | P | Na+:H+ antiporter |
| *Xoo0687* | 2.10 | N | with MotA forms the ion channels that couple flagellar rotation to proton/sodium motive force across the membrane and forms the stator elements of the rotary flagellar machine\|flagellar motor protein MotB |
| *Xoo0688* | 2.34 | N | With MotB forms the ion channels that couple flagellar rotation to proton/sodium motive force across the membrane and forms the stator elements of the rotary flagellar machine\|flagellar motor protein MotA |
| *Xoo0748* | 7.00 | S | hypothetical protein |
| *Xoo0749* | 2.99 | L | transposase |
| *Xoo0768* | 9.09 |  | hypothetical protein |
| *Xoo0770* | 4.29 | L | ATP-dependent RNA helicase |
| *Xoo0875* | 4.76 | L | transposase |
| *Xoo0901* | 2.53 | P | TonB-dependent receptor |
| *Xoo0903* | 3.21 | V | hypothetical protein |
| *Xoo0929* | 2.75 | K | transcriptional regulator |
| *Xoo0930* | 2.21 | T | hypothetical protein |
| *Xoo0988* | 2.19 | L | IS1478 transposase |
| *Xoo1011* | 7.38 | L | transposase |
| *Xoo1047* | 4.13 | S | Vgr-related protein |
| *Xoo1048* | 2.27 | S | hypothetical protein |
| *Xoo1102* | 3.33 | IQ | catalyzes the first of the two reduction steps in the elongation cycle of fatty acid synthesis\|3-ketoacyl-ACP reductase |
| *Xoo1103* | 4.55 | C | Mg++/citrate complex transporter |
| *Xoo1104* | 6.50 | P | porin |
| *Xoo1117* | 3.64 | C | involved in the transport of C4-dicarboxylates across the membrane\|C4-dicarboxylate transporter DctA |
| *Xoo1125* | 2.05 | S | hypothetical protein |
| *Xoo1184* | 2.58 | SI | long-chain acyl-CoA synthetase |
| *Xoo1186* | 3.12 | R | Avin0576\|short chain dehydrogenase |
| *Xoo1259* | 2.02 | E | peptidase |
| *Xoo1272* | 2.23 | L | transposase |
| *Xoo1273* | 4.50 | C | quinol oxidase subunit I |
| *Xoo1274* | 2.66 | C | quinol oxidase subunit II |
| *Xoo1309* | 5.56 | L | DNA transport competence protein |
| *Xoo1376* | 2.86 | S | hypothetical protein |
| *Xoo1380* | 2.36 | T | HrpX protein |
| *Xoo1400* | 2.37 | C | benzene 1,2-dioxygenase ferredoxin protein |
| *Xoo1401* | 3.08 | K | acetyltransferase |
| *Xoo1402* | 2.49 | E | cysteine desulfurase |
| *Xoo1403* | 2.38 | O | ABC transporter permease |
| *Xoo1404* | 2.34 | O | ABC transporter ATP-binding protein |
| *Xoo1405* | 2.54 | O | with SufCD activates cysteine desulfurase SufS\|cysteine desulfurase |
| *Xoo1406* | 2.27 | K | hypothetical protein |
| *Xoo1412* | 4.26 |  | hypothetical protein |
| *Xoo1448* | 5.20 | R | pirin |
| *Xoo1449* | 8.33 | O | hypothetical protein |
| *Xoo1471* | 2.21 | S | hypothetical protein |
| *Xoo1487* | 2.77 | O | cysteine protease |
| *Xoo1502* | 2.59 | R | nitrilase |
| *Xoo1518* | 3.46 |  | hypothetical protein |
| *Xoo1532* | 2.54 | P | outer membrane hemin receptor |
| *Xoo1551* | 2.61 | T | hypothetical protein |
| *Xoo1681* | 2.03 |  | hypothetical protein |
| *Xoo1688* | 2.40 | K | Z2969\|hypothetical protein |
| *Xoo1756* | 2.72 | R | hypothetical protein |
| *Xoo1803* | 5.24 | R | hypothetical protein |
| *Xoo1813* | 2.67 | M | hypothetical protein |
| *Xoo1824* | 3.00 | S | hypothetical protein |
| *Xoo1825* | 3.05 | C | cytochrome B561 |
| *Xoo1826* | 2.47 | S | hypothetical protein |
| *Xoo1833* | 2.29 | L | hypothetical protein |
| *Xoo1834* | 2.57 | L | ISxac3 transposase |
| *Xoo1887* | 2.04 | T | hypothetical protein |
| *Xoo1899* | 2.08 |  | hypothetical protein |
| *Xoo1901* | 30.99 | G | MFS transporter |
| *Xoo1952* | 3.40 | I | PHB depolymerase |
| *Xoo1978* | 2.28 | NU | pili assembly chaperone |
| *Xoo1998* | 6.79 | K | MarR family transcriptional regulator |
| *Xoo1999* | 5.41 | M | outer membrane efflux protein |
| *Xoo2000* | 4.78 | V | multidrug resistance efflux pump |
| *Xoo2001* | 4.87 | P | multidrug resistance membrane translocase |
| *Xoo2012* | 5.84 | L | Reut4275\|IS30 family transposase |
| *Xoo2035* | 2.96 | L | ISXo8 transposase |
| *Xoo2074* | 2.02 | E | 5-methyltetrahydrofolate--homocysteinemethyltransferase |
| *Xoo2075* | 2.73 | KQ | transcriptional regulator |
| *Xoo2076* | 3.51 | I | acyl-CoA dehydrogenase |
| *Xoo2102* | 3.00 | L | ISXo8 transposase |
| *Xoo2122* | 6.45 | L | transposase |
| *Xoo2123* | 6.60 | I | ACP phosphodieterase |
| *Xoo2124* | 2.55 | K | transcriptional regulator |
| *Xoo2132* | 2.44 | L | ISXo8 transposase |
| *Xoo2133* | 2.31 | R | hypothetical protein |
| *Xoo2228* | 2.02 | T | two-component system sensor protein |
| *Xoo2231* | 2.33 | G | Prop transport protein |
| *Xoo2254* | 2.74 | S | hypothetical protein |
| *Xoo2255* | 2.02 | E | long form of enzyme; catalyzes the formation of N'-5'-phosphoribosyl-ATP from phosphoribosyl pyrophosphate; crucial role in histidine biosynthesis; forms active dimers and inactive hexamers which is dependent on concentration of substrates and inhibitors\|ATP phosphoribosyltransferase |
| *Xoo2280* | 2.03 | L | Avin3395\|transposase |
| *Xoo2348* | 2.35 |  | hypothetical protein |
| *Xoo2354* | 2.32 | T | hypothetical protein |
| *Xoo2359* | 2.12 |  | hypothetical protein |
| *Xoo2375* | 8.31 | O | serine protease |
| *Xoo2376* | 4.15 | P | cation efflux system protein |
| *Xoo2377* | 2.84 | P | cation efflux system protein |
| *Xoo2493* | 2.67 | L | ISXo8 transposase |
| *Xoo2498* | 3.50 |  | hypothetical protein |
| *Xoo2558* | 2.84 | S | chemotaxis protein |
| *Xoo2559* | 2.77 |  | hypothetical protein |
| *Xoo2561* | 2.68 | T | hypothetical protein |
| *Xoo2565* | 2.12 |  | hypothetical protein |
| *Xoo2568* | 2.54 | NT | chemotaxis protein |
| *Xoo2583* | 2.05 | N | flagellar protein |
| *Xoo2587* | 2.42 | K | RNA polymerase sigma-54 factor |
| *Xoo2612* | 2.05 | N | flagellar biosynthesis |
| *Xoo2623* | 2.09 | T | chemotaxis related protein |
| *Xoo2624* | 2.14 | T | chemotaxis related protein |
| *Xoo2745* | 5.04 | L | RSp1628\|hypothetical protein |
| *Xoo2764* | 2.28 | K | repressor |
| *Xoo2778* | 2.56 | M | outer membrane protein OprN |
| *Xoo2779* | 2.63 | P | transporter |
| *Xoo2780* | 2.31 | V | transporter |
| *Xoo2787* | 2.68 | T | transcriptional regulator |
| *Xoo2799* | 2.89 | P | drug resistance translocase |
| *Xoo2803* | 2.82 | K | TetR/AcrR family transcriptional regulator |
| *Xoo2804* | 2.15 | M | multidrug resistance protein |
| *Xoo2808* | 2.10 | L | transposase |
| *Xoo2832* | 2.36 | D | chromosome partioning protein |
| *Xoo2833* | 2.13 | S | chemotaxis protein |
| *Xoo2834* | 2.48 |  | hypothetical protein |
| *Xoo2836* | 2.19 | T | chemotaxis protein |
| *Xoo2847* | 3.24 | NT | chemotaxis protein |
| *Xoo2848* | 2.37 | NT | chemotaxis protein |
| *Xoo2849* | 2.50 | M | hypothetical protein |
| *Xoo2850* | 2.69 | NT | chemotaxis protein |
| *Xoo2857* | 2.51 | NT | chemotaxis protein methyltransferase |
| *Xoo2931* | 4.13 | SP | hypothetical protein |
| *Xoo2933* | 3.18 | R | hypothetical protein |
| *Xoo2979* | 2.36 | C | hypothetical protein |
| *Xoo3046* | 2.07 | S | hypothetical protein |
| *Xoo3102* | 2.17 | H | siroheme synthase |
| *Xoo3126* | 2.08 | N | hypothetical protein |
| *Xoo3150* | 2.22 | R | hypothetical protein |
| *Xoo3157* | 2.29 | L | transposase |
| *Xoo3294* | 3.72 | R | hypothetical protein |
| *Xoo3295* | 3.02 | C | glutamate symporter |
| *Xoo3351* | 3.11 | L | transposase |
| *Xoo3364* | 7.61 |  | hypothetical protein |
| *Xoo3389* | 6.11 |  | acetyltransferase |
| *Xoo3428* | 3.17 |  | hypothetical protein |
| *Xoo3445* | 3.12 | L | transposase |
| *Xoo3503* | 2.67 | S | ORF15\|VGR-related protein |
| *Xoo3552* | 2.88 | L | IS1404 transposase |
| *Xoo3612* | 2.27 |  | hypothetical protein |
| *Xoo3645* | 3.57 | O | alkyl hydroperoxide reductase |
| *Xoo3646* | 3.91 | O | alkyl hydroperoxide reductase |
| *Xoo3647* | 3.18 | K | oxidative stress transcriptional regulator |
| *Xoo3659* | 2.52 | T | two-component system regulatory protein |
| *Xoo3698* | 2.48 | C | 2,4-dienoyl-CoA reductase |
| *Xoo3708* | 2.29 | L | transposase |
| *Xoo3719* | 2.07 | EG | hypothetical protein |
| *Xoo3809* | 2.28 | S | hypothetical protein |
| *Xoo3811* | 3.47 |  | hypothetical protein |
| *Xoo3855* | 3.49 |  | hypothetical protein |
| *Xoo3874* | 2.40 | L | ISXo8 transposase |
| *Xoo3909* | 2.22 | P | TonB-dependent receptor |
| *Xoo3928* | 4.13 | L | transposase |
| *Xoo3931* | 2.00 | S | hypothetical protein |
| *Xoo3939* | 3.17 | G | similar to full-length Gnd, these proteins seems to have a truncated C-terminal 6PGD domainin; in Methylobacillus flagellatus this gene is essential for NAD+-dependent oxidation of 6-phosphogluconate\|6-phosphogluconate dehydrogenase |
| *Xoo3977* | 2.38 | R | hypothetical protein |
| *Xoo4021* | 2.16 | T | histidine kinase-response regulator hybrid protein |
| *Xoo4045* | 4.24 | S | hypothetical protein |
| *Xoo4076* | 2.34 | R | nuclease |
| *Xoo4166* | 2.25 | K | B-lactamase regulatory protein |
| *Xoo4199* | 2.01 | M | hypothetical protein |
| *Xoo4220* | 2.66 | T | hypothetical protein |
| *Xoo4233* | 2.23 | L | IS1478 transposase |
| *Xoo4267* | 3.84 | C | hypothetical protein |
| *Xoo4290* | 3.05 | L | ISxac1 transposase |
| *Xoo4294* | 2.04 | C | NADH dehydrogenase |
| *Xoo4295* | 2.03 | S | hypothetical protein |
| *Xoo4296* | 2.11 | K | binds with the catalytic core of RNA polymerase to produce the holoenzyme; this sigma factor is responsible for the expression of heat shock promoters\|RNA polymerase factor sigma-32 |
| *Xoo4345* | 2.82 | I | dehydrogenase |
| *Xoo4346* | 3.29 | C | FMN oxidoreductase |
| *Xoo4349* | 2.08 | G | MFS transporter |
| *Xoo4401* | 2.01 | S | hypothetical protein |
| *Xoo4455* | 3.20 | K | MarR family transcriptional regulator |
| *Xoo4456* | 3.30 | O | organic hydroperoxide resistance protein |
| *Xoo4463* | 2.12 | S | Psyr4406\|hypothetical protein |
| *Xoo4472* | 2.49 | S | hypothetical protein |
| *Xoo4475* | 2.74 | Q | hemagglutinin |
| *Xoo4489* | 2.25 | V | BacA; phosphatase activity in Escherichia coli not kinase; involved in bacitracin resistance as bacitracin supposedly sequesters undecaprenyl disphosphate which reduces the pool of lipid carrier available to the cell\|undecaprenyl pyrophosphate phosphatase |
| *Xoo4493* | 2.33 | S | hypothetical protein |
| *Xoo4565* | 2.24 | H | hypothetical protein |
| *Xoo4718* | 2.33 |  | hypothetical protein |
| *Xoo4721* | 2.71 |  | hypothetical protein |
| *Xoo4766* | 2.74 |  | hypothetical protein |
| *Xoo4779* | 2.05 |  | hypothetical protein |
| *Xoo4922* | 2.20 | S | identified by sequence similarity; ORF located using Blastx/FrameD\|hypothetical protein |
| *YXoop10* | 2.01 |  | hypothetical protein |
| **45 min downregulated** | | | |
| *Xoo0034* | 0.50 | S | hypothetical protein |
| *Xoo0040* | 0.19 | O | thioredoxin |
| *Xoo0041* | 0.14 | N | AtsE |
| *Xoo0050* | 0.34 |  | hypothetical protein |
| *Xoo0051* | 0.26 |  | hypothetical protein |
| *Xoo0055* | 0.50 |  | hypothetical protein |
| *Xoo0080* | 0.33 | U | hypothetical protein |
| *Xoo0108* | 0.41 | R | ABC transporter substrate binding protein |
| *Xoo0197* | 0.35 | G | alpha-amylase |
| *Xoo0248* | 0.33 | S | tryptophan halogenase |
| *Xoo0249* | 0.20 | S | OmpA-like protein |
| *Xoo0250* | 0.42 | S | OmpA-like protein |
| *Xoo0252* | 0.19 | K | LacI family transcriptional regulator |
| *Xoo0333* | 0.42 | G | cation symporter |
| *Xoo0334* | 0.40 | G | NdvB protein |
| *Xoo0339* | 0.15 | G | fucose permease |
| *Xoo0340* | 0.12 | S | hypothetical protein |
| *Xoo0392* | 0.16 | S | Pass1-like protein |
| *Xoo0393* | 0.19 | S | protein SapC protein |
| *Xoo0394* | 0.02 | P | TonB-dependent receptor |
| *Xoo0416* | 0.39 | U | Bcep7270\|membrane-fusion protein |
| *Xoo0436* | 0.27 | S | hypothetical protein |
| *Xoo0486* | 0.48 | S | 4-carboxymuconolactone decarboxylase |
| *Xoo0556* | 0.35 | I | an AccC homodimer forms the biotin carboxylase subunit of the acetyl CoA carboxylase, an enzyme that catalyzes the formation of malonyl-CoA, which in turn controls the rate of fatty acid metabolism\|acetyl-CoA carboxylase biotin carboxylase subunit |
| *Xoo0557* | 0.37 | S | crystal structure of protein from Xanthomonas shows pentameric toroidal structure; physiological function is unknown\|hypothetical protein |
| *Xoo0558* | 0.48 | I | composes the biotin carboxyl carrier protein subunit of the acetyl-CoA carboxylase complex, the enzyme that catalyzes the carboxylation of acetyl-CoA to malonyl-CoA, which in turn controls the rate of fatty acid metabolism\|acetyl-CoA carboxylase biotin carboxyl carrier protein subunit |
| *Xoo0633* | 0.43 | S | hypothetical protein |
| *Xoo0643* | 0.42 | T | regulatory protein |
| *Xoo0660* | 0.43 |  | hypothetical protein |
| *Xoo0671* | 0.30 | M | hypothetical protein |
| *Xoo0696* | 0.45 |  | hypothetical protein |
| *Xoo0697* | 0.41 |  | hypothetical protein |
| *Xoo0699* | 0.32 |  | hypothetical protein |
| *Xoo0700* | 0.37 | G | L-sorbosone dehydrogenase |
| *Xoo0701* | 0.27 |  | hypothetical protein |
| *Xoo0740* | 0.08 | L | transposase |
| *Xoo0761* | 0.31 | I | beta-ketoacyl- synthase I |
| *Xoo0762* | 0.28 | I | catalyzes the dehydration of (3R)-3-hydroxydecanoyl-ACP to 2,3-decenoyl-ACP or 3,4-decenoyl-ACP\|3-hydroxydecanoyl-ACP dehydratase |
| *Xoo0837* | 0.30 |  | hypothetical protein |
| *Xoo0842* | 0.46 | UW | hypothetical protein |
| *Xoo0880* | 0.47 | I | ACP S-malonyltransferase |
| *Xoo0881* | 0.33 | IQ | Catalyzes the first of the two reduction steps in the elongation cycle of fatty acid synthesis\|3-ketoacyl-ACP reductase |
| *Xoo0892* | 0.11 | G | catalyzes the formation of pyruvate and succinate from 2-methylisocitrate\|2-methylisocitrate lyase |
| *Xoo0893* | 0.30 | C | catalyzes the synthesis of 2-methylcitrate from propionyl-CoA and oxaloacetate; also catalyzes the condensation of oxaloacetate with acetyl-CoA but with a lower specificity\|methylcitrate synthase |
| *Xoo0894* | 0.34 | C | catalyzes the conversion of citrate to isocitrate\|aconitate hydratase |
| *Xoo0895* | 0.28 |  | hypothetical protein |
| *Xoo0947* | 0.37 | H | required for the synthesis of the hydromethylpyrimidine moiety of thiamine\|thiamine biosynthesis protein ThiC |
| *Xoo1030* | 0.24 | L | Bcep0071\|DNA replication protein |
| *Xoo1097* | 0.42 | R | NonF-like protein |
| *Xoo1098* | 0.41 | G | amylosucrase or alpha amylase |
| *Xoo1099* | 0.26 | P | TonB-dependent receptor |
| *Xoo1100* | 0.21 | G | sugar transporter |
| *Xoo1179* | 0.48 | S | hypothetical protein |
| *Xoo1188* | 0.44 | T | response regulator |
| *Xoo1189* | 0.39 | T | Avin0574\|Signal transduction histidine kinase |
| *Xoo1217* | 0.44 |  | hypothetical protein |
| *Xoo1240* | 0.43 | L | Reut4275\|IS30 family transposase |
| *Xoo1261* | 0.30 | S | hypothetical protein |
| *Xoo1262* | 0.28 | K | hypothetical protein |
| *Xoo1263* | 0.22 | S | tryptophan halogenase |
| *Xoo1264* | 0.49 | R | hydrolase |
| *Xoo1266* | 0.47 | S | endoproteinase ArgC |
| *Xoo1354* | 0.13 | E | diaminopimelate decarboxylase |
| *Xoo1355* | 0.11 | Q | iron transporter |
| *Xoo1356* | 0.11 | P | transporter |
| *Xoo1357* | 0.08 | Q | hypothetical protein |
| *Xoo1358* | 0.06 | I | hypothetical protein |
| *Xoo1359* | 0.22 | P | citrate-dependent iron transporter |
| *Xoo1360* | 0.31 | G | 4-hydroxy-2-oxovalerate aldolase |
| *Xoo1388* | 0.45 | E | catalyzes the formation of O-succinyl-L-homoserine from succinyl-CoA and L-homoserine in methionine biosynthesis\|homoserine O-succinyltransferase |
| *Xoo1389* | 0.48 | I | protein RtxC |
| *Xoo1390* | 0.48 | E | protein RtxA |
| *Xoo1391* | 0.41 | E | hypothetical protein |
| *Xoo1524* | 0.47 | R | hypothetical protein |
| *Xoo1530* | 0.18 | P | TonB-dependent receptor |
| *Xoo1542* | 0.49 |  | hypothetical protein |
| *Xoo1569* | 0.12 | L | transposase |
| *Xoo1601* | 0.47 | G | trehalose-6-phosphate synthase |
| *Xoo1602* | 0.49 | G | hypothetical protein |
| *Xoo1604* | 0.43 | P | ferric enterobactin receptor |
| *Xoo1697* | 0.42 | R | V\|phage-related baseplate protein |
| *Xoo1733* | 0.45 | R | pyrroloquinoline quinone biosynthesis protein PqqE |
| *Xoo1734* | 0.40 | H | Required in the synthesis of PPQ, but its exact function is unknown\|pyrroloquinoline quinone biosynthesis protein PqqC |
| *Xoo1735* | 0.33 | R | possibly involved in transport of pyrroloquinoline quinone transport\|pyrroloquinoline quinone biosynthesis protein PqqB |
| *Xoo1769* | 0.08 | G | beta-galactosidase |
| *Xoo1770* | 0.08 | G | hypothetical protein |
| *Xoo1771* | 0.08 | S | hypothetical protein |
| *Xoo1779* | 0.49 | G | glucan 1,4-beta-glucosidase |
| *Xoo1780* | 0.42 | G | beta-mannosidase |
| *Xoo1781* | 0.47 | G | beta-hexosaminidase |
| *Xoo1782* | 0.42 | G | hypothetical protein |
| *Xoo1783* | 0.36 | G | alpha-L-fucosidase |
| *Xoo1784* | 0.28 | P | TonB-dependent receptor |
| *Xoo1821* | 0.49 | E | L-serine dehydratase |
| *Xoo1842* | 0.18 | C | methylmalonate-semialdehyde dehydrogenase |
| *Xoo1843* | 0.14 | I | acyl-CoA dehydrogenase |
| *Xoo1844* | 0.18 | S | Catalyzes the reversible hydration of unsaturated fatty acyl-CoA to beta-hydroxyacyl-CoA\|enoyl-CoA hydratase |
| *Xoo1845* | 0.20 | I | enoyl-CoA hydratase |
| *Xoo1846* | 0.20 | I | 3-hydroxyisobutyrate dehydrogenase |
| *Xoo1902* | 0.26 | S | hypothetical protein |
| *Xoo1912* | 0.47 | S | hypothetical protein |
| *Xoo1992* | 0.28 | P | iron permease |
| *Xoo2069* | 0.21 | K | transcriptional regulator |
| *Xoo2070* | 0.10 | G | glucose-galactose transporter |
| *Xoo2071* | 0.11 | G | fructokinase |
| *Xoo2072* | 0.10 | G | hypothetical protein |
| *Xoo2083* | 0.49 | S | hypothetical protein |
| *Xoo2126* | 0.31 | S | integrase |
| *Xoo2223* | 0.37 | R | oxidoreductase |
| *Xoo2284* | 0.02 | L | transposase |
| *Xoo2347* | 0.48 | R | hypothetical protein |
| *Xoo2351* | 0.07 | E | hypothetical protein |
| *Xoo2352* | 0.04 | G | beta-glucosidase |
| *Xoo2355* | 0.27 | R | sodium/glucose cotransport protein |
| *Xoo2356* | 0.32 | G | glucan 1,4-beta-glucosidase |
| *Xoo2382* | 0.40 | L | transposase |
| *Xoo2383* | 0.21 | S | hypothetical protein |
| *Xoo2507* | 0.46 |  | hypothetical protein |
| *Xoo2519* | 0.45 | L | Avin0470\|transposase |
| *Xoo2536* | 0.47 | K | TetR family transcriptional regulator |
| *Xoo2537* | 0.28 | I | 3-hydroxyacyl-CoA dehydrogenase |
| *Xoo2538* | 0.29 | I | Catalyzes the synthesis of acetoacetyl coenzyme A from two molecules of acetyl coenzyme A. It can also act as a thiolase, catalyzing the reverse reaction and generating two-carbon units from the four-carbon product of fatty acid oxidation\|acetyl-CoA acetyltransferase |
| *Xoo2689* | 0.31 | R | general stress protein |
| *Xoo2729* | 0.46 | S | hypothetical protein |
| *Xoo2760* | 0.31 | C | ubiquinol cytochrome C oxidoreductase, cytochrome C1 subunit |
| *Xoo2761* | 0.22 | C | ubiquinol cytochrome C oxidoreductase, cytochrome B subunit |
| *Xoo2762* | 0.34 | C | ubiquinol cytochrome C oxidoreductase, iron-sulfur subunit |
| *Xoo2797* | 0.43 | T | two-component system sensor protein |
| *Xoo2798* | 0.43 | T | two-component system regulatory protein |
| *Xoo2826* | 0.28 | P | TonB-dependent receptor |
| *Xoo2909* | 0.42 | G | MFS transporter |
| *Xoo2912* | 0.20 | S | hypothetical protein |
| *Xoo2913* | 0.21 | S | hypothetical protein |
| *Xoo2918* | 0.49 | G | regucalcin |
| *Xoo2973* | 0.48 | S | hypothetical protein |
| *Xoo3118* | 0.34 | L | IS1113 transposase |
| *Xoo3129* | 0.32 | G | hypothetical protein |
| *Xoo3149* | 0.31 | L | IS1478 transposase |
| *Xoo3161* | 0.37 | M | outer membrane lipoprotein Blc |
| *Xoo3164* | 0.21 | L | transposase |
| *Xoo3199* | 0.36 | U | protein PilX |
| *Xoo3200* | 0.40 | U | hypothetical protein |
| *Xoo3201* | 0.42 | U | pre-pilin leader sequence |
| *Xoo3202* | 0.40 | U | pre-pilin like leader sequence |
| *Xoo3323* | 0.35 | S | hypothetical protein |
| *Xoo3347* | 0.34 | L | transposase |
| *Xoo3349* | 0.41 | G | glycogen debranching protein |
| *Xoo3370* | 0.13 | IN | lipase/esterase |
| *Xoo3402* | 0.48 | S | hypothetical protein |
| *Xoo3423* | 0.15 | P | catalase |
| *Xoo3424* | 0.44 | S | RSp1239\|hypothetical protein |
| *Xoo3433* | 0.41 | E | alanyl dipeptidyl peptidase |
| *Xoo3435* | 0.35 | S | oligopeptide transporter |
| *Xoo3684* | 0.41 | C | converts threonine and NAD to 1,2-amino-3-oxobutanoate and NADH; functions in threonine catabolism\|L-threonine 3-dehydrogenase |
| *Xoo3685* | 0.42 | S | hypothetical protein |
| *Xoo3759* | 0.46 | E | hypothetical protein |
| *Xoo3780* | 0.06 |  | outer membrane hemin receptor |
| *Xoo3781* | 0.19 | S | hypothetical protein |
| *Xoo3799* | 0.43 | R | methyltransferase |
| *Xoo3800* | 0.42 | S | sulfotransferase |
| *Xoo3864* | 0.35 |  | hypothetical protein |
| *Xoo3865* | 0.33 | S | hypothetical protein |
| *Xoo3937* | 0.22 | R | hypothetical protein |
| *Xoo4019* | 0.38 | G | cellulase |
| *Xoo4071* | 0.35 | E | 4-hydroxyphenylpyruvate dioxygenase |
| *Xoo4096* | 0.18 | S | hypothetical protein |
| *Xoo4113* | 0.11 | S | hypothetical protein |
| *Xoo4115* | 0.36 | G | dehydrogenase |
| *Xoo4136* | 0.12 | S | hypothetical protein |
| *Xoo4137* | 0.19 | C | cytochrome C oxidase subunit III |
| *Xoo4138* | 0.27 | O | involved in the insertion of copper into subunit I of cytochrome C oxidase\|cytochrome C oxidase assembly protein |
| *Xoo4139* | 0.42 |  | hypothetical protein |
| *Xoo4140* | 0.15 | C | cytochrome C oxidase subunit I |
| *Xoo4141* | 0.32 | C | cytochrome C oxidase subunit II |
| *Xoo4142* | 0.41 |  | hypothetical protein |
| *Xoo4145* | 0.46 | E | proline utilization protein A; multifunctional protein that functions in proline catabolism in the first two enzymatic steps resulting in the conversion of proline to glutamate; in Escherichia coli this protein self regulates transcription via a DNA-binding domain at the N-terminus but the proteins from this group do not and in addition appear to have a truncated C-terminal domain\|bifunctional proline dehydrogenase/pyrroline-5-carboxylate dehydrogenase |
| *Xoo4150* | 0.42 | P | bacterioferritin-associated ferredoxin |
| *Xoo4217* | 0.23 | S | hypothetical protein |
| *Xoo4219* | 0.44 | K | MarR family transcriptional regulator |
| *Xoo4275* | 0.46 | S | hypothetical protein |
| *Xoo4276* | 0.31 | G | gluconolactonase |
| *Xoo4376* | 0.40 | E | dipeptidyl anminopeptidase |
| *Xoo4377* | 0.05 | I | biotin carboxylase |
| *Xoo4378* | 0.05 | I | acyl-CoA carboxyltransferase subunit beta |
| *Xoo4379* | 0.06 | I | acyl-CoA dehydrogenase |
| *Xoo4380* | 0.31 | K | AcrR family transcriptional regulator |
| *Xoo4396* | 0.25 | P | TonB-dependent receptor |
| *Xoo4431* | 0.39 | P | TonB-dependent receptor |
| *Xoo4432* | 0.48 | G | transporter |
| *Xoo4433* | 0.42 | G | xylosidase |
| *Xoo4446* | 0.40 | I | hypothetical protein |
| *Xoo4447* | 0.46 | C | oxidoreductase |
| *Xoo4481* | 0.50 | P | superoxide dismutase |
| *Xoo4499* | 0.50 | S | hypothetical protein |
| *Xoo4503* | 0.36 |  | hypothetical protein |
| *Xoo4508* | 0.39 | S | hypothetical protein |
| *Xoo4509* | 0.40 | S | hypothetical protein |
| *Xoo4535* | 0.14 | M | RTS beta protein |
| *Xoo4536* | 0.11 | Q | 2-hydroxyhepta-2,4-diene-1, 7-dioate isomerase |
| *Xoo4537* | 0.16 | IQ | oxidoreductase |
| *Xoo4538* | 0.16 | R | hypothetical protein |
| *Xoo4539* | 0.17 | C | oxidoreductase |
| *Xoo4543* | 0.32 | T | two-component system regulatory protein |
| *Xoo4544* | 0.37 | I | Acs; catalyzes the conversion of acetate and CoA to acetyl-CoA\|acetyl-CoA synthetase |
| *Xoo4545* | 0.13 | S | hypothetical protein |
| *Xoo4546* | 0.07 | S | hypothetical protein |
| *Xoo4637* | 0.46 | J | protein component of RNaseP which catalyzes the removal of the 5'-leader sequence from pre-tRNA to produce the mature 5'terminus; this enzyme also cleaves other RNA substrates\|ribonuclease P |
| *Xoo4759* | 0.39 | S | hypothetical protein |
| *Xoo4761* | 0.39 |  | hypothetical protein |
| *Xoo4790* | 0.31 |  | hypothetical protein |
| **60 min upregulated** | | | |
| *Xoo0135* | 2.11 | C | dihydrolipoamide acyltransferase |
| *Xoo0163* | 2.34 | S | hypothetical protein |
| *Xoo0177* | 2.60 | E | catalyzes the formation of glutamate from glutamine and alpha-ketoglutarate\|glutamate synthase subunit alpha |
| *Xoo0178* | 2.44 | E | glutamate synthase is composed of subunits alpha and beta; beta subunit is a flavin adenine dinucleotide-NADPH dependent oxidoreductase; provides electrons to the alpha subunit, which binds L-glutamine and 2-oxoglutarate and forms L-glutamate\|glutamate synthase subunit beta |
| *Xoo0222* | 3.26 | S | Vgr-related protein |
| *Xoo0223* | 2.38 | S | hypothetical protein |
| *Xoo0279* | 2.38 | L | ISXo8 transposase |
| *Xoo0280* | 3.00 | C | alcohol dehydrogenase |
| *Xoo0281* | 2.05 | G | cellulase |
| *Xoo0297* | 2.75 | L | transposase |
| *Xoo0337* | 2.90 | S | hypothetical protein |
| *Xoo0405* | 2.04 | K | LysR family transcriptional regulator |
| *Xoo0417* | 2.73 | P | catalase |
| *Xoo0418* | 2.45 | R | ankyrin-like protein |
| *Xoo0419* | 2.67 |  | hypothetical protein |
| *Xoo0420* | 2.73 |  | hypothetical protein |
| *Xoo0432* | 2.01 | R | hypothetical protein |
| *Xoo0594* | 2.09 | H | catalyzes the formation of formate and 2,5-diamino-6-hydroxy-4-(5-phosphoribosylamino)pyrimidine from GTP\|GTP cyclohydrolase |
| *Xoo0637* | 2.05 | L | transposase |
| *Xoo0664* | 3.50 | C | oxidoreductase |
| *Xoo0665* | 4.74 | R | tryptophan repressor binding protein |
| *Xoo0674* | 2.15 | L | transposase |
| *Xoo0675* | 2.36 | P | Na+:H+ antiporter |
| *Xoo0688* | 2.03 | N | With MotB forms the ion channels that couple flagellar rotation to proton/sodium motive force across the membrane and forms the stator elements of the rotary flagellar machine\|flagellar motor protein MotA |
| *Xoo0691* | 2.20 |  | hypothetical protein |
| *Xoo0748* | 6.58 | S | hypothetical protein |
| *Xoo0749* | 2.89 | L | transposase |
| *Xoo0753* | 2.15 | L | transposase |
| *Xoo0768* | 7.82 |  | hypothetical protein |
| *Xoo0770* | 4.28 | L | ATP-dependent RNA helicase |
| *Xoo0786* | 2.88 | L | IS1478 transposase |
| *Xoo0875* | 2.58 | L | transposase |
| *Xoo0901* | 2.72 | P | TonB-dependent receptor |
| *Xoo0909* | 2.45 | O | low molecular weight heat shock protein |
| *Xoo0929* | 2.53 | K | transcriptional regulator |
| *Xoo0930* | 2.38 | T | hypothetical protein |
| *Xoo1006* | 2.01 | L | transposase |
| *Xoo1047* | 3.60 | S | Vgr-related protein |
| *Xoo1048* | 2.01 | S | hypothetical protein |
| *Xoo1090* | 2.10 | SPO | glutaredoxin-like protein |
| *Xoo1102* | 2.40 | IQ | catalyzes the first of the two reduction steps in the elongation cycle of fatty acid synthesis\|3-ketoacyl-ACP reductase |
| *Xoo1103* | 3.59 | C | Mg++/citrate complex transporter |
| *Xoo1104* | 5.35 | P | porin |
| *Xoo1117* | 5.82 | C | involved in the transport of C4-dicarboxylates across the membrane\|C4-dicarboxylate transporter DctA |
| *Xoo1184* | 2.27 | SI | long-chain acyl-CoA synthetase |
| *Xoo1186* | 2.67 | R | Avin0576\|short chain dehydrogenase |
| *Xoo1204* | 2.01 | L | transposase |
| *Xoo1235* | 2.40 | L | Avin3395\|transposase |
| *Xoo1259* | 2.25 | E | peptidase |
| *Xoo1273* | 4.74 | C | quinol oxidase subunit I |
| *Xoo1274* | 3.04 | C | quinol oxidase subunit II |
| *Xoo1309* | 4.41 | L | DNA transport competence protein |
| *Xoo1376* | 4.26 | S | hypothetical protein |
| *Xoo1400* | 2.84 | C | benzene 1,2-dioxygenase ferredoxin protein |
| *Xoo1401* | 2.90 | K | acetyltransferase |
| *Xoo1402* | 2.45 | E | cysteine desulfurase |
| *Xoo1403* | 2.55 | O | ABC transporter permease |
| *Xoo1404* | 2.25 | O | ABC transporter ATP-binding protein |
| *Xoo1405* | 2.30 | O | with SufCD activates cysteine desulfurase SufS\|cysteine desulfurase |
| *Xoo1406* | 2.11 | K | hypothetical protein |
| *Xoo1412* | 4.66 |  | hypothetical protein |
| *Xoo1448* | 5.30 | R | pirin |
| *Xoo1449* | 9.00 | O | hypothetical protein |
| *Xoo1454* | 2.07 | S | hypothetical protein |
| *Xoo1471* | 2.51 | S | hypothetical protein |
| *Xoo1502* | 2.43 | R | nitrilase |
| *Xoo1532* | 2.08 | P | outer membrane hemin receptor |
| *Xoo1551* | 2.16 | T | hypothetical protein |
| *Xoo1568* | 2.15 | L | transposase |
| *Xoo1629* | 2.08 | C | cytochrome O ubiquinol oxidase subunit II |
| *Xoo1637* | 2.15 | L | transposase |
| *Xoo1677* | 2.01 | L | transposase |
| *Xoo1688* | 2.23 | K | Z2969\|hypothetical protein |
| *Xoo1700* | 2.39 | R | J\|phage-related baseplate assembly protein |
| *Xoo1712* | 2.37 | R | N\|phage-related major capsid protein |
| *Xoo1803* | 7.98 | R | hypothetical protein |
| *Xoo1817* | 2.66 | E | catalyzes the formation of O-acetyl -L-homoserine from L-homoserine and acetyl-CoA\|homoserine O-acetyltransferase |
| *Xoo1818* | 2.03 | E | catalyzes the formation of cystathionine from L-cysteine and O-succinyl-L-homoserine\|cystathionine gamma-synthase |
| *Xoo1820* | 2.15 | E | homoserine dehydrogenase |
| *Xoo1824* | 3.03 | S | hypothetical protein |
| *Xoo1825* | 3.17 | C | cytochrome B561 |
| *Xoo1826* | 2.72 | S | hypothetical protein |
| *Xoo1833* | 5.03 | L | hypothetical protein |
| *Xoo1899* | 2.29 |  | hypothetical protein |
| *Xoo1900* | 2.73 | C | GTN reductase |
| *Xoo1901* | 28.09 | G | MFS transporter |
| *Xoo1951* | 2.24 | S | hypothetical protein |
| *Xoo1952* | 2.84 | I | PHB depolymerase |
| *Xoo1978* | 2.95 | NU | pili assembly chaperone |
| *Xoo1998* | 4.85 | K | MarR family transcriptional regulator |
| *Xoo1999* | 5.09 | M | outer membrane efflux protein |
| *Xoo2000* | 4.42 | V | multidrug resistance efflux pump |
| *Xoo2001* | 4.56 | P | multidrug resistance membrane translocase |
| *Xoo2012* | 7.04 | L | Reut4275\|IS30 family transposase |
| *Xoo2035* | 2.83 | L | ISXo8 transposase |
| *Xoo2074* | 2.22 | E | 5-methyltetrahydrofolate--homocysteinemethyltransferase |
| *Xoo2075* | 3.24 | KQ | transcriptional regulator |
| *Xoo2076* | 3.94 | I | acyl-CoA dehydrogenase |
| *Xoo2102* | 2.37 | L | ISXo8 transposase |
| *Xoo2108* | 2.15 | L | transposase |
| *Xoo2111* | 2.03 | L | ISXo8 transposase |
| *Xoo2122* | 6.18 | L | transposase |
| *Xoo2123* | 6.13 | I | ACP phosphodieterase |
| *Xoo2124* | 2.07 | K | transcriptional regulator |
| *Xoo2132* | 2.02 | L | ISXo8 transposase |
| *Xoo2133* | 2.32 | R | hypothetical protein |
| *Xoo2228* | 2.12 | T | two-component system sensor protein |
| *Xoo2231* | 2.45 | G | Prop transport protein |
| *Xoo2254* | 3.12 | S | hypothetical protein |
| *Xoo2255* | 2.35 | E | long form of enzyme; catalyzes the formation of N'-5'-phosphoribosyl-ATP from phosphoribosyl pyrophosphate; crucial role in histidine biosynthesis; forms active dimers and inactive hexamers which is dependent on concentration of substrates and inhibitors\|ATP phosphoribosyltransferase |
| *Xoo2256* | 2.22 | E | catalyzes the oxidation of L-histidinol to L-histidinaldehyde and then to L-histidine in histidine biosynthesis; functions as a dimer\|histidinol dehydrogenase |
| *Xoo2257* | 2.13 | E | catalyzes the formation of L-histidinol phosphate from imidazole-acetol phosphate and glutamate in histidine biosynthesis\|histidinol-phosphate aminotransferase |
| *Xoo2258* | 2.01 | E | catalyzes the formation of 3-(imidazol-4-yl)-2-oxopropyl phosphate from D-ethythro-1-(imidazol-4-yl)glycerol 3-phosphate and histidinol from histidinol phosphate\|imidazole glycerol-phosphate dehydratase/histidinol phosphatase |
| *Xoo2260* | 2.32 | E | catalyzes the formation of 5-(5-phospho-1-deoxyribulos-1-ylamino)methylideneamino-l- (5-phosphoribosyl)imidazole-4-carboxamide from 1-(5-phosphoribosyl)-5-[(5- phosphoribosylamino)methylideneamino] imidazole-4-carboxamide\|1-(5-phosphoribosyl)-5-[(5-phosphoribosylamino)methylideneamino]imidazole-4-carboxamide isomerase |
| *Xoo2281* | 2.17 | L | IS163, transposase |
| *Xoo2348* | 2.18 |  | hypothetical protein |
| *Xoo2354* | 2.30 | T | hypothetical protein |
| *Xoo2364* | 2.07 | H | catalyzes the formation of tetrahydrofolate and 2-dehydropantoate from 5,10-methylenetetrahydrofolate and 3-methyl-2-oxobutanoate\|3-methyl-2-oxobutanoatehydroxymethyltransferase |
| *Xoo2375* | 9.39 | O | serine protease |
| *Xoo2376* | 3.93 | P | cation efflux system protein |
| *Xoo2377* | 2.86 | P | cation efflux system protein |
| *Xoo2493* | 2.25 | L | ISXo8 transposase |
| *Xoo2496* | 2.13 | L | IS1478 transposase |
| *Xoo2498* | 2.01 |  | hypothetical protein |
| *Xoo2502* | 2.01 | L | transposase |
| *Xoo2558* | 2.34 | S | chemotaxis protein |
| *Xoo2561* | 2.10 | T | hypothetical protein |
| *Xoo2568* | 2.20 | NT | chemotaxis protein |
| *Xoo2587* | 2.16 | K | RNA polymerase sigma-54 factor |
| *Xoo2627* | 3.83 | L | Reut4275\|IS30 family transposase |
| *Xoo2713* | 2.07 | F | ribonuclease |
| *Xoo2726* | 2.03 | O | metalloprotease\|heat shock protein HtpX |
| *Xoo2745* | 3.57 | L | RSp1628\|hypothetical protein |
| *Xoo2778* | 2.31 | M | outer membrane protein OprN |
| *Xoo2779* | 2.18 | P | transporter |
| *Xoo2780* | 2.10 | V | transporter |
| *Xoo2787* | 2.35 | T | transcriptional regulator |
| *Xoo2792* | 2.15 | L | transposase |
| *Xoo2799* | 3.18 | P | drug resistance translocase |
| *Xoo2803* | 2.09 | K | TetR/AcrR family transcriptional regulator |
| *Xoo2804* | 2.03 | M | multidrug resistance protein |
| *Xoo2809* | 2.01 | K | LacI family transcriptional regulator |
| *Xoo2832* | 2.15 | D | chromosome partioning protein |
| *Xoo2835* | 2.01 | T | chemotaxis response regulator |
| *Xoo2836* | 2.04 | T | chemotaxis protein |
| *Xoo2847* | 3.26 | NT | chemotaxis protein |
| *Xoo2849* | 2.35 | M | hypothetical protein |
| *Xoo2850* | 2.64 | NT | chemotaxis protein |
| *Xoo2857* | 2.40 | NT | chemotaxis protein methyltransferase |
| *Xoo2866* | 2.62 | S | hypothetical protein |
| *Xoo2896* | 2.27 |  | hypothetical protein |
| *Xoo2931* | 3.36 | SP | hypothetical protein |
| *Xoo2933* | 2.50 | R | hypothetical protein |
| *Xoo2979* | 3.04 | C | hypothetical protein |
| *Xoo3126* | 2.33 | N | hypothetical protein |
| *Xoo3157* | 2.44 | L | transposase |
| *Xoo3294* | 4.99 | R | hypothetical protein |
| *Xoo3295* | 3.03 | C | glutamate symporter |
| *Xoo3310* | 2.38 | L | ISXo8 transposase |
| *Xoo3337* | 2.01 | L | transposase |
| *Xoo3351* | 2.44 | L | transposase |
| *Xoo3364* | 5.33 |  | hypothetical protein |
| *Xoo3389* | 6.05 |  | acetyltransferase |
| *Xoo3400* | 3.29 | C | hemoprotein; NADPH dependent; with the alpha subunit (a flavoprotein) catalyzes the reduction of sulfite to sulfide\|sulfite reductase subunit beta |
| *Xoo3408* | 2.41 | H | siroheme synthase |
| *Xoo3428* | 3.14 |  | hypothetical protein |
| *Xoo3445* | 4.72 | L | transposase |
| *Xoo3493* | 2.03 | S | RSp74\|hypothetical protein |
| *Xoo3503* | 2.36 | S | ORF15\|VGR-related protein |
| *Xoo3552* | 2.04 | L | IS1404 transposase |
| *Xoo3612* | 2.37 |  | hypothetical protein |
| *Xoo3645* | 3.39 | O | alkyl hydroperoxide reductase |
| *Xoo3646* | 4.03 | O | alkyl hydroperoxide reductase |
| *Xoo3647* | 3.06 | K | oxidative stress transcriptional regulator |
| *Xoo3659* | 3.26 | T | two-component system regulatory protein |
| *Xoo3666* | 2.01 | T | two-component system regulatory protein |
| *Xoo3698* | 2.94 | C | 2,4-dienoyl-CoA reductase |
| *Xoo3699* | 2.16 |  | hypothetical protein |
| *Xoo3708* | 2.44 | L | transposase |
| *Xoo3716* | 2.01 | L | transposase |
| *Xoo3718* | 2.05 | L | transposase |
| *Xoo3795* | 2.17 | K | TetR/AcrR family transcriptional regulator |
| *Xoo3809* | 2.50 | S | hypothetical protein |
| *Xoo3810* | 2.04 | E | 5,10-methylenetetrahydrofolate reductase |
| *Xoo3811* | 3.10 |  | hypothetical protein |
| *Xoo3855* | 3.07 |  | hypothetical protein |
| *Xoo3874* | 2.26 | L | ISXo8 transposase |
| *Xoo3909* | 2.08 | P | TonB-dependent receptor |
| *Xoo3928* | 2.38 | L | transposase |
| *Xoo3939* | 3.14 | G | similar to full-length Gnd, these proteins seems to have a truncated C-terminal 6PGD domainin; in Methylobacillus flagellatus this gene is essential for NAD+-dependent oxidation of 6-phosphogluconate\|6-phosphogluconate dehydrogenase |
| *Xoo3977* | 2.41 | R | hypothetical protein |
| *Xoo4001* | 2.18 | F | hypothetical protein |
| *Xoo4006* | 2.14 | M | hypothetical protein |
| *Xoo4026* | 2.01 | L | transposase |
| *Xoo4057* | 2.15 | L | transposase |
| *Xoo4104* | 2.05 | L | transposase |
| *Xoo4107* | 2.01 | L | transposase |
| *Xoo4119* | 2.01 | L | transposase |
| *Xoo4220* | 2.25 | T | hypothetical protein |
| *Xoo4227* | 2.15 | L | transposase |
| *Xoo4233* | 2.20 | L | IS1478 transposase |
| *Xoo4266* | 2.23 | S | hypothetical protein |
| *Xoo4267* | 3.53 | C | hypothetical protein |
| *Xoo4290* | 2.20 | L | ISxac1 transposase |
| *Xoo4294* | 2.42 | C | NADH dehydrogenase |
| *Xoo4345* | 2.85 | I | dehydrogenase |
| *Xoo4346* | 3.08 | C | FMN oxidoreductase |
| *Xoo4349* | 2.42 | G | MFS transporter |
| *Xoo4385* | 3.23 | S | hypothetical protein |
| *Xoo4455* | 2.79 | K | MarR family transcriptional regulator |
| *Xoo4456* | 3.08 | O | organic hydroperoxide resistance protein |
| *Xoo4463* | 2.00 | S | Psyr4406\|hypothetical protein |
| *Xoo4472* | 2.11 | S | hypothetical protein |
| *Xoo4475* | 2.13 | Q | hemagglutinin |
| *Xoo4476* | 2.33 | S | microcystin dependent protein |
| *Xoo4489* | 2.01 | V | BacA; phosphatase activity in Escherichia coli not kinase; involved in bacitracin resistance as bacitracin supposedly sequesters undecaprenyl disphosphate which reduces the pool of lipid carrier available to the cell\|undecaprenyl pyrophosphate phosphatase |
| *Xoo4493* | 2.53 | S | hypothetical protein |
| *Xoo4555* | 2.28 | P | alkaline phosphatase |
| *Xoo4579* | 2.01 | L | transposase |
| *Xoo4718* | 2.47 |  | hypothetical protein |
| *Xoo4721* | 2.45 |  | hypothetical protein |
| *Xoo4766* | 4.93 |  | hypothetical protein |
| *Xoo4823* | 2.59 |  | hypothetical protein |
| *Xoo4922* | 2.58 | S | identified by sequence similarity; ORF located using Blastx/FrameD\|hypothetical protein |
| **60 min downregulated** | | | |
| *Xoo0040* | 0.10 | O | thioredoxin |
| *Xoo0041* | 0.12 | N | AtsE |
| *Xoo0050* | 0.40 |  | hypothetical protein |
| *Xoo0051* | 0.38 |  | hypothetical protein |
| *Xoo0055* | 0.33 |  | hypothetical protein |
| *Xoo0105* | 0.48 |  | hypothetical protein |
| *Xoo0108* | 0.43 | R | ABC transporter substrate binding protein |
| *Xoo0113* | 0.49 | G | catalyzes the transfer of a segment of a 1,4-alpha-D-glucan chain to a primary hydroxy group in a similar glucan chain\|glycogen branching protein |
| *Xoo0114* | 0.44 | G | malto-oligosyltrehalose trehalohydrolase |
| *Xoo0116* | 0.49 | G | malto-oligosyltrehalose synthase |
| *Xoo0197* | 0.34 | G | alpha-amylase |
| *Xoo0248* | 0.32 | S | tryptophan halogenase |
| *Xoo0249* | 0.19 | S | OmpA-like protein |
| *Xoo0250* | 0.43 | S | OmpA-like protein |
| *Xoo0252* | 0.21 | K | LacI family transcriptional regulator |
| *Xoo0311* | 0.41 | S | hypothetical protein |
| *Xoo0318* | 0.36 | L | transposase |
| *Xoo0333* | 0.43 | G | cation symporter |
| *Xoo0334* | 0.39 | G | NdvB protein |
| *Xoo0339* | 0.11 | G | fucose permease |
| *Xoo0340* | 0.13 | S | hypothetical protein |
| *Xoo0392* | 0.20 | S | Pass1-like protein |
| *Xoo0393* | 0.18 | S | protein SapC protein |
| *Xoo0394* | 0.03 | P | TonB-dependent receptor |
| *Xoo0436* | 0.23 | S | hypothetical protein |
| *Xoo0467* | 0.46 | G | catalyzes the formation of D-glucono-1,5-lactone 6-phosphate from D-glucose 6-phosphate\|glucose-6-phosphate 1-dehydrogenase |
| *Xoo0486* | 0.44 | S | 4-carboxymuconolactone decarboxylase |
| *Xoo0556* | 0.35 | I | an AccC homodimer forms the biotin carboxylase subunit of the acetyl CoA carboxylase, an enzyme that catalyzes the formation of malonyl-CoA, which in turn controls the rate of fatty acid metabolism\|acetyl-CoA carboxylase biotin carboxylase subunit |
| *Xoo0557* | 0.35 | S | crystal structure of protein from Xanthomonas shows pentameric toroidal structure; physiological function is unknown\|hypothetical protein |
| *Xoo0558* | 0.43 | I | composes the biotin carboxyl carrier protein subunit of the acetyl-CoA carboxylase complex, the enzyme that catalyzes the carboxylation of acetyl-CoA to malonyl-CoA, which in turn controls the rate of fatty acid metabolism\|acetyl-CoA carboxylase biotin carboxyl carrier protein subunit |
| *Xoo0643* | 0.44 | T | regulatory protein |
| *Xoo0656* | 0.42 | T | transcriptional regulator |
| *Xoo0660* | 0.50 |  | hypothetical protein |
| *Xoo0671* | 0.22 | M | hypothetical protein |
| *Xoo0698* | 0.49 | T | sensor histidine kinase |
| *Xoo0699* | 0.33 |  | hypothetical protein |
| *Xoo0700* | 0.35 | G | L-sorbosone dehydrogenase |
| *Xoo0701* | 0.21 |  | hypothetical protein |
| *Xoo0708* | 0.46 | R | hypothetical protein |
| *Xoo0761* | 0.29 | I | beta-ketoacyl- synthase I |
| *Xoo0762* | 0.24 | I | catalyzes the dehydration of (3R)-3-hydroxydecanoyl-ACP to 2,3-decenoyl-ACP or 3,4-decenoyl-ACP\|3-hydroxydecanoyl-ACP dehydratase |
| *Xoo0782* | 0.44 | I | o-antigen acetylase |
| *Xoo0842* | 0.44 | UW | hypothetical protein |
| *Xoo0880* | 0.38 | I | ACP S-malonyltransferase |
| *Xoo0881* | 0.29 | IQ | Catalyzes the first of the two reduction steps in the elongation cycle of fatty acid synthesis\|3-ketoacyl-ACP reductase |
| *Xoo0892* | 0.13 | G | catalyzes the formation of pyruvate and succinate from 2-methylisocitrate\|2-methylisocitrate lyase |
| *Xoo0893* | 0.31 | C | catalyzes the synthesis of 2-methylcitrate from propionyl-CoA and oxaloacetate; also catalyzes the condensation of oxaloacetate with acetyl-CoA but with a lower specificity\|methylcitrate synthase |
| *Xoo0894* | 0.34 | C | catalyzes the conversion of citrate to isocitrate\|aconitate hydratase |
| *Xoo0895* | 0.27 |  | hypothetical protein |
| *Xoo0947* | 0.32 | H | required for the synthesis of the hydromethylpyrimidine moiety of thiamine\|thiamine biosynthesis protein ThiC |
| *Xoo0990* | 0.17 | L | transposase |
| *Xoo1097* | 0.35 | R | NonF-like protein |
| *Xoo1098* | 0.41 | G | amylosucrase or alpha amylase |
| *Xoo1099* | 0.29 | P | TonB-dependent receptor |
| *Xoo1100* | 0.22 | G | sugar transporter |
| *Xoo1188* | 0.40 | T | response regulator |
| *Xoo1189* | 0.38 | T | Avin0574\|Signal transduction histidine kinase |
| *Xoo1240* | 0.43 | L | Reut4275\|IS30 family transposase |
| *Xoo1261* | 0.26 | S | hypothetical protein |
| *Xoo1262* | 0.28 | K | hypothetical protein |
| *Xoo1263* | 0.21 | S | tryptophan halogenase |
| *Xoo1264* | 0.49 | R | hydrolase |
| *Xoo1354* | 0.33 | E | diaminopimelate decarboxylase |
| *Xoo1355* | 0.27 | Q | iron transporter |
| *Xoo1356* | 0.34 | P | transporter |
| *Xoo1357* | 0.25 | Q | hypothetical protein |
| *Xoo1358* | 0.21 | I | hypothetical protein |
| *Xoo1388* | 0.39 | E | catalyzes the formation of O-succinyl-L-homoserine from succinyl-CoA and L-homoserine in methionine biosynthesis\|homoserine O-succinyltransferase |
| *Xoo1389* | 0.47 | I | protein RtxC |
| *Xoo1391* | 0.32 | E | hypothetical protein |
| *Xoo1428* | 0.40 | O | osmotically inducible protein |
| *Xoo1524* | 0.39 | R | hypothetical protein |
| *Xoo1530* | 0.20 | P | TonB-dependent receptor |
| *Xoo1601* | 0.36 | G | trehalose-6-phosphate synthase |
| *Xoo1602* | 0.42 | G | hypothetical protein |
| *Xoo1636* | 0.47 | M | hypothetical protein |
| *Xoo1686* | 0.43 |  | hypothetical protein |
| *Xoo1733* | 0.42 | R | pyrroloquinoline quinone biosynthesis protein PqqE |
| *Xoo1734* | 0.33 | H | Required in the synthesis of PPQ, but its exact function is unknown\|pyrroloquinoline quinone biosynthesis protein PqqC |
| *Xoo1735* | 0.37 | R | possibly involved in transport of pyrroloquinoline quinone transport\|pyrroloquinoline quinone biosynthesis protein PqqB |
| *Xoo1769* | 0.09 | G | beta-galactosidase |
| *Xoo1770* | 0.09 | G | hypothetical protein |
| *Xoo1771* | 0.09 | S | hypothetical protein |
| *Xoo1779* | 0.42 | G | glucan 1,4-beta-glucosidase |
| *Xoo1780* | 0.38 | G | beta-mannosidase |
| *Xoo1781* | 0.45 | G | beta-hexosaminidase |
| *Xoo1782* | 0.40 | G | hypothetical protein |
| *Xoo1783* | 0.35 | G | alpha-L-fucosidase |
| *Xoo1784* | 0.35 | P | TonB-dependent receptor |
| *Xoo1821* | 0.43 | E | L-serine dehydratase |
| *Xoo1842* | 0.16 | C | methylmalonate-semialdehyde dehydrogenase |
| *Xoo1843* | 0.12 | I | acyl-CoA dehydrogenase |
| *Xoo1844* | 0.13 | S | Catalyzes the reversible hydration of unsaturated fatty acyl-CoA to beta-hydroxyacyl-CoA\|enoyl-CoA hydratase |
| *Xoo1845* | 0.16 | I | enoyl-CoA hydratase |
| *Xoo1846* | 0.13 | I | 3-hydroxyisobutyrate dehydrogenase |
| *Xoo1874* | 0.33 | L | helicase-like protein |
| *Xoo1875* | 0.40 | L | catalyzes the ATP-dependent formation of a phosphodiester at the site of a single strand break in duplex DNA\|ATP-dependent DNA ligase |
| *Xoo1885* | 0.49 | S | hypothetical protein |
| *Xoo1902* | 0.18 | S | hypothetical protein |
| *Xoo2069* | 0.22 | K | transcriptional regulator |
| *Xoo2070* | 0.10 | G | glucose-galactose transporter |
| *Xoo2071* | 0.11 | G | fructokinase |
| *Xoo2072* | 0.10 | G | hypothetical protein |
| *Xoo2126* | 0.28 | S | integrase |
| *Xoo2158* | 0.35 | L | transposase |
| *Xoo2215* | 0.48 | L | transposase |
| *Xoo2217* | 0.44 |  | transposase |
| *Xoo2223* | 0.31 | R | oxidoreductase |
| *Xoo2301* | 0.21 | L | transposase |
| *Xoo2351* | 0.07 | E | hypothetical protein |
| *Xoo2352* | 0.05 | G | beta-glucosidase |
| *Xoo2355* | 0.29 | R | sodium/glucose cotransport protein |
| *Xoo2356* | 0.30 | G | glucan 1,4-beta-glucosidase |
| *Xoo2382* | 0.39 | L | transposase |
| *Xoo2383* | 0.20 | S | hypothetical protein |
| *Xoo2411* | 0.27 | E | aminotransferase |
| *Xoo2507* | 0.40 |  | hypothetical protein |
| *Xoo2519* | 0.28 | L | Avin0470\|transposase |
| *Xoo2537* | 0.38 | I | 3-hydroxyacyl-CoA dehydrogenase |
| *Xoo2538* | 0.40 | I | Catalyzes the synthesis of acetoacetyl coenzyme A from two molecules of acetyl coenzyme A. It can also act as a thiolase, catalyzing the reverse reaction and generating two-carbon units from the four-carbon product of fatty acid oxidation\|acetyl-CoA acetyltransferase |
| *Xoo2647* | 0.45 |  | hypothetical protein |
| *Xoo2689* | 0.27 | R | general stress protein |
| *Xoo2729* | 0.39 | S | hypothetical protein |
| *Xoo2760* | 0.26 | C | ubiquinol cytochrome C oxidoreductase, cytochrome C1 subunit |
| *Xoo2761* | 0.20 | C | ubiquinol cytochrome C oxidoreductase, cytochrome B subunit |
| *Xoo2762* | 0.33 | C | ubiquinol cytochrome C oxidoreductase, iron-sulfur subunit |
| *Xoo2797* | 0.42 | T | two-component system sensor protein |
| *Xoo2798* | 0.37 | T | two-component system regulatory protein |
| *Xoo2826* | 0.30 | P | TonB-dependent receptor |
| *Xoo2909* | 0.50 | G | MFS transporter |
| *Xoo2912* | 0.13 | S | hypothetical protein |
| *Xoo2913* | 0.18 | S | hypothetical protein |
| *Xoo3019* | 0.49 | V | hypothetical protein |
| *Xoo3118* | 0.34 | L | IS1113 transposase |
| *Xoo3129* | 0.40 | G | hypothetical protein |
| *Xoo3132* | 0.47 | E | amino acid permease |
| *Xoo3149* | 0.20 | L | IS1478 transposase |
| *Xoo3161* | 0.43 | M | outer membrane lipoprotein Blc |
| *Xoo3199* | 0.35 | U | protein PilX |
| *Xoo3200* | 0.34 | U | hypothetical protein |
| *Xoo3201* | 0.36 | U | pre-pilin leader sequence |
| *Xoo3202* | 0.40 | U | pre-pilin like leader sequence |
| *Xoo3292* | 0.46 | M | hypothetical protein |
| *Xoo3349* | 0.35 | G | glycogen debranching protein |
| *Xoo3370* | 0.14 | IN | lipase/esterase |
| *Xoo3392* | 0.49 | S | hypothetical protein |
| *Xoo3402* | 0.45 | S | hypothetical protein |
| *Xoo3423* | 0.12 | P | catalase |
| *Xoo3424* | 0.28 | S | RSp1239\|hypothetical protein |
| *Xoo3433* | 0.45 | E | alanyl dipeptidyl peptidase |
| *Xoo3435* | 0.32 | S | oligopeptide transporter |
| *Xoo3661* | 0.35 | L | transposase |
| *Xoo3684* | 0.39 | C | converts threonine and NAD to 1,2-amino-3-oxobutanoate and NADH; functions in threonine catabolism\|L-threonine 3-dehydrogenase |
| *Xoo3780* | 0.28 |  | outer membrane hemin receptor |
| *Xoo3799* | 0.45 | R | methyltransferase |
| *Xoo3800* | 0.43 | S | sulfotransferase |
| *Xoo3864* | 0.46 |  | hypothetical protein |
| *Xoo3879* | 0.35 | L | transposase |
| *Xoo3937* | 0.14 | R | hypothetical protein |
| *Xoo4019* | 0.31 | G | cellulase |
| *Xoo4020* | 0.42 | S | chemotaxis protein |
| *Xoo4071* | 0.34 | E | 4-hydroxyphenylpyruvate dioxygenase |
| *Xoo4090* | 0.35 | L | transposase |
| *Xoo4096* | 0.15 | S | hypothetical protein |
| *Xoo4113* | 0.07 | S | hypothetical protein |
| *Xoo4115* | 0.38 | G | dehydrogenase |
| *Xoo4136* | 0.13 | S | hypothetical protein |
| *Xoo4137* | 0.16 | C | cytochrome C oxidase subunit III |
| *Xoo4138* | 0.20 | O | involved in the insertion of copper into subunit I of cytochrome C oxidase\|cytochrome C oxidase assembly protein |
| *Xoo4139* | 0.34 |  | hypothetical protein |
| *Xoo4140* | 0.16 | C | cytochrome C oxidase subunit I |
| *Xoo4141* | 0.35 | C | cytochrome C oxidase subunit II |
| *Xoo4142* | 0.40 |  | hypothetical protein |
| *Xoo4145* | 0.47 | E | proline utilization protein A; multifunctional protein that functions in proline catabolism in the first two enzymatic steps resulting in the conversion of proline to glutamate; in Escherichia coli this protein self regulates transcription via a DNA-binding domain at the N-terminus but the proteins from this group do not and in addition appear to have a truncated C-terminal domain\|bifunctional proline dehydrogenase/pyrroline-5-carboxylate dehydrogenase |
| *Xoo4173* | 0.46 | L | ISxac1 transposase |
| *Xoo4194* | 0.47 | S | hypothetical protein |
| *Xoo4217* | 0.21 | S | hypothetical protein |
| *Xoo4219* | 0.46 | K | MarR family transcriptional regulator |
| *Xoo4275* | 0.41 | S | hypothetical protein |
| *Xoo4276* | 0.32 | G | gluconolactonase |
| *Xoo4376* | 0.44 | E | dipeptidyl anminopeptidase |
| *Xoo4377* | 0.05 | I | biotin carboxylase |
| *Xoo4378* | 0.06 | I | acyl-CoA carboxyltransferase subunit beta |
| *Xoo4379* | 0.08 | I | acyl-CoA dehydrogenase |
| *Xoo4380* | 0.33 | K | AcrR family transcriptional regulator |
| *Xoo4396* | 0.28 | P | TonB-dependent receptor |
| *Xoo4431* | 0.33 | P | TonB-dependent receptor |
| *Xoo4433* | 0.42 | G | xylosidase |
| *Xoo4446* | 0.44 | I | hypothetical protein |
| *Xoo4447* | 0.45 | C | oxidoreductase |
| *Xoo4499* | 0.50 | S | hypothetical protein |
| *Xoo4508* | 0.30 | S | hypothetical protein |
| *Xoo4535* | 0.14 | M | RTS beta protein |
| *Xoo4536* | 0.12 | Q | 2-hydroxyhepta-2,4-diene-1, 7-dioate isomerase |
| *Xoo4537* | 0.19 | IQ | oxidoreductase |
| *Xoo4538* | 0.19 | R | hypothetical protein |
| *Xoo4539* | 0.19 | C | oxidoreductase |
| *Xoo4543* | 0.41 | T | two-component system regulatory protein |
| *Xoo4544* | 0.48 | I | Acs; catalyzes the conversion of acetate and CoA to acetyl-CoA\|acetyl-CoA synthetase |
| *Xoo4545* | 0.15 | S | hypothetical protein |
| *Xoo4546* | 0.11 | S | hypothetical protein |
| *Xoo4547* | 0.50 | S | hypothetical protein |
| *Xoo4713* | 0.36 | S | hypothetical protein |
| *Xoo4723* | 0.40 | L | identified by sequence similarity; ORF located using Blastx/FrameD\|IS1404 transposase |
| *Xoo4790* | 0.38 |  | hypothetical protein |

*FC: fold change (compared with zero time)
