# Supplementary material for: Time-resolved pathogenic gene expression analysis of the plant pathogen Xanthomonas oryzae pv. oryzae
Source: BMC Genomics. 2016 May 10;17:345. doi: 10.1186/s12864-016-2657-7 (PMC4862043; doi:10.1186/s12864-016-2657-7)
Supplement: Additional file 6: Figure S2. — Time-resolved expression of hrp genes including hpaB, hrpE, hrpD6, hrpD5, hpa1 and hpa2. The unit of time is min. Red lines indicate the expression levels of genes in control (untreated) Xoo cells. Y-axis represents fold-change. (PPTX 51 kb) [file 12864_2016_2657_MOESM6_ESM.pptx]

## Slide 1
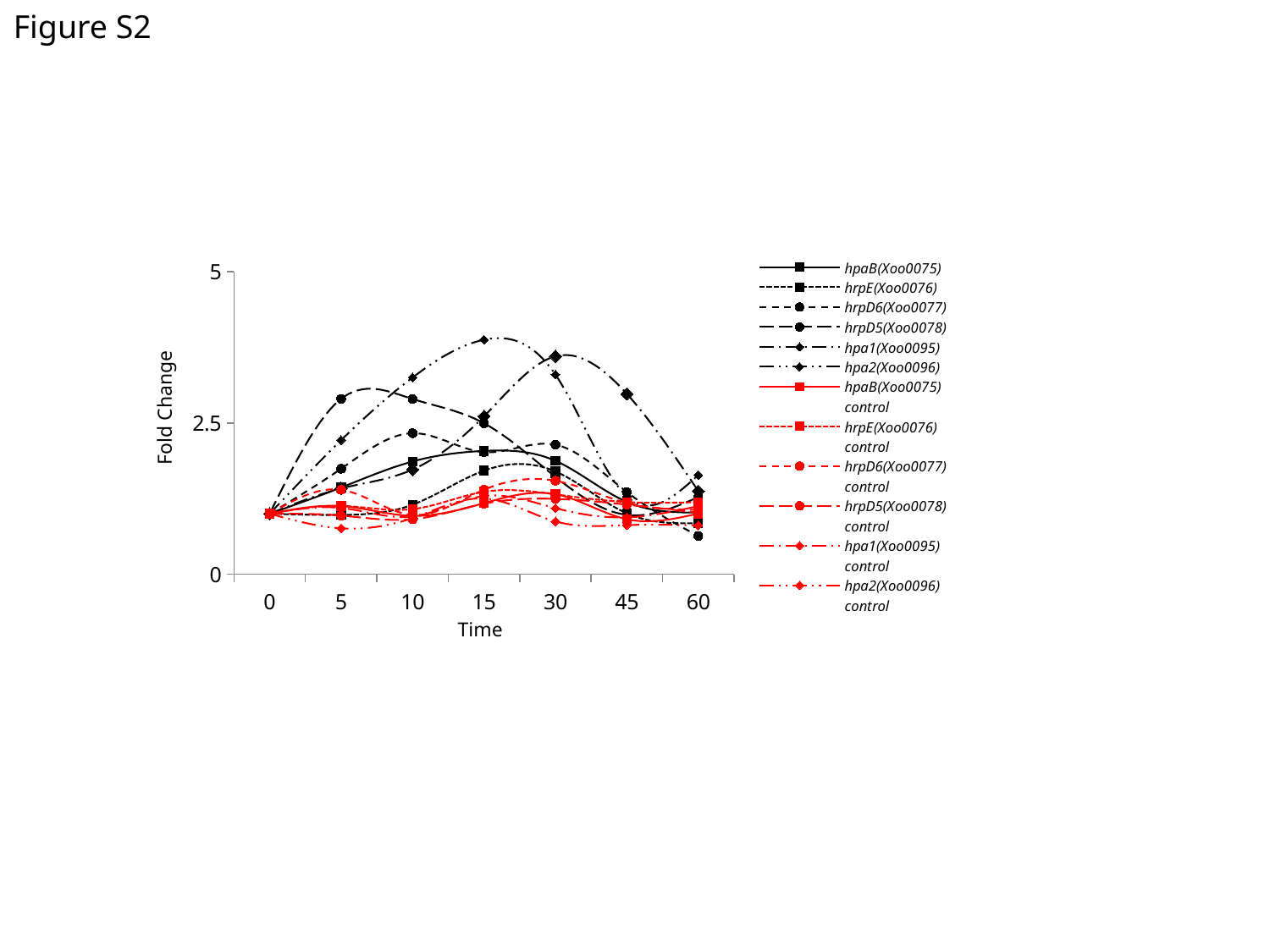

Figure S2
### Chart
| Category | hpaB(Xoo0075) | hrpE(Xoo0076) | hrpD6(Xoo0077) | hrpD5(Xoo0078) | hpa1(Xoo0095) | hpa2(Xoo0096) | hpaB(Xoo0075) control | hrpE(Xoo0076) control | hrpD6(Xoo0077) control | hrpD5(Xoo0078) control | hpa1(Xoo0095) control | hpa2(Xoo0096) control |
|---|---|---|---|---|---|---|---|---|---|---|---|---|
| 0 | 1.0 | 1.0 | 1.0 | 1.0 | 1.0 | 1.0 | 1.0 | 1.0 | 1.0 | 1.0 | 1.0 | 1.0 |
| 5 | 1.433662280701754 | 0.980843366348378 | 1.743259085580305 | 2.897717666948436 | 1.417270929466052 | 2.215579710144928 | 1.126628075253256 | 1.128896497610386 | 1.39511201629328 | 0.969887076537014 | 1.096417281348788 | 0.759856630824373 |
| 10 | 1.862938596491228 | 1.149457327560643 | 2.330597889800703 | 2.895181741335588 | 1.72972972972973 | 3.25 | 0.965267727930535 | 1.075825665168699 | 0.980312287847929 | 0.90840652446675 | 0.9536354056902 | 0.92831541218638 |
| 15 | 2.037280701754386 | 1.711464325959314 | 2.016412661195779 | 2.494505494505494 | 2.609096901779829 | 3.873188405797101 | 1.175108538350217 | 1.362365361295385 | 1.396469789545146 | 1.165621079046424 | 1.292413066385669 | 1.253285543608124 |
| 30 | 1.873355263157895 | 1.697882687859557 | 2.139507620164127 | 1.608622147083686 | 3.600527356624918 | 3.297101449275363 | 1.323444283646888 | 1.319280975818532 | 1.543788187372709 | 1.245294855708908 | 1.092202318229716 | 0.873357228195938 |
| 45 | 1.195175438596492 | 1.006879781744855 | 1.35873388042204 | 0.981403212172443 | 2.98022412656559 | 1.284420289855072 | 0.909551374819103 | 1.193665739353734 | 1.179226069246436 | 1.149937264742786 | 0.94204425711275 | 0.812425328554361 |
| 60 | 1.018640350877193 | 0.843662890694502 | 0.63305978898007 | 1.273879966187658 | 1.373764007910349 | 1.639492753623188 | 1.003617945007236 | 1.190955132320423 | 1.064494229463679 | 1.018193224592221 | 1.126448893572181 | 0.807646356033453 |Fold Change
Time
