# Supplementary material for: Time-resolved pathogenic gene expression analysis of the plant pathogen Xanthomonas oryzae pv. oryzae
Source: BMC Genomics. 2016 May 10;17:345. doi: 10.1186/s12864-016-2657-7 (PMC4862043; doi:10.1186/s12864-016-2657-7)
Supplement: Additional file 7: Table S5. — Fold changes of transcriptional expression levels of hrp genes in Xoo and Xcc from two different in vitro assay systems. (DOCX 19 kb) [file 12864_2016_2657_MOESM7_ESM.docx]

**Table S5.** Fold changes of transcriptional expression levels of *hrp* genes in *Xoo* and *Xcc* from two different *in vitro* assay systems.

| **Gene name** | **Annotation** | **Fold Change** | | | | | | |
| --- | --- | --- | --- | --- | --- | --- | --- | --- |
|  |  | Xoo (control: 0 min)* | | | | | | Xcc (MMX/NYG)** |
|  |  | 5 | 10 | 15 | 30 | 45 | 60 |  |
| *hpa2* | Hpa2 protein | 2.22 | 3.25 | 3.87 | 3.30 | 1.28 | 1.64 | 10.60 |
| *hpa1* | Hpa1 protein | 1.42 | 1.73 | 2.61 | 3.60 | 2.98 | 1.37 | 60.26 |
| *hrcT* | HrpB8 protein | 1.49 | 0.96 | 0.85 | 0.98 | 0.66 | 0.70 | 9.09 |
| *hrpB7* | HrpB7 protein | 1.79 | 1.12 | 1.03 | 0.83 | 0.93 | 0.83 | 7.41 |
| *hrcN* | HrpB6 protein | 1.16 | 0.94 | 0.87 | 0.67 | 0.69 | 0.60 | 13.72 |
| *hrpB5* | HrpB5 protein | 1.45 | 0.93 | 1.19 | 0.90 | 1.05 | 0.73 | 14.97 |
| *hrpB4* | HrpB4 protein | 1.42 | 1.26 | 0.90 | 0.80 | 1.01 | 0.89 | 16.76 |
| *hrpB3* | HrpB3 protein | 1.16 | 0.92 | 0.84 | 0.77 | 0.74 | 0.55 | - |
| *hrpB2* | HrpB2 protein | 1.11 | 1.12 | 0.97 | 0.94 | 0.88 | 0.54 | 15.67 |
| *hrpB1* | HrpB1 protein | 0.94 | 0.72 | 0.71 | 0.68 | 0.70 | 0.64 | 12.14 |
| *hrcU* | HrcU protein | 1.34 | 1.16 | 1.48 | 1.23 | 0.91 | 0.82 | 7.70 |
| *hrcV* | HrcV | 1.64 | 1.57 | 1.54 | 1.25 | 1.04 | 0.84 | 13.04 |
| *hpaP* | HpaP protein | 2.53 | 1.45 | 1.12 | 1.47 | 1.66 | 1.27 | 10.23 |
| *hrcR* | HrcR protein | 1.73 | 1.50 | 1.55 | 1.50 | 0.87 | 0.84 | 9.99 |
| *hpaA* | HpaA protein | 1.46 | 1.22 | 1.21 | 0.97 | 0.67 | 0.76 | 13.44 |
| *hrpD5* | HrpD5 protein | 2.90 | 2.90 | 2.49 | 1.61 | 0.98 | 1.27 | 17.18 |
| *hrpD6* | HrpD6 protein | 1.74 | 2.33 | 2.02 | 2.14 | 1.36 | 0.63 | 12.55 |
| *hrpE* | HrpE protein | 0.98 | 1.15 | 1.71 | 1.70 | 1.01 | 0.84 | 5.77 |
| *hpaB* | HpaB protein | 1.43 | 1.86 | 2.04 | 1.87 | 1.20 | 1.02 | 11.18 |
| *hrpF* | HrpF protein | 1.14 | 1.32 | 1.21 | 0.87 | 0.68 | 0.65 | 5.21 |
| *hrpXct* | HrpX protein | 2.13 | 4.34 | 6.54 | 4.88 | 2.38 | 1.64 | 3.85 |
| *hrpG* | HrpG protein | 3.66 | 4.90 | 4.26 | 2.09 | 1.30 | 1.25 | 2.34 |

* Data from this study

** Data from reference [30]
