# Supplementary material for: Time-resolved pathogenic gene expression analysis of the plant pathogen Xanthomonas oryzae pv. oryzae
Source: BMC Genomics. 2016 May 10;17:345. doi: 10.1186/s12864-016-2657-7 (PMC4862043; doi:10.1186/s12864-016-2657-7)
Supplement: Additional file 8: Figure S3. — Time-resolved expression of genes related to iron-uptake. (A) Among tonB genes, Xoo2114 was upregulated 6-fold. (B) Upregulated TonB-dependent receptor genes; fecA (Xoo0901) and cirA (Xoo3793) were upregulated 80- and 20-fold, respectively. (C) Downregulated TonB-dependent receptor genes; iroN (Xoo0394), fyuA (Xoo1099), iroN (Xoo1784) and cirA (Xoo4431) were downregulated 50-, 5-, 5- and 4-fold, respectively. (D) tolR (Xoo1666, exbD-like) was upregulated 2.5-fold. The unit of time is min. Red lines indicate the expression levels of genes in control (untreated) Xoo cells. Y-axis represents fold-change. (PPTX 84 kb) [file 12864_2016_2657_MOESM8_ESM.pptx]

## Slide 1
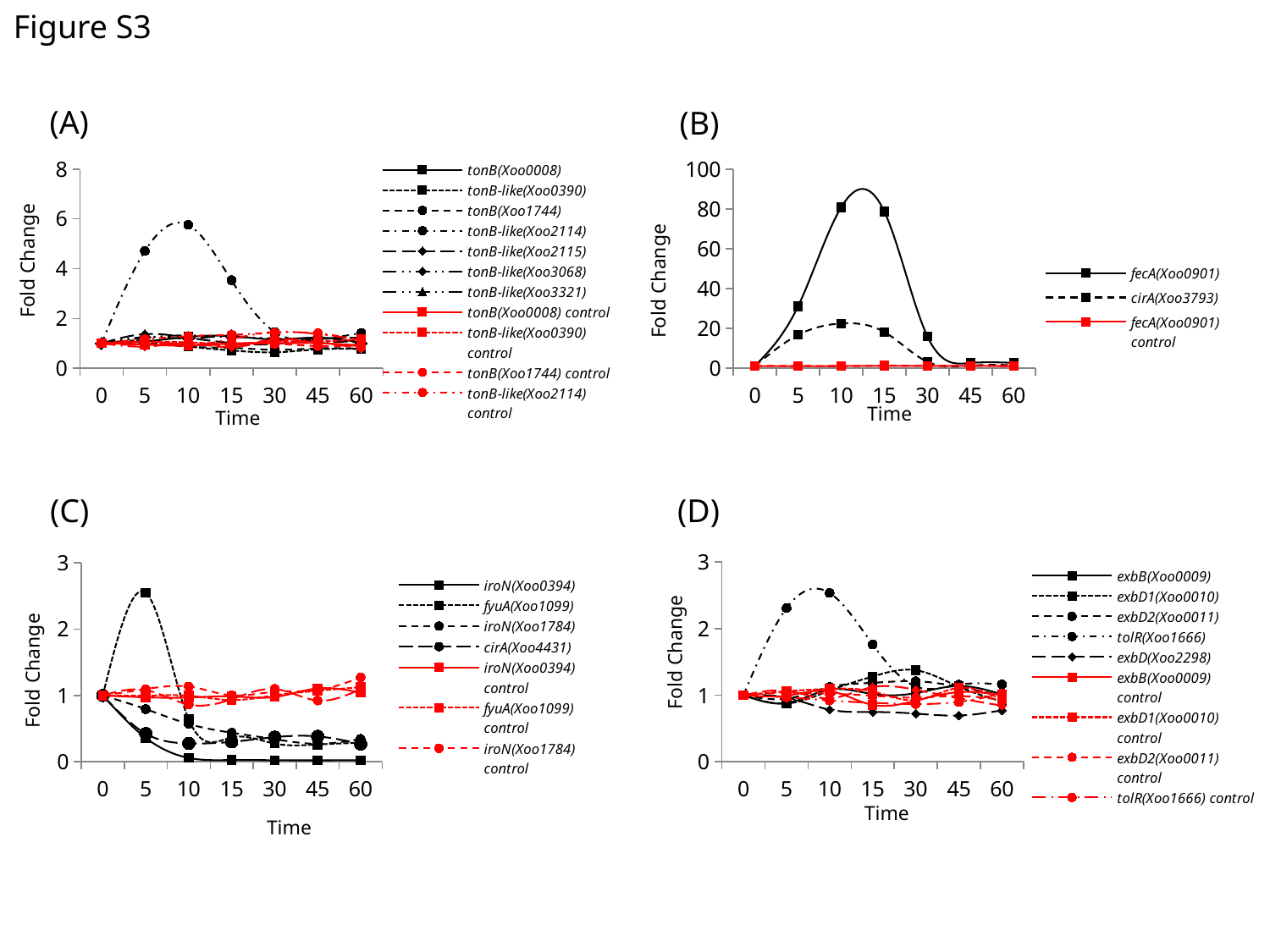

Figure S3
### Chart
| Category | fecA(Xoo0901) | cirA(Xoo3793) | fecA(Xoo0901) control | cirA(Xoo3793) control |
|---|---|---|---|---|
| 0 | 1.0 | 1.0 | 1.0 | 1.0 |
| 5 | 31.02788586251621 | 16.62965340179717 | 0.90580204778157 | 1.049629207073588 |
| 10 | 80.84046692607004 | 22.38318356867779 | 1.018430034129693 | 0.889332572732459 |
| 15 | 78.70233463035018 | 18.09435173299102 | 1.006825938566553 | 1.146035367940673 |
| 30 | 15.8242542153048 | 3.083440308087291 | 1.045733788395904 | 0.996006845407872 |
| 45 | 2.511673151750973 | 1.228498074454428 | 1.047098976109215 | 1.077581289218482 |
| 60 | 2.570687418936447 | 1.443517329910141 | 0.972696245733788 | 0.909298345693098 |Fold Change
Time
### Chart
| Category | tonB(Xoo0008) | tonB-like(Xoo0390) | tonB(Xoo1744) | tonB-like(Xoo2114) | tonB-like(Xoo2115) | tonB-like(Xoo3068) | tonB-like(Xoo3321) | tonB(Xoo0008) control | tonB-like(Xoo0390) control | tonB(Xoo1744) control | tonB-like(Xoo2114) control | tonB-like(Xoo2115) control | tonB-like(Xoo3068) control | tonB-like(Xoo3321) control |
|---|---|---|---|---|---|---|---|---|---|---|---|---|---|---|
| 0 | 1.0 | 1.0 | 1.0 | 1.0 | 1.0 | 1.0 | 1.0 | 1.0 | 1.0 | 1.0 | 1.0 | 1.0 | 1.0 | 1.0 |
| 5 | 1.075970996078775 | 1.107474429583006 | 1.021896162528217 | 4.704312477907387 | 1.372093023255814 | 1.243984708792444 | 1.229638009049774 | 0.950594434525106 | 1.060926181441876 | 1.064027149321267 | 1.16301600673968 | 0.942980463425716 | 0.86991489029437 | 1.037806301050175 |
| 10 | 1.256544502617801 | 0.883141406194121 | 0.86568848758465 | 5.756804524566984 | 1.227707233556855 | 1.199910051720261 | 1.317873303167421 | 0.906893698558551 | 1.039036449350351 | 0.956900452488688 | 1.285874754282505 | 1.00261244888687 | 1.003591785742172 | 0.922987164527421 |
| 15 | 1.287739052334114 | 0.714312280952721 | 0.820541760722347 | 3.534994697773065 | 1.013554001997432 | 1.289521025410389 | 1.252262443438914 | 0.994883072769237 | 0.983155110957801 | 0.96289592760181 | 1.342179163156417 | 0.849727396637892 | 0.918872491606153 | 1.17316219369895 |
| 30 | 1.158031939363404 | 0.639939918460768 | 0.73803611738149 | 1.458112407211029 | 0.973890711941789 | 1.155498088599056 | 1.194570135746607 | 1.003070156338458 | 1.070110382890652 | 0.984276018099548 | 1.425723111485538 | 1.091435711040436 | 1.149449519793863 | 1.45460910151692 |
| 45 | 1.228679708208285 | 0.758372076389386 | 0.79706546275395 | 1.153057617532697 | 1.179198173776573 | 1.202496064762762 | 1.075037707390649 | 1.004637895745329 | 1.075227089801081 | 0.889027149321267 | 1.405925301881494 | 1.018059972739664 | 1.058249394862184 | 1.372462077012835 |
| 60 | 1.026199916756117 | 0.77513768686074 | 0.789390519187359 | 1.416401555319901 | 1.224283064631189 | 1.000337306049022 | 1.086726998491704 | 0.91490658886034 | 1.174054271587904 | 0.938348416289593 | 1.101516427969671 | 0.886983189459336 | 0.791207933161552 | 1.143523920653442 |Fold Change
Time
(A)
(B)
### Chart
| Category | iroN(Xoo0394) | fyuA(Xoo1099) | iroN(Xoo1784) | cirA(Xoo4431) | iroN(Xoo0394) control | fyuA(Xoo1099) control | iroN(Xoo1784) control | cirA(Xoo4431) control |
|---|---|---|---|---|---|---|---|---|
| 0 | 1.0 | 1.0 | 1.0 | 1.0 | 1.0 | 1.0 | 1.0 | 1.0 |
| 5 | 0.360482732058031 | 2.550374359647971 | 0.79444498116124 | 0.424443106562312 | 0.972311078127799 | 0.997393489930803 | 1.095771555158275 | 1.081903528650049 |
| 10 | 0.0583643599948646 | 0.641304347826087 | 0.57010916819631 | 0.276941601444913 | 0.969291399032679 | 0.996276414186862 | 1.135364645071024 | 0.863386209129168 |
| 15 | 0.0262164591089999 | 0.368809930382241 | 0.437812771712878 | 0.300421432871764 | 0.98595081505745 | 0.930803363639185 | 1.00212782495482 | 0.943023632243445 |
| 30 | 0.0235973809218128 | 0.274037830027584 | 0.34094290406724 | 0.371161950632149 | 0.976738234767254 | 1.002172091724331 | 1.055673228269952 | 1.098089996762707 |
| 45 | 0.0212350751059186 | 0.260442663864443 | 0.271055936624481 | 0.382901866345575 | 1.106251759346931 | 1.070779160331399 | 1.081284856493267 | 0.923599870508255 |
| 60 | 0.0243676980356914 | 0.287994220412452 | 0.345097092068399 | 0.267609873570138 | 1.044348337896973 | 1.126353678592485 | 1.272400458599716 | 1.096147620589188 |Fold Change
Time
### Chart
| Category | exbB(Xoo0009) | exbD1(Xoo0010) | exbD2(Xoo0011) | tolR(Xoo1666) | exbD(Xoo2298) | exbB(Xoo0009) control | exbD1(Xoo0010) control | exbD2(Xoo0011) control | tolR(Xoo1666) control | exbD(Xoo2298) control |
|---|---|---|---|---|---|---|---|---|---|---|
| 0 | 1.0 | 1.0 | 1.0 | 1.0 | 1.0 | 1.0 | 1.0 | 1.0 | 1.0 | 1.0 |
| 5 | 0.880599976654605 | 0.874300292126258 | 0.947386045932075 | 2.308012305110815 | 0.963980507097959 | 0.992878068771494 | 1.058826039644099 | 1.036879539233216 | 0.968022194641162 | 1.065219107777339 |
| 10 | 1.083319715186179 | 1.068196960292468 | 1.122267477629724 | 2.535586939802838 | 0.781976128257645 | 1.06110960353106 | 1.094373439733715 | 1.022662542128831 | 0.920384025699058 | 0.952980655349388 |
| 15 | 1.01666277576748 | 1.28103912669624 | 1.185398771095034 | 1.761273858631056 | 0.747227911575676 | 0.853136474145617 | 1.055564530632276 | 0.991352711277077 | 0.88424472512229 | 1.120686932491117 |
| 30 | 1.030827594257033 | 1.374274667015927 | 1.209525181648976 | 1.129658113682444 | 0.722791157567625 | 0.917413841800087 | 0.924899944828679 | 0.962807955822086 | 0.868073300722786 | 1.079628898539281 |
| 45 | 1.137483950040854 | 1.125722485749591 | 1.124685570101319 | 1.157868978535971 | 0.694046189702662 | 1.04469205677608 | 1.109077663540292 | 0.98634076488552 | 0.898298897568811 | 0.953928148440584 |
| 60 | 1.011876969767713 | 0.904714450853886 | 0.994579734749663 | 1.16272809900021 | 0.772159050780422 | 1.001977083552548 | 0.938403201765482 | 0.930038449975474 | 1.026757684164415 | 0.842202921437031 |Fold Change
Time
(C)
(D)
