# Supplementary material for: Time-resolved pathogenic gene expression analysis of the plant pathogen Xanthomonas oryzae pv. oryzae
Source: BMC Genomics. 2016 May 10;17:345. doi: 10.1186/s12864-016-2657-7 (PMC4862043; doi:10.1186/s12864-016-2657-7)
Supplement: Additional file 9: Figure S4. — Time-resolved expression of two component system (TCS) genes. (A) phoB-phoR genes were upregulated within 5 min. (B) ppk was upregulated within 5 min whereas ppx expression was not changed. (C) phoP-phoQ genes were also upregulated within 5 min. The unit of time is min. Red lines indicate the expression levels of genes in control (untreated) Xoo cells. Y-axis represents fold-change. (PPTX 71 kb) [file 12864_2016_2657_MOESM9_ESM.pptx]

## Slide 1
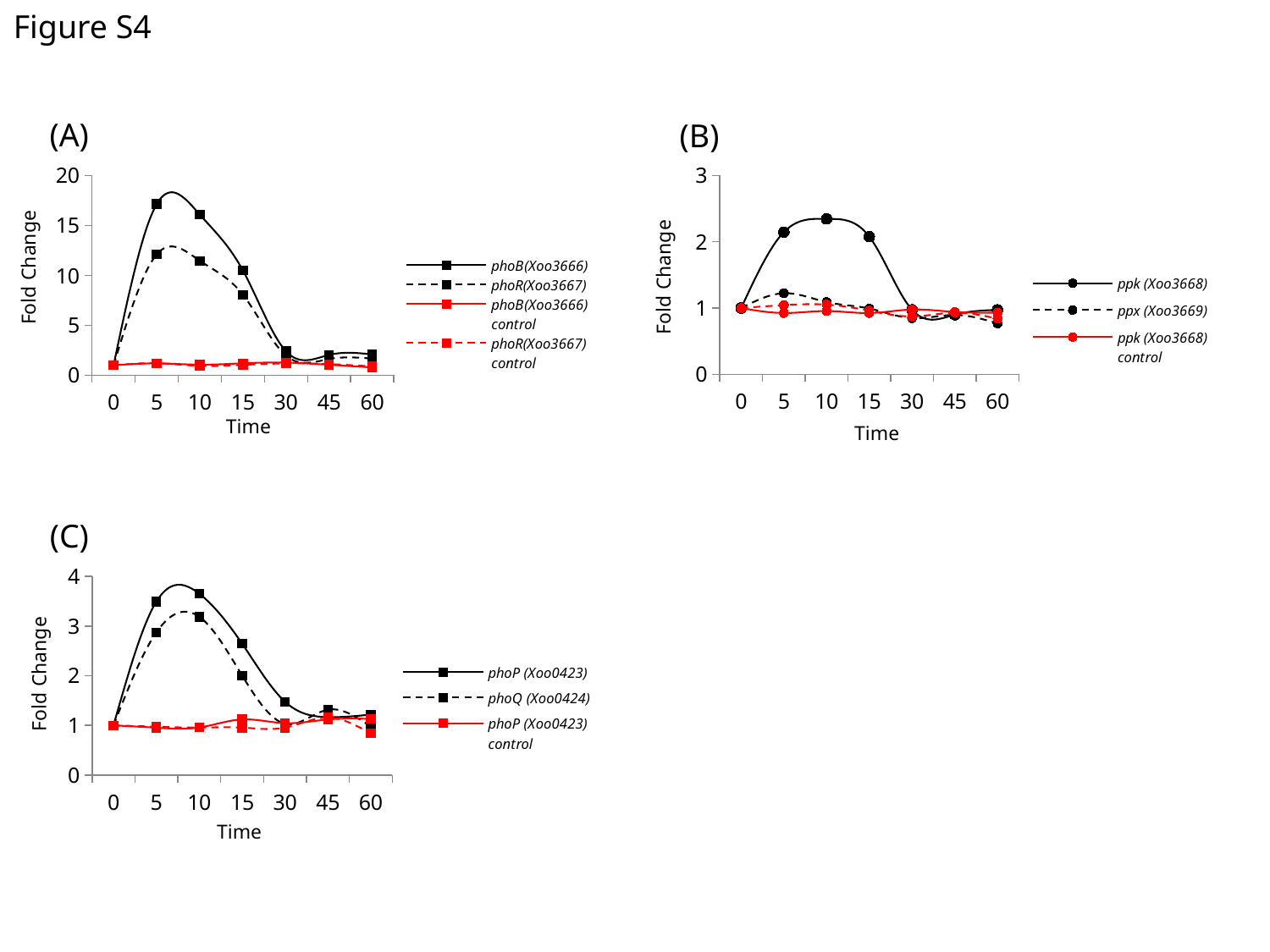

Figure S4
### Chart
| Category | ppk (Xoo3668) | ppx (Xoo3669) | ppk (Xoo3668) control | ppx (Xoo3669) control |
|---|---|---|---|---|
| 0 | 1.0 | 1.0 | 1.0 | 1.0 |
| 5 | 2.146330214784557 | 1.223972659831624 | 0.925687722873153 | 1.044692737430168 |
| 10 | 2.345631835551456 | 1.090522630657664 | 0.955489047376465 | 1.052199720670391 |
| 15 | 2.077348794307551 | 0.993331666249896 | 0.925369332654101 | 0.96106843575419 |
| 30 | 0.973975490842008 | 0.847878636325748 | 0.977903718797758 | 0.873690642458101 |
| 45 | 0.899657398866781 | 0.896474118529632 | 0.941161487519104 | 0.92981843575419 |
| 60 | 0.972987218342338 | 0.768775527215137 | 0.931227712684667 | 0.840869413407821 |Fold Change
Time
### Chart
| Category | phoB(Xoo3666) | phoR(Xoo3667) | phoB(Xoo3666) control | phoR(Xoo3667) control |
|---|---|---|---|---|
| 0 | 1.0 | 1.0 | 1.0 | 1.0 |
| 5 | 17.14699984575043 | 12.1025641025641 | 1.186654478976234 | 1.2 |
| 10 | 16.09285824463983 | 11.46429980276134 | 1.059140767824497 | 0.936379613356766 |
| 15 | 10.49344439302792 | 8.061538461538461 | 1.195978062157221 | 1.037961335676626 |
| 30 | 2.399660650933209 | 1.964102564102564 | 1.26526508226691 | 1.185237258347979 |
| 45 | 2.038870893105044 | 1.626429980276134 | 1.045612431444241 | 1.116695957820738 |
| 60 | 2.086996760758908 | 1.6698224852071 | 0.785923217550274 | 0.882601054481547 |Fold Change
Time
(A)
(B)
### Chart
| Category | phoP (Xoo0423) | phoQ (Xoo0424) | phoP (Xoo0423) control | phoQ (Xoo0424) control |
|---|---|---|---|---|
| 0 | 1.0 | 1.0 | 1.0 | 1.0 |
| 5 | 3.492380136229912 | 2.87312734082397 | 0.95365915175973 | 0.974000616586168 |
| 10 | 3.649406754954016 | 3.185767790262173 | 0.957859609638741 | 0.956736203884493 |
| 15 | 2.643019321324134 | 2.001310861423221 | 1.119911916495286 | 0.954989209742061 |
| 30 | 1.475028933395764 | 1.033333333333333 | 1.045167048232509 | 0.954783681019423 |
| 45 | 1.16654150948397 | 1.31498127340824 | 1.118272476815025 | 1.158359880793341 |
| 60 | 1.216969547048794 | 0.973314606741573 | 1.13111637111482 | 0.83742678039256 |Fold Change
Time
(C)
