# Supplementary material for: Time-resolved pathogenic gene expression analysis of the plant pathogen Xanthomonas oryzae pv. oryzae
Source: BMC Genomics. 2016 May 10;17:345. doi: 10.1186/s12864-016-2657-7 (PMC4862043; doi:10.1186/s12864-016-2657-7)
Supplement: Additional file 10: Table S6. — Fold changes of transcriptional expression levels of chemotaxis and motility-related genes in Xoo and Xcc from two different in vitro assay systems. (DOCX 25 kb) [file 12864_2016_2657_MOESM10_ESM.docx]

**Table S6.** Fold changes of transcriptional expression levels of chemotaxis and motility-related genes in *Xoo* and *Xcc* from two different *in vitro* assay systems.

| **Gene name** | **Annotation** | **Fold Change** | | | | | | |
| --- | --- | --- | --- | --- | --- | --- | --- | --- |
|  |  | Xoo (control: 0 min)* | | | | | | Xcc (MMX/NYG)** |
|  |  | 5 | 10 | 15 | 30 | 45 | 60 |  |
| *flgM* | flagella protein | 0.56 | 0.56 | 0.99 | 2.03 | 1.52 | 1.19 | 0.54 |
| *flgA* | flagella protein | 0.45 | 0.84 | 1.66 | 2.20 | 1.97 | 1.35 | 9.83 |
| *flgB* | flagella protein | 0.38 | 0.78 | 1.63 | 2.15 | 1.64 | 1.19 | 10.15 |
| *flgC* | flagella biosynthesis, cell-proximal portion of basal-body rod | 0.34 | 0.70 | 1.35 | 1.81 | 1.64 | 1.27 | 7.10 |
| *flgD* | flagella protein | 0.29 | 0.65 | 1.59 | 2.28 | 1.34 | 1.35 | 3.00 |
| *flgE* | flagella biosynthesis, hook protein | 0.25 | 0.49 | 0.92 | 1.40 | 0.98 | 1.31 | 1.15 |
| *flgF* | flagella protein | 0.40 | 0.70 | 1.44 | 2.62 | 1.74 | 1.72 | 1.00 |
| *flgG* | flagella biosynthesis, cell-distal portion of basal-body rod | 0.38 | 0.83 | 1.54 | 1.65 | 1.22 | 1.27 | 6.37 |
| *flgH* | flagella L-ring protein | 0.37 | 0.78 | 1.30 | 1.64 | 1.23 | 1.44 | 4.84 |
| *flgI* | flagella protein | 0.39 | 0.77 | 1.48 | 1.91 | 1.60 | 1.64 | 2.96 |
| *flgJ* | flagella protein | 0.42 | 0.78 | 1.62 | 2.42 | 1.80 | 1.65 | 1.45 |
| *flgK* | flagella protein | 0.44 | 0.66 | 1.33 | 2.15 | 1.61 | 1.57 | 1.22 |
| *flgL* | flagella protein | 0.55 | 0.59 | 1.22 | 2.41 | 1.61 | 1.58 | 0.50 |
| *fliC* | flagella protein | 0.57 | 0.46 | 0.61 | 2.27 | 1.74 | 1.49 | 0.05 |
| *fliD* | flagella protein | 0.51 | 0.64 | 1.13 | 1.94 | 1.53 | 1.48 | 1.33 |
| *fliS* | flagella protein | 0.54 | 0.56 | 1.09 | 2.68 | 2.08 | 1.87 | 0.30 |
| *fliE* | flagella protein | 0.64 | 1.48 | 2.82 | 2.93 | 2.13 | 1.61 | 27.96 |
| *fliF* | flagella protein | 0.53 | 0.91 | 2.00 | 2.64 | 1.83 | 1.54 | 9.39 |
| *fliG* | flagella protein | 0.48 | 0.79 | 1.54 | 2.10 | 1.57 | 1.50 | 2.35 |
| *fliH* | flagella protein | 0.55 | 0.87 | 1.33 | 1.89 | 1.96 | 1.64 | 1.37 |
| *fliI* | flagella protein | 0.54 | 0.69 | 1.20 | 1.80 | 1.74 | 1.59 | 0.74 |
| *fliJ* | flagella FliJ protein | 0.47 | 0.57 | 1.31 | 2.26 | 1.56 | 1.29 | 0.31 |
| *fliK* | flagella protein | 0.52 | 0.52 | 0.92 | 1.79 | 1.64 | 1.53 | 0.34 |
| *fliL* | flagella biosynthesis protein | 0.52 | 0.99 | 2.16 | 2.49 | 1.64 | 1.39 | 28.46 |
| *fliM* | flagella protein | 0.47 | 0.84 | 1.78 | 2.24 | 1.50 | 1.35 | 10.37 |
| *fliN* | flagella protein | 0.47 | 0.72 | 1.66 | 2.32 | 1.56 | 1.42 | 4.41 |
| *fliO* | flagella protein | 0.60 | 0.76 | 1.60 | 2.42 | 1.53 | 1.56 | 11.81 |
| *fliP* | flagella biosynthetic protein | 0.63 | 0.83 | 1.57 | 2.75 | 1.82 | 1.73 | 4.00 |
| *fliQ* | flagella biosynthesis | 0.67 | 1.03 | 2.40 | 2.73 | 2.34 | 1.75 | 11.61 |
| *fliR* | flagella biosynthetic protein | 0.73 | 0.87 | 1.60 | 2.28 | 2.01 | 1.37 | 8.66 |
| *flhB* | flagella protein | 0.48 | 0.77 | 1.97 | 2.49 | 1.86 | 1.50 | 12.45 |
| *flhA* | flagella biosynthetic protein FlhA | 0.46 | 0.71 | 1.31 | 1.92 | 1.54 | 1.29 | 7.55 |
| *flhF* | flagella biosynthetic protein | 0.43 | 0.84 | 1.33 | 1.69 | 1.51 | 1.55 | 8.16 |
| *fleN* | flagella biosynthesis switch protein | 0.39 | 0.72 | 1.42 | 2.00 | 1.50 | 1.51 | 2.14 |
| *cheW* | chemotaxis protein | 0.76 | 0.33 | 0.43 | 1.54 | 1.37 | 1.25 | 0.64 |
| *cheY* | chemotaxis response regulator | 0.46 | 0.60 | 1.12 | 2.19 | 1.70 | 1.81 | 0.11 |
| *cheA* | chemotaxis protein | 0.66 | 0.51 | 0.84 | 2.52 | 2.21 | 2.06 | 0.09 |
| *tsr* | chemotaxis protein | 1.11 | 1.21 | 1.22 | 1.36 | 1.29 | 1.35 | 0.92 |
| *tsr* | chemotaxis protein | 0.71 | 0.59 | 0.75 | 2.06 | 1.88 | 1.57 | 0.23 |
| *tsr* | chemotaxis protein | 0.29 | 0.20 | 0.34 | 1.26 | 1.02 | 0.81 | 0.43 |
| *tsr* | chemotaxis protein | 0.56 | 0.44 | 0.76 | 2.35 | 1.82 | 1.66 | 1.37 |
| *tsr* | chemotaxis protein | 1.57 | 1.26 | 2.06 | 1.69 | 1.60 | 1.07 | 0.12 |
| *tsr* | chemotaxis protein | 0.94 | 0.98 | 1.62 | 5.05 | 3.18 | 3.32 | 0.17 |
| *tsr* | chemotaxis protein | 0.61 | 0.53 | 0.86 | 3.31 | 2.30 | 2.01 | 0.47 |
| *cheW* | chemotaxis protein | 0.53 | 0.29 | 0.63 | 3.39 | 2.56 | 2.67 | 0.05 |
| *cheR* | chemotaxis protein methyltransferase | 0.41 | 0.33 | 0.97 | 3.75 | 2.72 | 2.51 | 0.04 |
| *cheD* | chemotaxis protein | 0.73 | 0.80 | 1.09 | 1.93 | 1.33 | 1.34 | 2.03 |
| *cheB* | glutamate methylesterase | 0.79 | 0.73 | 0.68 | 1.25 | 1.11 | 1.00 | 1.29 |

* Data from this study

** Data from reference [30]
