# Supplementary material for: Time-resolved pathogenic gene expression analysis of the plant pathogen Xanthomonas oryzae pv. oryzae
Source: BMC Genomics. 2016 May 10;17:345. doi: 10.1186/s12864-016-2657-7 (PMC4862043; doi:10.1186/s12864-016-2657-7)
Supplement: Additional file 11: Figure S5. — Time-resolved expression of EAL-GGDEF and HD-GYP containing genes. The unit of time is min. Red lines indicate expression levels of genes in control (untreated) Xoo cells. Y-axis represents fold-change. (PPTX 70 kb) [file 12864_2016_2657_MOESM11_ESM.pptx]

## Slide 1
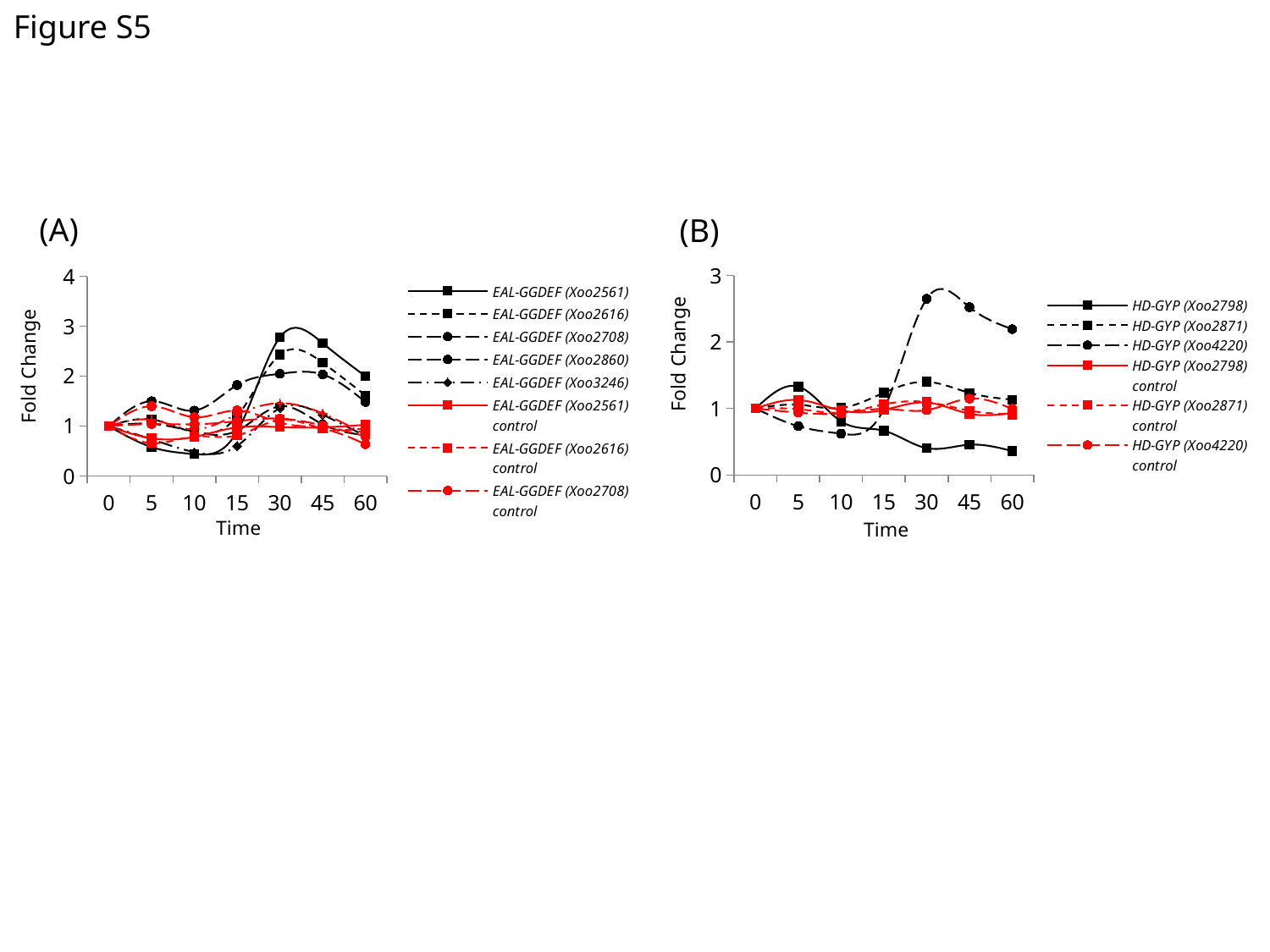

Figure S5
### Chart
| Category | HD-GYP (Xoo2798) | HD-GYP (Xoo2871) | HD-GYP (Xoo4220) | HD-GYP (Xoo2798) control | HD-GYP (Xoo2871) control | HD-GYP (Xoo4220) control |
|---|---|---|---|---|---|---|
| 0 | 1.0 | 1.0 | 1.0 | 1.0 | 1.0 | 1.0 |
| 5 | 1.329091551785083 | 1.056070664398969 | 0.733161229758273 | 1.128084236995128 | 0.989205794487346 | 0.937022900763359 |
| 10 | 0.805761753269707 | 1.0084764360564 | 0.620980990377846 | 0.97045418827597 | 0.93791218082408 | 0.926208651399491 |
| 15 | 0.665075998586073 | 1.236791572941241 | 0.992959399202065 | 0.979412226936979 | 1.062595118651711 | 0.993956743002544 |
| 30 | 0.404029692470838 | 1.400559609370714 | 2.649143393569584 | 1.08989470375609 | 1.096048700749676 | 0.974872773536896 |
| 45 | 0.455461293743372 | 1.234322708070445 | 2.523351325979817 | 0.91984912776992 | 0.967166450594668 | 1.147900763358779 |
| 60 | 0.360551431601272 | 1.133154112031601 | 2.192677775170148 | 0.933836240766934 | 0.909757059917705 | 0.995229007633588 |Fold Change
Time
### Chart
| Category | EAL-GGDEF (Xoo2561) | EAL-GGDEF (Xoo2616) | EAL-GGDEF (Xoo2708) | EAL-GGDEF (Xoo2860) | EAL-GGDEF (Xoo3246) | EAL-GGDEF (Xoo2561) control | EAL-GGDEF (Xoo2616) control | EAL-GGDEF (Xoo2708) control | EAL-GGDEF (Xoo2860) control | EAL-GGDEF (Xoo3246) control |
|---|---|---|---|---|---|---|---|---|---|---|
| 0 | 1.0 | 1.0 | 1.0 | 1.0 | 1.0 | 1.0 | 1.0 | 1.0 | 1.0 | 1.0 |
| 5 | 0.580477673935618 | 1.13508064516129 | 1.498619547211485 | 1.045465777118854 | 0.736576121288692 | 0.754412316935787 | 0.65728476821192 | 1.040997229916898 | 1.398069067953955 | 1.127487562189055 |
| 10 | 0.440290758047767 | 0.872983870967742 | 1.310877967973495 | 0.889547813194959 | 0.479469361970941 | 0.784829140067593 | 0.791390728476821 | 1.03601108033241 | 1.177992325782894 | 0.933457711442786 |
| 15 | 0.891658013153341 | 1.195564516129032 | 1.822749861954721 | 0.907338769458858 | 0.602021478205938 | 0.975591438227563 | 0.811258278145695 | 1.099168975069252 | 1.313405124396584 | 1.232587064676617 |
| 30 | 2.779854620976116 | 2.433467741935484 | 2.048591938155716 | 1.398937484556462 | 1.359444093493367 | 0.979346601577169 | 1.125827814569536 | 1.151246537396122 | 1.067335066221067 | 1.458955223880597 |
| 45 | 2.661128418137764 | 2.270161290322581 | 2.034235229155163 | 1.03409933283914 | 1.229311433986102 | 0.975591438227563 | 0.955298013245033 | 1.01218836565097 | 0.947023146429014 | 1.254353233830846 |
| 60 | 1.997923156801661 | 1.61491935483871 | 1.483158475980121 | 0.818260439831974 | 0.80227416298168 | 1.025385764061723 | 0.954659949622166 | 0.796700372538585 | 0.637361664453298 | 0.827909542195257 |Fold Change
Time
(A)
(B)
