# Supplementary material for: Time-resolved pathogenic gene expression analysis of the plant pathogen Xanthomonas oryzae pv. oryzae
Source: BMC Genomics. 2016 May 10;17:345. doi: 10.1186/s12864-016-2657-7 (PMC4862043; doi:10.1186/s12864-016-2657-7)
Supplement: Additional file 12: Figure S6. — Time-resolved expression of gumH–gumM genes. The unit of time is min. Red lines indicate expression levels of genes in control (untreated) Xoo cells. Y-axis represents fold-change. (PPTX 51 kb) [file 12864_2016_2657_MOESM12_ESM.pptx]

## Slide 1
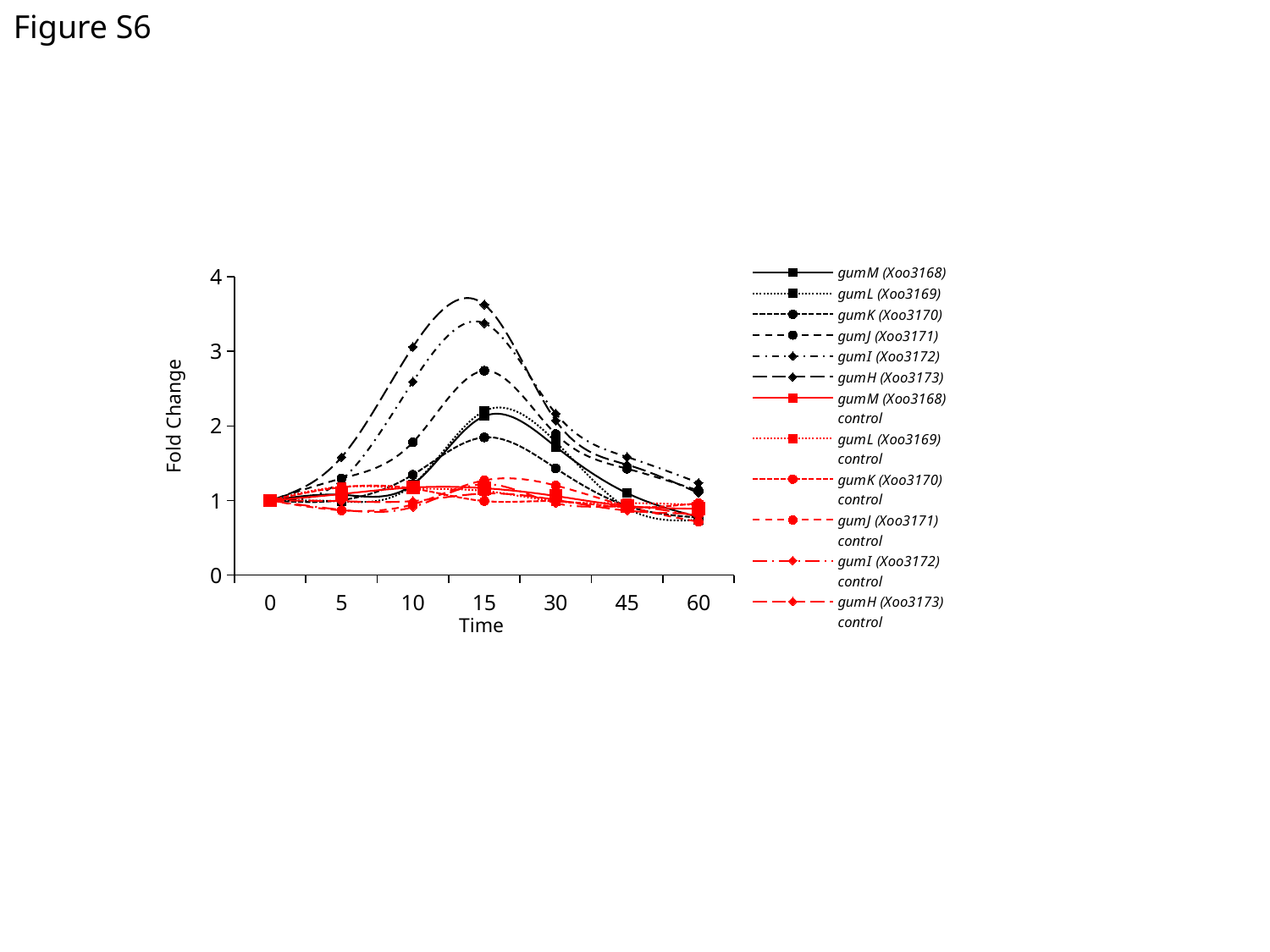

Figure S6
### Chart
| Category | gumM (Xoo3168) | gumL (Xoo3169) | gumK (Xoo3170) | gumJ (Xoo3171) | gumI (Xoo3172) | gumH (Xoo3173) | gumM (Xoo3168) control | gumL (Xoo3169) control | gumK (Xoo3170) control | gumJ (Xoo3171) control | gumI (Xoo3172) control | gumH (Xoo3173) control |
|---|---|---|---|---|---|---|---|---|---|---|---|---|
| 0 | 1.0 | 1.0 | 1.0 | 1.0 | 1.0 | 1.0 | 1.0 | 1.0 | 1.0 | 1.0 | 1.0 | 1.0 |
| 5 | 1.076814181079584 | 0.988596957105814 | 1.005708228002935 | 1.295266468056935 | 1.273355225908731 | 1.579828690485376 | 1.087098162148872 | 1.184377221899037 | 1.177646408369838 | 0.868649698456506 | 0.875508289020957 | 0.990694894997439 |
| 10 | 1.214501138671755 | 1.212127839045269 | 1.345388567234772 | 1.780315568796205 | 2.592231910315933 | 3.058955166293695 | 1.174073391111931 | 1.16275612436171 | 1.16114489856675 | 0.948686496984565 | 0.912834949431759 | 0.993341301007341 |
| 15 | 2.130239428817628 | 2.199162735480646 | 1.845959390035065 | 2.738938541321858 | 3.376288076095573 | 3.62589705995833 | 1.165158882732104 | 1.128823641771496 | 0.99284940411701 | 1.270887267592375 | 1.2194831487436 | 1.092804825506247 |
| 30 | 1.720994645165261 | 1.78643506513793 | 1.43105276033597 | 1.890323292507999 | 2.164986977692221 | 2.071456130874295 | 1.060667527198968 | 0.990498351754896 | 0.9901331178497 | 1.201063068588368 | 0.964237305807528 | 1.008451425644528 |
| 45 | 1.098602818981966 | 0.89924708675685 | 0.926771589333768 | 1.425797197396005 | 1.583172913599819 | 1.479280808704375 | 0.926793287848055 | 0.96632409023334 | 0.910815293667333 | 0.921189819073904 | 0.919299343134189 | 0.864606453815947 |
| 60 | 0.779282329045362 | 0.739479521384611 | 0.771670879882574 | 1.133620214057155 | 1.238138376174839 | 1.100084883092831 | 0.890097731882722 | 0.940404628013703 | 0.964062433547399 | 0.714300316876214 | 0.790428526743822 | 0.82525183541062 |Fold Change
Time
