# Supplementary material for: Time-resolved pathogenic gene expression analysis of the plant pathogen Xanthomonas oryzae pv. oryzae
Source: BMC Genomics. 2016 May 10;17:345. doi: 10.1186/s12864-016-2657-7 (PMC4862043; doi:10.1186/s12864-016-2657-7)
Supplement: Additional file 13: Table S7. — Fold changes of transcriptional expression levels of gum genes in Xoo and Xcc from two different in vitro assay systems. (DOCX 17 kb) [file 12864_2016_2657_MOESM13_ESM.docx]

**Table S7.** Fold changes of transcriptional expression levels of *gum* genes in *Xoo* and *Xcc* from two different *in vitro* assay systems.

| **Gene name** | **Annotation** | **Fold Change** | | | | | | |
| --- | --- | --- | --- | --- | --- | --- | --- | --- |
|  |  | Xoo (control: 0 min)* | | | | | | Xcc (MMX/NYG)** |
|  |  | 5 | 10 | 15 | 30 | 45 | 60 |  |
| *gumB* | GumB protein | 4.89 | 5.55 | 5.23 | 2.89 | 1.69 | 2.03 | 3.62 |
| *gumC* | GumC protein | 4.11 | 5.35 | 4.95 | 2.60 | 1.57 | 1.47 | 3.33 |
| *gumD* | GumD protein | 4.00 | 7.11 | 5.80 | 2.26 | 1.35 | 1.20 | 6.41 |
| *gumE* | GumE protein | 2.83 | 5.15 | 4.69 | 2.15 | 1.16 | 1.00 | 4.15 |
| *gumF* | GumF protein | 2.32 | 3.61 | 3.79 | 2.10 | 1.42 | 1.02 | 3.88 |
| *gumG* | GumG protein | 2.13 | 3.42 | 3.65 | 2.50 | 1.68 | 1.40 | 3.35 |
| *gumH* | GumH protein | 1.58 | 3.06 | 3.63 | 2.07 | 1.48 | 1.10 | 2.64 |
| *gumI* | GumI protein | 1.27 | 2.59 | 3.38 | 2.16 | 1.58 | 1.24 | 2.87 |
| *gumJ* | GumJ protein | 1.30 | 1.78 | 2.74 | 1.89 | 1.43 | 1.13 | 3.03 |
| *gumK* | GumK protein | 1.01 | 1.35 | 1.85 | 1.43 | 0.93 | 0.77 | 2.13 |
| *gumL* | GumL protein | 0.99 | 1.21 | 2.20 | 1.79 | 0.90 | 0.74 | 1.97 |
| *gumM* | GumM protein | 1.08 | 1.21 | 2.13 | 1.72 | 1.10 | 0.78 | 2.34 |
| *gumN* | GumN protein | 1.78 | 2.04 | 2.21 | 1.71 | 1.66 | 1.42 | 3.23 |

* Data from this study

** Data from reference [30]
