# Supplementary material for: Time-resolved pathogenic gene expression analysis of the plant pathogen Xanthomonas oryzae pv. oryzae
Source: BMC Genomics. 2016 May 10;17:345. doi: 10.1186/s12864-016-2657-7 (PMC4862043; doi:10.1186/s12864-016-2657-7)
Supplement: Additional file 14: Figure S7. — Time-resolved expression of T2SS substrate genes. (A) Genes like htrA, egl (Xoo0281), celS and xynB were upregulated. (B) Genes like egl (Xoo0282), Xoo1077, engXCA and Xoo4035 were downregulated. egl (Xoo0283) was downregulated within 10 min and upregulated subsequently. The unit of time is min. Red lines indicate the expression levels of genes in control (untreated) Xoo cells. Y-axis represents fold-change. (PPTX 59 kb) [file 12864_2016_2657_MOESM14_ESM.pptx]

## Slide 1
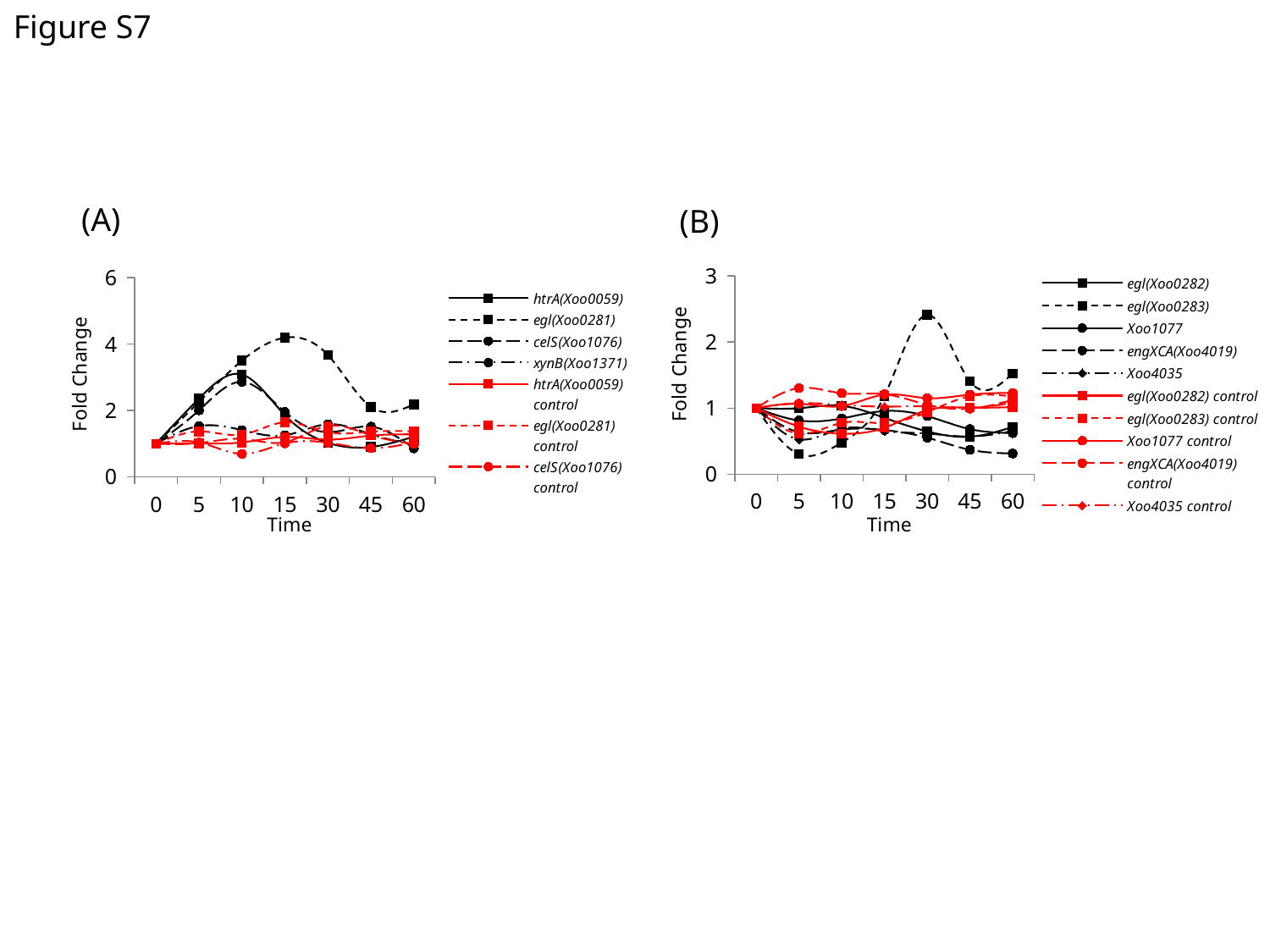

Figure S7
### Chart
| Category | egl(Xoo0282) | egl(Xoo0283) | Xoo1077 | engXCA(Xoo4019) | Xoo4035 | egl(Xoo0282) control | egl(Xoo0283) control | Xoo1077 control | engXCA(Xoo4019) control | Xoo4035 control |
|---|---|---|---|---|---|---|---|---|---|---|
| 0 | 1.0 | 1.0 | 1.0 | 1.0 | 1.0 | 1.0 | 1.0 | 1.0 | 1.0 | 1.0 |
| 5 | 0.99900314795383 | 0.302488050006128 | 0.813298355583609 | 0.636158487180002 | 0.527654377323545 | 0.719785886166086 | 0.612191103789127 | 1.066349425697262 | 1.302885144451422 | 1.072464225522809 |
| 10 | 1.033630640083945 | 0.479347959308739 | 0.843344437187818 | 0.675124048293457 | 0.689748889395912 | 0.613465599371409 | 0.776386600768808 | 1.031159452592163 | 1.224907917478525 | 1.049311021758204 |
| 15 | 0.852098635886674 | 1.184213751685256 | 0.956078356970071 | 0.667711283874726 | 0.652129272973735 | 0.702204979619899 | 0.773640856672158 | 1.203844039287359 | 1.210925615447854 | 1.022439923495957 |
| 30 | 0.647901364113326 | 2.414389018262042 | 0.87862258691837 | 0.552649376912621 | 0.615227690614557 | 0.969061533172912 | 0.939373970345964 | 1.150708034294715 | 1.051210181187331 | 1.025981075980129 |
| 45 | 0.567156348373557 | 1.404829023164603 | 0.68165018115215 | 0.369144954030147 | 0.570159309613604 | 1.007611845013014 | 1.180010982976387 | 1.204323244030371 | 0.991274007806785 | 1.003818889933911 |
| 60 | 0.715215110178384 | 1.523961269763451 | 0.621076404978127 | 0.313338288568807 | 0.654041793608826 | 1.015371016058537 | 1.177375068643603 | 1.230348890771037 | 1.124694946304076 | 1.082336529418077 |Fold Change
Time
### Chart
| Category | htrA(Xoo0059) | egl(Xoo0281) | celS(Xoo1076) | xynB(Xoo1371) | htrA(Xoo0059) control | egl(Xoo0281) control | celS(Xoo1076) control | xynB(Xoo1371) control |
|---|---|---|---|---|---|---|---|---|
| 0 | 1.0 | 1.0 | 1.0 | 1.0 | 1.0 | 1.0 | 1.0 | 1.0 |
| 5 | 2.360700440239174 | 2.260704778233995 | 2.005452562704471 | 1.531671858774662 | 1.007846174568966 | 1.369408945686901 | 1.018387553041019 | 1.037510656436487 |
| 10 | 3.083875418884289 | 3.502594414113613 | 2.863685932388222 | 1.40809968847352 | 1.038119612068965 | 1.272803514376997 | 1.144271570014144 | 0.692242114236999 |
| 15 | 1.857612195282213 | 4.194423137661869 | 1.955288985823337 | 1.2533748701973 | 1.202889278017241 | 1.644329073482428 | 1.045261669024045 | 1.0076726342711 |
| 30 | 1.024837374334713 | 3.670035644993909 | 1.347873500545256 | 1.586708203530633 | 1.123282596982759 | 1.345047923322684 | 1.536067892503536 | 1.028985507246377 |
| 45 | 0.900979039358697 | 2.109371474980824 | 1.513631406761178 | 1.28245067497404 | 1.2431640625 | 1.36082268370607 | 1.256011315417256 | 0.861892583120205 |
| 60 | 1.212990341021092 | 2.183549158507423 | 0.859323882224646 | 0.966770508826584 | 1.28094692887931 | 1.37276357827476 | 0.988684582743988 | 1.041773231031543 |Fold Change
Time
(A)
(B)
