# Supplementary material for: Time-resolved pathogenic gene expression analysis of the plant pathogen Xanthomonas oryzae pv. oryzae
Source: BMC Genomics. 2016 May 10;17:345. doi: 10.1186/s12864-016-2657-7 (PMC4862043; doi:10.1186/s12864-016-2657-7)
Supplement: Additional file 15: Table S8. — Virulence of wild-type and mutant Xoo strains on rice. (DOCX 16 kb) [file 12864_2016_2657_MOESM15_ESM.docx]

**Table S8**. Virulence of wild-type and mutant *Xoo* strains on rice

| Gene | Locus tag | Lesion length (cm) | Reference | WAI^*1^ | Host^*2^ |
| --- | --- | --- | --- | --- | --- |
| Wild-type | (KACC10331)^*3^ | 14.3 | this study | 2 | M23 |
| *oprO* | *Xoo1104* | 16.4 | this study | 2 | M23 |
| *hrpX* | *Xoo1380* | 0.46 | this study | 2 | M23 |
| *fliC* | *Xoo2581* | 18.6 | this study | 2 | M23 |
| *fruA* | *Xoo2812* | 20.5 | this study | 2 | M23 |
| *gumC* | *Xoo3178* | 1.3 | this study | 2 | M23 |
| Wild-type | (KACC10859)^*4^ | 46.8 | [46] | 3 | M23 |
| *hrpG* | *Xoo1379* | 0 | [46] | 3 | M23 |
| *hpaB* | *Xoo0075* | 0 | [46] | 3 | M23 |
| *hrpE* | *Xoo0076* | 0 | [46] | 3 | M23 |
| *hrpD6* | *Xoo0077* | 0 | [46] | 3 | M23 |
| *hrpD5* | *Xoo0078* | 0 | [46] | 3 | M23 |
| *hpa1* | *Xoo0095* | 34.5 | [46] | 3 | M23 |
| *hpa2* | *Xoo0096* | 7.27 | [46] | 3 | M23 |
| Wild-type | (KACC10859) | 13.4 | [47] | 3 | IR24 |
| *gumB* | *Xoo3179* | 1.0 | [47] | 3 | IR24 |
| *gumC* | *Xoo3178* | 1.4 | [47] | 3 | IR24 |
| *gumD* | *Xoo3177* | 1.5 | [47] | 3 | IR24 |
| *gumE* | *Xoo3176* | 1.4 | [47] | 3 | IR24 |
| *gumF* | *Xoo3175* | 18.3 | [47] | 3 | IR24 |
| *gumG* | *Xoo3174* | 16.4 | [47] | 3 | IR24 |
| *gumH* | *Xoo3173* | 2.1 | [47] | 3 | IR24 |
| *gumI* | *Xoo3172* | 13.6 | [47] | 3 | IR24 |
| *gumK* | *Xoo3170* | 1.6 | [47] | 3 | IR24 |

^*1^: Weeks afer inoculation

^*2^: Milyang23 (M23), IR24 are susceptible rice.

^*3, *4^: *Xanthomonas oryzae* pv. *oryzae* (*Xoo*) wild-type
