# Supplementary material for: Time-resolved pathogenic gene expression analysis of the plant pathogen Xanthomonas oryzae pv. oryzae
Source: BMC Genomics. 2016 May 10;17:345. doi: 10.1186/s12864-016-2657-7 (PMC4862043; doi:10.1186/s12864-016-2657-7)
Supplement: Additional file 16: Figure S8. — Representation of continuous time-resolved gene expression of selected genes. (A) Scaled continuous time-resolved gene expression. The absolute expression levels of each gene were log2-scaled and the highest level was set to 1.0. The expression levels at each time point were fitted by non-linear regression method to derive time-resolved continuous gene expression levels. (B) Unscaled continuous time-resolved gene expression. The absolute expression levels of each gene were log2-scaled as in (A), but the highest level was not set to 1.0. The expression levels at each time point were also fitted by non-linear regression method to derive the time-resolved continuous gene expression levels. (PPTX 3239 kb) [file 12864_2016_2657_MOESM16_ESM.pptx]

## Slide 1
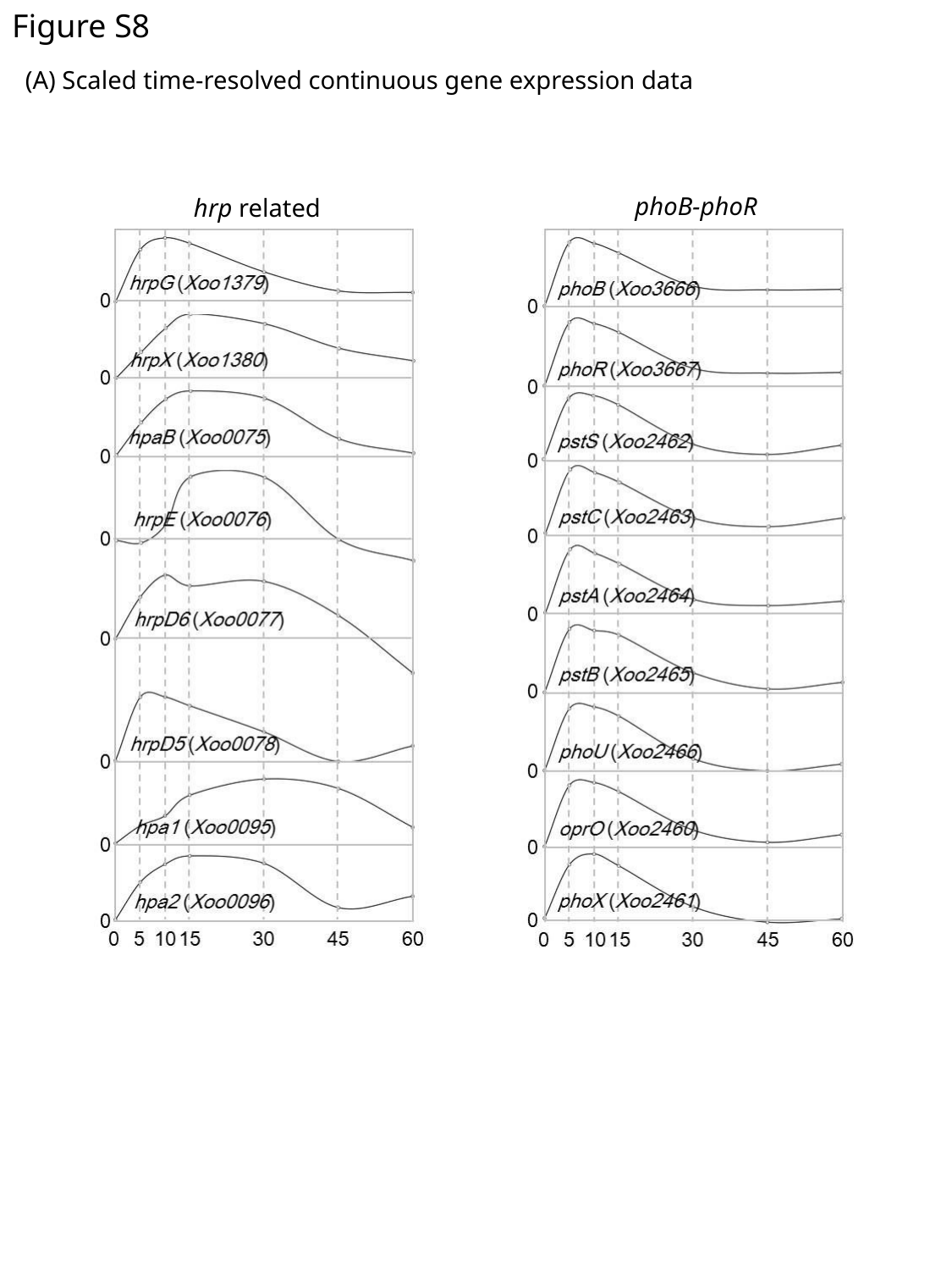

Figure S8
(A) Scaled time-resolved continuous gene expression data
phoB-phoR
hrp related

## Slide 2
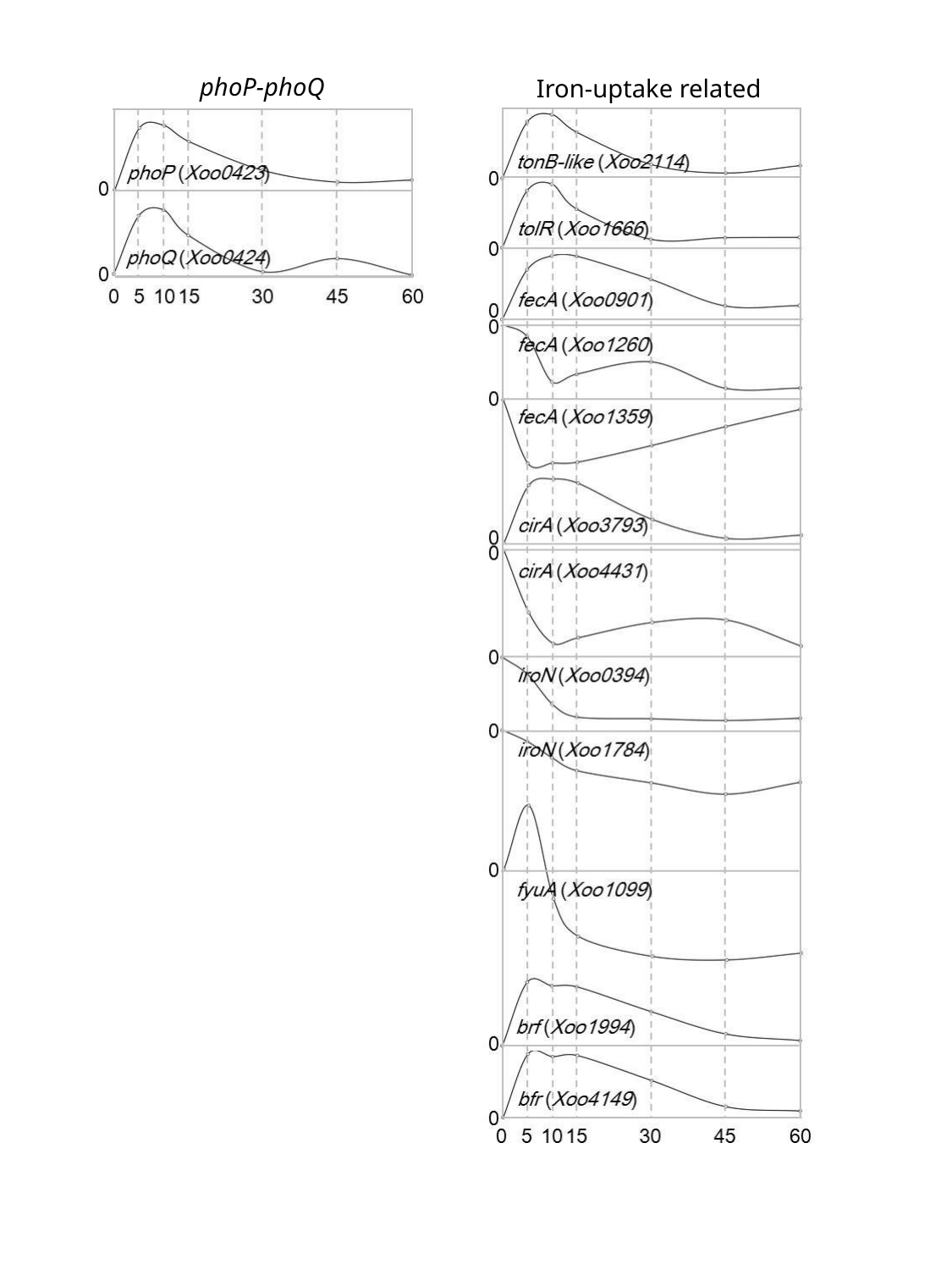

phoP-phoQ
Iron-uptake related

## Slide 3
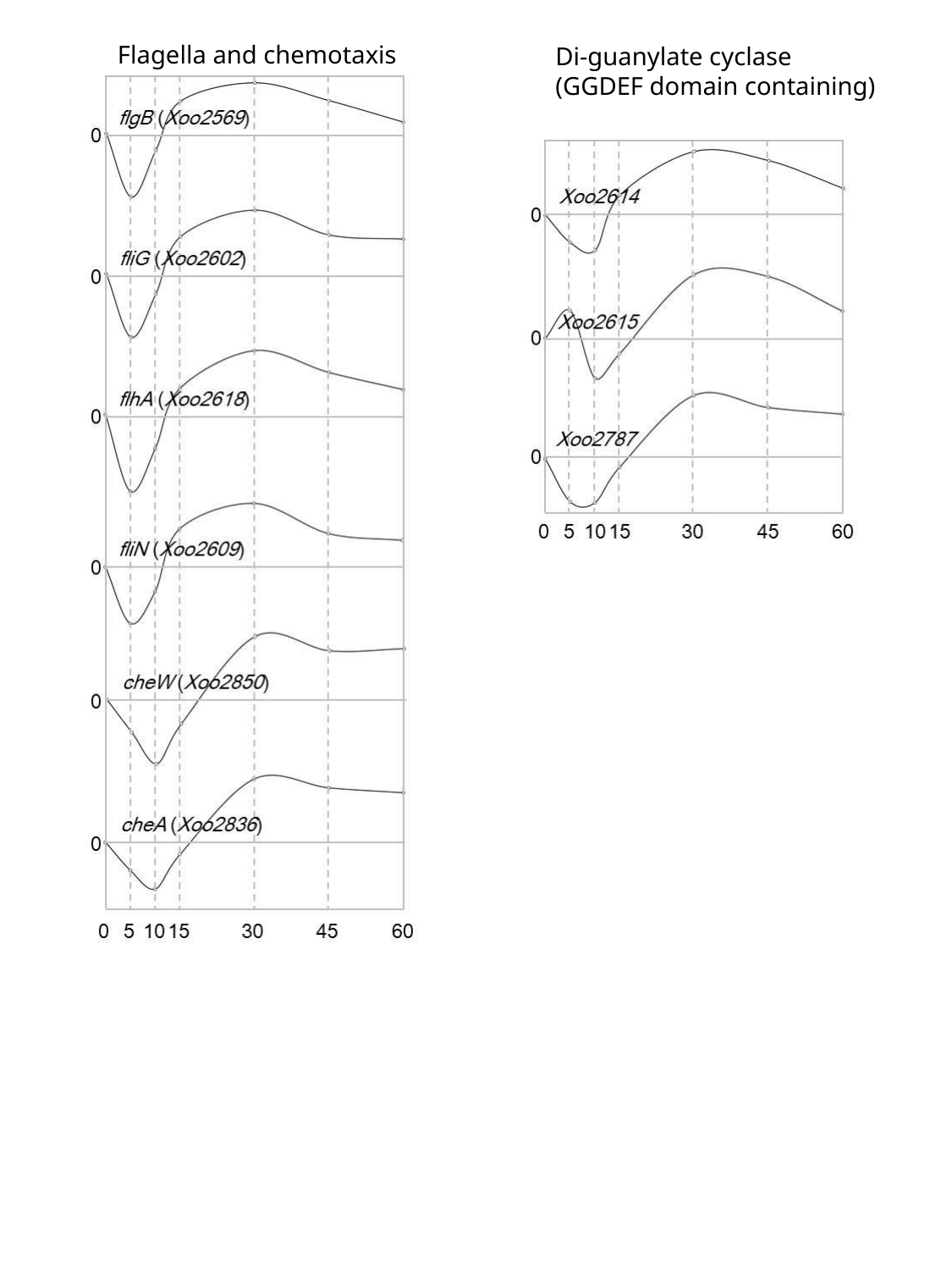

Flagella and chemotaxis
Di-guanylate cyclase
(GGDEF domain containing)

## Slide 4
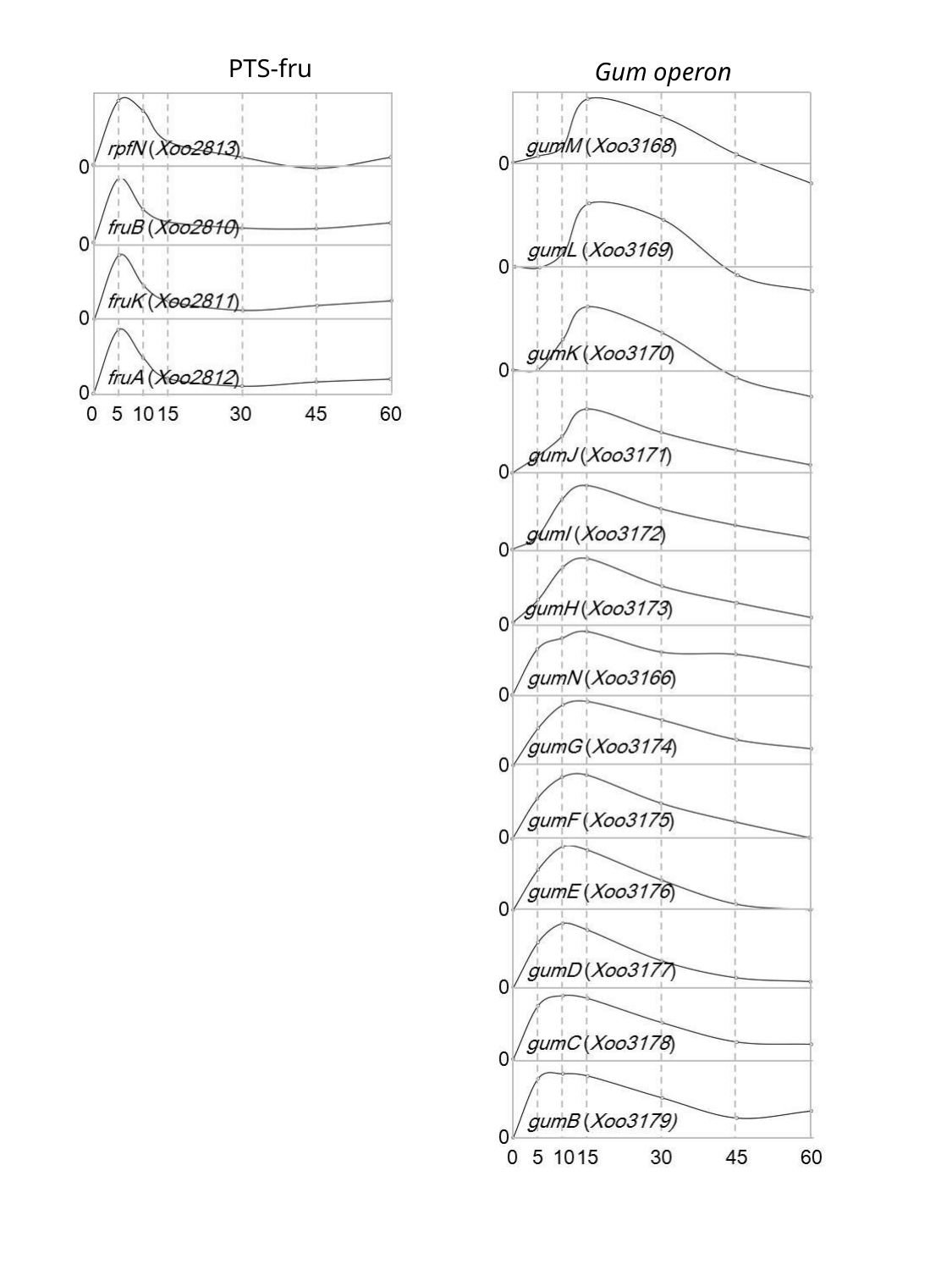

PTS-fru
Gum operon

## Slide 5
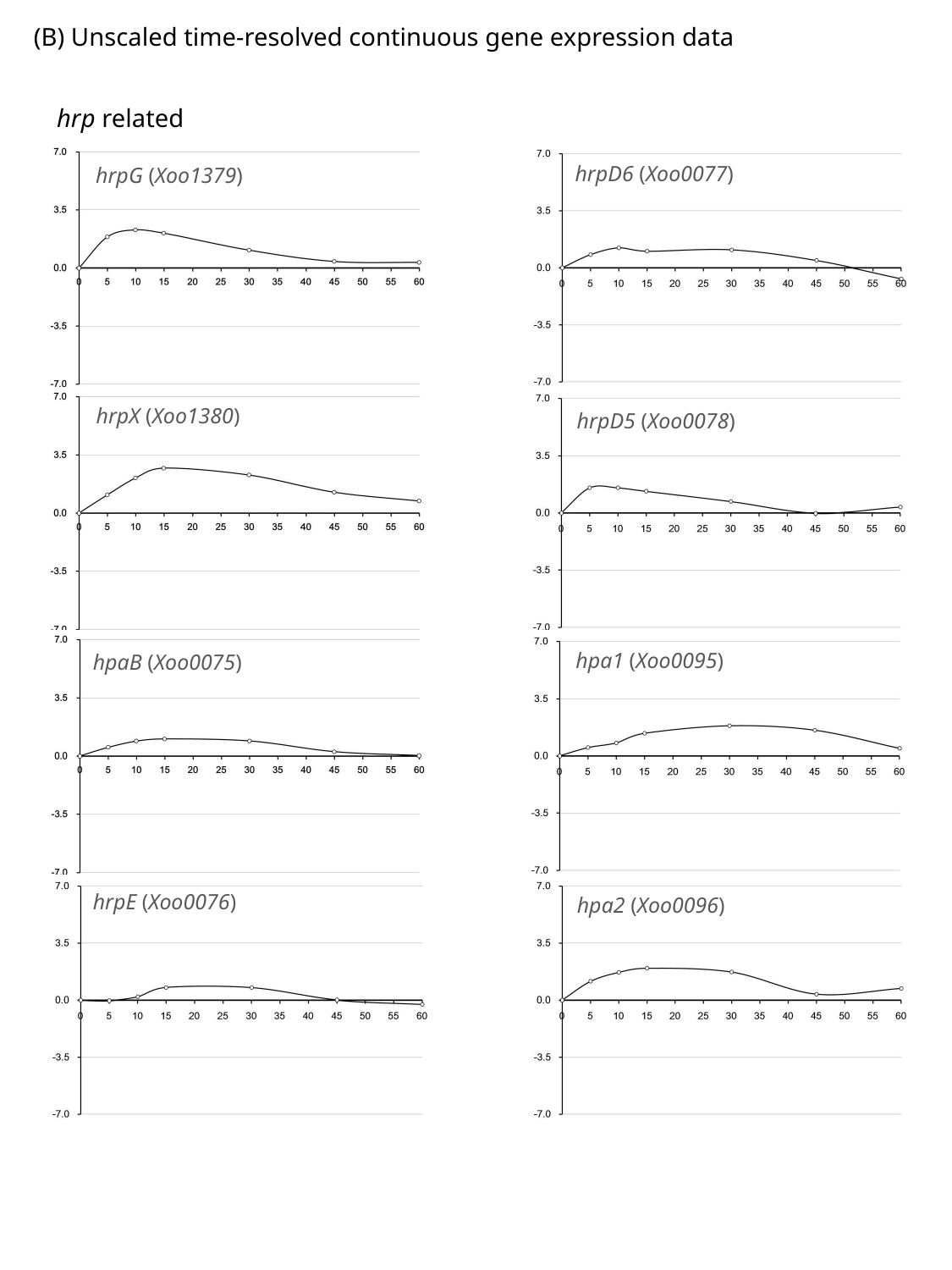

(B) Unscaled time-resolved continuous gene expression data
hrp related
hrpG (Xoo1379)
hrpD6 (Xoo0077)
hrpX (Xoo1380)
hrpD5 (Xoo0078)
hpaB (Xoo0075)
hpa1 (Xoo0095)
hrpE (Xoo0076)
hpa2 (Xoo0096)

## Slide 6
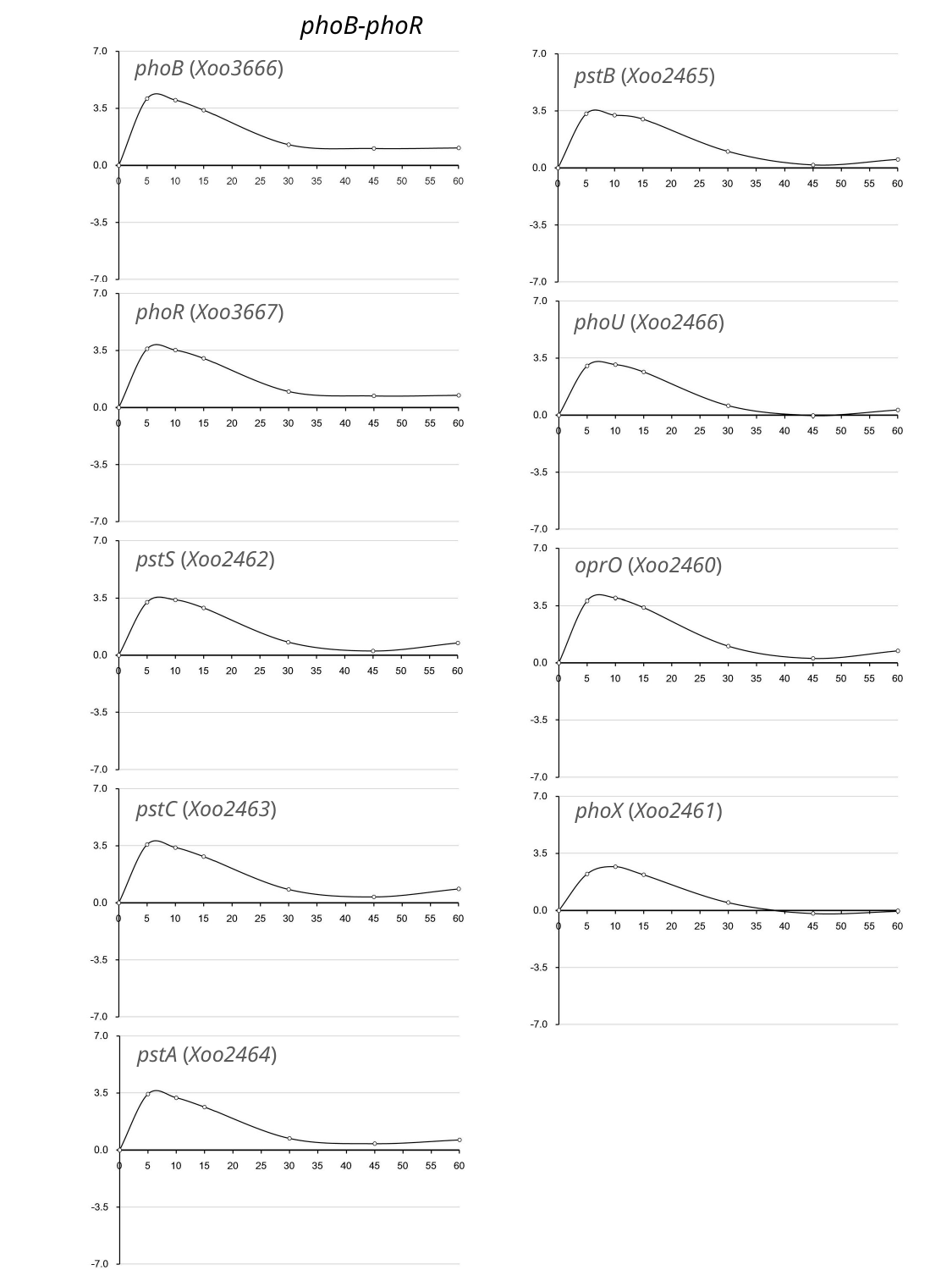

phoB-phoR
phoB (Xoo3666)
pstB (Xoo2465)
phoR (Xoo3667)
phoU (Xoo2466)
pstS (Xoo2462)
oprO (Xoo2460)
pstC (Xoo2463)
phoX (Xoo2461)
pstA (Xoo2464)

## Slide 7
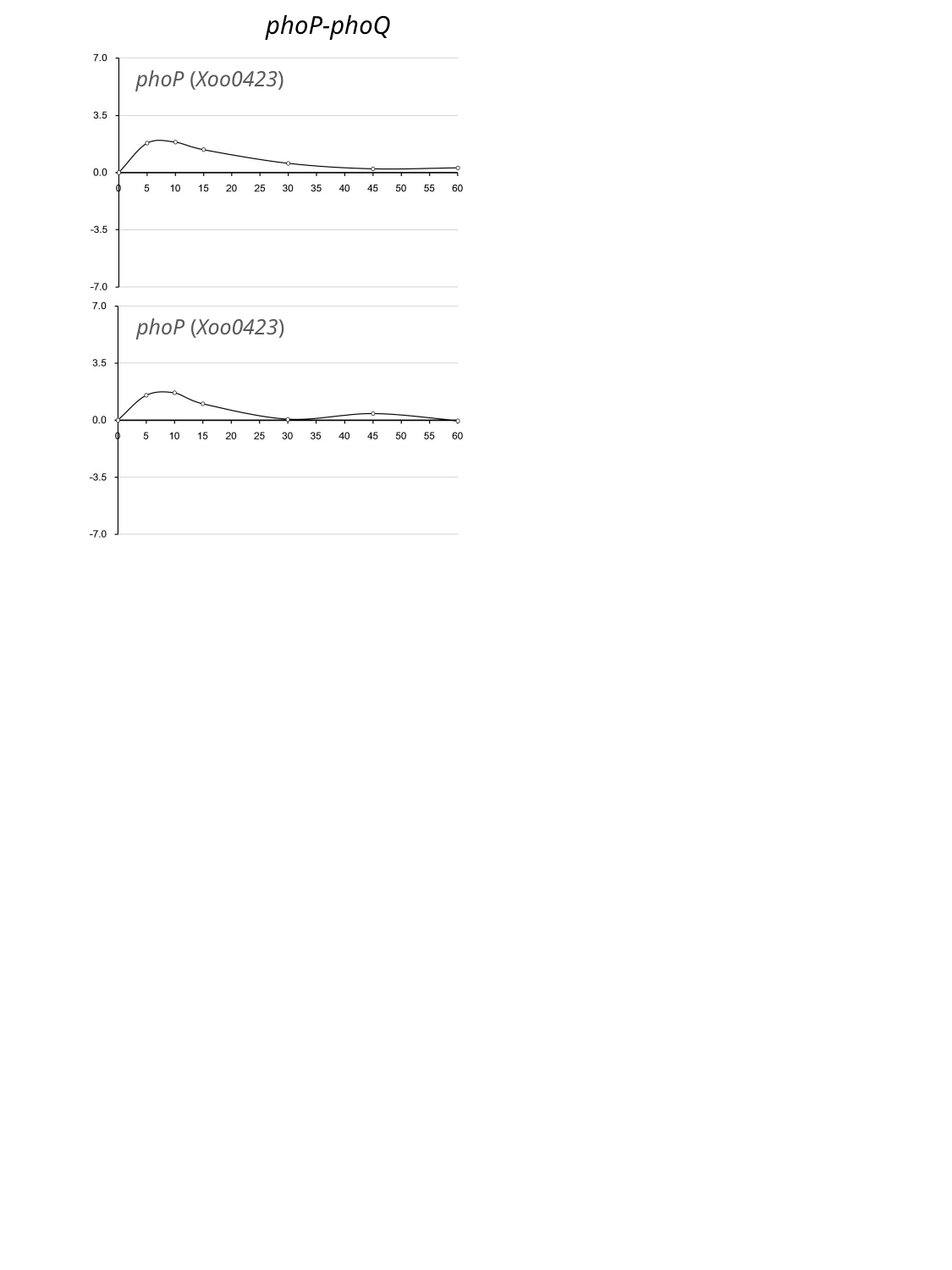

phoP-phoQ
phoP (Xoo0423)
phoP (Xoo0423)

## Slide 8
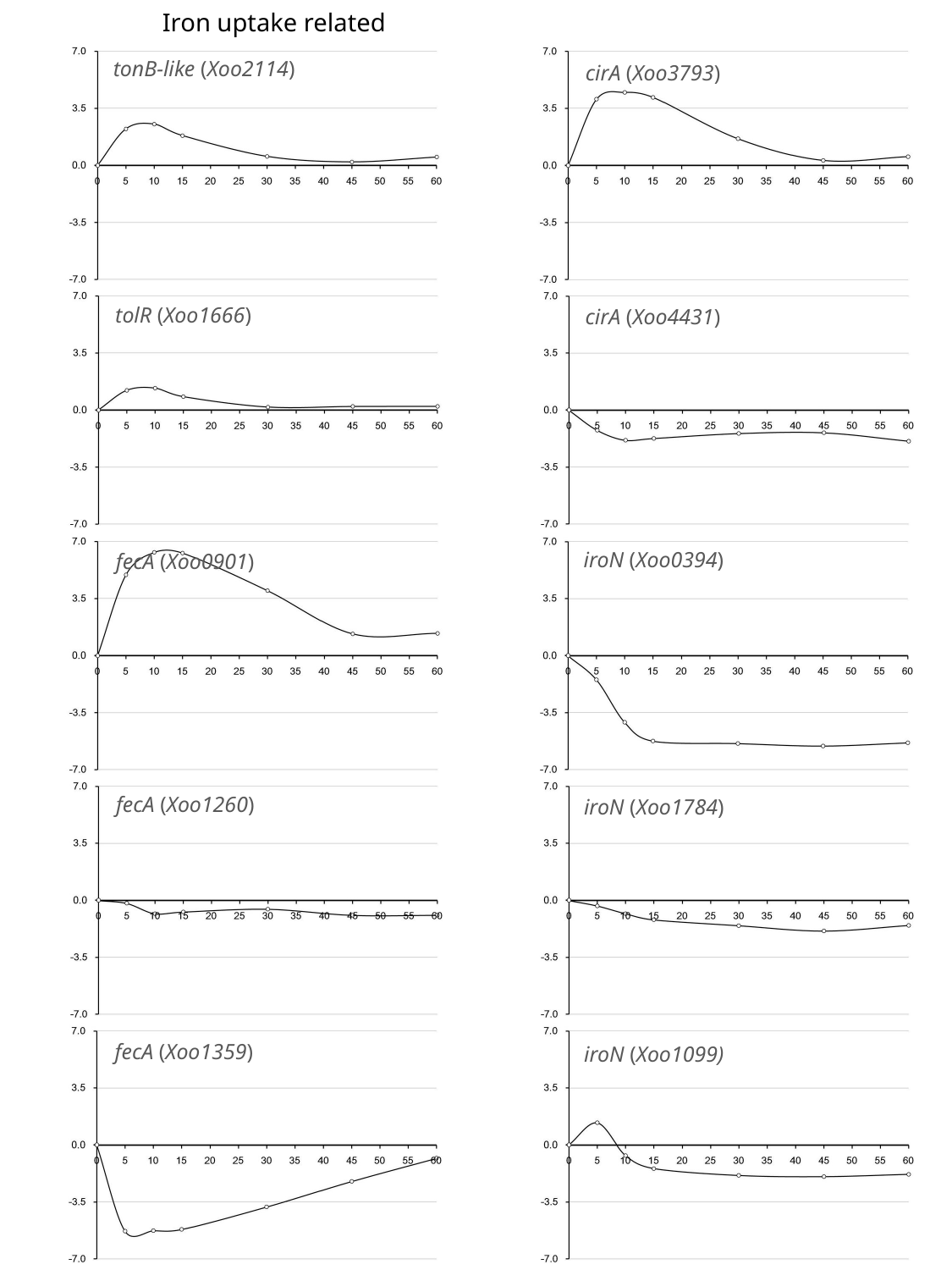

Iron uptake related
tonB-like (Xoo2114)
cirA (Xoo3793)
tolR (Xoo1666)
cirA (Xoo4431)
fecA (Xoo0901)
iroN (Xoo0394)
fecA (Xoo1260)
iroN (Xoo1784)
fecA (Xoo1359)
iroN (Xoo1099)

## Slide 9
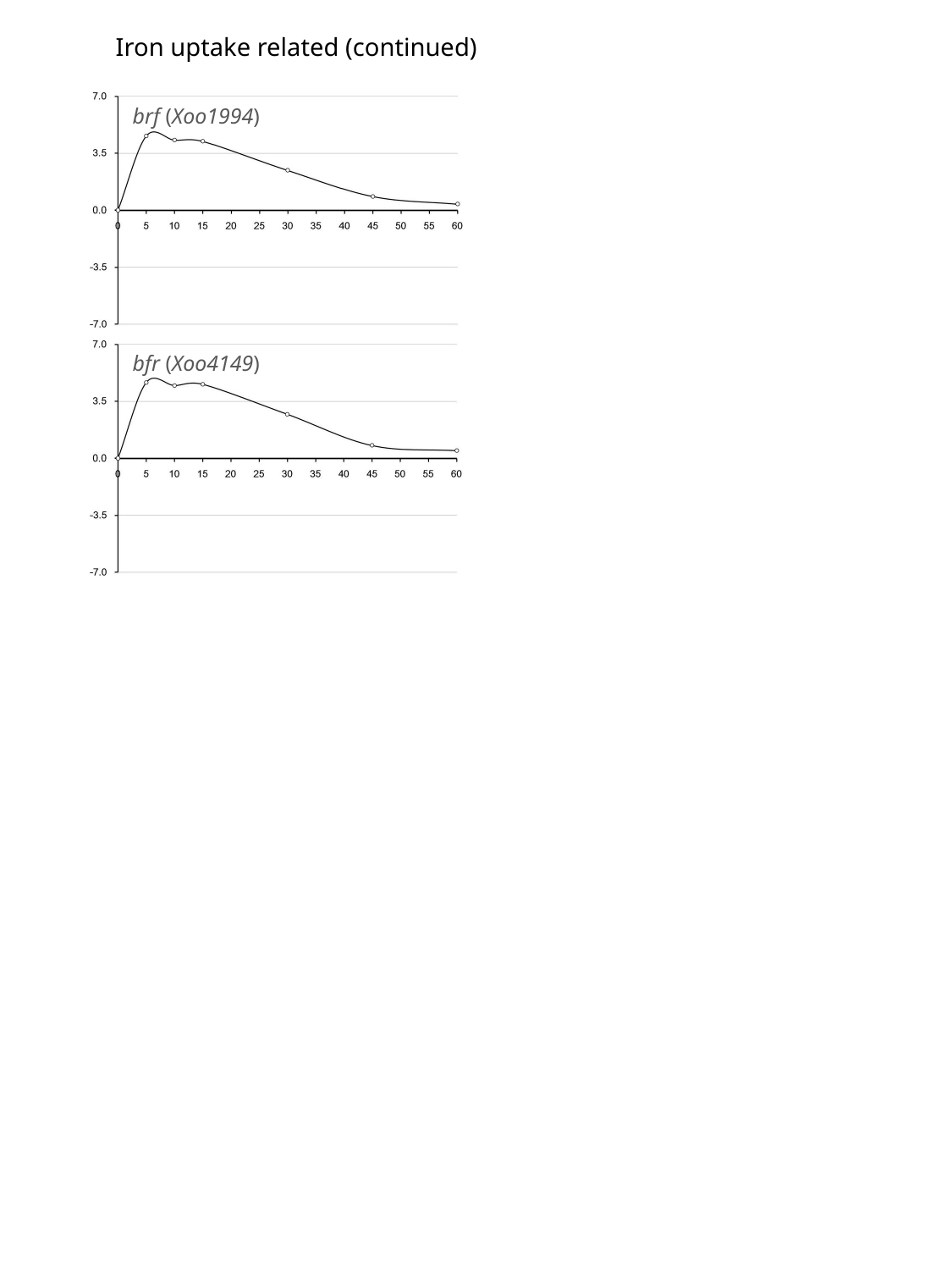

Iron uptake related (continued)
brf (Xoo1994)
bfr (Xoo4149)

## Slide 10
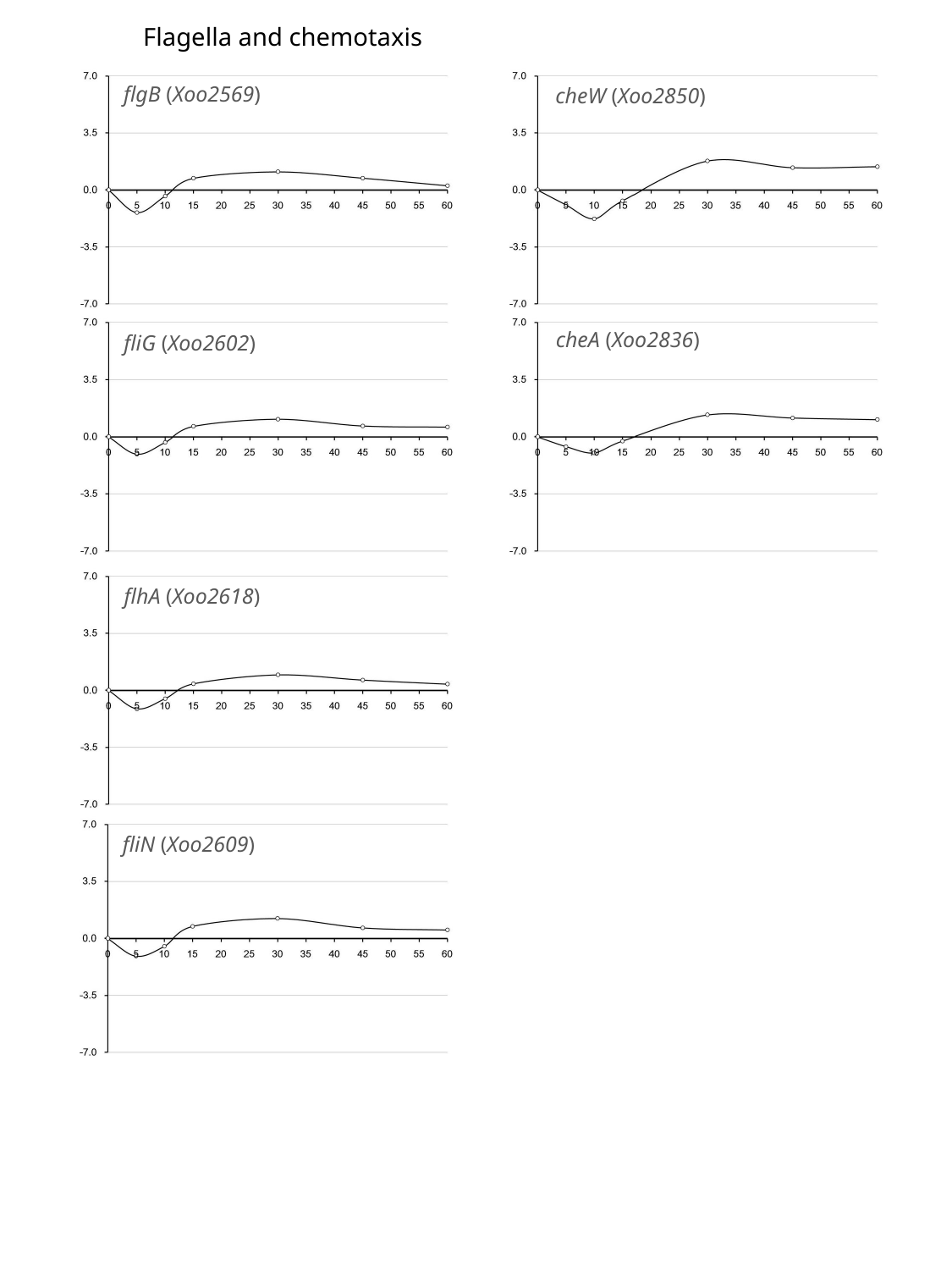

Flagella and chemotaxis
flgB (Xoo2569)
cheW (Xoo2850)
fliG (Xoo2602)
cheA (Xoo2836)
flhA (Xoo2618)
fliN (Xoo2609)

## Slide 11
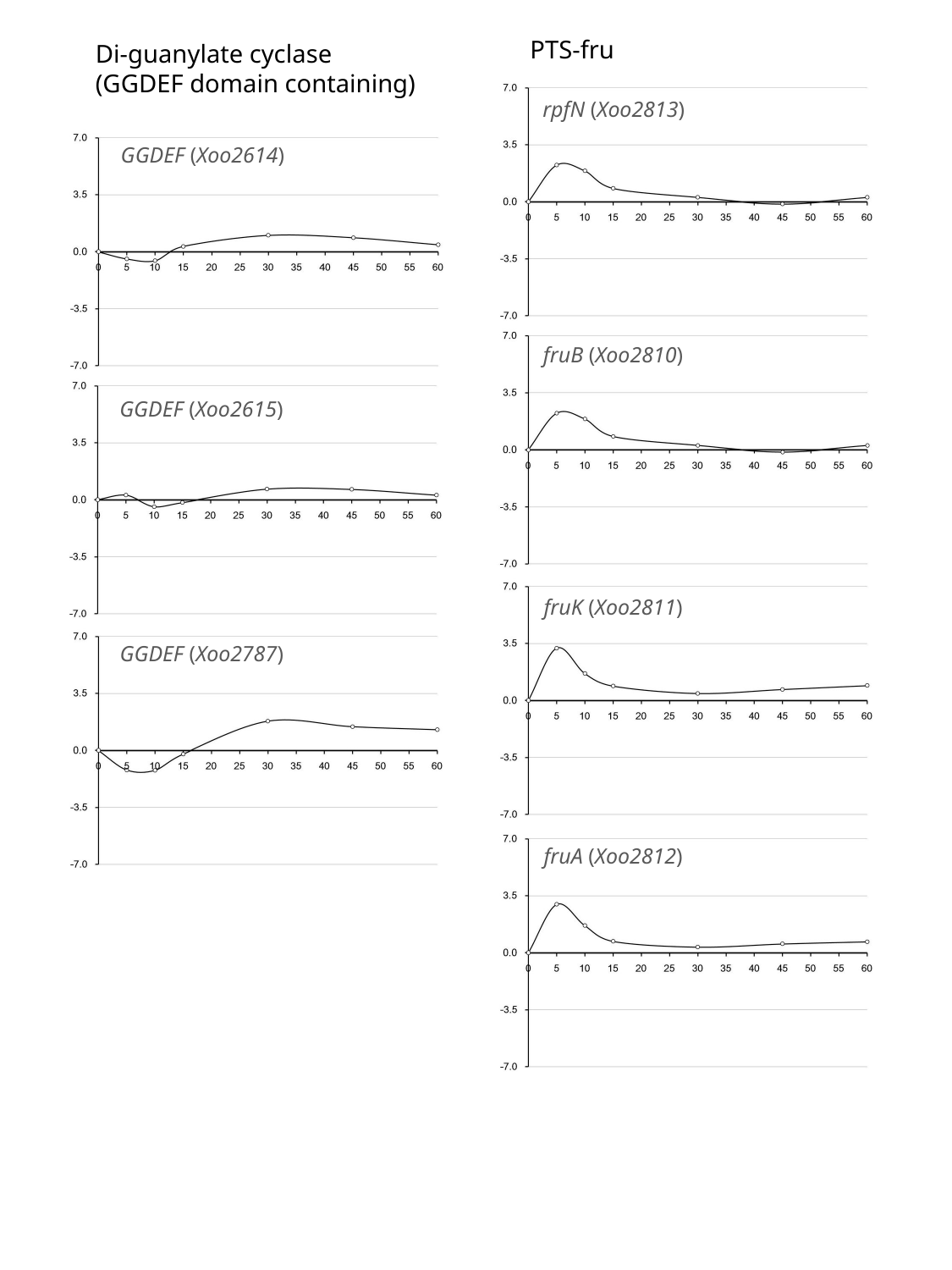

PTS-fru
Di-guanylate cyclase
(GGDEF domain containing)
rpfN (Xoo2813)
GGDEF (Xoo2614)
fruB (Xoo2810)
GGDEF (Xoo2615)
fruK (Xoo2811)
GGDEF (Xoo2787)
fruA (Xoo2812)

## Slide 12
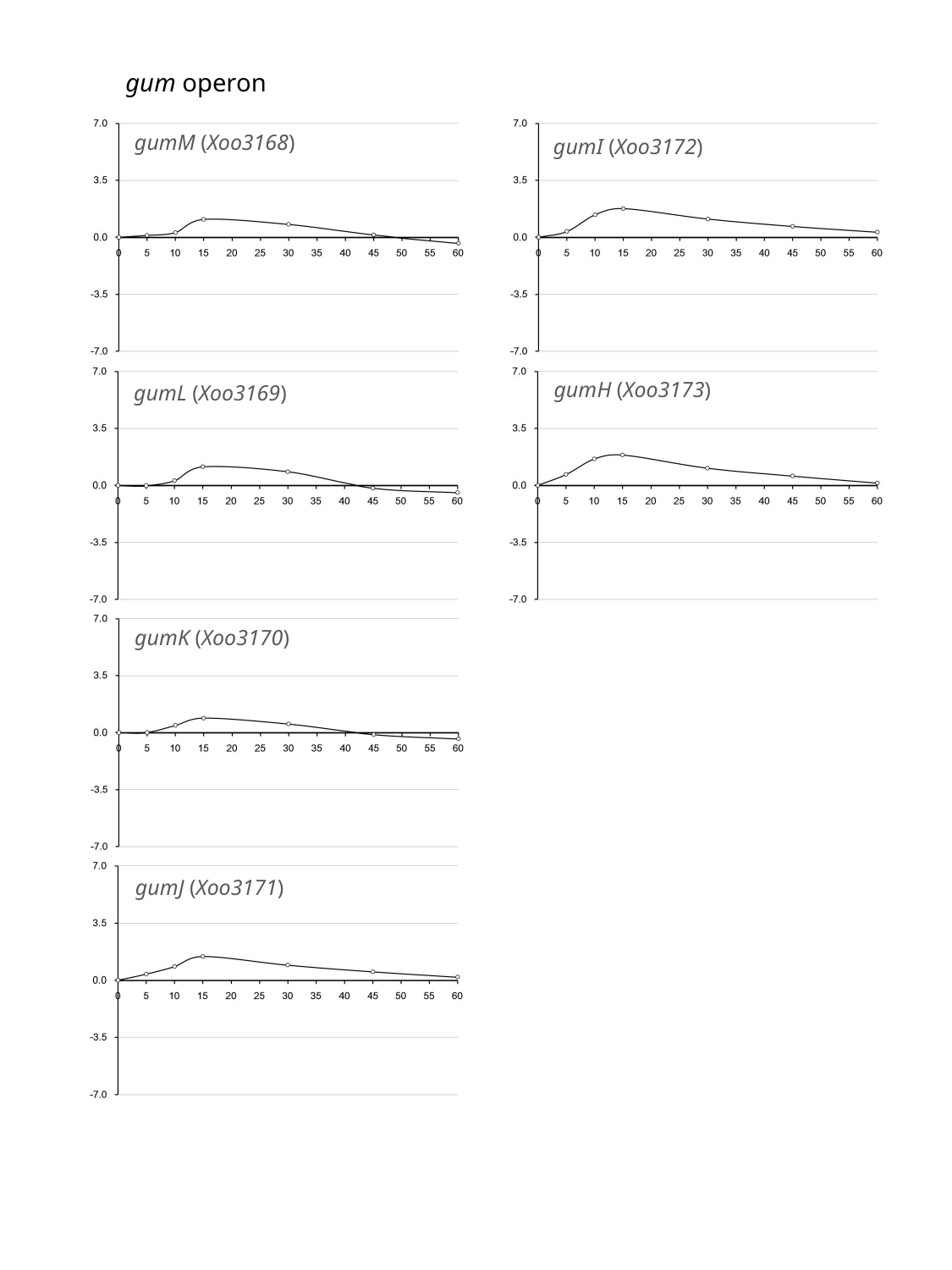

gum operon
gumM (Xoo3168)
gumI (Xoo3172)
gumL (Xoo3169)
gumH (Xoo3173)
gumK (Xoo3170)
gumJ (Xoo3171)

## Slide 13
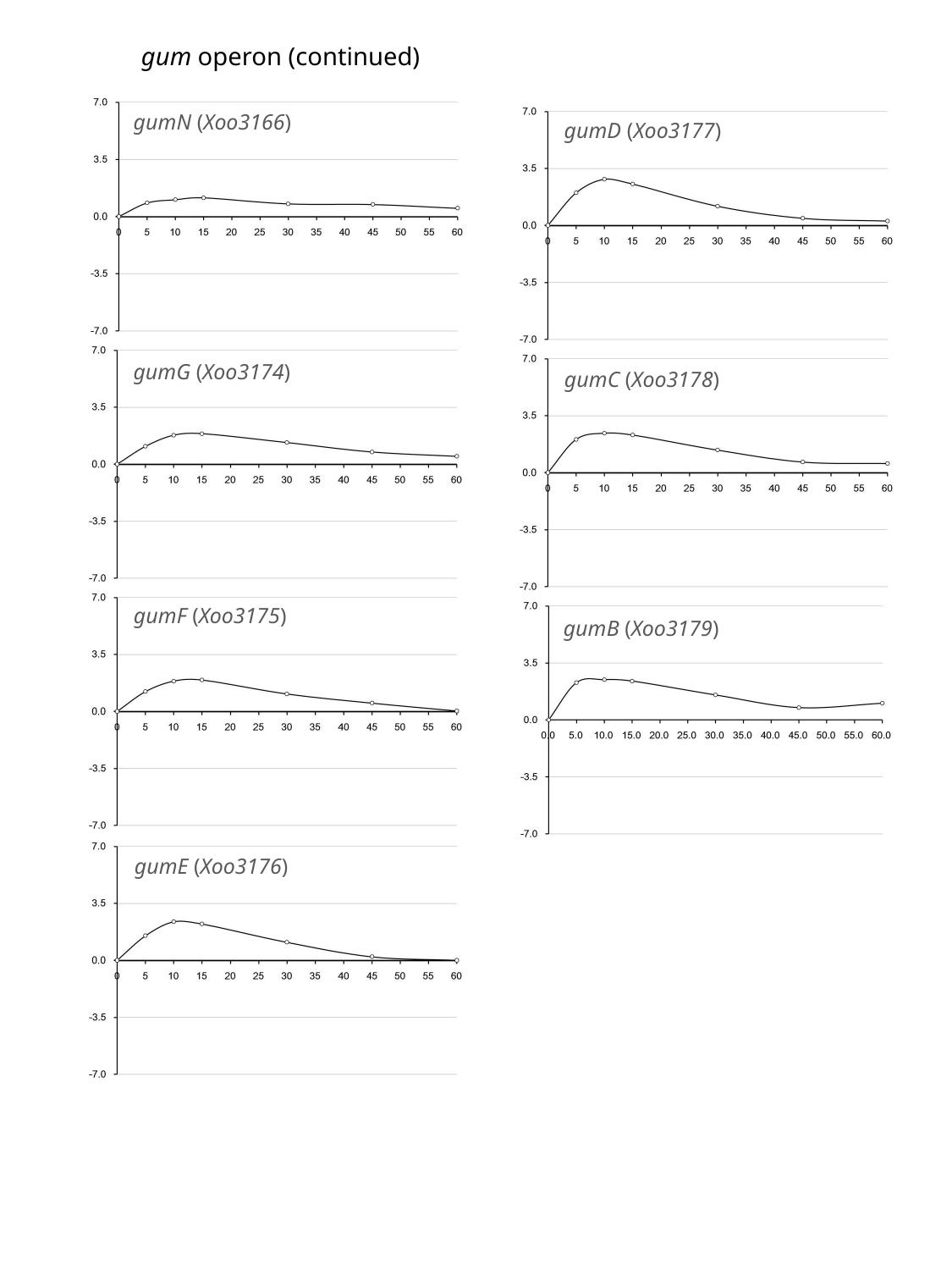

gum operon (continued)
gumN (Xoo3166)
gumD (Xoo3177)
gumG (Xoo3174)
gumC (Xoo3178)
gumF (Xoo3175)
gumB (Xoo3179)
gumE (Xoo3176)
